# Molecular profiling of BRAF-V600E-mutant metastatic colorectal cancer in the phase 3 BEACON CRC trial

**DOI:** 10.1038/s41591-024-03235-9

**Published:** 2024-09-23

**Authors:** Scott Kopetz, Danielle A. Murphy, Jie Pu, Fortunato Ciardiello, Jayesh Desai, Eric Van Cutsem, Harpreet Singh Wasan, Takayuki Yoshino, Hedieh Saffari, Xiaosong Zhang, Phineas Hamilton, Tao Xie, Rona Yaeger, Josep Tabernero

**Affiliations:** 1 University of Texas MD Anderson Cancer Center, Houston, TX USA; 2 Pfizer, La Jolla, CA USA; 3 Pfizer, New York, NY USA; 4 University of Campania Luigi Vanvitelli, Naples, Italy; 5 Peter MacCallum Cancer Centre, Melbourne, Victoria Australia; 6 University Hospitals Gasthuisberg Leuven and KU Leuven, Leuven, Belgium; 7 Division of Cancer, Hammersmith Hospital, Imperial College London, London, UK; 8 National Cancer Center Hospital East, Kashiwa, Japan; 9 Pfizer, South San Francisco, CA USA; 10 Memorial Sloan Kettering Cancer Center, New York, NY USA; 11 Vall d’Hebron Hospital Campus and Vall d’Hebron Institute of Oncology (VHIO), University of Vic—Central University of Catalonia, Barcelona, Spain

**Keywords:** Biomarkers, Medical research, Cancer, Genetics, Molecular biology

## Abstract

The BEACON CRC study demonstrated that encorafenib (Enco)+cetuximab (Cetux)±binimetinib (Bini) significantly improved overall survival (OS) versus Cetux + chemotherapy in previously treated patients with *BRAF*-V600E-mutant mCRC, providing the basis for the approval of the Enco+Cetux regimen in the United States and the European Union. A greater understanding of biomarkers predictive of response to Enco+Cetux±Bini treatment is of clinical relevance. In this prespecified, exploratory biomarker analysis of the BEACON CRC study, we characterize genomic and transcriptomic correlates of clinical outcomes and acquired resistance mechanisms through integrated clinical and molecular analysis, including whole-exome and -transcriptome tissue sequencing and circulating tumor DNA genomic profiling. Tumors with higher immune signatures showed a trend towards increased OS benefit with Enco+Bini+Cetux. *RAS*, *MAP2K1* and *MET* alterations were most commonly acquired with Enco+Cetux±Bini, and more frequent in patients with a high baseline cell-cycle gene signature; baseline *TP53* mutation was associated with acquired *MET* amplification. Acquired mutations were subclonal and polyclonal, with evidence of increased tumor mutation rate with Enco+Cetux±Bini and mutational signatures (SBS17a/b). These findings support treatment with Enco+Cetux±Bini for patients with *BRAF*-V600E-mutant mCRC and provide insights into the biology of response and resistance to MAPK-pathway-targeted therapy. ClinicalTrials.gov registration: NCT02928224

## Abstract (web summary)

Prespecified exploratory genomic and transcriptomic profiling of tumor tissues and circulating tumor DNA from patients with BRAF-V600E-mutant metastatic colorectal cancer who participated in the phase 3 BEACON CRC trial identifies biomarkers of response and mechanisms of acquired resistance to treatment with the BRAF inhibitor encorafenib plus the anti-EGFR antibody cetuximab, with or without the MEK inhibitor binimetinib.

## Main

*BRAF*-V600E mutations occur in approximately 10% of tumors in patients with mCRC and are associated with poor prognosis relative to wild-type *BRAF* tumors^1,2^. The BEACON CRC study (NCT02928224; Binimetinib, Encorafenib and Cetuximab Combined to Treat *BRAF*-Mutant Colorectal Cancer) demonstrated that the BRAF inhibitor Enco plus the anti-epidermal growth factor receptor (EGFR) antibody Cetux, with or without the MEK inhibitor Bini, improved OS, objective response rate and progression-free survival (PFS) compared with Cetux plus chemotherapy in patients with *BRAF*-V600E-mutant mCRC who had been previously treated^3,4^. A greater understanding of the *BRAF*-mutant (BM) biological landscape and prognostic and predictive determinants is important for optimizing therapy.

Transcriptional profiling of CRC tumors has identified subtypes that have similar biological characteristics. These include the consensus molecular subtypes (CMS) and BM subtypes^5,6^. These subtypes may have utility in predicting prognosis and treatment response in patients with CRC^5,7^. Previously treated patients with *BRAF*-V600E-mutant CRC have shown acquired resistance alterations in the MAPK pathway and amplifications in *MET* following treatment with BRAF inhibitor combinations^8–10^. To our knowledge, clinical associations with molecular subtypes and mechanisms of acquired resistance following treatment with a BRAF inhibitor plus an anti-EGFR antibody, with or without a MEK inhibitor, versus conventional cytotoxic chemotherapy plus an anti-EGFR antibody, have not been studied in large, randomized trials.

Through genomic and transcriptional profiling of tumor tissue and genomic profiling of plasma samples obtained from the BEACON CRC study cohort, we characterized the molecular landscape of *BRAF*-V600E-mutant mCRC treated with Enco+Cetux±Bini versus Cetux plus chemotherapy to understand the evolving biology of these tumors on treatment. The objective of this analysis was to identify molecular correlates of clinical outcomes and define resistance mechanisms acquired following treatment. The integrated tumor and plasma molecular profiling analyses performed in this study provide comprehensive data from the largest randomized study in *BRAF*-V600E-mutant CRC.

## Results

### BEACON CRC biomarker analysis set

**Fig. 1:**
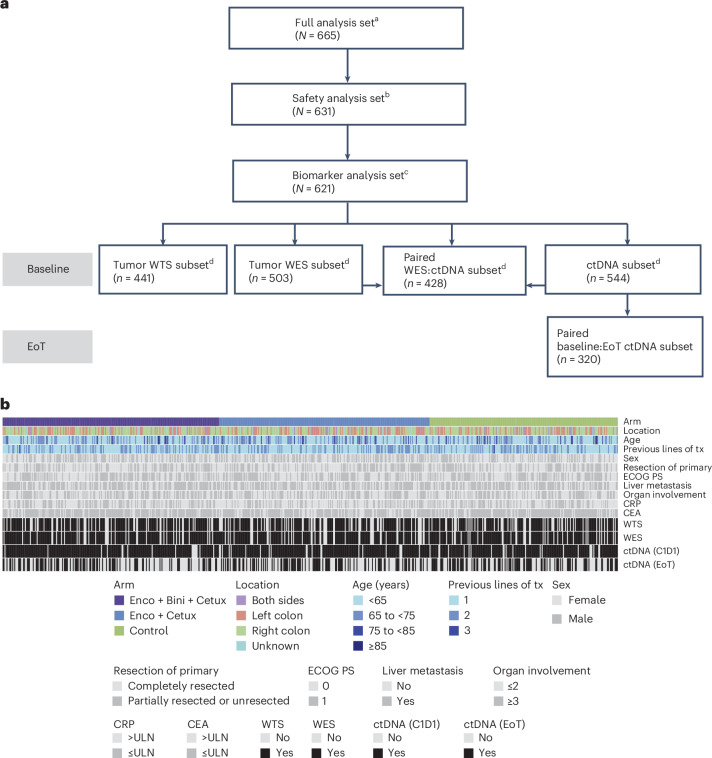
Study cohorts for BEACON CRC biomarker analyses. **a**, Patient flow in analysis sets. ^a^Full analysis set included patients who were enrolled to receive randomized treatment. ^b^Safety analysis set included patients who received one or more dose of any trial drug and had one or more post-treatment safety assessment. ^c^Biomarker analysis set included patients who had baseline tumors analyzed by WTS or WES or who were analyzed for ctDNA at baseline. ^d^Biomarker analysis subsets were not mutually exclusive. **b**, Heatmap of patient characteristics and molecular profiling coverage in the biomarker analysis set, across treatment arms. CEA, carcinoembryonic antigen; CRP, C-reactive protein; ECOG PS, Eastern Cooperative Oncology Group performance status; tx, treatment; ULN, upper limit of normal.

Of the 665 patients enrolled in the full analysis set, 621 (93.4%) were included in the biomarker analysis set (Fig. 1a). To assess the molecular landscape of baseline tumors, we analyzed 503 patients (81.0%) by whole-exome sequencing (WES) and 441 (71.0%) by whole transcriptome sequencing (WTS). For assessment of the genomic landscape by circulating tumor DNA (ctDNA) profiling, we analyzed 544 patients (87.6%) at baseline and 320 (51.5%) with paired end-of-treatment (EoT) samples. Baseline biomarker analyses were performed in a representative patient population (Fig. 1b). The number of patients was balanced among the three treatment arms (Enco+Bini+Cetux, Enco+Cetux and control) for all biomarker analysis subsets (Supplementary Table 1).

### Baseline molecular profiling of patients

**Fig. 2:**
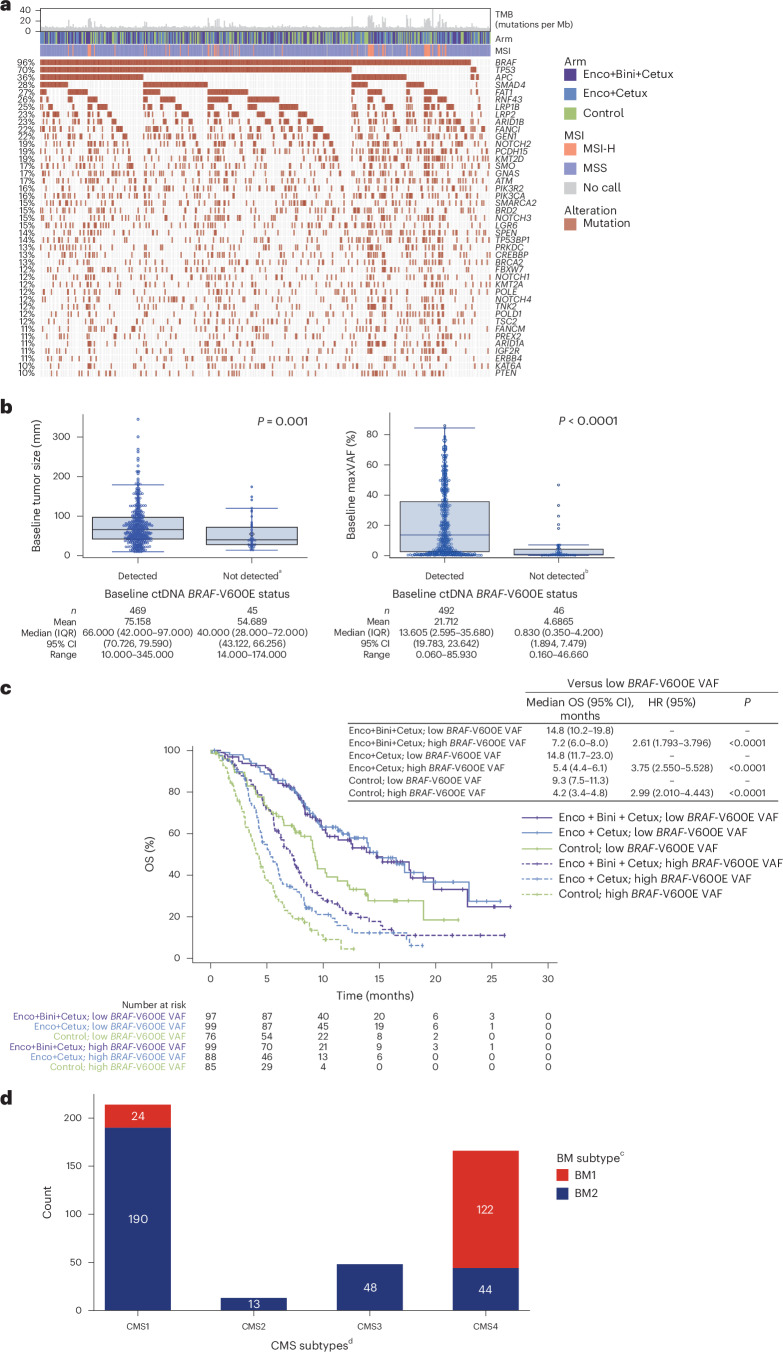
Mutational profiling of patients with BRAF-V600E-mutant mCRC in BEACON CRC. **a**, Oncoprint showing frequency of cancer gene mutations in baseline tumors profiled by the GuardantOMNI panel, as well as TMB and MSI status (top bars), across treatment arms determined using WES. *BRAF* alterations included V600E and other alterations. The MSS group also included patients who were MSI-low. **b**, Baseline tumor size (left panel) and baseline ctDNA maxVAF (right panel) by baseline ctDNA *BRAF*-V600E status. *P* values were based on the Wilcoxon rank-sum test (two-sided). The boxes show medians and IQR; the whiskers represent 1.5 × IQR. **c**, Kaplan–Meier plots of OS by ctDNA *BRAF-*V600E VAF status (split on median) in each treatment arm. *P* values (two-sided Wald test) were based on the Cox model without adjustment for baseline covariates. **d**, Distribution of BM1 and BM2 subtypes across CMS subgroups. ^a^The ‘not detected’ group included patients with no ctDNA detected. ^b^Patients with no ctDNA detected were excluded from the analysis. ^c^The BM1 subtype is characterized by high KRAS/mTOR/AKT/4EBP1, epithelial–mesenchymal transition (EMT) and immune infiltration, whereas the BM2 subtype is characterized by cell-cycle checkpoint dysregulation^5^. ^d^CMS1 is characterized by MSI and immune patterns and enrichment for *BRAF* mutations; CMS2 by chromosomal instability and WNT activation; CMS3 by metabolic pattern and enrichment for *KRAS* mutations and CMS4 by EMT, angiogenesis and stromal infiltration^5^.

**Extended Data Fig. 1:**
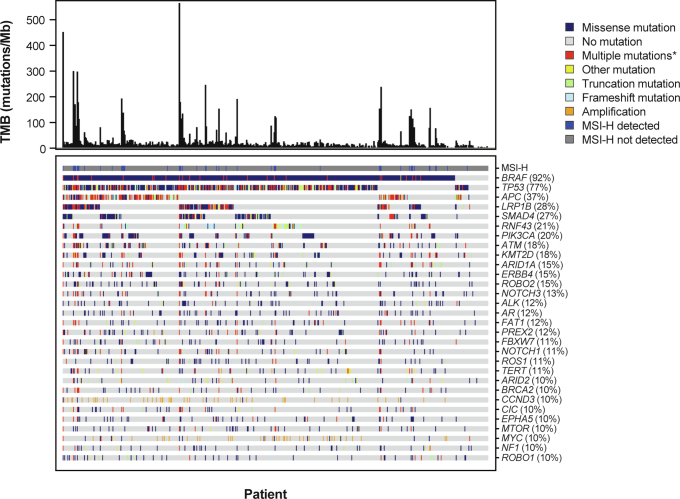
Genomic ctDNA profiling of baseline plasma samples. *Multiple mutations were defined as genes with ≥ 2 alterations of different categories (for example missense mutation + amplification). ctDNA, circulating tumor DNA; MSI-H, microsatellite instability-high; mut; mutation; TMB, tumor mutational burden.

Genomic profiling of baseline tumor tissue and ctDNA showed that *BRAF*-V600E was detected by WES and ctDNA in 476 of 503 (94.6%) and 492 of 544 patients (90.4%) analyzed, respectively (Fig. 2a, Extended Data Fig. 1 and Supplementary Table 2). Of the patients who had genomic profiling performed on both tumor and ctDNA samples, 368 of 404 (91.1%) had *BRAF*-V600E detected in both (Supplementary Table 3).

**Extended Data Fig. 2:**
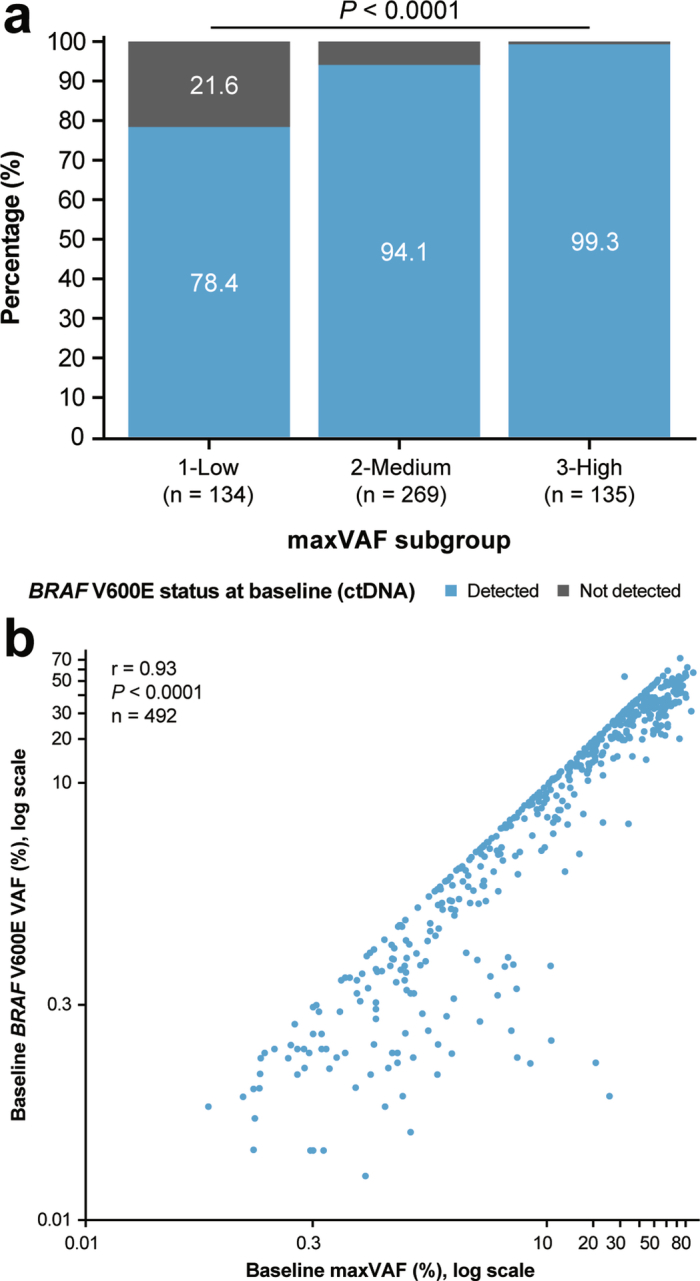
Association between *BRAF* V600E VAF and ctDNA maxVAF at baseline. **a**, Percentages of baseline *BRAF* V600E positivity across ctDNA maxVAF subgroups. The low maxVAF subgroup included patients with ctDNA maxVAF below the first quartile (2.01%); the high maxVAF subgroup included patients with ctDNA maxVAF in the third quartile (33.77%) and above; the medium maxVAF subgroup included the remaining patients. P-value was calculated based on logistic regression analysis (two-sided Wald test). **b**, Pearson correlation and the associated two-sided P value of the association between *BRAF* V600E VAF and ctDNA maxVAF at baseline. Only patients with *BRAF* V600E ctDNA detected were included in the analysis involving VAF. Silent mutations, clonal hematopoiesis of indeterminate potential, and alterations from genes with copy number alterations were excluded. ctDNA, circulating tumor DNA; max, maximum; VAF, variant allele frequency.

Compared with patients who had detectable *BRAF*-V600E by ctDNA genomic profiling, those who had undetectable *BRAF*-V600E had smaller baseline tumor sizes (median (interquartile range; IQR) 40.00 mm (28.00–72.00) versus 66.00 mm (42.00–97.00); *P* = 0.001) and lower baseline ctDNA maximum variant allele frequency (maxVAF) (0.830% (0.350–4.200) versus 13.605% (2.595–35.680); *P* < 0.0001) (Fig. 2b). Consistent with this finding, when maxVAF was below the first quartile (2.01%), the percentage of patients with detectable *BRAF*-V600E was 78.4%; when maxVAF was in the third quartile (33.77%) and above, the percentage increased to >99% (*P* < 0.0001) (Extended Data Fig. 2a). maxVAF by ctDNA genomic profiling correlated with *BRAF*-V600E VAF (*r* = 0.93; *P* < 0.0001; Extended Data Fig. 2b). Patients with liver metastases at baseline had higher *BRAF*-V600E VAF than those who did not (*P* < 0.0001), whereas *BRAF*-V600E VAF did not differ between patients with three or more involved organs and those with fewer involved organs (*P* = 0.25) (Supplementary Fig. 1a,b).

**Extended Data Fig. 3:**
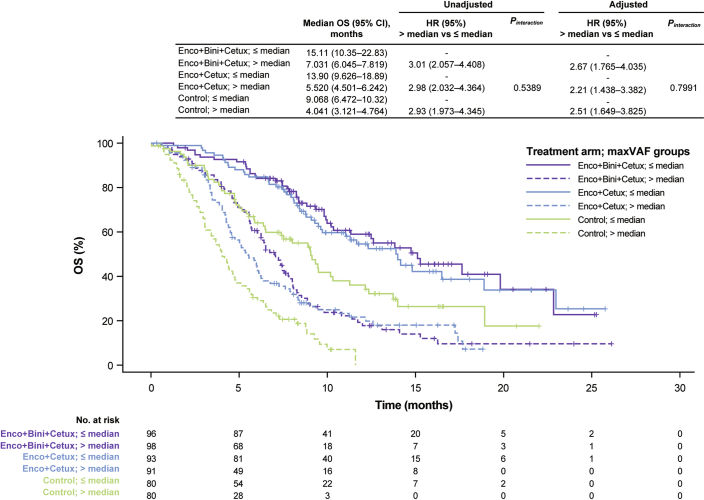
Kaplan–Meier plots of OS by ctDNA maxVAF status in each treatment arm. ctDNA maxVAF was categorized as ≤ or > the median of 11.005. Cox proportional hazard models with or without adjustment of baseline covariates (ECOG performance status, C-reactive protein, number of organs, tumor status, cetuximab source, and prior use of irinotecan at randomization) were used. A two-sided Wald test was used for P value of interaction terms, where ctDNA maxVAF was regarded as a continuous variable. Bini, binimetinib; Cetux, cetuximab; CI, confidence interval; ctDNA, circulating tumor DNA; ECOG, Eastern Cooperative Oncology Group; Enco, encorafenib; HR, hazard ratio; max, maximum; OS, overall survival; VAF, variant allele frequency.

Evaluation of *BRAF*-V600E VAF levels in ctDNA at baseline showed that, in all treatment arms, OS was longer in patients with low (≤median (7.385%)) *BRAF*-V600E VAF than in those with high (>median) *BRAF-*V600E VAF (*P* < 0.0001 for all arms) (Fig. 2c). maxVAF by ctDNA genomic profiling showed similar associations with OS in all treatment arms (Extended Data Fig. 3). Overall, results were similar with or without adjustments for baseline covariates (Extended Data Fig. 3).

The cancer-associated genes comutated most frequently in tumor tissue were *TP53* (70%), *APC* (36%), *SMAD4* (28%), *FAT1* (27%), *RNF43* (26%) and *LRP1B* (25%) (Fig. 2a). Microsatellite instability (MSI)-high (MSI-H) was detected in 11.1% of patients (Supplementary Table 2). The median tumor mutational burden (TMB) was 7.740 mutations (mut)/Mb, and 15.7% of patients had a TMB of ≥10 mut/Mb (Supplementary Table 2). ctDNA genomic profiling showed similar frequently mutated cancer-associated genes; MSI-H was detected in 8.8% of patients and median TMB was 11.480 mut/Mb across all arms (Extended Data Fig. 1 and Supplementary Table 2).

Tumors were classified into CMS and BM subtypes by transcriptomic analyses. Most patients analyzed had either CMS1 (214/441; 48.5%) or CMS4 (166/441; 37.6%) tumors; 33.1% (146/441) of patients were classified as having the BM1 subtype, which was observed predominately in those with CMS4 tumors (122/166; 73.5%) (Fig. 2d).

### Associations of CRC subtypes and biomarkers with OS benefit

**Extended Data Fig. 4:**
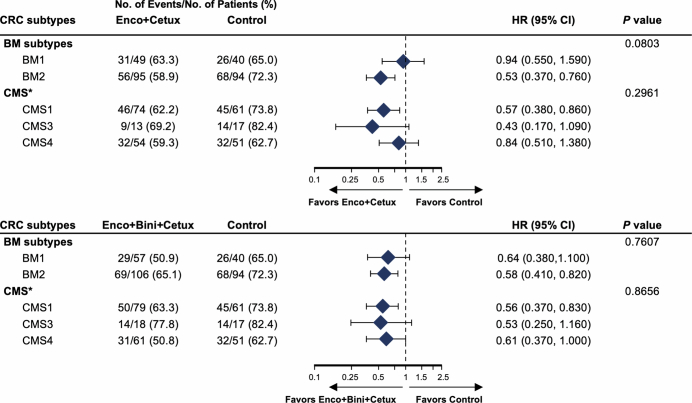
Association between OS and CRC subtypes. Forest plots of HRs for OS with Enco+Cetux (top panel) or Enco+Bini+Cetux (bottom panel) vs control in subgroups defined by BM subtypes and CMS. The diamonds show HRs and the lines show 95% CI. P values for interaction (two-sided Wald test) were based on the Cox model without adjustment for baseline covariates. *The CMS2 subgroup was excluded due to small sample size.

We examined the association between OS benefit and the CMS and BM subtypes in the BEACON CRC patient population. OS benefit with Enco+Cetux versus control was observed irrespective of BM subtype (*P*_interaction_ = 0.0803) or CMS (*P*_interaction_ = 0.2961), with a trend towards increased benefit in the BM2 and CMS1 subgroups (Extended Data Fig. 4 and Supplementary Fig. 2a (adjusted analyses)). Similarly, OS benefit with Enco+Bini+Cetux versus control was observed across the cohort with no differences according to BM subtype (*P*_interaction_ = 0.7607) or CMS (*P*_interaction_ = 0.8656) (Extended Data Fig. 4 and Supplementary Fig. 2a (adjusted analyses)).

**Fig. 3:**
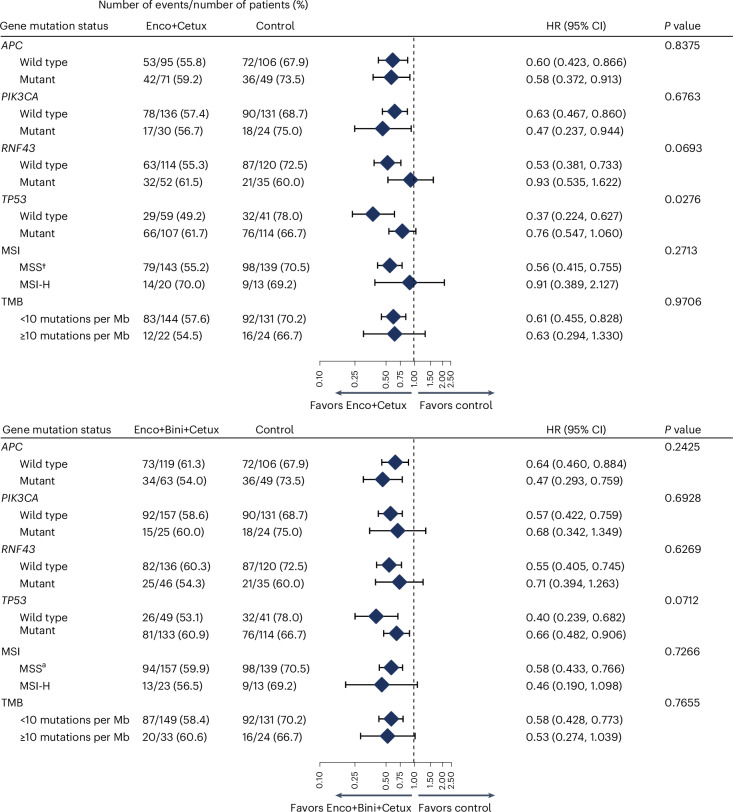
Association between OS tumor baseline mutational status. Forest plots of HRs for OS with Enco+Cetux (top panel) or Enco+Bini+Cetux (bottom panel) versus control in subgroups defined by specific gene mutations, MSI status or TMB levels. The diamonds show HRs and the lines show 95% CI. *P* values for interaction (two-sided Wald test) were based on the Cox model without adjustment for baseline covariates. ^a^The MSS subgroup included patients with MSI-low; those with inconclusive results were excluded from the analysis.

We examined whether the mutational status of the most frequently comutated genes in CRC (*APC*, *PIK3CA*, *RNF43* and *TP53*), MSI status or TMB level was associated with differences in OS in patients treated with either Enco+Bini+Cetux or Enco+Cetux compared with control (Fig. 3 and Supplementary Fig. 2b (adjusted analyses)). Patients with wild-type *TP53* showed greater OS benefit with Enco+Cetux (hazard ratio (HR), 0.37 (95% confidence interval (CI), 0.224–0.627) for wild type and 0.76 (95% CI, 0.547–1.060) for mutant; *P*_interaction_ = 0.0276), with a similar trend observed for the OS difference in the Enco+Bini+Cetux versus control arms (*P*_interaction_ = 0.0712). We did not observe differences in OS benefit in subgroups defined by other specific gene mutations, MSI-H or TMB. Specifically, OS benefit was observed in patients treated with Enco+Cetux±Bini regardless of *RNF43* mutational status (Enco+Cetux: unadjusted HR, 0.53 (95% CI, 0.381–0.733) for wild type and 0.93 (95% CI, 0.535–1.622) for mutant; *P*_interaction_ = 0.0693 and Enco+Bini+Cetux: unadjusted HR, 0.55 (95% CI, 0.405–0.745) for wild type and 0.71 (95% CI, 0.394–1.263) for mutant; *P*_interaction_ = 0.6269) (Fig. 3 and Supplementary Fig. 2b). Subgroup analyses showed similar OS findings by *RNF43* mutational status in MSS patients (Supplementary Table 4).

### Immune associations with OS benefit from Enco+Cetux±Bini

**Fig. 4:**
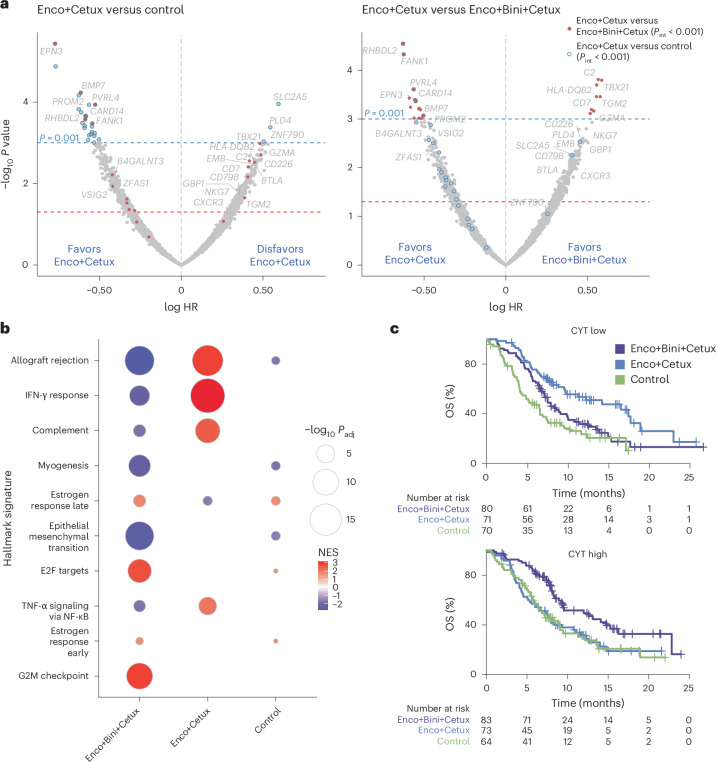
Immune gene associations with OS benefit from Enco+Cetux±Bini. **a**, Associations between OS and immune gene expression (scaled) were determined by Cox proportional hazards models (two-sided Wald test). Genes in the top quartile of variance in expression (based on IQR and measured in log_2_(TPM)) were tested. **b**, GSEA hallmark genesets; highest average absolute normalized enrichment score (NES) across arms are shown; point size indicates Benjamini–Hochberg-adjusted *P* values from permutation testing. **c**, Kaplan–Meier plots of OS by cytolytic score (split on median). *P* values for interaction (two-sided Wald test) were based on Cox proportional hazard models with and without adjustment for baseline covariates.

**Extended Data Fig. 5:**
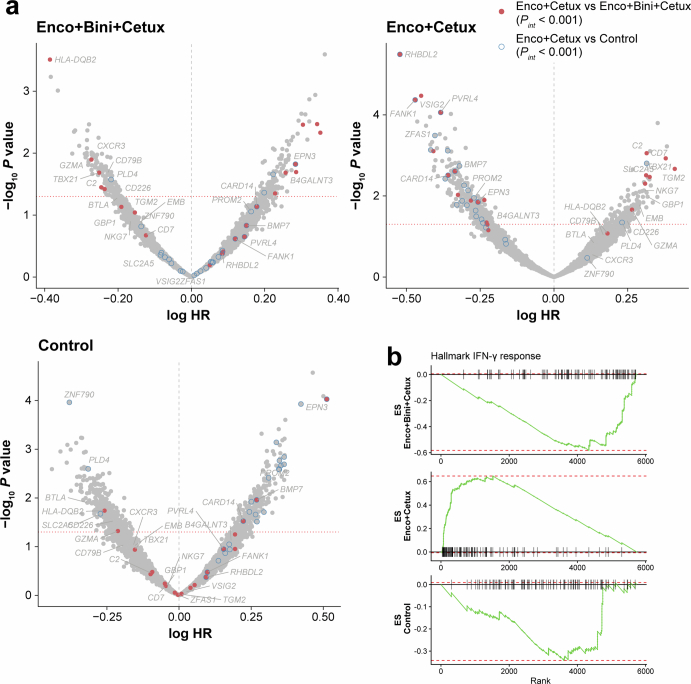
Immune gene associations with OS benefit in each treatment arm. **a**, Associations between OS and immune gene expression (scaled) in each treatment arm were determined by Cox proportional hazards models (two-sided Wald test). Genes in the top quartile of variance in expression (based on IQR and measured in log_2_[TPM]) were tested. Genes showing an interaction (unadjusted P value < 0.001) for Enco+Cetux vs Control are shown with blue circles and for Enco+Cetux vs Enco+Bini+Cetux (top panel) are shown as red dots across panels. **b**, Enrichment plots from GSEA against the hallmark IFN-γ gene signature in each treatment arm.

To evaluate gene expression profiles predictive of OS benefit from Enco+Bini+Cetux or Enco+Cetux, we used Cox models to test for interactions between univariable gene expression and treatment arm (Fig. 4a). The expression of genes linked to T-cell biology and immune processes (for example, *TBX21*, *GZMA*, *TLR7*, *CD7*)^11–14^ was strongly enriched in patients who had an OS benefit from Enco+Bini+Cetux, more modestly from control and in those who had decreased OS benefit from Enco+Cetux (all *P*_interaction_ < 0.001 for Enco+Cetux versus Enco+Bini+Cetux; *P*_interaction_ < 0.001 for Enco+Cetux versus control) (Fig. 4a and Extended Data Fig. 5a). Univariable tests of gene expression associations with OS in each arm (Fig. 4a), followed by gene set enrichment analysis (GSEA) against hallmark genesets, confirmed these observations (Fig. 4b and Extended Data Fig. 5b), revealing that immune processes (for example, interferon (IFN)-γ signaling, allograft rejection) were enriched most strongly in patients who had prolonged OS in the Enco+Bini+Cetux arm and in those who had shorter OS in the Enco+Cetux arm (Supplementary Fig. 3).

**Extended Data Fig. 6:**
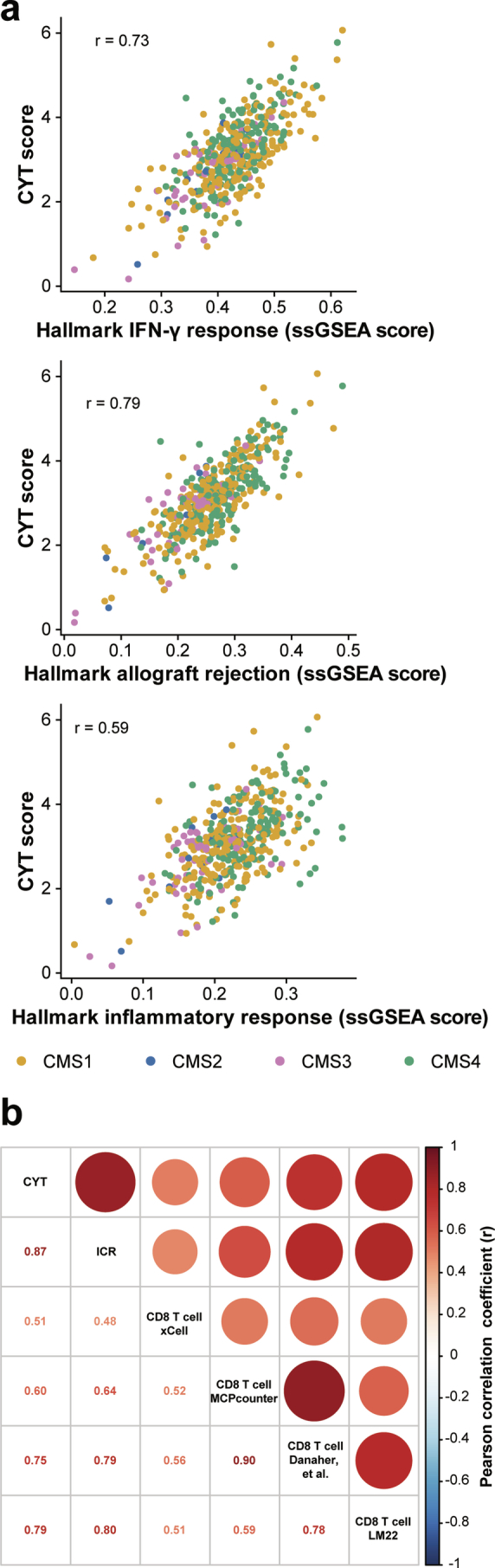
Correlation between tumor immune status and cytolytic score. **a**, Correlation between cytolytic score and ssGSEA scores for the IFN-γ response (top panel), allograft rejection (middle panel) and inflammatory response (bottom panel) hallmark signatures; r denotes Pearson correlation. Data points are color coded based on CMS classification. **b**, Pearson correlation between cytolytic score and sample scores for multiple metrics of CD8^+^ T-cell infiltration and gene signatures of immune infiltration. CMS, consensus molecular subtypes; CYT, cytolytic; ssGSEA, single-sample gene set enrichment analysis; ICR, immune constant of rejection.

**Extended Data Fig. 7:**
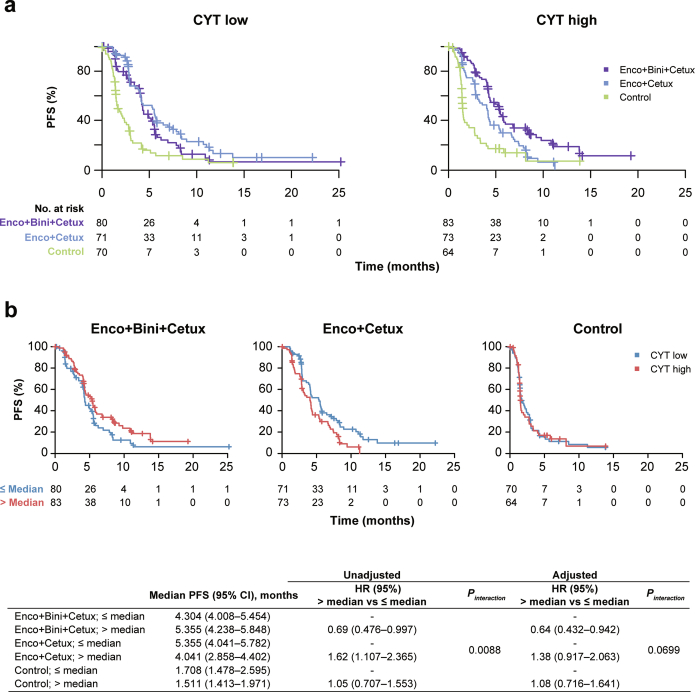
Kaplan–Meier plots of PFS by cytolytic score between and within each treatment arm. **a**, Kaplan–Meier plots of PFS by cytolytic score between treatment arms (inter-arm comparison). **b**, Kaplan–Meier plots of PFS by cytolytic score within each treatment arm (intra-arm comparison). Tumors were categorized as cytolytic high or low, defined as score > and ≤ the median value of 3.11, respectively. Cox proportional hazard models with or without adjustment of baseline covariates (ECOG performance status, C-reactive protein, number of organs, tumor status, cetuximab source, and prior use of irinotecan at randomization) were used. A two-sided Wald test was used for P value of interaction terms, where cytolytic score was regarded as a continuous variable. Bini, binimetinib; Cetux, cetuximab; CYT, cytolytic; ECOG, Eastern Cooperative Oncology Group; Enco, encorafenib; HR, hazard ratio; PFS, progression-free survival.

**Extended Data Fig. 8:**
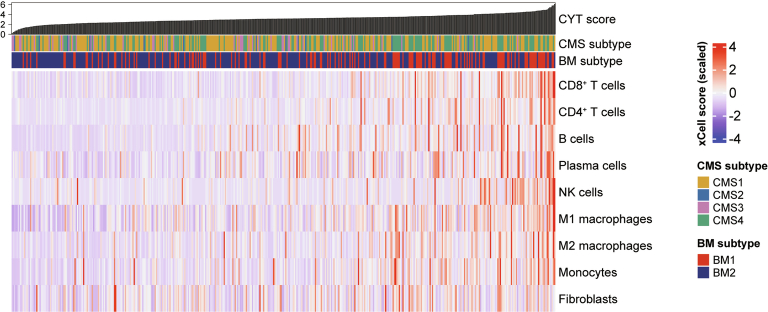
Abundance of immune cell types and fibroblasts (estimated using xCell) by CYT score and CRC subtype. Bini, binimetinib; BM, *BRAF* mutant; Cetux, cetuximab; CMS, consensus molecular subtypes; CRC, colorectal cancer; CYT, cytolytic; Enco, encorafenib; HR, hazard ratio; IQR, interquartile range; NES, normalized enrichment score; NK, natural killer; OS, overall survival; TPM, transcripts per million.

To evaluate the association between a simplified metric of tumor immune status and OS, we applied a cytolytic score used widely to measure the immune status of the tumor microenvironment (mean of log *GZMA* and *PRF1* expression)^13^ to characterize tumors as cytolytic high (>median of 3.11 log_2_(transcripts per million (TPM)) or cytolytic low (≤median). The cytolytic score was correlated with single-sample GSEA scores for the hallmark IFN-γ response (*r* = 0.73; *P* < 0.001), allograft rejection (*r* = 0.79; *P* < 0.001) and inflammatory response (*r* = 0.59; *P* < 0.001) signatures (Extended Data Fig. 6a), as well as sample scores for multiple metrics inferring CD8^+^ T-cell infiltration and other gene signatures identifying immune infiltration^15^ (all *P* < 0.001 and Extended Data Fig. 6b). Patients with cytolytic-low tumors had longer OS than those with cytolytic-high tumors in the Enco+Cetux arm (HR, 1.85 (95% CI, 1.203–2.847) for cytolytic-high versus cytolytic-low]); in contrast, Enco+Bini+Cetux-treated patients with cytolytic-high tumors had longer OS (HR, 0.56 (95% CI, 0.377–0.844) for cytolytic-high versus cytolytic-low); *P*_interaction_ = 0.0012) (Fig. 4c and Supplementary Table 5). Similar trends were observed for PFS, albeit slightly less pronounced (HR, 1.62 (95% CI, 1.107–2.365) for cytolytic-high versus cytolytic-low in the Enco+Cetux arm and HR, 0.69 (95% CI, 0.476–0.997) in the Enco+Bini+Cetux arm; *P*_interaction_ = 0.0088) (Extended Data Fig. 7a,b). Inferring immune cell type abundance using xCell^16^ affirmed an enrichment of lymphocytes (for example T cells, B cells, plasma cells and natural killer cells) in tumors with higher cytolytic scores (Extended Data Fig. 8).

BM1 tumors had higher cytolytic scores than BM2 tumors (*P* < 0.0001) (Supplementary Fig. 4, top panel). There was a range of cytolytic scores across the CMS subtypes, with potentially higher cytolytic scores in tumors characterized as CMS1 and CMS4 (CMS4 versus CMS1; *P* = 0.0861) (Supplementary Fig. 4, bottom panel).

### Acquired resistance alterations with Enco+Cetux±Bini

**Fig. 5:**
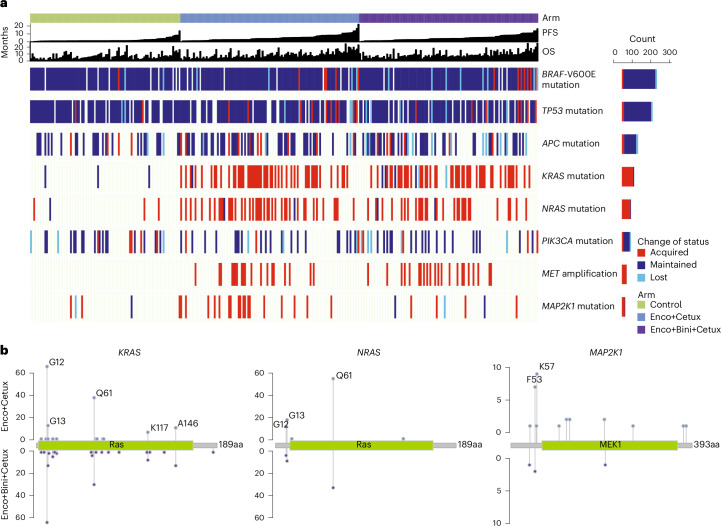
Top acquired resistance alterations in BEACON CRC. **a**, Status of selected gene alterations (analyzed by ctDNA genomic profiling) at EoT versus baseline and survival outcomes by treatment arm. **b**, Mutational landscape of *KRAS*, *NRAS* and *MAP2K1* variants in the Enco+Cetux and Enco+Bini+Cetux arms.

We analyzed paired cycle 1 day 1 (C1D1) and EoT samples using ctDNA genomic profiling to assess the landscape of acquired mutations by characterizing alterations as maintained, lost or acquired. Mutations in *BRAF*-V600E were predominantly maintained across all treatment arms (Fig. 5a).

The most frequently (that is, top) acquired putative resistance alterations were mutations in *KRAS*, *NRAS* and *MAP2K1* and amplification of *MET* (Fig. 5a). *KRAS*, *BRAF* and *IGF1R* amplifications were acquired at frequencies of 8.9%, 5.4% and 4.5%, respectively, in the Enco+Bini+Cetux arm and 4.5%, 1.8% and 0.9% in the Enco+Cetux arm. One patient in the control arm developed *BRAF* amplification (Supplementary Table 6).

Of the 318 patients with paired samples, acquired *MET* fusions were found in 13 (4.1%), *BRAF* fusions in 11 (3.5%) and *ALK* fusions in 7 (2.2%). Acquired fusions were also found in *RET*, *NTRK1* and *MERTK* at very low frequencies. Acquired *EGFR* mutations were observed at a low frequency in the Enco+Bini+Cetux and Enco+Cetux arms (eight and four patients, respectively) (Supplementary Table 6).

**Extended Data Fig. 9:**
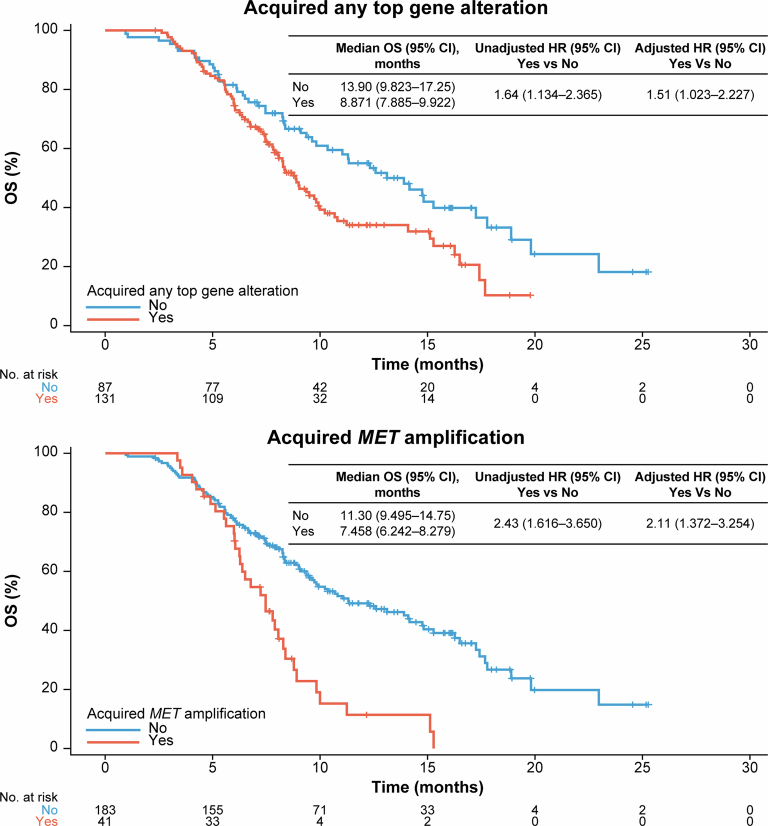
Kaplan-Meier plots of OS by presence of top acquired alterations (top panel) or MET amplification (bottom panel) following treatment with Enco+Cetux±Bini. Cox proportional hazard models with and without adjustment of baseline covariates (ECOG performance status, C-reactive protein, number of organs, tumor status, cetuximab source, and prior use of irinotecan at randomization) were used. Bini, binimetinib; Cetux, cetuximab; CI, confidence interval; Enco, encorafenib; HR hazard ratio; OS, overall survival.

Retrospective analyses in patients who did not have a mutation in *KRAS*, *NRAS* or *MAP2K1* or *MET* amplification at baseline showed that patients who did not acquire any of these alterations at EoT had longer survival than those who did (OS: HR, 1.64 (95% CI, 1.134–2.365); PFS: HR, 1.75 (95% CI, 1.258–2.435)) (Extended Data Fig. 9, top panel and Supplementary Fig. 5a,b (adjusted analyses)). Patients whose tumors acquired *MET* amplification at EoT had shorter survival (OS: HR, 2.43 (95% CI, 1.616–3.650); PFS: HR, 2.28 (95% CI, 1.560–3.321)) than those whose tumors did not (Extended Data Fig. 9 (bottom panel) and Supplementary Fig. 5a,b (adjusted analyses)).

Among patients who did not have baseline alterations in the top acquired genes, the frequency of any alterations in these genes at EoT was 58.3%, 61.8% and 6.8% in the Enco+Bini+Cetux, Enco+Cetux and control arms, respectively (Supplementary Table 7). Of the patients with paired ctDNA data, the frequency for two or more acquired alterations was 34.8%, 41.1% and 0%, respectively (Supplementary Fig. 6). *KRAS* mutations were acquired in 42.5% and 45.0% of Enco+Bini+Cetux- and Enco+Cetux-treated patients, respectively, and *NRAS* mutations in 25.5% and 37.3%; these mutations were found predominantly in hotspot regions of the respective genes (Fig. 5b and Supplementary Table 7). Acquired *MAP2K1* mutations were observed in 16.1% of patients treated with Enco+Cetux but in only 3.7% of patients treated with Enco+Bini+Cetux (*P* = 0.0028 for between-group difference) and were primarily RAF dependent, including F53L, K57E/N, C121S and E203K (Fig. 5b and Supplementary Tables 7 and 8). *MET* amplification was acquired in 22 of 112 (19.6%) and 19 of 112 (17.0%) Enco+Bini+Cetux- and Enco+Cetux-treated patients, respectively (Supplementary Table 7).

### Characterization of acquired resistance alterations

**Fig. 6:**
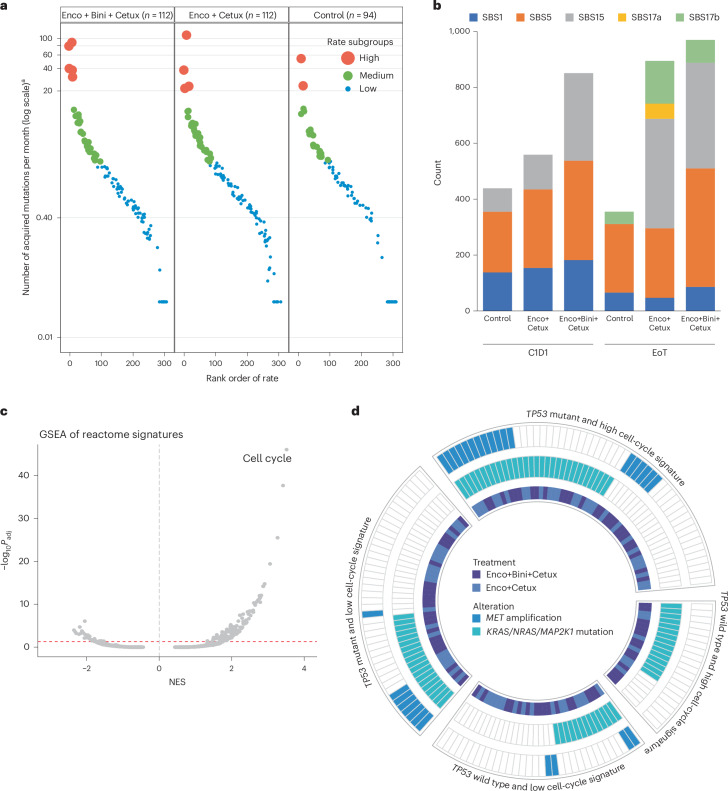
Characterization of acquired putative resistance alterations. **a**, Subgroups based on rate of acquiring resistance. Poisson mixture model was used to identify the low-, medium- and high-rate subgroups. **b**, Number of mutations grouped by SBS mutational signature at C1D1 and EoT. **c**, GSEA against hallmark reactome signatures based on the association between baseline gene expression and acquiring one or more top alteration at EoT. The dashed red line corresponds to an adjusted *P* value of 0.05 from GSEA. **d**, Association of top acquired alterations with baseline *TP53* mutation status and GSEA hallmark cell-cycle signature (split on median) (OR = 4.3; *P* = 0.006); Benjamini–Hochberg-adjusted *P* values (two-sided) are based on permutation tests (two-sided). ^a^The value 0 was coded as half of the smallest nonzero value, 0.03, for display.

**Extended Data Fig. 10:**
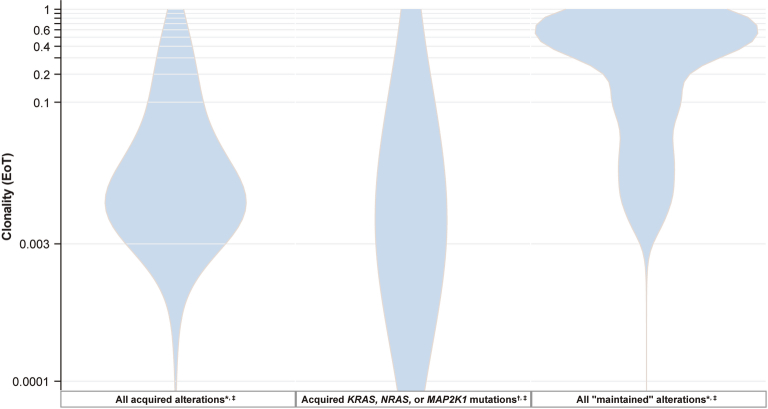
Clonality of acquired alterations determined by ctDNA genomic profiling analysis. Violin plots showing clonality (EoT VAF/EoT max VAF) of all acquired putative resistance alterations (left); acquired *KRAS*, *NRAS*, and *MAP2K1* mutations (middle); and all maintained putative resistance alterations (right). *P value for comparison between all acquired vs maintained putative resistance alterations was based on the Wilcoxon rank-sum test (two-sided). The resulting p-value from the analysis is < 0.0001. ^†^Only mutations were evaluated in the *KRAS*, *NRAS*, and *MAP2K1* group. ^‡^Silent mutations, clonal hematopoiesis of indeterminate potential, and alterations from genes that also had copy number alterations were excluded from the analysis.

To assess the clonality of acquired resistance alterations, the VAF of the alteration versus the maxVAF for any somatic alteration in the sample at EoT was determined. Acquired resistance alterations were predominately subclonal (≤ 0.3 EoT VAF/maxVAF) compared with alterations that were detected at baseline and maintained following treatment (*P* < 0.0001) (Extended Data Fig. 10). The rate of alterations acquired on treatment (number of acquired mutations per month) can be used to estimate subsequent rates of new alterations. Among patients in the Enco+Bini+Cetux and Enco+Cetux arms, the rate was approximately 1.7 and 1.5 times that in patients in the control arm, respectively (Fig. 6a). The rate at which alterations were acquired was found to represent a continuum across patients, with a subset showing >20 acquired mutations per month (Fig. 6a).

To understand the nature of these acquired mutations, we evaluated the single-base substitution (SBS) mutational signatures (Catalogue of Somatic Mutations in Cancer)^17^ from ctDNA genomic profiling by pooling genomic data by treatment arm and timepoint (C1D1 and EoT). We observed that SBS1 and SBS5—potentially caused by spontaneous or clock-like mutation processes—and SBS15—associated with DNA mismatch repair^18^—were identified at both C1D1 and EoT across all treatment groups. However, SBS17, which is associated with reactive oxygen species (ROS)^19^, was observed only at EoT; SBS17b (mainly T>G mutations) was identified in all treatment arms and at higher frequencies in the Enco+Bini+Cetux and Enco+Cetux arms, while SBS17a (mainly T>C mutations) was observed in Enco+Cetux-treated patients (Fig. 6b).

To determine the baseline biological tumor characteristics associated with acquired resistance alterations following treatment with Enco+Cetux±Bini, we compared the genomic and transcriptomic profiles of baseline tumor tissues between patients who did and did not subsequently acquire a presumptive resistance alteration (that is, top acquired resistance alteration). GSEA analyses showed prominent enrichment of the hallmark cell-cycle signature in tumors of patients who acquired alterations compared with those who did not following treatment with Enco+Cetux±Bini, with *CCNB1* and *CDK1* in the leading edge and more highly expressed (Fig. 6c and Supplementary Figs. 7 and 8). A higher proportion of patients with the BM2 subtype acquired resistance than those with the BM1 subtype (*P* = 0.029) (Supplementary Fig. 9). Analysis of baseline tumor mutations showed that a higher proportion of patients with *TP53* mutation acquired *MET* amplification following treatment with Enco+Cetux±Bini than those with wild-type *TP53* (odds ratio = 4.3; *P* = 0.006) (Fig. 6d); this difference between the *TP53* mutant and wild-type subgroups was consistent when considering the acquisition of any putative resistance amplification (that is, *MET*, *KRAS*, *BRAF*, *IGF1R*; odds ratio = 4.2; *P* = 0.0006) (Supplementary Fig. 10).

## Discussion

The primary analysis of BEACON CRC showed that Enco+Cetux, with or without Bini, significantly improved OS compared with control (Cetux+irinotecan, or Cetux+folinic acid, fluorouracil and irinotecan (FOLFIRI)) in patients with BRAF-V600-mutant mCRC. Here, we report a prespecified, retrospective, exploratory biomarker analysis of this large, phase 3 dataset.

Overall, the clinical benefit observed with treatment with Enco+Bini+Cetux and Enco+Cetux compared with control was maintained across CMS and BM subtypes, regardless of specific gene mutations, MSI or TMB. CMS and BM subtypes have come to define molecular paradigms for CRC, and their analyses were included here to contextualize our findings. Nonetheless, results in this study were supported by unbiased differential expression and GSEA, which served to further highlight immunological features of the TME as potential determinants of treatment benefit. While most mCRCs have microsatellite stability (MSS) and hence are considered immune cold, those that have MSS and are *BRAF*-V600E mutant have been reported to show some degree of immune activation^2,20–22^. Immune signatures may be enriched in BM1 and CMS1/CMS4 CRC subtypes, which have been shown to have a stronger immune profile and greater inflammatory response compared with other CRC subtypes^5,23^.

In the present study, patients with tumors that had a stronger immune signature (for example, increased T cells or cytolytic score) showed a trend towards decreased benefit from treatment with Enco+Cetux and increased benefit from treatment with Enco+Bini+Cetux. The observed trend in treatment outcomes with Enco+Bini+Cetux compared with Enco+Cetux could be explained by immune or inflammatory signals within the tumor microenvironment. It was previously reported that BRAF inhibitors alone showed stimulatory effects on T cells, potentially through paradoxical activation of ERK^24^. In contrast, MEK inhibitors led to partial and transient inhibition of T cell proliferation and potentially induced regenerative stem cell-like memory CD8^+^ T cells^25,26^, which might confer protection against T cell exhaustion and support antitumor responses. Therefore, patients with *BRAF*-V600E-mutant CRC, who had higher levels of immune activity, might derive further benefit from the addition of a MEK inhibitor or immunotherapy in combination with BRAF/EGFR inhibition, possibly accounting for the aforementioned findings in the present study. However, these trends should be interpreted with caution due to the retrospective nature of the analyses and small sample sizes, warranting further evaluation in future prospective trials. Nevertheless, our findings also support ongoing trials, including SEAMARK (NCT05217446) in MSI-H CRC and other studies in MSS CRC (NCT04017650 and NCT05308446 (SWOG2107)), which are evaluating whether the combination of Enco+Cetux with checkpoint inhibitors will increase clinical efficacy^27–29^.

Although a potential association between clinical outcomes and immune gene signature was observed in the present study, this was not seen with specific gene mutations, including *RNF43*. However, a study previously reported that *RNF43* mutations predicted response to BRAF inhibitor in combination with an anti-EGFR monoclonal antibody in *BRAF*-V600E-mutant mCRC^30^. The observed disparity in the predictive value of *RNF43* mutations might be attributed to differences in patient population, sample size or comparator arm (chemotherapy ± antiangiogenic therapy). The predictive value of specific *RNF43* mutations in patients who receive anti-BRAF/EGFR therapy requires further investigation in adequately powered prospective studies.

We observed a highly specific trend of acquired MAPK pathway alterations (*KRAS* and *NRAS* mutations) and *MET* amplifications that were similarly represented in the Enco+Bini+Cetux and Enco+Cetux arms but nearly absent in the control group. Our findings align with reports from previous smaller studies^8–10,31^, suggesting that targeting downstream MAPK activity or MET blockade could potentially help delay and overcome acquired resistance to Enco+Cetux, with or without Bini^8–10^. Activation of other receptor tyrosine kinases as part of a compensatory mechanism can also lead to the feedback activation of MEK, and the addition of SHP2 inhibitor has shown the potential to overcome resistance to MEK inhibitors^32–34^. Acquired *MAP2K1* mutations observed following treatment in this study were also previously identified in patients with *BRAF*-V600E-mutant CRC treated with EGFR/BRAF inhibitors^8,10^. Most of the detected *MAP2K1* mutations were primarily RAF dependent and therefore sensitive to MEK inhibition. Notably, acquired *MAP2K1* mutations were around five times more common in Enco+Cetux-treated patients compared with Enco+Bini+Cetux-treated patients, which is consistent with the ability of Bini to inhibit common *MAP2K1*-activating mutations. Nevertheless, the frequency of RAF-dependent MEK resistance mutations was relatively low compared with that of alterations in *KRAS*, *NRAS* and *MET*, which were observed in both treatment arms. Acquired *KRAS*, *BRAF* and *IGF1R* amplifications were observed at slightly higher frequencies in the Enco+Bini+Cetux arm (8.9%, 5.4% and 4.5%, respectively) than in the Enco+Cetux arm (4.5%, 1.8% and 0.9%). Known *EGFR* resistance mutations in the EGFR extracellular domain, which may be associated with resistance to Cetux^35^, were rarely acquired. In the present study, patients who had acquired top putative resistance alterations (that is, in *RAS*, *MAP2K1* and *MET*) had shorter PFS and OS, with similar HRs for both survival variables. These data suggest that patients who developed these alterations might experience faster progression on treatment, leading to shorter survival. Nevertheless, this analysis was only based on EoT samples, thus limiting interpretation; we do not have longitudinal sampling during treatment before progression, which would allow for more appropriate statistical evaluation of the association between acquired resistance alterations and survival.

Adaptive mutability refers to an increased mutation rate enabling cancer cells to evade therapeutic pressure^36^. This was reported in CRC in ref. ^36^; EGFR/BRAF inhibition downregulated DNA repair pathways and increased expression of low-fidelity DNA polymerases to promote DNA damage, mutability and microsatellite instability^36^. In a recent study of EGFR inhibition in RAS/BRAF/EGFR wild-type mCRC, disease progression on treatment was observed to be driven by the acquisition of newly acquired alterations rather than the expansion of preexisting resistance subclones, supporting the concept of adaptive mutability^37^. Results from our analysis suggest the presence of adaptive mutability in a cohort of patients with *BRAF*-V600E CRC treated with BRAF inhibitor in combination with an anti-EGFR monoclonal antibody, with or without a MEK inhibitor. Our results demonstrate a high number of emergent alterations in samples from patients on treatment. In particular, the rate of acquiring new alterations was higher with Enco+Cetux±Bini treatment, with a greater number of patients who had >20 acquired alterations per month in these treatment arms than in the control arm.

Acquired resistance alterations with Enco+Cetux±Bini showed a potential association with SBS17a/b mutational signatures, characterized mainly by T>C and T>G mutations^17,38^. SBS17b mutational signature was reported to be the main contributor of specific *KRAS/NRAS* and *EGFR* mutations that were enriched at acquired resistance to Cetux^39^. BRAF inhibitor-resistant melanoma has been reported to show elevated ROS levels^40^, consistent with the association of SBS17 signature with mutability and ROS^19^. Increased ROS levels may, in turn, induce DNA damage and affect the DNA damage response, potentially contributing to adaptive mutability^36^. The present analysis reports a potential association between SBS17a/b mutational signatures and acquired resistance alterations observed via ctDNA analysis in a large registrational study. Although limited by the size of the sequencing panel used, the potential to detect the SBS17b mutational signature in ctDNA samples provides an advantage by allowing easier sample collection for evaluation of resistance mechanisms during progression and warrants further exploration. Of note, SBS17a/b signatures were not observed in baseline tumor samples; however, paired progression biopsies were not available for longitudinal comparison.

The enrichment of cell-cycle gene expression at baseline in patients who acquired these top resistance alterations in the present study might also support the theory of adaptive mutability^36^ in that reduced DNA repair coupled with high-cycling tumors allows a greater chance for DNA errors to accumulate and mutations to emerge from treatment pressure, contributing to the acquired resistance phenotype and observed worse survival outcomes. In contrast, low-cycling tumors may take a longer time to accumulate putative resistance alterations, delaying the development of acquired resistance. Of note, we observed a potential association between the BM2 subtype and acquired resistance, consistent with the reported dysregulation of the cell cycle in the BM2 CRC subtype^5^. Additionally, consistent with *TP53* mutations being associated with genomic instability^41^, we found that acquired *MET* amplification in the Enco+Cetux±Bini arms occurred in a higher proportion of patients with *TP53* mutations than in those without. The roles of upregulated cell-cycle gene expression and *TP53* mutations should be further evaluated in *BRAF*-V600E-mutant mCRC to determine whether they could be potential prognostic biomarkers in this patient population.

This study has several strengths. First, it is based on a large dataset for a *BRAF*-V600E-mutation-driven subset of CRC, from a phase 3 clinical trial with integrated tumor and ctDNA analyses via full WES/WTS sequencing and ctDNA genomic profiling. Second, the patients included in this analysis were representative of those randomly assigned to both targeted treatment arms and the control arm. Third, our detailed investigations provided insights into the potential effect of immune status within the tumor microenvironment on treatment outcomes in mCRC and explored characteristics of alterations acquired following treatment with BRAF inhibitor plus an anti-EGFR monoclonal antibody, with or without a MEK inhibitor, versus chemotherapy plus anti-EGFR therapy. A key limitation of this biomarker analysis is its retrospective nature; the biomarkers predictive of response and resistance to Enco+Cetux±Bini, in *BRAF*-V600E-mutant CRC need to be further evaluated prospectively. In addition, as acquired mutations were assessed using EoT samples, the exact timing of the occurrence of these mutations was unknown, which might limit interpretation of the association between acquired alterations and OS outcomes.

Results from this prespecified, exploratory biomarker analysis of BEACON CRC support the use of Enco+Cetux±Bini, for the treatment of patients with *BRAF*-V600E-mutant mCRC and provide important insights into the biology of response and resistance to MAPK-pathway-targeted therapy.

## Methods

### Ethics statement

The trial was approved by the institutional review board or independent ethics committee at each center (Supplementary Information) and was conducted in accordance with the requirements of the regulatory authorities of each country and with the provisions of the Declaration of Helsinki and the Good Clinical Practice guidelines of the International Council on Harmonisation. All patients provided written informed consent.

### Study design and participants

The BEACON CRC trial was a global, multicenter, randomized, open-label phase 3 trial. The study design has been reported previously^3^. The latest version of the protocol is available as Supplementary Information.

Patients with histologically or cytologically confirmed, mCRC with the *BRAF*-V600E mutation who had disease progression after one or two previous treatment regimens were enrolled. From May 2017 through January 2019, patients were assigned randomly 1:1:1 to receive encorafenib, binimetinib and cetuximab (Enco+Bini+Cetux); encorafenib and cetuximab (Enco+Cetux); or investigators’ choice of either cetuximab with irinotecan or FOLFIRI (control). Encorafenib was administered at a dose of 300 mg daily; binimetinib at 45 mg twice daily; cetuximab at 400 mg m^−2^ as an initial dose, then 250 mg m^−2^ weekly; irinotecan at 180 mg m^−2^ on days 1 and 15; folinic acid at 180 mg m^−2^ on days 1 and 15; and fluorouracil at 400 mg m^−2^ as an initial dose, then 1,200 mg m^−2^ per day for 2 days, initiated on days 1 and 15. Treatment was administered in 28-day cycles until disease progression, unacceptable toxic effects, withdrawal of consent, initiation of subsequent anticancer therapy or death.

This prespecified, retrospective, exploratory biomarker analysis of the BEACON CRC study was performed in enrolled patients who received one or more dose of study drug (safety set) and had tumor and/or plasma specimens available for WES, WTS or ctDNA analyses, hereafter referred to as the biomarker analysis set. The provision and analysis of tumor samples at EoT were not mandatory.

### Endpoints

This exploratory biomarker analysis evaluated genomic and transcriptomic correlates of survival outcomes. Patients were grouped by CRC subtype, individual gene alterations and gene signatures, on the basis of analyses of their blood and tissue samples at baseline and EoT, where available. PFS, assessed by blinded independent central review and investigators, was defined as the time from randomization to the earliest documented disease progression or death due to any cause. OS was defined as the time from randomization to death due to any cause. The association between acquired gene alterations in ctDNA and survival outcomes was analyzed to explore the potential mechanisms of resistance to encorafenib plus cetuximab, with or without binimetinib.

### WES and analysis

WES of formalin-fixed paraffin-embedded (FFPE) samples collected at patient screening was performed by Personalis Inc. using their Accuracy and Content Enhanced (ACE) Cancer Exome panel v.3 (sequenced on Illumina NovaSeq)^42^. Variant calls were generated using BWA, GATK, MuTect, Vardict and Picard and further processed using Personalis proxy normal and custom filters to remove germline variants. Matched normal controls were not available, but variants were filtered for presumptive somatic status based on Personalis’ internal control pipeline. MSI status and TMB were also determined by Personalis. Somatic mutations were reported if above a 5% VAF threshold. Nonsynonymous variants with at least five unique supporting reads in a sample were retained for further analyses. Putative mutations in *TET2* were among the most prevalent reported (in 37% of cases); however, as we were unable to verify that these were not germline and/or clonal hematopoiesis of indeterminant potential mutations, *TET2* was excluded from further WES analyses.

### WTS and analysis

RNA sequencing (RNA-seq) of FFPE tumor samples was conducted at Personalis Inc. DNA and RNA were dual isolated from FFPE samples with the AllPrep DNA/RNA FFPE Kit (Qiagen). Exome capture was performed using Agilent SureSelect Clinical Research Exome v.2 (Agilent Technologies) according to the manufacturer’s recommendations. Additional supplementation with Personalis ACE proprietary target probes was performed to enhance coverage in difficult-to-sequence regions within sets of biomedically and medically relevant genes. Details regarding the Personalis ACE assay design are further described in ref. ^43^. In brief, manufacturer protocols were modified such that the average library insert length was adjusted to around 250 bp, and the Stranded RNA-seq Kit (Kapa Biosystems) was used for RNA-seq. Sequencing was performed on NovaSeq 6000 sequencers (Illumina) with paired-end, 2 × 150-bp read lengths and using Illumina’s proprietary reversible terminator-based method. For RNA-seq, tumor specimens were sequenced to an average output of 100 million paired-end reads (total of 200 million reads) across the 74.8 Mb NeXT assay genomic footprint, and FASTQ files were generated. The assay provided normalized gene-level expression values as TPM, which were then log-transformed (as log_2_(TPM + 1)) for downstream analyses. We observed a potential modest batch effect in sequencing associated with sample type, where samples from patients with and without successful resections were separated in an exploratory principal component analysis (Supplementary Fig. 11). We thus confirmed key results on gene expression data batch-corrected for resection status using ComBat in the SVA R package v.3.50.0 (ref. ^44^).

#### Molecular subtyping

CMSs were assigned to each sample using the CMSclassifier package v.1.0.0 (ref. ^45^) and the single-sample predictor method after mapping gene symbols to Entrez IDs using AnnotationDbi (Bioconductor) v.1.62.2 (ref. ^46^). Not all patients received an unambiguous subtyping call; thus, the nearest subtype prediction was used to estimate a subtype for all cases. BM subtypes were assigned by summing the weighted *z*-score expression of each of 44 subtyping genes reported in ref. ^5^, using the gene weights provided therein. Samples with a score of >1.5 were categorized as BM1; those with a score of ≤1.5 were categorized as BM2.

#### Associations with outcome

Genes in the top quartile of variable expression across samples (by IQR) were included in analyses. Univariable expression (scaled as *z*-score) associations with OS were evaluated in each arm separately using Cox proportional hazards models. Genes were ranked according to model *z*-scores for GSEA tests using the fgsea package v.1.28.0 (Bioconductor) against hallmark genesets accessed via the MSigDB Rr package v.6.2.1 (refs. ^47,48^). To identify additional signals that might be predictive of therapeutic benefit in specific arms, Cox models were fitted for the interaction between gene and arm for analysis sets, including the control and Enco+Cetux arms and the Enco+Cetux and Enco+Bini+Cetux arms separately, with GSEA performed after ranking of genes based on interaction *z*-scores.

#### Signature estimation

Signatures of immune infiltration were estimated using several approaches. Briefly, the cytolytic score was estimated as the (geometric) mean of *PRF1* and *GZMA* expression^13^; the immunological constant of rejection as the (geometric) mean of the expression of genes in the 20-gene signature^49^; and the CD8 T cell signature from ref. ^50^ as the (geometric) mean of *CD8A* and *CD8B*. xCell v.1.1.0 and MCPcounter v.1.2.0 were run with default parameters^16,51^. LM22 signature scores were calculated via ssGSEA of LM22 signature genes^52^.

### Genomic ctDNA analysis

Plasma samples were collected for ctDNA analysis before first therapy exposure at C1D1 and at EoT and sequenced using the GuardantOMNI assay (Guardant Health, Inc.)—a targeted next-generation sequencing panel of around 500 genes—to evaluate somatic alterations, including single-nucleotide mutations; insertions and deletions; copy number alteration and gene fusion events. Variants were detected and reported based on molecule support, which corresponded to variants with ≥0.01% VAF.

All types of somatic alterations were called by Guardant Health using its proprietary bioinformatics pipeline^53^. Aneuploidy, silent mutations and mutations probably arising from clonal hematopoiesis of indeterminate potential were excluded from downstream analysis. For analyses of paired ctDNA data, the resulting alterations were further evaluated for each patient with both C1D1 and EoT samples and characterized at an alteration level as acquired (that is, found in EoT sample only), maintained (found in both C1D1 and EoT samples), or lost (found in C1D1 samples only) for all treatment arms. For gene-level data, if there was more than one alteration for a gene, maintained alteration are shown rather than acquired or lost alterations, and acquired alterations are shown rather than lost alterations. For ctDNA clonality analyses, clonality was a measurement of VAF/maxVAF at EoT on a sample level.

The association of baseline ctDNA *BRAF*-V600E status with baseline tumor size and baseline ctDNA maxVAF was examined. Baseline tumor size was defined as the sum of target lesions assessed before randomization; the latest measurement was used if a lesion was assessed several times.

#### Mutational signature analysis

For treatment group comparisons, we pooled the somatic protein-altering variants detected in ctDNA samples in a group-wise manner and then applied SigProfilerExtractor v.1.1 (ref. ^54^) to quantify the mutational signatures active in each group, based on a set of predefined SBS mutational signatures (Catalogue of Somatic Mutations in Cancer Mutational Signatures v.3.3, June 2022)^17^ obtained from the Wellcome Sanger Institute. Baseline alterations were defined as C1D1 VAF > 1%, and acquired alterations were defined as C1D1 VAF = 0 and EoT VAF > 0.

As with survival analyses, identification of gene expression correlates of mutation acquisition was performed using variance-filtered and *z*-scaled gene expression. GSEA analyses were based on genes ordered by *t*-statistics from univariable linear models.

### Statistical analysis

Calculation of the overall sample size required for the primary endpoints, statistical testing schemes and preplanned interim analysis was described previously^3^. All data reported here are from prespecified exploratory biomarker analyses performed using data (cutoff 15 August 2019 (ref. ^4^)) from patients who received at least one dose of study drug; had at least one post-treatment assessment, which may include death; and had at least one valid biomarker measurement at the timepoint(s) of interest (baseline versus EoT). For analyses involving treatment, patients were analyzed according to the actual treatment received.

For biomarkers without a predefined cut point, subgroup analysis was performed based on a median split to maintain balance between subgroup size, and testing of treatment-by-biomarker interactions was performed using continuous measurements. Identification of optimal biomarker thresholds was not the objective of the analyses. Exploratory biomarker analyses (for example, subgroup analysis) are subject to both type I error (false-positive) due to multiple comparisons and type II error (false-negative) due to lack of statistical power (for example, test of interaction in subgroup analysis)^55^. The objective of the biomarker analysis is hypothesis generating; therefore, to reduce potential type II error^56,57^, the results of statistical analyses are presented without correction for multiple comparisons. To assess the effect of potential confounders, results based on models with adjustment of key baseline covariates (Eastern Cooperative Oncology Group performance status, C-reactive protein, number of organs, tumor status, cetuximab source and previous use of irinotecan at randomization) are provided.

Kaplan–Meier estimates of OS and PFS were presented by treatment arm and biomarker subgroup. The median PFS and OS (in months) were summarized, along with 95% CIs. Cox proportional hazards models were used to calculate HRs and 95% CIs in each treatment arm or biomarker subgroup, either with or without adjustment of baseline measurements of known prognostic factors. The interaction term was evaluated using the Cox proportional hazards model, including treatment, biomarker and the interaction term, with the *P* value determined by the two-sided Wald test. The Fisher exact test was used to compare proportions between independent samples with small sample size. The McNemar’s test was used to compare proportions between paired samples (for example baseline versus EoT). Depending on the data distribution, either the Wilcoxon rank-sum test or one-way analysis of variance was used to compare biomarker levels between independent samples. The Poisson mixture model was used to identify patient subgroups that acquired mutations at different rates. For visual display of patient subgroups, a rate was calculated using the number of acquired mutations and time on treatment for each patient, which allowed comparison of the number of mutations emerging that was adjusted for time on treatment. The negative binomial model was used to compare the rates of alteration acquisition across treatment arms. For all analyses involving rate of acquired mutations, a constant rate of acquisition was assumed. Statistical analyses were done using SAS v.9.4 or R v.4.0 and above.

### Reporting summary

Further information on research design is available in the Nature Portfolio Reporting Summary linked to this article.

## Online content

Any methods, additional references, Nature Portfolio reporting summaries, source data, extended data, supplementary information, acknowledgements, peer review information; details of author contributions and competing interests; and statements of data and code availability are available at 10.1038/s41591-024-03235-9.

## Supplementary information

Supplementary Information List of IRBs and IECs, Protocol, and Supplementary Tables 1–8 and Figs. 1–11.

Reporting Summary

## Data Availability

The analyses in this paper were based on a data cutoff of 15 August 2019. Upon request, and subject to review, Pfizer will provide the data that support the findings of this study. Subject to certain criteria, conditions and exceptions, Pfizer may also provide access to the related individual deidentified participant data. Pfizer will also consider requests for the protocol, data dictionary and statistical analysis plan. See https://www.pfizer.com/science/clinical-trials/trial-data-and-results for more information. Data may be requested from Pfizer trials 24 months after study completion. The BEACON CRC study was completed in November 2022. The deidentified participant data will be made available to researchers whose proposals meet the research criteria and other conditions, and for which an exception does not apply, via a secure portal. To gain access, data requestors must enter into a data access agreement with Pfizer.
