# Supplementary material for: Molecular profiling of BRAF-V600E-mutant metastatic colorectal cancer in the phase 3 BEACON CRC trial
Source: Nat Med. 2024 Sep 23;30(11):3261–71. doi: 10.1038/s41591-024-03235-9 (PMC11564101; doi:10.1038/s41591-024-03235-9)
Supplement: Supplementary file 1 — List of IRBs and IECs, Protocol, and Supplementary Tables 1–8 and Figs. 1–11. [file 41591_2024_3235_MOESM1_ESM.pdf]

nature medicine

Supplementary information https://doi.org/10.1038/s41591-024-03235-9

Molecular profiling of BRAF-V600E-mutant metastatic colorectal cancer in the phase 3 BEACON CRC trial

In the format provided by the authors and unedited

LIST OF INDEPENDENT ETHICS COMMITTEE (IEC) OR INSTITUTIONAL REVIEW BOARD (IRB)

ARGENTINA

Study Site Number	Independent Ethics Committee or Institutional Review Board Address(es)
3003	Comite Independiente De Etica Para Ensayos En Farmacologia Clinica<br>J.E. Uriburu 774, 1st floor<br>Caba, BUENOS AIRES C1027AAp<br>ARGENTINA
3004	Comite de Etica en Investigacion Fundacion OncoSalud<br>Siria 16, Edificio Ex-INAN - Unidade II - Ministerio da Saude<br>Pergamino, BUENOS AIRES 2700<br>ARGENTINA
3005	Comite Institutional de Etica de la Investigation en Salud de la Clinica Universtiaria Reina Fabiola (CIEIS)<br>Jacinto Rios 554, Floor 8, Office 3<br>Cordoba, CORDOBA X5004FHP<br>ARGENTINA
3006	Comite Independiente De Etica Para Ensayos En Farmacologia Clinica<br>J. E. Uriburu 774. 1st Floor<br>CABA, BUENOS AIRES C1027AAP<br>ARGENTINA
3010	Comite de Etica Independiente Patagonico<br>Urquiza 646<br>Santa Rosa, LA PAMPA L6300EAN<br>ARGENTINA

AUSTRALIA

Study Site Number	Independent Ethics Committee or Institutional Review Board Address(es)
3101	Monash Health Human Research Ethics Committee<br>Monash Health, Monash Medical Centre, Level 2, I Block, 246 Clayton Road<br>Clayton, VIC 3168<br>AUSTRALIA
3102	Monash Health Human Research Ethics Committee<br>246 Clayton Road,Monash Health, Monash Medical Centre, level 2, I Block<br>Clayton, VIC 3168<br>AUSTRALIA
3103	Monash Health HREC<br>246 Clayton Road,Level 2, I Block, Monash Medical Centre<br>Melbourne, CLAYTON/VIC 3168<br>AUSTRALIA
3104	Monash Health Human Research Ethics Committee<br>Monash Health, Monash Medical Centre, Level 2, I Block, 246 Clayton Road<br>Clayton, VIC 3168<br>AUSTRALIA
3105	Monash Health HREC<br>246 Clayton Road,Level 2, I Block, Monash Medical Centre<br>Clayton, VICTORIA 3168<br>AUSTRALIA
3106	Monash Health Human Research Ethics Committee<br>Monash Health, Monash Medical Centre, Level 2, I Block, 246 Clayton Road<br>Clayton, VICTORIA (VIC) 3168<br>AUSTRALIA
3107	Monash Health HREC<br>246 Clayton Road,Level 2, I Block, Monash Medical Centre<br>Clayton, VICTORIA 3168<br>AUSTRALIA

AUSTRIA

Study Site Number	Independent Ethics Committee or Institutional Review Board Address(es)
3201	Ethik-Kommission der Medizinischen Universitaet Wien und des Allgemeinen Krankenhauses der Stadt<br>Wien - AKH<br>Borschkegasse 8b/E 06<br>Wien, WIEN 1090<br>AUSTRIA
3202	Ethik-Kommission der Medizinischen Universitaet Wien und des Allgemeinen Krankenhauses der Stadt<br>Wien - AKH<br>Borschkegasse 8b/E 06<br>Wien, WIEN 1090<br>AUSTRIA

BELGIUM

Study Site Number	Independent Ethics Committee or Institutional Review Board Address(es)
3301	Commissie voor Medische Ethiek van de Universitaire Ziekenhuizen Katholieke Universiteit Leuven<br>Herestraat 49<br>Leuven, 3000<br>BELGIUM
3302	Commissie voor Medische Ethiek van de Universitaire Ziekenhuizen Katholieke Universiteit Leuven<br>Herestraat 49<br>Leuven, 3000<br>BELGIUM
3303	Commissie voor Medische Ethiek van de Universitaire Ziekenhuizen Katholieke Universiteit Leuven<br>Herestraat 49<br>Leuven, 3000<br>BELGIUM
3304	Commissie voor Medische Ethiek van de Universitaire Ziekenhuizen Katholieke Universiteit Leuven<br>Herestraat 49<br>Leuven, 3000<br>BELGIUM
3305	Commissie voor Medische Ethiek van de Universitaire Ziekenhuizen Katholieke Universiteit Leuven<br>Herestraat 49<br>Leuven, 3000<br>BELGIUM
3306	Commissie voor Medische Ethiek van de Universitaire Ziekenhuizen Katholieke Universiteit Leuven<br>Herestraat 49<br>Leuven, 3000<br>BELGIUM

Study Site Number	Independent Ethics Committee or Institutional Review Board Address(es)
3307	Commissie voor Medische Ethiek van de Universitaire Ziekenhuizen Katholieke Universiteit Leuven<br>Herestraat 49<br>Leuven, 3000<br>BELGIUM
3308	Commissie voor Medische Ethiek van de Universitaire Ziekenhuizen Katholieke Universiteit Leuven<br>Herestraat 49<br>Leuven, 3000<br>BELGIUM
3309	Commissie voor Medische Ethiek van de Universitaire Ziekenhuizen Katholieke Univeristeit Leuven<br>Herestraat 49<br>Leuven, 3000<br>BELGIUM
3310	Commissie voor Medische Ethiek van de Universitaire Ziekenhuizen Katholieke Universiteit Leuven<br>Herestraat 49<br>Leuven, 3000<br>BELGIUM
3311	Commissie voor Medische Ethiek van de Universitaire Ziekenhuizen Katholieke Universiteit Leuven<br>Herestraat 49<br>Leuven, 3000<br>BELGIUM

BRAZIL

Study Site Number	Independent Ethics Committee or Institutional Review Board Address(es)
3401	Comissao Nacional de Etica em Pesquisa - CONEP<br>SEPN 510 NORTE, Bloco A - 3° Andar - Asa Norte, Edificio Ex-INAN - Unidade II - Ministerio da Saude<br>Brasilia, DF 70750-521<br>BRAZIL<br><br>Comite de Etica em Pesquisa da Irmandade da Santa Casa de Misericordia de Porto Alegre<br>Rua Professor Annes Dias, 295, 6° Andar - Hospital Dom Vicente Scherer<br>Porto Alegre, SP 90020-090<br>BRAZIL
3402	Comissao Nacional de Etica em Pesquisa - CONEP (Inactive IRB/IEC)<br>SEPN 510 NORTE, Bloco A - 3° Andar - Asa Norte, Edificio Ex-INAN - Unidade II - Ministerio da Saude<br>Brasilia, DF 70750-521<br>BRAZIL<br><br>Comissao Nacional de Etica em Pesquisa - CONEP<br>SRTV 701, Via W 5 Norte, lote D - Asa Norte, Edificio PO 700, 3° andar<br>Brasilia, DF 70719-040<br>BRAZIL<br><br>Comite de Etica em Pesquisa da Faculdade de Ciencias Medicas e da Saude PUCSP<br>Rua Joubert Wey, 290<br>Sorocaba, SP 18030-070<br>BRAZIL

Study Site Number	Independent Ethics Committee or Institutional Review Board Address(es)
3403	Comissao Nacional de Etica em Pesquisa - CONEP (Inactive IRB/IEC)<br>SEPN 510 NORTE, Bloco A - 3° Andar - Asa Norte, Edificio Ex-INAN - Unidade II - Ministerio da Saude<br>Brasilia, DF 70750-521<br>BRAZIL<br><br>Comissao Nacional de Etica em Pesquisa - CONEP<br>SRTV 701, Via W 5 Norte, lote D - Asa Norte, Edificio PO 700, 3° andar<br>Brasilia, DF 70719-040<br>BRAZIL<br><br>Comite de Etica em Pesquisa da Santa Casa de Misericordia de Pelotas<br>Praca Piratinino de Almeida 53<br>Pelotas, RS 96015-290<br>BRAZIL
3404	Comissao Nacional de Etica em Pesquisa - CONEP (Inactive IRB/IEC)<br>SEPN 510 NORTE, Bloco A - 3° Andar - Asa Norte, Edificio Ex-INAN - Unidade II - Ministerio da Saude<br>Brasilia, DF 70750-521<br>BRAZIL<br><br>Comissao Nacional de Etica em Pesquisa - CONEP<br>SRTV 701, Via W 5 Norte, lote D - Asa Norte, Edificio PO 700, 3° andar<br>Brasilia, DF 70719-040<br>BRAZIL<br><br>Comite de Etica em Pesquisa em Seres Humanos da Faculdade de Medicina de Sao Jose do Rio Preto<br>FAMERP<br>Av. Brigadeiro Faria Lima, 5416, Vila Sao Pedro<br>Sao Jose do Rio Preto, SP 15090-000<br>BRAZIL

Study Site Number	Independent Ethics Committee or Institutional Review Board Address(es)
3405	Comissao Nacional de Etica em Pesquisa - CONEP (Inactive IRB/IEC)<br>SEPN 510 NORTE, Bloco A - 3° Andar - Asa Norte, Edificio Ex-INAN - Unidade II - Ministerio da Saude<br>Brasilia, DF 70750-521<br>BRAZIL<br><br>Comissao Nacional de Etica em Pesquisa - CONEP<br>SRTV 701, Via W 5 Norte, lote D - Asa Norte, Edificio PO 700, 3° andar<br>Brasilia, DF 70719-040<br>BRAZIL<br><br>Comite de Etica em Pesquisa da Universidade de Passo Fundo<br>Universidade de Passo Fundo - Km 292-BR 285, Bairro Sao Jose<br>Passo Fundo, RS 99052-900<br>BRAZIL
3406	Comissao Nacional de Etica em Pesquisa - CONEP (Inactive IRB/IEC)<br>SEPN 510 NORTE, Bloco A - 3° Andar - Asa Norte, Edificio Ex-INAN - Unidade II - Ministerio da Saude<br>Brasilia, DF 70750-521<br>BRAZIL<br><br>Comissao Nacional de Etica em Pesquisa - CONEP<br>SRTV 701, Via W 5 Norte, lote D - Asa Norte, Edificio PO 700, 3° andar<br>Brasilia, DF 70719-040<br>BRAZIL<br><br>Comite de Etica em Pesquisa em Seres Humanos do Centro Universitario UNIVATES<br>Avenida Avelino Tallini, 171, Bairro Universitario<br>Lajeado, RS 95900-000<br>BRAZIL

Study Site Number	Independent Ethics Committee or Institutional Review Board Address(es)
3407	Comissao Nacional de Etica em Pesquisa - CONEP<br>SEPN 510 NORTE, Bloco A - 3° Andar - Asa Norte, Edificio Ex-INAN - Unidade II - Ministerio da Saude<br>Brasilia, DF 70750-521<br>BRAZIL
	Comitê de Ética em Pesquisa do Centro Integrado de Atenção à Saúde (CIAS) - Unimed Vitória<br>Rua Marins Alvarino, 365, Bairro Itararé<br>Vitória, ES 29047-660<br>BRAZIL
3408	Comissao Nacional de Etica em Pesquisa - CONEP (Inactive IRB/IEC)<br>SEPN 510 NORTE, Bloco A - 3° Andar - Asa Norte, Edificio Ex-INAN - Unidade II - Ministerio da Saude<br>Brasilia, DF 70750-521<br>BRAZIL
	Comissao Nacional de Etica em Pesquisa - CONEP<br>SRTV 701, Via W 5 Norte, lote D - Asa Norte, Edificio PO 700, 3° andar<br>Brasilia, DF 70719-040<br>BRAZIL
	Comite de Etica em Pesquisa do Hospital Mae De Deus (Inactive IRB/IEC)<br>Rua da Costa, 150<br>Porto Alegre, SP 90110-270<br>BRAZIL
	Comite de Etica em Pesquisa do Hospital Mae De Deus<br>Rua Grao Para, 160, 2° floor - Menino Deus<br>Porto Alegre, RS 90850-170<br>BRAZIL

Study Site Number	Independent Ethics Committee or Institutional Review Board Address(es)
3409	Comissao Nacional de Etica em Pesquisa - CONEP (Inactive IRB/IEC)<br>SEPN 510 NORTE, Bloco A-3 Andar -Asa Norte, Edificio Ex -INAN Unidade II -Ministerio da Saude<br>Brasilia, DF 70750-521<br>BRAZIL<br><br>Comissao Nacional de Etica em Pesquisa - CONEP<br>SRTV 701, Via W 5 Norte, lote D - Asa Norte, Edificio PO 700, 3° andar<br>Brasilia, DF 70719-040<br>BRAZIL<br><br>Comitê de Ética em Pesquisa da Faculdade de Medicina do ABC (Inactive IRB/IEC)<br>Avenida Principe de Gales, n°821, Predio CEPES - 1° Andar<br>Santo Andre, SP 09060-650<br>BRAZIL<br><br>Comitê de Ética em Pesquisa da Faculdade de Medicina do ABC<br>Av. Lauro Gomes, 2000, Predio Cepes - 1º Andar<br>Santo Andre, SP 09060-870<br>BRAZIL
3410	Comissao Nacional de Etica em Pesquisa - CONEP (Inactive IRB/IEC)<br>SEPN 510 NORTE, Bloco A-3 Andar -Asa Norte, Edificio Ex -INAN Unidade II -Ministerio da Saude<br>Brasilia, DF 70750-521<br>BRAZIL<br><br>Comissao Nacional de Etica em Pesquisa - CONEP<br>SRTV 701, Via W 5 Norte, lote D - Asa Norte, Edificio po 700, 3 andar<br>Brasilia, DF 70719-040<br>BRAZIL<br><br>Comitê de Ética em Pesquisa da Faculdade de Medicina do ABC<br>Av. Lauro Gomes, 2000, Prédio Cepes, Vila Sacadura Cabral<br>Santo André, SÃO PAULO 09060-870<br>BRAZIL

Study Site Number	Independent Ethics Committee or Institutional Review Board Address(es)
3411	Comissao Nacional de Etica em Pesquisa - CONEP (Inactive IRB/IEC)<br>SEPN 510 NORTE, Bloco A - 3° Andar - Asa Norte, Edificio Ex-INAN - Unidade II - Ministerio da Saude<br>Brasilia, DF 70750-521<br>BRAZIL<br><br>Comissao Nacional de Etica em Pesquisa - CONEP<br>SRTV 701, Via W 5 Norte, lote D - Edificio PO 700, 3° Andar, Edificio Ex-INAN - Unidade II - Ministerio da Saude<br>Brasilia, DF 70719-040<br>BRAZIL<br><br>Comite de Etica em Pesquisa da Fundacao Pio XII - Hospital do Cancer de Barretos<br>Rua Antenor Duarte Villela, 1331<br>Barretos, SAO PAULO 14784-400<br>BRAZIL
3412	Comissao Nacional de Etica em Pesquisa - CONEP (Inactive IRB/IEC)<br>SEPN 510 NORTE, Bloco A - 3° Andar - Asa Norte, Edificio Ex-INAN - Unidade II - Ministerio da Saude<br>Brasilia, DF 70750-521<br>BRAZIL<br><br>Comissao Nacional de Etica em Pesquisa - CONEP<br>SRTV 701, Via W 5 Norte, lote D - Asa Norte, Edificio PO 700, 3° andar<br>Brasilia, DF 70719-040<br>BRAZIL<br><br>Comite de Etica em Pesquisa da Universidade de Caxias do Sul (Inactive IRB/IEC)<br>Rua Francisco Getulio Vargas, 1130, Bloco M - Sala 106 - Bairro Petropolis<br>Caxias do Sul, RS 95070-560<br>BRAZIL

Study Site Number	Independent Ethics Committee or Institutional Review Board Address(es)
3413	Comissao Nacional de Etica em Pesquisa - CONEP (Inactive IRB/IEC)<br>SEPN 510 NORTE, Bloco A-3 Andar -Asa Norte, Edificio Ex -INAN Unidade II -Ministerio da Saude<br>Brasilia, DF 70750-521<br>BRAZIL
	Comissao Nacional de Etica em Pesquisa - CONEP<br>SRTV 701, Via W 5 Norte, lote D - Asa Norte, Edificio po 700, 3 andar<br>Brasilia, DF 70719-040<br>BRAZIL
	Comitê de Ética e Pesquisa do Hospital de Clínicas de Porto Alegre - CEP/HCPA<br>Rua Ramiro Barcelos, 2350, 2°Andar - Sala 2227<br>Porto Alegre, RS 90035-903<br>BRAZIL
3414	Comissao Nacional de Etica em Pesquisa - CONEP<br>SRTV 701, Via W 5 Norte, lote D - Edificio PO 700, 3 andar, Edificio Ex-INAN - Unidade II - Ministerio da Saude<br>Brasilia, DF 70719-040<br>BRAZIL
	Comitê de Ética em Pesquisa da Maternidade Climério de Oliveira - UFBA<br>Rua Do Limoeiro, 137, Nazaré<br>Salvador, BAHIA 40055-150<br>BRAZIL

Study Site Number	Independent Ethics Committee or Institutional Review Board Address(es)
3415	Comissao Nacional de Etica em Pesquisa - CONEP (Inactive IRB/IEC)<br>SEPN 510 NORTE, Bloco A - 3° Andar - Asa Norte, Edificio Ex-INAN - Unidade II - Ministerio da Saude<br>Brasilia, DF 70750-521<br>BRAZIL<br><br>Comissao Nacional de Etica em Pesquisa - CONEP<br>SRTV 701, Via W 5 Norte, lote D - Asa Norte, Edificio PO 700, 3° andar<br>Brasilia, DF 70719-040<br>BRAZIL<br><br>Comitê de Ética em Pesquisa do Hospital Lifecenter<br>Avenida do Contorno, 4747<br>Belo Horizonte, MINAS GERAIS 30110-921<br>BRAZIL

CANADA

Study Site Number	Independent Ethics Committee or Institutional Review Board Address(es)
3501	UBC BC Cancer Research Ethics Board<br>Fairmont Medical Building 1315-750, West Broadway<br>Vancouver, BRITISH COLUMBIA V5Z 1J3<br>CANADA
3502	Ontario Cancer Research Ethics Board<br>661 University Avenue,Suite 510<br>Toronto, ONTARIO M5G 0A3<br>CANADA
3503	Ontario Cancer Research Ethics Board<br>661 University Avenue,Suite 510<br>Toronto, ONTARIO M5G 0A3<br>CANADA
3505	Ontario Cancer Research Ethics Board<br>MaRS Centre, 661 University Avenue, Suite 510<br>Toronto, ONTARIO M5G 0A3<br>CANADA
3506	Research Ethics Committee - Centre intégré universitaire de santé et de services sociaux de l'Ouest-de-l'Île-de-Montréal - CIUSS-ODIM-Centre hospitalier de St. Mary<br>3830 Lacombe Avenue, Pavillon Hayes, bureau 4710<br>Montreal, QUEBEC H3T 1M5<br>CANADA

CHILE

Study Site Number	Independent Ethics Committee or Institutional Review Board Address(es)
5602	Comite de Etica Cientifico del Servicio de Salud Metropolitano Oriente<br>Av. Salvador 364, Providencia<br>Santiago, METROPOLITANA 7500922<br>CHILE
5603	Comite Etico Cientifico Hospital Dr. Gustavo Fricke Servicio de Salud Vina del Mar - Quillota<br>Calle Limache 1307<br>Vina del Mar, VALPARAISO 2520563<br>CHILE

CZECH REPUBLIC

Study Site Number	Independent Ethics Committee or Institutional Review Board Address(es)
3602	Eticka komise Fakultni nemocnice Olomouc<br>I.P.Pavlova 185/6<br>Olomouc, 779 00<br>CZECH REPUBLIC
3603	Eticka komise Fakultni nemocnice Olomouc<br>I. P. Pavlova 185/6<br>Olomouc, 779 00<br>CZECH REPUBLIC
	Eticka komise Masarykova onkologickeho ustavu<br>Zluty kopec 7<br>Brno, 656 53<br>CZECH REPUBLIC
3605	Eticka Komise FN Hradec Kralove<br>Sokolska 581<br>Hradec Kralove, 500 05<br>CZECH REPUBLIC
	Eticka komise Fakultni nemocnice Olomouc<br>I. P. Pavlova 185/6<br>Olomouc, 779 00<br>CZECH REPUBLIC

DENMARK

Study Site Number	Independent Ethics Committee or Institutional Review Board Address(es)
3701	De Videnskabsetiske Komiteer for Region Syddanmark<br>Regionshuset, Damhaven 12<br>Vejle, DK-7100<br>DENMARK

FRANCE

Study Site Number	Independent Ethics Committee or Institutional Review Board Address(es)
3804	CPP SUD-OUEST et OUTRE-MER II<br>ARS Midi Pyrenees, 10 chemin du raisin - BUREAU 1028<br>Toulouse Cedex 9, 31050<br>FRANCE
3805	CPP SUD-OUEST et OUTRE-MER II<br>ARS Midi Pyrenees, 10 chemin du raisin - BUREAU 1028<br>Toulouse Cedex 9, 31050<br>FRANCE
3806	CPP SUD-OUEST et OUTRE-MER II<br>Chemin du raisin 10 - BUREAU 1028,ARS Midi Pyrenees<br>Toulouse Cedex 9, 31050<br>FRANCE
3807	CPP SUD-OUEST et OUTRE-MER II<br>Chemin du raisin 10 - BUREAU 1028,ARS Midi Pyrenees<br>Toulouse Cedex 9, 31050<br>FRANCE
3808	CPP SUD-OUEST et OUTRE-MER II<br>Chemin du raisin 10 - BUREAU 1028,ARS Midi Pyrenees<br>Toulouse Cedex 9, 31050<br>FRANCE
3811	CPP SUD-OUEST et OUTRE-MER II<br>Chemin du raisin 10 - BUREAU 1028,ARS Midi Pyrenees<br>Toulouse Cedex 9, 31050<br>FRANCE
3812	CPP SUD-OUEST et OUTRE-MER II<br>ARS Midi Pyrenees, 10 chemin du raisin - BUREAU 1028<br>Toulouse Cedex 9, 31050<br>FRANCE

Study Site Number	Independent Ethics Committee or Institutional Review Board Address(es)
3814	CPP SUD-OUEST et OUTRE-MER II<br>Chemin du raisin 10 - BUREAU 1028,ARS Midi Pyrenees<br>Toulouse Cedex 9, 31050<br>FRANCE
3815	CPP SUD-OUEST et OUTRE-MER II<br>Chemin du raisin 10 - BUREAU 1028,ARS Midi Pyrenees<br>Toulouse Cedex 9, 31050<br>FRANCE

GERMANY

Study Site Number	Independent Ethics Committee or Institutional Review Board Address(es)
3902	Ethikkommission der Medizinischen Hochschule Hannover<br>Carl-Neuberg-Str. 1<br>Hannover, NIEDERSACHSEN 30625<br>GERMANY
3903	Ethikkommission der Medizinischen Hochschule Hannover<br>Carl-Neuberg-Str. 1<br>Hannover, NIEDERSACHSEN 30625<br>GERMANY
3904	Ethikkommission der Medizinischen Hochschule Hannover<br>Carl-Neuberg-Str. 1<br>Hannover, NIEDERSACHSEN 30625<br>GERMANY
3906	Ethikkommission der Medizinischen Hochschule Hannover<br>Carl-Neuberg-Str. 1<br>Hannover, NIEDERSACHSEN 30625<br>GERMANY
3908	Ethikkommission der Medizinischen Hochschule Hannover<br>Carl-Neuberg-Str. 1<br>Hannover, NIEDERSACHSEN 30625<br>GERMANY
3909	Ethikkommission der Medizinischen Hochschule Hannover<br>Carl-Neuberg-Str. 1<br>Hannover, NIEDERSACHSEN 30625<br>GERMANY
3911	Ethikkommission der Medizinischen Hochschule Hannover<br>Carl-Neuberg-Str. 1<br>Hannover, NIEDERSACHSEN 30625<br>GERMANY

Study Site Number	Independent Ethics Committee or Institutional Review Board Address(es)
3912	Ethikkommission der Medizinischen Hochschule Hannover<br>Carl-Neuberg-Str. 1<br>Hannover, NIEDERSACHSEN 30625<br>GERMANY
3913	Ethikkommission der Medizinischen Hochschule Hannover<br>Carl-Neuberg-Str. 1<br>Hannover, NIEDERSACHSEN 30625<br>GERMANY
3914	Ethikkommission der Medizinischen Hochschule Hannover<br>Carl-Neuberg-Str. 1<br>Hannover, NIEDERSACHSEN 30625<br>GERMANY
3915	Ethikkommission der Medizinischen Hochschule Hannover<br>Carl-Neuberg-Str. 1<br>Hannover, NIEDERSACHSEN 30625<br>GERMANY

HUNGARY

Study Site Number	Independent Ethics Committee or Institutional Review Board Address(es)
4001	Egészségügyi Tudományos Tanács Klinikai Farmakológiai Etikai Bizottsága<br>Arany J. u. 6-8.<br>Budapest, 1051<br>HUNGARY
4002	Egészségügyi Tudományos Tanács Klinikai Farmakológiai Etikai Bizottsága<br>Arany J. u. 6-8.<br>Budapest, 1051<br>HUNGARY
4003	Egészségügyi Tudományos Tanács Klinikai Farmakológiai Etikai Bizottsága<br>Arany J. u. 6-8.<br>Budapest, 1051<br>HUNGARY
4004	Egeszsegugyi Tudomanyos Tanacs Klinikai Farmakologiai Etikai Bizottsaga<br>Arany J. u. 6-8<br>Budapest, 1051<br>HUNGARY
4007	Egészségügyi Tudományos Tanács Klinikai Farmakológiai Etikai Bizottsága<br>Arany J. u. 6-8.<br>Budapest, 1051<br>HUNGARY
4009	Egészségügyi Tudományos Tanács Klinikai Farmakológiai Etikai Bizottsága<br>Arany J. u. 6-8.<br>Budapest, 1051<br>HUNGARY
4011	Egészségügyi Tudományos Tanács Klinikai Farmakológiai Etikai Bizottsága<br>Arany J. u. 6-8.<br>Budapest, 1051<br>HUNGARY

ISRAEL

Study Site Number	Independent Ethics Committee or Institutional Review Board Address(es)
4201	Helsinki Committee of the Barzilai Medical Center<br>Barzilai Medical Center<br>2 Hahistadrout St.<br>Ashkelon, 7830604<br>ISRAEL
4203	Helsinki Committee of Meir Medical Center<br>59 Tshernichovsky St.<br>Kfar Saba, 4428164<br>ISRAEL
4204	Helsinki Committee of Tel Aviv Sourasky Medical Center<br>Tel Aviv Sourasky Medical Center<br>6 Weizmann St.<br>Tel Aviv, 6423906<br>ISRAEL
4205	Helsinki Committee of Rabin Medical Center, Beilinson Hospital<br>39 Jabotinski St.<br>Petah Tikva, 4941492<br>ISRAEL
4206	Helsinki Committee of Hadassah Medical Organization, Hadassah Medical Center, Ein-Karem<br>Kiryat Hadassah<br>Jerusalem, 9112001<br>ISRAEL
4207	Helsinki Committee of Soroka University Medical Center<br>Itzchak Rager Blvd.<br>Be’er-Sheva, 8410101<br>ISRAEL
4208	Helsinki Committee of the Chaim Sheba Medical Center<br>NAP<br>Tel-Hashomer, 5262100<br>ISRAEL

ITALY

Study Site Number	Independent Ethics Committee or Institutional Review Board Address(es)
4101	Comitato Etico Azienda Ospedaliera Universitaria Policlinico S. Orsola-Malpighi<br>Via P. Albertoni 15<br>Bologna, BO 40138<br>ITALY
4102	Comitato Etico Area Vasta Nord Ovest Azienda Ospedaliero Universitaria Careggi<br>Largo Brambilla, 3<br>Firenze, 50134<br>ITALY
4106	Comitato Etico Regionale delle Marche<br>Via conca 71<br>Torrette di Ancona, ANCONA 60020<br>ITALY
4107	COMITATO ETICO INDIPENDENTE ISTITUTO CLINICO HUMANITAS<br>Via Manzoni 56<br>Rozzano (MI), MILAN 20089<br>ITALY
4109	Comitato Etico Ospedale San Raffaele<br>Via Olgettina, 60<br>Milano, MI 20132<br>ITALY
4112	Comitato Etico Indipendente Fondazione PTV Policlinico Tor Vergata<br>Viale Oxford, 81<br>Roma, ROMA 00133<br>ITALY
4113	Comitato Etico Provinciale di Modena<br>Via Del Pozzo, 71<br>Modena, MODENA 41124<br>ITALY

Study Site Number	Independent Ethics Committee or Institutional Review Board Address(es)
4114	Comitato Etico degli IRCCS Istituto Europeo di Oncologia e Centro Cardiologico Monzino<br>Via Ripamonti 435<br>Milano, MILANO 20141<br>ITALY
4115	Comitato Etico Seconda Universita degli Studi di Napoli - AO-SUN-AORN "Ospedale Dei Colli"<br>Via Costantinopoli, 104<br>Napoli, NAPOLI 80138<br>ITALY
4117	Comitato Etico della Provincia di Bergamo c/o ASST Papa Giovanni XXIII<br>Piazza OMS 1<br>Bergamo, BG 24127<br>ITALY
4118	Comitato Etico Bergamo<br>Piazza OMS 1<br>Bergamo, BERGAMO 24127<br>ITALY
4119	Comitato Etico per la Sperimentazione Clinica (CESC) Istituto Oncologico Veneto IRCCS<br>Palazzo Santo Stefano<br>Piazza Antonore, 3<br>Padova, PD 35121<br>ITALY
4120	Comitato Etico Area Vasta Nord Ovest c/o Azienda Ospedaliero Universitaria Pisana<br>via Roma, 67<br>Pisa, PISA 56126<br>ITALY
4122	Comitato Etico Val Padana<br>Viale Concordia, 1<br>Cremona, CR 26100<br>ITALY

Study Site Number	Independent Ethics Committee or Institutional Review Board Address(es)
4123	Comitato Etico Indipendente AOU di Cagliari<br>Via Ospedale, 54<br>Cagliari, CAGLIARI 09124<br>ITALY
4124	Comitato Etico della Fondazione IRCCS Istituto Nazionale dei Tumori<br>Via Giacomo Venezian, 1<br>Milano, MI 20133<br>ITALY
4125	Comitato Etico Milano Area 3 ASST Grande Ospedale Metropolitano Niguarda<br>Piazza Ospedale Maggiore 3<br>Milano, MILANO 20162<br>ITALY

JAPAN

Study Site Number	Independent Ethics Committee or Institutional Review Board Address(es)
5501	National Cancer Center Institutional Review Board<br>5-1-1, Tsukiji<br>Chuo-ku, TOKYO 104-0045<br>JAPAN
	National Canter Center Institutional Review Board<br>6-5-1, Kashiwanoha<br>Kashiwa-shi, CHIBA 277-8577<br>JAPAN
5502	Aichi Cancer Center Hospital Institutional Review Board<br>1-1 Kanokoden, Chikusa-ku<br>Nagoya, AICHI 464-8681<br>JAPAN
5503	National Cancer Center EAST Institutional Review Board<br>6-5-1 Kashiwanoha<br>Kashiwa, CHIBA 277-8577<br>JAPAN
	National Cancer Center Hospital Institutional Review Board<br>5-1-1 Tsukiji<br>Chuo-ku, TOKYO 104-0045<br>JAPAN
5504	Osaka University Hospital Institutional Review Board<br>2-15 Yamadaoka<br>Suita, OSAKA 565-0871<br>JAPAN
5505	Kanagawa Cancer Center Institutional Review Board<br>Gastroenterological surgery<br>2-3-2 Nakao, Asahiku<br>Yokohama, KANAGAWA 241-8515<br>JAPAN

Study Site Number	Independent Ethics Committee or Institutional Review Board Address(es)
5506	National Hospital Organization Kyushu Cancer Center Institutional Review Board<br>3-1-1 Notame, Minami-ku<br>Fukuoka, FUKUOKA 811-1395<br>JAPAN
5507	Osaka National Hospital Institutional Review Board<br>2-1-14 Hoenzaka, Chuo-ku<br>Osaka, OSAKA 540-0006<br>JAPAN
5508	Hokkaido University Hospital Institutional Review Board<br>Kita14 Nishi5 Kita-ku<br>Sapporo, HOKKAIDO 060-8648<br>JAPAN
5509	St. Marianna University Goup Institution Review Board<br>2-16-1 Sugao Miyamae-ku<br>Kawasaki, KANAGAWA 216-8511<br>JAPAN
5510	Kanazawa University Hospital IRB<br>13-1, Takaramachi<br>Kanazawa, ISHIKAWA 920-8641<br>JAPAN

SOUTH KOREA (REPUBLIC OF KOREA)

Study Site Number	Independent Ethics Committee or Institutional Review Board Address(es)
4301	IRB of Korea University Anam Hospital<br>73, Inchon-ro, Seongbuk-gu<br>Seoul, 02841<br>SOUTH KOREA (REPUBLIC OF KOREA)
4302	IRB of Korea University Guro Hospital<br>148, Gurodong-ro, Guro-gu<br>Seoul, 08308<br>SOUTH KOREA (REPUBLIC OF KOREA)
4303	IRB of Seoul National University Bundang Hospital<br>82, Gumi-ro 173 Beon-gil, Bundang-gu<br>Seongnam-si, GYEONGGI-DO 13620<br>SOUTH KOREA (REPUBLIC OF KOREA)
4304	IRB of Seoul National University Hospital<br>101, Daehak-ro, Jongno-gu<br>Seoul, 03080<br>SOUTH KOREA (REPUBLIC OF KOREA)
4305	IRB of Dong-A University Hospital<br>26, Daesingongwon-ro, Seo-gu<br>Busan, 49201<br>SOUTH KOREA (REPUBLIC OF KOREA)
4306	Severance Hospital, Yonsei University Health System IRB<br>50-1, Yonsei-ro, Seodaemun-gu<br>Seoul, 03722<br>SOUTH KOREA (REPUBLIC OF KOREA)
4307	The Catholic University of Korea Seoul St. Mary's Hospital Institutional Review Board<br>222 Banpo-daero, Seocho-gu<br>Seoul, 06591<br>SOUTH KOREA (REPUBLIC OF KOREA)

Study Site Number	Independent Ethics Committee or Institutional Review Board Address(es)
4308	Gachon University Gil Medical Center IRB<br>21, Namdong-daero 774 beon-gil, Namdong-gu<br>Incheon, 21565<br>SOUTH KOREA (REPUBLIC OF KOREA)
4310	IRB of Chonnam National University Hwasun Hospital<br>322, Seoyang-ro, Hwasun-eup<br>Hwasun-gun, JEOLLANAM-DO 58128<br>SOUTH KOREA (REPUBLIC OF KOREA)
	IRB of Seoul National University Bundang Hospital (Inactive IRB/IEC)<br>82, Gumi-ro 173 Beon-gil, Bundang-gu<br>Seongnam-Si, GYEONGGI-DO 13620<br>SOUTH KOREA (REPUBLIC OF KOREA)
4311	IRB of Hallym University Sacred Heart Hospital<br>22, Gwanpyeong-ro 170beon-gil, Dongan-gu<br>Anyang-si, 14068<br>SOUTH KOREA (REPUBLIC OF KOREA)
4312	IRB of Samsung Medical Center<br>81, Irwon-ro, Gangnam-gu<br>Seoul, 06351<br>SOUTH KOREA (REPUBLIC OF KOREA)
4313	IRB of Asan Medical Center<br>88, Olympic-ro 43-gil, Songpa-gu<br>Seoul, 05505<br>SOUTH KOREA (REPUBLIC OF KOREA)
4315	IRB of Ajou University Hospital<br>164, Worldcup-ro, Yeongtong-gu,<br>Suwon-si, GYEONGGI-DO 16499<br>SOUTH KOREA (REPUBLIC OF KOREA)

MEXICO

Study Site Number	Independent Ethics Committee or Institutional Review Board Address(es)
4401	Comite de Etica en Investigacion de Inter Hosp, S.A. de C.V./Centro Medico Dalinde<br>Tuxpan 25, Colonia Roma Sur<br>Delegacion Cuauhtemoc, CIUDAD DE MEXICO 06760<br>MEXICO
4402	Comite de Etica en Investigacion de Inter Hosp, S.A. de C.V./Centro Medico Dalinde<br>Tuxpan 25, Colonia Roma Sur, Delegacion Cuauhtemoc<br>Cuidad de Mexico, 06760<br>MEXICO

NETHERLANDS

Study Site Number	Independent Ethics Committee or Institutional Review Board Address(es)
4501	METC AVL<br>Plesmanlaan 121<br>Amsterdam, NOORD HOLLAND 1066 CX<br>NETHERLANDS
4503	Medical Ethical Committee (METC)<br>Antoni van Leeuwenhoek (AvL)<br>Amsterdam, NOORD-HOLLAND 1066 CX<br>NETHERLANDS
4504	METC AVL<br>Plesmanlaan 121<br>Amsterdam, NOORD-HOLLAND 1066 CX<br>NETHERLANDS
4505	Medical Ethical Committee (METC)<br>Antoni van Leeuwenhoek (AvL), Plesmanlaan 121<br>Amsterdam, NOORD-HOLLAND 1066 CX<br>NETHERLANDS
4507	Medical Ethical Committee (METC)<br>Antoni van Leeuwenhoek (AvL), Plesmanlaan 121<br>Amsterdam, NOORD-HOLLAND 1066 CX<br>NETHERLANDS

NORWAY

Study Site Number	Independent Ethics Committee or Institutional Review Board Address(es)
4601	Regionale Komiteer for Medisinsk og Helsefaglig Forskningsetikk (REK Soer-Oest)<br>Gullhaugveien 1-3<br>Oslo, 0484<br>NORWAY<br><br>Regionale Komiteer for Medisinsk og Helsefaglig Forskningsetikk (REK Soer-Oest)<br>Postbox 1130 Blinderen<br>Oslo, 0318<br>NORWAY

POLAND

Study Site Number	Independent Ethics Committee or Institutional Review Board Address(es)
4702	Komisja Bioetyczna przy Okregowej Izbie Lekarskiej w Krakowie<br>Ul. Krupnicza 11a<br>Krakow, 31-123<br>POLAND
4703	Komisja Bioetyczna przy Okregowej Izbie Lekarskiej w Krakowie<br>ul. Krupnicza 11a<br>Kraków, 31-123<br>POLAND
4704	Komisja Bioetyczna przy Okregowej Izbie Lekarskiej w Krakowie<br>ul. Krupnicza 11a<br>Krakow, 31-123<br>POLAND
4705	Komisja Bioetyczna przy Okregowej Izbie Lekarskiej w Krakowie<br>ul. Krupnicza 11a<br>Krakow, 31-123<br>POLAND
4707	Komisja Bioetyczna przy Okregowej Izbie Lekarskiej w Krakowie<br>ul. Krupnicza 11a<br>Krakow, 31-123<br>POLAND
4708	Komisja Bioetyczna przy Okregowej Izbie Lekarskiej w Krakowie<br>ul. Krupnicza 11a<br>Krakow, 31-123<br>POLAND

RUSSIAN FEDERATION

Study Site Number	Independent Ethics Committee or Institutional Review Board Address(es)
4812	Ethics Committee of MRNTS n.a. A.F. Tsyb - branch FGBU"NMIRTS" of Minzdrav of Russia<br>Koroleva street 4<br>Obninsk, 249036<br>RUSSIAN FEDERATION
4816	LEC of LLC "EVIMED"<br>9V Bliukhera Ul.<br>Chelyabinsk, 454048<br>RUSSIAN FEDERATION
4817	N.N. Blokhin Russian Cancer Research Center Ethics Committee<br>23 Kashirskoye shosse, 24 Kashirskoye shosse<br>Moscow, 115478<br>RUSSIAN FEDERATION
4822	Ethics Committee of Pavlov First Saint Petersburg State Medical University<br>6-8, ul. Lva Tolstogo, lit. Ch<br>St. Petersburg, 198022<br>RUSSIAN FEDERATION
4828	N.N. Blokhin Russian Cancer Research Center Ethics Committee<br>23 Kashirskoye shosse, 24 Kashirskoye shosse<br>Moscow, 115478<br>RUSSIAN FEDERATION
4829	EC of OBUZ "Kursk Regional Clinical Oncology Dispensary"<br>20 Pirogova Str<br>Kursk, 305 035<br>RUSSIAN FEDERATION

SPAIN

Study Site Number	Independent Ethics Committee or Institutional Review Board Address(es)
4901	Comite Etico de Investigacion Clinica Corporacion Sanitaria Parc Tauli<br>Fundacio Parc Tauli Edifici Santa Fe Ala izquierda, 2a planta<br>Sabadell, BARCELONA 08208<br>SPAIN
4902	Comite Etico de Investigacion Clinica Corporacion Sanitaria Parc Tauli<br>Fundacio Parc Tauli Edifici Santa Fe Ala izquierda, 2a planta, C/ Parc Tauli, 1<br>Sabadell, BARCELONA 08208<br>SPAIN
4903	Comite Etico de Investigacion Clinica Corporacion Sanitaria Parc Tauli<br>Fundacio Parc Tauli Edifici Santa Fe Ala izquierda, 2a planta, C/ Parc Tauli, 1<br>Sabadell, BARCELONA 08208<br>SPAIN
4904	Comite Etico de Investigacion Clinica Corporacio Sanitaria Parc Tauli<br>Fundacio Parc Tauli Edifici Santa Fe Ala izquierda, 2a planta, C/ Parc Tauli, 1<br>Sabadell, BARCELONA 08208<br>SPAIN
4905	Comite Etico de Investigacion Clinica Corporacio Sanitaria Parc Tauli<br>Fundacio Parc Tauli Edifici Santa Fe Ala izquierda, 2a planta, C/ Parc Tauli, 1<br>Sabadell, BARCELONA 08208<br>SPAIN
4906	Comite Etico de Investigacion Clinica Corporacion Sanitaria Parc Tauli<br>Fundacio Parc Tauli Edifici Santa Fe Ala izquierda, 2a planta, C/ Parc Tauli, 1<br>Sabadell, BARCELONA 08208<br>SPAIN
4907	Comite Etico de Investigacion Clinica Corporacio Sanitaria Parc Tauli<br>Fundacio Parc Tauli Edifici Santa Fe Ala izquierda, 2a planta, C/ Parc Tauli, 1<br>Sabadell, BARCELONA 08208<br>SPAIN

Study Site Number	Independent Ethics Committee or Institutional Review Board Address(es)
4908	Comite Etico de Investigacion Clinica Corporacio Sanitaria Parc Tauli<br>Fundacio Parc Tauli Edifici Santa Fe Ala izquierda, 2a planta, C/ Parc Tauli, 1<br>Sabadell, BARCELONA 08208<br>SPAIN
4909	Comite Etico de Investigacion Clinica Corporacio Sanitaria Parc Tauli<br>Fundacio Parc Tauli Edific Santa Fe Ala izquierda, 2a planta, C/ Parc Tauli, 1<br>Sabadell, BARCELONA 08208<br>SPAIN
4910	Comite Etico de Investigacion Clinica Corporacio Sanitaria Parc Tauli<br>Fundacio Parc Tauli Edific Santa Fe Ala izquierda, 2a planta, C/ Parc Tauli, 1<br>Sabadell, BARCELONA 08208<br>SPAIN
4911	Comite Etico de Investigacion Clinica Corporacion Sanitaria Parc Tauli<br>Fundacio Parc Tauli Edifici Santa Fe Ala izquierda, 2a planta, C/ Parc Tauli, 1<br>Sabadell, BARCELONA 08208<br>SPAIN
4912	Comite Etico de Investigacion Clinica Corporacio Sanitaria Parc Tauli<br>Fundacio Parc Tauli Edific Santa Fe Ala izquierda, 2a planta, C/ Parc Tauli, 1<br>Sabadell, BARCELONA 08208<br>SPAIN
4913	Comite Etico de Investigacion Clinica Corporacio Sanitaria Parc Tauli<br>Fundacio Parc Tauli Edifici Santa Fe Ala izquierda, 2a planta, C/ Parc Tauli, 1<br>Sabadell, BARCELONA 08208<br>SPAIN
4914	Comite Etico de Investigacion Clinica Corporacio Sanitaria Parc Tauli<br>Fundacio Parc Tauli Edifici Santa Fe Ala izquierda, 2a planta, C/ Parc Tauli, 1<br>Sabadell, BARCELONA 08208<br>SPAIN

Study Site Number	Independent Ethics Committee or Institutional Review Board Address(es)
4915	Comite Etico de Investigacion Clinica Corporacio Sanitaria Parc Tauli<br>Fundacio Parc Tauli Edifici Santa Fe Ala izquierda, 2a planta, C/ Parc Tauli, 1<br>Sabadell, BARCELONA 08208<br>SPAIN
4916	Comite Etico de Investigacion Clinica Corporacio Sanitaria Parc Tauli<br>Fundacio Parc Tauli Edifici Santa Fe Ala izquierda, 2a planta, C/ Parc Tauli, 1<br>Sabadell, BARCELONA 08208<br>SPAIN
4917	Comite Etico de Investigacion Clinica Corporacion Sanitaria Parc Tauli<br>Fundacio Parc Tauli Edifici Santa Fe Ala izquierda, 2a planta, C/ Parc Tauli, 1<br>Sabadell, BARCELONA 08208<br>SPAIN
4918	Comite Etico de Investigacion Clinica Corporacio Sanitaria Parc Tauli<br>Fundacio Parc Tauli Edific Santa Fe Ala izquierda, 2a planta, C/ Parc Tauli, 1<br>Sabadell, BARCELONA 08208<br>SPAIN
4919	Comite Etico de Investigacion Clinica Corporacio Sanitaria Parc Tauli<br>Fundacio Parc Tauli Edifici Santa Fe Ala izquierda, 2a planta, C/ Parc Tauli, 1<br>Sabadell, BARCELONA 08208<br>SPAIN
4921	Comite Etico de Investigacion Clinica Corporacio Sanitaria Parc Tauli<br>Fundacio Parc Tauli Edifici Santa Fe Ala izquierda, 2a planta, C/ Parc Tauli, 1<br>Sabadell, BARCELONA 08208<br>SPAIN
4922	Comite Etico de Investigacion Clinica Corporacio Sanitaria Parc Tauli<br>Fundacio Parc Tauli Edific Santa Fe Ala izquierda, 2a planta, C/ Parc Tauli, 1<br>Sabadell, BARCELONA 08208<br>SPAIN

TAIWAN

Study Site Number	Independent Ethics Committee or Institutional Review Board Address(es)
5001	Institutional Review Board-II Kaohsiung Medical University Chung-Ho Memorial Hospital<br>No. 100 Tzyou 1st Road<br>Kaohsiung, 807<br>TAIWAN
5002	Chang Gung Medical Foundation Institutional Review Board<br>No. 199, Tung Hwa North Road<br>Taipei, 10507<br>TAIWAN
5003	Institutional Review Board National Cheng Kung University Hospital<br>138 Sheng-Li Rd.,<br>Tainan, 704<br>TAIWAN
5004	Research Ethics Committee China Medical University & Hospital<br>No. 2, Yude Road<br>Taichung, 40447<br>TAIWAN
5005	Research Ethics Committee A, National Taiwan University Hospital<br>7, Chung-Shan South Road<br>Taipei, 100<br>TAIWAN
5006	Institution Review Board Committee A, Changhua Christian Hospital<br>135, Nanxiao St.<br>Changhua City, CHANGHUA COUNTY 500<br>TAIWAN

TURKEY

Study Site Number	Independent Ethics Committee or Institutional Review Board Address(es)
5101	Republic of Turkey Trakya University Edirne Clinical Research Ethics Committee (Inactive IRB/IEC)<br>Trakya Universitesi Balkan Yerleskesi Tip Fakultesi<br>Temel Bilimler Binasi D1, Blok Kat 3<br>Edirne, 22030<br>TURKEY<br><br>Trakya University Medical Faculty Clinical Research Ethics Committee<br>Trakya Universitesi Balkan Yerleskesi Tip Fakultesi<br>Temel Bilimler Binasi D1, Blok Kat 3<br>Edirne, 22030<br>TURKEY
5102	Republic of Turkey Trakya University Edirne Clinical Research Ethics Committee (Inactive IRB/IEC)<br>Trakya Universitesi BalkanYerleskesi Tip Fakultesi<br>Temel Bilimler Binasi D1, Blok Kat 3<br>Edirne, 22030<br>TURKEY<br><br>Trakya University Medical Faculty Clinical Research Ethics Committee<br>Trakya Universitesi Balkan Yerleskesi Tip Fakultes<br>Edirne, 22030<br>TURKEY
5103	Republic of Turkey Trakya University Edirne Clinical Research Ethics Committee<br>Trakya Universitesi Balkan Yerleskesi Tip Fakultesi<br>Temel Bilimler Binasi D1, Blok Kat 3<br>Edirne, 22030<br>TURKEY
5104	Republic of Turkey Trakya University Edirne Clinical Research Ethics Committee<br>Trakya Universitesi Balkan Yerleskesi Tip Fakultesi<br>Temel Bilimler Binasi D1, Blok Kat 3<br>Edirne, 22030<br>TURKEY

Study Site Number	Independent Ethics Committee or Institutional Review Board Address(es)
5105	Republic of Turkey Trakya University Edirne Clinical Research Ethics Committee<br>Trakya Universitesi BalkanYerleskesi Tip Fakultesi<br>Temel Bilimler Binasi D1, Blok Kat 3<br>Edirne, 22030<br>TURKEY
5106	Republic of Turkey Trakya University Edirne Clinical Research Ethics Committee<br>Trakya Universitesi BalkanYerleskesi Tip Fakultesi<br>Temel Bilimler Binasi D1, Blok Kat 3<br>Edirne, 22030<br>TURKEY
5107	Republic of Turkey Trakya University Edirne Clinical Research Ethics Committee<br>Trakya Universitesi BalkanYerleskesi Tip Fakultesi<br>Temel Bilimler Binasi D1, Blok Kat 3<br>Edirne, 22030<br>TURKEY

UKRAINE

Study Site Number	Independent Ethics Committee or Institutional Review Board Address(es)
5201	Komisiia z pytan etyky pry Kyivskii klinichnii likarni na zaliznychnomu transporti No3<br>vul. Simferopolska 8<br>Kyiv, KYIVSKA OBLAST' 02096<br>UKRAINE
5202	Komissia z pytan etyky pry Tsentralnii miskii klinchnii likarni m. Uzhgorod<br>vul. Hryboiedova, 20<br>Uzhgorod, ZAKARPATS'KA OBLAST' 88000<br>UKRAINE
5203	Komisiia z pytan etyky KZ, Dnipropetrovska miska bahatoprofilna klinichna likarnia Nº4<br>Dnipropetrovskoi oblasnoi rady<br>vul. Blyzhnia, 31<br>Dnipropetrovsk, DNIPROPETROVSKA OBLAST' 49102<br>UKRAINE
5212	Komisiia z pytan etyky, Vinnytskyi oblasnyi klinichnyi onkolohichnyi dyspanser<br>Khmelnytske shose 84<br>Vinnytsia, VINNYTS'KA OBLAST' 21029<br>UKRAINE

UNITED KINGDOM

Study Site Number	Independent Ethics Committee or Institutional Review Board Address(es)
5301	Health Research Authority - East of England - Cambridge South Research Ethics Committee<br>The Old Chapel<br>Nottingham, NOTTINGHAMSHIRE NG1 6FS<br>UNITED KINGDOM
5304	Health Research Authority - East of England - Cambridge South Research Ethics Committee<br>The Old Chapel, Royal Standard Place<br>Nottingham, NOTTINGHAMSHIRE NG1 6FS<br>UNITED KINGDOM<br><br>Joint Research Compliance Office, Imperial College London (Inactive IRB/IEC)<br>Room 221, Medical School Building, St. Mary's Campus, Norfolk Place<br>London, LONDON W2 1PG<br>UNITED KINGDOM
5306	Health Research Authority - East of England - Cambridge South Research Ethics Committee<br>The Old Chapel, Royal Standard Palace<br>Nottingham, NOTTINGHAMSHIRE NG1 6FS<br>UNITED KINGDOM
5308	Health Research Authority - North West - Haydock Research Ethics Committee<br>3rd Floor - Barlow House, 4 Minshull Street<br>Manchester, MANCHESTER M1 3DZ<br>UNITED KINGDOM
5309	Health Research Autority - East of England - Cambridge South Research Ethics Commitee<br>The Old Chapel, Royal Standard Place<br>Nottingham, NOTTINGHAM NG1 6FS<br>UNITED KINGDOM
5310	Health Research Authority - East of England - Cambridge South Research Ethics Committee<br>The Old Chapel, Royal Standard Place<br>Nottingham, NOTTINGHAMSHIRE NG1 6FS<br>UNITED KINGDOM

Study Site Number	Independent Ethics Committee or Institutional Review Board Address(es)
5311	Health Research Authority - East of England - Cambridge South Research Ethics Committee<br>The Old Chapel, Royal Standard Palace<br>Nottingham, NOTTINGHAMSHIRE NG1 6FS<br>UNITED KINGDOM

UNITED STATES

Study Site Number	Independent Ethics Committee or Institutional Review Board Address(es)
5401	Vanderbilt University Institutional Review Board<br>1313 21st Ave S, 504 Oxford House<br>Nashville, TN 37232<br>UNITED STATES
5402	Memorial Sloan Kettering Institutional Review Board<br>1275 York Avenue<br>New York, NY 10065<br>UNITED STATES
5403	Western Institutional Review Board<br>1019 39th Avenue SE,Suite 120<br>Puyallup, WASHINGTON 98374-2115<br>UNITED STATES
5406	Mayo Clinic Institutional Review Board<br>200 First St SW,Bldg 201 Rm 4-60<br>Rochester, MN 55905<br>UNITED STATES
5409	Western Institutional Review Board<br>1019 39th Ave SE,,Ste 120<br>Puyallup, WA 98374-2115<br>UNITED STATES
5412	Broward Health Institutional Review Board-Broward Health Medical Center<br>1600 S Andrews Ave<br>Fort Lauderdale, FL 33316<br>UNITED STATES
5413	WESTERN INSTITUTIONAL REVIEW BOARD<br>1019 39st Ave SE,Ste 120<br>Puyallup, WA 98374-2115<br>UNITED STATES

Study Site Number	Independent Ethics Committee or Institutional Review Board Address(es)
5414	WESTERN INSTITUTIONAL REVIEW BOARD<br>1019 39st Ave SE,Ste 120<br>Puyallup, WA 98374-2115<br>UNITED STATES
5415	OHSU Institutional Review Board<br>3181 Sw Sam Jackson Park Rd,L106-RI<br>Portland, OR 97239<br>UNITED STATES
5417	WESTERN INSTITUTIONAL REVIEW BOARD<br>1019 39st Ave SE,Ste 120<br>Puyallup, WA 98374-2115<br>UNITED STATES
5419	WESTERN INSTITUTIONAL REVIEW BOARD<br>1019 39TH AVE SE,Ste 120<br>PUYALLUP, WA 98374-2115<br>UNITED STATES
5421	Western Institutional Review Board<br>1019 39th Avenue SE,Suite 120<br>Puyallup, WASHINGTON 98374-2115<br>UNITED STATES
5423	Saint Louis University Institutional Review Board<br>3556 Caroline Street, Caroline Building, C110<br>St. Louis, MO 63104<br>UNITED STATES
	WESTERN INSTITUTIONAL REVIEW BOARD<br>1019 39th Ave SE,Ste 120<br>Puyallup, WA 98374-2115<br>UNITED STATES

Study Site Number	Independent Ethics Committee or Institutional Review Board Address(es)
5427	University of Southern California (USC) Institutional Review Board<br>1640 Marengo Street,Suite 700<br>Los Angeles, CA 90033<br>UNITED STATES<br><br>University of Southern California Health Sciences Campus Institutional Review Board (Inactive IRB/IEC)<br>1200 North State Street,LAC+USC Medical Center, General Hospital, Suite 4700<br>Los Angeles, CA 90033<br>UNITED STATES
5428	WESTERN INSTITUTIONAL REVIEW BOARD<br>1019 39th Ave SE,Ste 120<br>Puyallup, WA 98374-2115<br>UNITED STATES
5431	Cleveland Clinic Institutional Review Board<br>9500 Euclid Ave,OS-1<br>Cleveland, OH 44195<br>UNITED STATES
5432	WESTERN INSTITUTIONAL REVIEW BOARD<br>1019 39TH AVE SE,Ste 120<br>PUYALLUP, WA 98374-2115<br>UNITED STATES
5433	WESTERN INSTITUTIONAL REVIEW BOARD<br>1019 39th Ave SE,Ste 120<br>Puyallup, WA 98374-2115<br>UNITED STATES
5434	University of Iowa IRB-01, Human Subjects Office<br>105 Hardin Library for the Health Sciences, 600 Newton Road<br>Iowa City, IA 52242<br>UNITED STATES

Study Site Number	Independent Ethics Committee or Institutional Review Board Address(es)
5435	Mayo Clinic Institutional Review Board<br>200 First St Sw,201 Bldg - Rm 4-60<br>Rochester, MN 55905<br>UNITED STATES
5436	University of Chicago Institutional Review Board<br>5751 S. Woodlawn Ave.,McGiffert Hall, 2nd fl<br>Chicago, IL 60637<br>UNITED STATES
5437	The University of Texas MD Anderson Cancer Center Institutional Review Board<br>7007 Bertner Avenue, Unit 1637<br>Houston, TEXAS 77030-4009<br>UNITED STATES
5440	Johns Hopkins Medicine Institutional Review Board<br>1620 McElderry St,Reed Hall, B-130<br>Baltimore, MD 21205-1911<br>UNITED STATES
5441	Mayo Clinic Institutional Review Board<br>200 First St SW<br>Rochester, MN 55905<br>UNITED STATES
5443	Human Subjects Committee<br>University of Kansas Medical Center<br>3901 Rainbow Boulevard<br>Kansas City, KS 66160<br>UNITED STATES
5444	Institutional Review Board, Dana-Farber Cancer Institute/Dana-Farber/Harvard Cancer Center<br>450 Brookline Ave,OS229<br>Boston, MA 02215<br>UNITED STATES

Study Site Number	Independent Ethics Committee or Institutional Review Board Address(es)
5445	Washington University in St. Louis - Human Research Protection Office<br>660 S Euclid Ave,Campus Box #8089<br>St. Louis, MO 63110<br>UNITED STATES
5447	WESTERN INSTITUTIONAL REVIEW BOARD<br>1019 39th Ave SE,Ste 120<br>Puyallup, WA 98374-2115<br>UNITED STATES
5449	Ochsner Clinic Foundation Institutional Review Board<br>1514 Jefferson Hwy<br>New Orleans, LA 70121<br>UNITED STATES
5450	Baylor Scott and White Research Institute Institutional Review Board-Gold<br>2401 S 31st St<br>Temple, TX 76508<br>UNITED STATES
5452	WESTERN INSTITUTIONAL REVIEW BOARD<br>1019 39st Ave SE,Ste 120<br>Puyallup, WA 98374-2115<br>UNITED STATES

Clinical Study ARRAY-818-302

The BEACON CRC Study (Binimetinib, Encorafenib, And Cetuximab COmbiNed to Treat *BRAF*-mutant ColoRectal Cancer):

A Multicenter, Randomized, Open-label, 3-Arm Phase 3 Study of Encorafenib + Cetuximab Plus or Minus Binimetinib vs. Irinotecan/Cetuximab or Infusional 5-Fluorouracil (5-FU)/Folinic Acid (FA) /Irinotecan (FOLFIRI)/Cetuximab with a Safety Lead-in of Encorafenib + Binimetinib + Cetuximab in Patients with *BRAF* V600E-mutant Metastatic Colorectal Cancer

Protocol Version 1.0	26 April 2016
Protocol Version 2.0	18 July 2016
Protocol Version 3.0	16 May 2017
Protocol Version 4.0	05 September 2017
Protocol Version 5.0	11 April 2018
Protocol Version 6.0	19 September 2018
Protocol Version 7.0	25 January 2019
Protocol Version 8.0	11 July 2019
EudraCT Number:	2015-005805-35
IND Number:	115298

Array BioPharma Inc.

3200 Walnut Street
Boulder, CO 80301
Phone: (303) 381-6600
Fax: (303) 386-1240

CONFIDENTIAL INFORMATION

This document contains trade secrets and other confidential and proprietary information belonging to Array BioPharma Inc. Except as otherwise agreed to in writing, by accepting or reviewing this document you agree to hold this information in confidence and not to disclose it to others (except where required by applicable law) or to use it for unauthorized purposes. In the event of any actual or suspected breach of this obligation, Array BioPharma Inc. should be promptly notified.

SIGNATURE PAGE (SPONSOR)

I have read and understand the contents of the clinical protocol for Clinical Study ARRAY-818-302 dated 11 July 2019 and agree to meet all obligations of Array BioPharma Inc. as detailed in all applicable regulations and guidelines. In addition, I will ensure that the Investigators are informed of all relevant information that becomes available during the conduct of this study.

Victor Sandor, MD
Chief Medical Officer
Array BioPharma Inc.

July 16/2019
Date

PRINCIPAL INVESTIGATOR AGREEMENT

I have read and understand the contents of the clinical protocol for Clinical Study ARRAY-818-302 dated 11 July 2019 and will adhere to the study requirements as presented, including all statements regarding confidentiality. In addition, I will conduct the study in accordance with the requirements of this protocol and also protect the rights, safety, privacy and well-being of study patients in accordance with the following:

- International Conference on Harmonisation (ICH) of Technical Requirements for Registration of Pharmaceuticals for Human Use Harmonised Tripartite Guideline for Good Clinical Practice (GCP) E6(R1)
- All applicable laws and regulations, including, without limitation, data privacy laws and regulations
- Requirements for reporting serious adverse events (SAEs) defined in Section 10.9 of this protocol
- Terms outlined in the Clinical Study Site Agreement

My signature also acknowledges that:

- Neither my subinvestigators nor I are members of the Ethics Committee (EC) reviewing this protocol, or
- I and/or my subinvestigators are members of the EC, but I/we will not participate in the initial review or continuing review of this study

______________________________ ______________________________

Name of Principal Investigator Signature of Principal Investigator

____

Date

PROTOCOL SYNOPSIS

**Title**	A Multicenter, Randomized, Open-label, 3-Arm Phase 3 Study of Encorafenib + Cetuximab Plus or Minus Binimetinib vs. Irinotecan/Cetuximab or Infusional 5-Fluorouracil (5-FU)/Folinic Acid (FA)/Irinotecan (FOLFIRI)/Cetuximab with a Safety Lead-in of Encorafenib + Binimetinib + Cetuximab in Patients with *BRAF* V600E-mutant Metastatic Colorectal Cancer
**Protocol Number**	ARRAY-818-302
**Phase**	3
**Study Centers**	Approximately 300 international study centers
**Objectives**	<u>**Safety Lead-in**</u><br>In patients with *BRAF* V600E-mutant (*BRAF*^{V600E}) metastatic colorectal cancer (mCRC):<br>**Primary:**<br>• Assess the safety/tolerability of the combination of encorafenib + binimetinib + cetuximab<br>**Secondary:**<br>• Assess the activity of encorafenib + binimetinib + cetuximab as measured by blinded independent central review (BICR)-determined and Investigator-determined objective response rate (ORR), duration of response (DOR), progression-free survival (PFS) and time to response<br>• Characterize the pharmacokinetics (PK) of encorafenib, cetuximab, binimetinib and the active metabolite of binimetinib (AR00426032)<br>**Exploratory:**<br>• Assess the activity of encorafenib + binimetinib + cetuximab as measured by overall survival (OS)<br><u>**Randomized Phase 3**</u><br>In patients with *BRAF*^{V600E} mCRC:<br>**Primary:**<br>• Compare the activity of encorafenib + binimetinib + cetuximab (Triplet Arm) vs. irinotecan/cetuximab or 5-fluorouracil (5-FU)/folinic acid (FA)/irinotecan (FOLFIRI)/cetuximab (Control Arm) as measured by overall survival (OS)

	• Compare the activity of encorafenib + binimetinib + cetuximab (Triplet Arm) vs. irinotecan/cetuximab or FOLFIRI/cetuximab (Control Arm) as measured by ORR per BICR<br>**Key Secondary:**<br>• Compare the activity of encorafenib + cetuximab (Doublet Arm) vs. irinotecan/cetuximab or FOLFIRI/cetuximab (Control Arm) as measured by OS<br>**Other Secondary:**<br>• Compare the Investigator-determined ORR of encorafenib + binimetinib + cetuximab (Triplet Arm) vs. irinotecan/cetuximab or FOLFIRI/cetuximab (Control Arm)<br>• Compare the BICR-determined and Investigator-determined ORR of encorafenib + cetuximab (Doublet Arm) vs. irinotecan/cetuximab or FOLFIRI/ cetuximab (Control Arm)<br>• Compare the BICR-determined and Investigator-determined progression-free survival (PFS) of encorafenib + binimetinib + cetuximab (Triplet Arm) vs. irinotecan/cetuximab or FOLFIRI/cetuximab (Control Arm)<br>• Compare the BICR-determined and Investigator-determined PFS of encorafenib + cetuximab (Doublet Arm) vs. irinotecan/cetuximab or FOLFIRI/cetuximab (Control Arm)<br>• Compare the activity of Triplet Arm vs. Doublet Arm as measured by OS<br>• Compare BICR-determined and Investigator-determined ORR of Triplet Arm vs. Doublet Arm<br>• Compare the BICR-determined and Investigator-determined PFS of Triplet Arm vs. Doublet Arm<br>• Compare BICR-determined and Investigator-determined DOR of Triplet Arm vs. Control Arm, of Doublet Arm vs. Control Arm and of Triplet Arm vs. Doublet Arm<br>• Compare BICR-determined and Investigator-determined time to response of Triplet Arm vs. Control Arm, of Doublet Arm vs. Control Arm and of Triplet Arm vs. Doublet Arm<br>• Assess the safety/tolerability of Triplet Arm, of Doublet Arm and of Control Arm<br>• Compare the effect on quality of life (QoL) of Triplet Arm vs. Control Arm, of Doublet Arm vs. Control Arm and of Triplet Arm vs. Doublet Arm

	• Characterize the PK of encorafenib, cetuximab, binimetinib and the active metabolite of binimetinib (AR00426032)<br>• Assess for drug interactions between encorafenib, cetuximab, binimetinib and the active metabolite of binimetinib (AR00426032) based on PK modeling<br>**Exploratory:**<br>• Assess the relationship between changes in tumor markers (carcinoembryonic antigen [CEA] and carbohydrate antigen 19-9 [CA19-9]) and radiographic response to treatment<br>• Assess blood- and tissue-based predictive biomarkers of activity
**Endpoints**	**<u>Safety Lead-in</u>**<br>**Primary:**<br>• Incidence of dose-limiting toxicities (DLTs)<br>• Incidence and severity of adverse events (AEs) graded according to the National Cancer Institute (NCI) Common Terminology Criteria for Adverse Events (CTCAE), version 4.03 (v.4.03), and changes in clinical laboratory parameters, vital signs, electrocardiograms (ECGs), echocardiogram (ECHO)/multi-gated acquisition (MUGA) scans and ophthalmic examinations<br>• Incidence of dose interruptions, dose modifications and discontinuations due to AEs<br>**Secondary:**<br>• ORR (by BICR and Investigator) per the Response Evaluation Criteria in Solid Tumors (RECIST), version 1.1 (v1.1), defined as the number of patients achieving an overall best response of complete response (CR) or partial response (PR) divided by the total number of patients<br>• DOR (by BICR and Investigator), defined as the time from first radiographic evidence of response to the earliest documented disease progression or death due to underlying disease<br>• PFS (by BICR and Investigator), defined as the time from first dose to the earliest documented disease progression or death due to any cause<br>• Time to response (by BICR and Investigator), defined as the time from first dose to first radiographic evidence of response

	• PK parameters of encorafenib, cetuximab, binimetinib and the active metabolite of binimetinib (AR00426032)<br>**Exploratory:**<br>• OS, defined as the time from first dose to death due to any cause<br><br>**<u>Randomized Phase 3</u>**<br>**Primary:**<br>• OS, defined as the time from randomization to death due to any cause, of Triplet Arm vs. Control Arm<br>• Confirmed ORR (by BICR) per RECIST, v1.1 of Triplet Arm vs. Control Arm<br>**Key Secondary:**<br>• OS of Doublet Arm vs. Control Arm<br>**Other Secondary:**<br>• Confirmed ORR (by Investigator) per RECIST, v1.1 of Triplet Arm vs. Control Arm<br>• Confirmed ORR (by BICR and Investigator) per RECIST, v1.1 of Doublet Arm vs. Control Arm<br>• PFS (by BICR and Investigator), defined as the time from randomization to the earliest documented disease progression or death due to any cause, of Triplet Arm vs. Control Arm<br>• PFS (by BICR and Investigator) of Doublet Arm vs. Control Arm<br>• OS of Triplet Arm vs. Doublet Arm<br>• Confirmed ORR (by BICR and Investigator) per RECIST, v1.1 of Triplet Arm vs. Doublet Arm<br>• PFS (by BICR and Investigator) of Triplet Arm vs. Doublet Arm<br>• DOR (by BICR and Investigator) of Triplet Arm vs. Control Arm, of Doublet Arm vs. Control Arm and of Triplet Arm vs. Doublet Arm<br>• Time to response (by BICR and Investigator), defined as the time from randomization to first radiographic evidence of response, of Triplet Arm vs. Control Arm, of Doublet Arm vs. Control Arm and of Triplet Arm vs. Doublet Arm

	• Incidence and severity of AEs, graded according to NCI CTCAE, v 4.03, and changes in clinical laboratory parameters, vital signs, ECGs, ECHO/MUGA scans and ophthalmic examinations<br>• Change from baseline in the European Organization for Research and Treatment of Cancer (EORTC) Quality of Life Questionnaire for Cancer Patients (QLQ-C30), Functional Assessment of Cancer Therapy-Colon Cancer (FACT-C), EuroQol-5D-5L (EQ-5D-5L), and Patient Global Impression of Change (PGIC) of Triplet Arm vs. Control Arm, of Doublet Arm vs. Control Arm and of Triplet Arm vs. Doublet Arm<br>• Model-based PK parameters of encorafenib, cetuximab, binimetinib and the active metabolite of binimetinib (AR00426032)<br>• Model-based PK assessment of drug-drug interactions between encorafenib, cetuximab, binimetinib and the active metabolite of binimetinib (AR00426032)<br>**Exploratory:**<br>• Changes in CEA and CA19-9<br>• Genomic and proteomic analysis of blood and tissue samples at baseline and at end of treatment (optional for tumor samples at end of treatment)
**Design**	This is a multicenter, randomized, open-label, 3-arm Phase 3 study to evaluate encorafenib + cetuximab plus or minus binimetinib versus Investigator's choice of either irinotecan/cetuximab or FOLFIRI/cetuximab, as controls, in patients with *BRAF*^{V600E} mCRC whose disease has progressed after 1 or 2 prior regimens in the metastatic setting. The study contains a Safety Lead-in Phase in which the safety and tolerability of encorafenib + binimetinib + cetuximab will be assessed prior to the Phase 3 portion of the study.<br>**BRAF Testing**<br>Patients will be eligible for the study based on identification of a *BRAF*^{V600E} mutation in the tumor as determined by the central laboratory as part of the Molecular Prescreening for the trial or by a local assay result obtained any time prior to Screening. Only polymerase chain reaction (PCR) and next generation sequencing (NGS)-based local assays results will be acceptable. If the patient is enrolled based on local assay results, the *BRAF* mutation status must be confirmed by the central laboratory no later than 30 days

	from first dose of study treatment.<br><br>In cases where there is discordance between the local assay and central laboratory results, or if the central laboratory is not able to confirm presence of a *BRAF*^{V600E} mutation due to inadequate or poor sample condition within 30 days of initiating study therapy, patients may only continue treatment if there is no clinical indication of deterioration or disease progression and the investigator determines that the patient is deriving benefit. In such instances, patients must be informed that the *BRAF* mutation status is unconfirmed and must sign a separate informed consent form that includes this information and describes alternative treatment options.<br><br>Central laboratory *BRAF* mutation tests with a definitive result (positive or negative) cannot be repeated to resolve a discordant result. Patients whose sample is determined to be inadequate or who have an indeterminate result on central testing may have samples resubmitted for testing. Lack of *BRAF*^{V600E} confirmation by the central laboratory may be due to discordance between the local assay and central laboratory results (potential false positive local assay results), or due to inadequate or poor sample condition for central testing (indeterminate results). If at any time in the study there is lack of *BRAF*^{V600E} confirmation in a total of 37 patients (6% of the total planned enrollment of the randomized portion of the trial) or discordance between the local assay and the central laboratory in 18 patients (3% of the total planned enrollment), all subsequent patients will be required to have *BRAF*^{V600E} determined by the central laboratory for treatment (i.e., local *BRAF* testing will no longer be accepted for trial eligibility). Information regarding sites and laboratories associated with discordant results will be maintained and results from laboratories with more than 1 prior discordant result will not be accepted. Sites with more than 2 randomized patients having indeterminate results after initiation of protocol version 6 will be required to enroll all subsequent patients based only on central laboratory assay results.<br><br>**Molecular Prescreening**<br><br>Prior to eligibility assessment for study enrollment/randomization, patients may undergo molecular tumor prescreening with the central laboratory *BRAF* mutation assay at any time prior to Screening as long as they meet all the Molecular Prescreening inclusion/exclusion criteria. Note that tumor samples previously determined to be *BRAF*wild-type (BRAF^{wt}) by local assessment may be submitted to the central laboratory. In particular, tumors with clinicopathological features of *BRAF* mutations may be

	considered for testing by central laboratory regardless of the results of prior local *BRAF* mutation testing.<br>**Safety Lead-in**<br>The Safety Lead-in will be conducted at a limited number of sites. Dose-limiting toxicities will be evaluated and the tolerability of the binimetinib, encorafenib, and cetuximab combination will be assessed by the Sponsor and the Investigator in (approximately) weekly communications. The Data Monitoring Committee (DMC) will evaluate the safety data at pre-specified intervals and at additional points during the conduct of the Safety Lead-in, if necessary. The first 9 evaluable patients will be enrolled on a rolling basis in a single cohort to evaluate the combination of encorafenib 300 mg once daily (QD) + binimetinib 45 mg twice daily (BID) + cetuximab 400 mg/m^2 followed by 250 mg/m^2 IV weekly. Additional patients will be enrolled based on assessments of the safety data by the DMC during the Safety Lead-in.<br>Following study treatment discontinuation, patients who provide informed consent for survival follow-up will continue to be assessed to determine survival status until withdrawal of consent, patient is lost to follow-up, death or defined end of study.<br>If informed consent cannot be obtained due to the patient being lost to follow-up or previous withdrawal of consent, attempts to determine survival status will be made via access to public records where permitted by local laws.<br>The doses for the Triplet Arm in the randomized Phase 3 portion of the study will be determined after a total of 25-30 patients have been treated at the proposed doses and their data evaluated by the DMC.<br>In the Japanese Safety Lead-in portion of the study, a separate cohort of patients will be evaluated at a limited number of sites in Japan. Six patients will be enrolled on a rolling basis in a single cohort to evaluate the triplet dose. The starting dose in Japanese patients will be the dose assessed to be tolerable in the Safety Lead-in for non-Japanese patients. Similar to the Safety Lead-in for non-Japanese patients, the DMC will evaluate the safety data to confirm tolerability.<br>**Phase 3**<br>Once the tolerability of the proposed doses for the Triplet Arm has been established, the Phase 3 portion of the study will begin and eligible patients will be randomized in a 1:1:1 ratio to the Triplet Arm, Doublet Arm or Control Arm. The number of 3rd-line patients (those having received 2 prior regimens) will be limited to 215 (35% of the total randomized) after which only patients with

	1 prior regimen will be randomized. Patients with 2 prior regimens who have entered Screening at the time that the limit has been reached will be permitted to continue into the study if they are otherwise determined to be eligible. Patients randomized to the Control Arm may be treated with either irinotecan + cetuximab or FOLFIRI + cetuximab as per Investigator's choice. The choice of irinotecan or FOLFIRI must be declared prior to randomization.<br><br>Randomization will be stratified by baseline Eastern Cooperative Oncology Group performance status (ECOG PS; 0 vs. 1), prior use of irinotecan (yes vs. no) and cetuximab source (US-licensed vs. EU-approved). The DMC will review the available safety information after the first 30 patients in the randomized Phase 3 portion of the study (i.e., approximately 10 patients in each arm) have had the opportunity to complete at least 1 cycle of treatment to confirm tolerability.<br><br>Treatment will be administered in 28-day cycles until disease progression, unacceptable toxicity, withdrawal of consent, initiation of subsequent anticancer therapy or death. In special circumstances (defined in Section 4.5), continuation of treatment beyond disease progression may be allowed. After treatment is discontinued, randomized Phase 3 patients will continue to be followed for OS unless consent for survival follow-up is withdrawn.<br><br>Upon implementation of protocol v8.0, patients randomized to the Control arm who meet crossover eligibility criteria may cross over to receive the triplet regimen. Crossover patients will undergo study activities and assessments similar to those performed for patients randomized to the Triplet arm and will be treated until disease progression, unacceptable toxicity, withdrawal of consent, initiation of subsequent anticancer therapy or death. In special circumstances (defined in Section 4.5), continuation of treatment beyond disease progression may be allowed. After treatment is discontinued, patients will continue to be followed for OS unless consent for survival follow-up is withdrawn.<br><br>**End of Study**<br><br>End of study will be defined as the point when all patients have the opportunity to be followed for at least 1 year after the randomization date of the last patient enrolled **and** at least 80% of patients have an OS event (or are lost to follow-up). Any patients still receiving study drugs at the end of the study will be allowed to continue at the discretion of the Investigator and as long as none of the treatment discontinuation criteria are met. After the end of the study, access to study drugs will be provided in accordance

	with local regulations and requirements.
**Treatment Regimens**	The investigational products in this study are encorafenib and binimetinib, which will be administered orally (PO) in combination with cetuximab (i.e., encorafenib + binimetinib + cetuximab [Safety Lead-in and Triplet Arm] and encorafenib + cetuximab [Doublet Arm]). The comparator combination treatment will be the Investigator's choice of either irinotecan/cetuximab or FOLFIRI/cetuximab (Control Arm).<br>Patients will receive the following per 28-day cycle:<br>**Safety Lead-in and Triplet Arm:**<br>• Encorafenib: 300 mg (4 × 75 mg oral capsule) QD PO<br>• Binimetinib: 45 mg (3 × 15 mg oral tablet) BID PO<br>• Cetuximab: 400 mg/m^2 initial dose (120-minute intravenous [IV] infusion on Cycle 1 Day 1), then 250 mg/m^2 (60-minute IV infusion) once weekly thereafter<br>**Doublet Arm:**<br>• Encorafenib: 300 mg (4 × 75 mg oral capsule) QD PO<br>• Cetuximab: 400 mg/m^2 initial dose (120-minute IV infusion on Cycle 1 Day 1), then 250 mg/m^2 (60-minute IV infusion) once weekly thereafter<br>**Control Arm:**<br>• Irinotecan: 180 mg/m^2 IV on Days 1 and 15<br>• Cetuximab: 400 mg/m^2 initial dose (120-minute IV infusion on Cycle 1 Day 1), then 250 mg/m^2 (60-minute IV infusion) once weekly thereafter<br>OR<br>• Irinotecan: 180 mg/m^2 IV on Days 1 and 15<br>• FA: 400 mg/m^2 IV on Days 1 and 15<br>• 5-FU: 400 mg/m^2 IV on Day 1 and 15, then 1200 mg/m^2/d x 2 days (total 2400 mg/m^2 over 46-48 hours) continuous infusion<br>• Cetuximab: 400 mg/m^2 initial dose (120-minute IV infusion on Cycle 1 Day 1), then 250 mg/m^2 (60-minute IV infusion) once weekly thereafter<br>For an individual patient, the dose of study drug(s) may be reduced or interrupted as appropriate based on protocol-defined treatment modifications.

	In the Phase 3 portion of the study, patients must receive their first dose of study drug within 5 days of randomization.<br>**Crossover**<br>Patients crossing over to the triplet regimen will receive study treatments as described above, although the cetuximab dose will be 250 mg/m^2 IV weekly or the last tolerated dose level the patient received in the Control Arm.
**Study Population**	Approximately 646 to 651 patients (includes 31 to 36 patients for the non-Japanese and Japanese Safety Lead-in study portions; additional patients may be included if lower dose levels are evaluated during the Safety Lead-in) with *BRAF*^{V600E} mCRC whose disease has progressed after 1 or 2 prior regimens in the metastatic setting.
**Duration of Study Participation**	Patients may enter into the Molecular Prescreening Phase of the study to determine the *BRAF* mutation status of their tumor using the central laboratory assay at any time prior to the Screening Period of the study. The study consists of a 28-day Screening Period, followed by 28-day treatment cycles until disease progression, unacceptable toxicity, withdrawal of consent, initiation of subsequent anticancer therapy or death. After discontinuation of study treatment, there will be a 30-day safety follow-up period and a survival follow-up period.
**Eligibility Criteria**	Eligibility will be determined separately for molecular tumor testing during a Prescreening Phase and for enrollment/randomization in the study during a Screening Phase.
**Inclusion and Exclusion Criteria for Molecular Prescreening**	Patients may undergo molecular tumor prescreening at any time prior to Screening as long as they meet all the following inclusion/exclusion criteria:<br>**Inclusion Criteria for Molecular Prescreening**<br>All the following inclusion criteria must be met for a patient to be eligible to undergo molecular tumor prescreen:<br>1. Provide a signed and dated prescreening informed consent document<br>2. Age ≥ 18 years at time of informed consent<br>3. Histologically- or cytologically-confirmed CRC that is metastatic<br>4. Eligible to receive cetuximab per locally approved label with regard to tumor *RAS* status

	5. Able to provide a sufficient amount of representative tumor specimen (primary or metastatic, archival or newly obtained) for central laboratory testing of *BRAF* and *KRAS* mutation status (minimum of 6 slides; optimally up to 15 slides).<br>**Exclusion Criteria for Molecular Prescreening**<br>Patients meeting any of the following criteria at Prescreening are not eligible to undergo molecular tumor prescreen:<br>1. Leptomeningeal disease<br>2. History or current evidence of retinal vein occlusion (RVO) or current risk factors for RVO (e.g., uncontrolled glaucoma or ocular hypertension, history of hyperviscosity or hypercoagulability syndromes)<br>3. Known history of acute or chronic pancreatitis<br>4. History of chronic inflammatory bowel disease or Crohn's disease requiring medical intervention (immunomodulatory or immunosuppressive medications or surgery) ≤12 months prior to randomization<br>5. Concurrent neuromuscular disorder that is associated with the potential of elevated creatine kinase (CK) (e.g., inflammatory myopathies, muscular dystrophy, amyotrophic lateral sclerosis, spinal muscular atrophy)<br>6. Known history of human immunodeficiency virus (HIV) infection<br>7. Known history of Gilbert's syndrome or is known to have any of the following genotypes: UGT1A1*6/*6, UGT1A1*28/*28, or UGT1A1*6/*28<br>8. Known contraindication to receive cetuximab or irinotecan at the planned doses; refer to the most recent cetuximab and irinotecan summary of product characteristics (SPC) or local label as applicable<br>9. Prior anti-epidermal growth factor receptor (EGFR) treatment<br>10. More than 2 prior regimens in the metastatic setting<br>Note:<br>a. Disease relapse during treatment or within 6 months following adjuvant therapy will be considered

	metastatic disease.<br>b. Maintenance therapy given in the metastatic setting will not be considered a separate regimen.
**Patient Eligibility**	**Inclusion Criteria**<br>All the following inclusion criteria must be met for a patient to be included in the study:<br>1. Provide a signed and dated Screening informed consent document<br>2. Age ≥ 18 years at time of informed consent<br>3. Histologically- or cytologically-confirmed CRC that is metastatic<br>4. Presence of *BRAF*^{V600E} in tumor tissue previously determined by a local assay at any time prior to Screening **or** by the central laboratory<br>Notes:<br>a) Only PCR and NGS-based local assays results will be acceptable.<br>b) Central testing cannot be repeated to resolve discordances with a local result once the central laboratory delivers a definitive result (positive or negative).<br>c) If the result from the central laboratory is indeterminate or the sample is deemed is inadequate for testing, additional samples may be submitted.<br>d) If at any time in the Phase 3 portion of the study there is lack of *BRAF*^{V600E} confirmation by the central laboratory (for any reason including discordance and inadequate available tissue) in 37 total patients or discordance (a valid result of "no *BRAF*^{V600E} mutation" as determined by the central laboratory) between the local assay and the central laboratory in 18 patients, all subsequent patients will be required to have *BRAF*^{V600E} determined by the central laboratory prior to enrollment (see Section 7.1.1).<br>e) Results from local laboratories with more than 1 discordant result leading to patient enrollment will not

	be accepted for further patient enrollment.<br>f) Sites with more than 2 randomized patients having indeterminate results after initiation of protocol version 6 will be required to enroll all subsequent patients based only on central laboratory assay results.<br>5. Able to provide a sufficient amount of representative tumor specimen (primary or metastatic, archival or newly obtained) for confirmatory central laboratory testing of *BRAF* and *KRAS* mutation status (minimum of 6 slides; optimally up to 15 slides)<br>Note: Tumor samples must be submitted to the central laboratory for *BRAF* testing as soon as possible following the signing of the Molecular Prescreening informed consent. The *BRAF* status must be confirmed no later than 30 days following first dose of study drug.<br>6. Eligible to receive cetuximab per locally approved label with regard to tumor *RAS* status<br>7. Progression of disease after 1 or 2 prior regimens in the metastatic setting<br>Notes:<br>a. Disease relapse during treatment or within 6 months following adjuvant therapy will be considered metastatic disease.<br>b. Patients who have received 2 prior regimens (i.e. those entering the study in the 3rd line setting), must have received or have been offered and refused prior oxaliplatin unless it was contraindicated due to underlying conditions.<br>c. Maintenance therapy given in the metastatic setting will not be considered a separate regimen.<br>d. In the Phase 3 portion of study, the number of patients having received 2 prior regimens will be limited to 215 (35% of the total randomized). Patients with 2 prior regimens who have entered Screening at the time that the limit has been reached will be permitted to continue into the study if they are otherwise determined to be eligible.<br>8. Evidence of measurable or evaluable non-measurable

	disease per RECIST, v1.1<br>9. ECOG PS of 0 or 1<br>10. Adequate bone marrow function characterized by the following at screening:<br>• Absolute neutrophil count (ANC) ≥ 1.5 × 10^9/L;<br>• Platelets ≥ 100 × 10^9/L;<br>• Hemoglobin ≥ 9.0 g/dL<br>Note: Transfusions will be allowed to achieve this. Transfusions will be permitted provided the patient has not received more than 2 units red blood cells in the prior 4 weeks to achieve this criteria.<br>11. Adequate renal function characterized by serum creatinine ≤ 1.5 × upper limit of normal (ULN), or calculated by Cockroft-Gault formula or directly measured creatinine clearance ≥ 50 mL/min at screening<br>12. Adequate electrolytes at Baseline, defined as serum potassium and magnesium levels within institutional normal limits (Note: replacement treatment to achieve adequate electrolytes will be allowed)<br>13. Adequate hepatic function characterized by the following at screening:<br>• Serum total bilirubin ≤ 1.5 × ULN and < 2 mg/dL<br>Note: Patients who have a total bilirubin level > 1.5 × ULN will be allowed if their indirect bilirubin level is ≤ 1.5 × ULN<br>• Alanine aminotransferase (ALT) and/or aspartate aminotransferase (AST) ≤ 2.5 × ULN, or ≤ 5 × ULN in presence of liver metastases<br>14. Adequate cardiac function characterized by the following at screening:<br>• Left ventricular ejection fraction (LVEF) ≥ 50% as determined by a MUGA scan or ECHO;<br>• Mean triplicate QT interval corrected for heart rate using Fridericia's formula (QTcF) value ≤480 msec<br>15. Able to take oral medications

	16. Willing and able to comply with scheduled visits, treatment plan, laboratory tests and other study procedures<br>17. Female patients are either postmenopausal for at least 1 year, are surgically sterile for at least 6 weeks, or must agree to take appropriate precautions to avoid pregnancy from screening through follow-up if of childbearing potential<br>Note: Permitted contraceptive methods listed in Section 5.3.1 should be communicated to the patients and their understanding confirmed. For all females, the pregnancy test result must be negative at screening.<br>18. Males must agree to take appropriate precautions to avoid fathering a child from screening through 90 days following end of therapy.<br>Note: Permitted contraceptive methods listed in Section 5.3.1 should be communicated to the patients and their understanding confirmed.<br>19. Patients under guardianship or partial guardianship will be eligible unless prohibited by local laws or by local/central ethic committees (e.g., France, Germany). Where allowed, all procedures prescribed by law must be followed.<br>**Exclusion Criteria**<br>Patients meeting any of the following criteria will not be included in the study:<br>1. Prior treatment with any RAF inhibitor, MEK inhibitor, cetuximab, panitumumab or other EGFR inhibitors<br>2. Prior irinotecan hypersensitivity or toxicity that would suggest an inability to tolerate irinotecan 180 mg/m^2 every 2 weeks<br>3. Symptomatic brain metastasis<br>Notes: Patients previously treated or untreated for this condition who are asymptomatic in the absence of corticosteroid and anti-epileptic therapy are allowed. Brain metastases must be stable for ≥ 4 weeks, with imaging (e.g., magnetic resonance imaging [MRI] or computed tomography [CT]) demonstrating no current evidence of progressive brain metastases at screening.<br>4. Leptomeningeal disease

	5. History or current evidence of RVO or current risk factors for RVO (e.g., uncontrolled glaucoma or ocular hypertension, history of hyperviscosity or hypercoagulability syndromes) at the time of screening<br>6. Use of any herbal medications/supplements or any medications or foods that are strong inhibitors or inducers of cytochrome P450 (CYP) 3A4/5 ≤ 1 week prior to the start of study treatment<br>7. Known history of acute or chronic pancreatitis<br>8. History of chronic inflammatory bowel disease or Crohn's disease requiring medical intervention (immunomodulatory or immunosuppressive medications or surgery) ≤ 12 months prior to randomization<br>9. Impaired cardiovascular function or clinically significant cardiovascular diseases, including any of the following:<br>a. History of acute myocardial infarction, acute coronary syndromes (including unstable angina, coronary artery bypass graft [CABG], coronary angioplasty or stenting) ≤ 6 months prior to start of study treatment;<br>b. Symptomatic congestive heart failure (i.e., Grade 2 or higher), history or current evidence of clinically significant cardiac arrhythmia and/or conduction abnormality ≤ 6 months prior to start of study treatment, except atrial fibrillation and paroxysmal supraventricular tachycardia<br>10. Uncontrolled hypertension defined as persistent systolic blood pressure ≥ 150 mmHg or diastolic blood pressure ≥ 100 mmHg despite current therapy<br>11. Impaired hepatic function, defined as Child-Pugh class B or C<br>12. Impaired gastrointestinal (GI) function or disease that may significantly alter the absorption of encorafenib or binimetinib (e.g., ulcerative diseases, uncontrolled vomiting, malabsorption syndrome, small bowel resection with decreased intestinal absorption)<br>13. Concurrent or previous other malignancy within 5 years of

	study entry, except cured basal or squamous cell skin cancer, superficial bladder cancer, prostate intraepithelial neoplasm, carcinoma in-situ of the cervix, or other noninvasive or indolent malignancy without Sponsor approval<br>14. History of thromboembolic or cerebrovascular events ≤ 6 months prior to starting study treatment, including transient ischemic attacks, cerebrovascular accidents, deep vein thrombosis or pulmonary emboli<br>15. Concurrent neuromuscular disorder that is associated with the potential of elevated CK (e.g., inflammatory myopathies, muscular dystrophy, amyotrophic lateral sclerosis, spinal muscular atrophy)<br>16. Treatment with any of the following:<br>• Cyclical chemotherapy within a period of time that was shorter than the cycle length used for that treatment (e.g., 6 weeks for nitrosourea, mitomycin-C) prior to starting study treatment<br>• Biologic therapy (e.g., antibodies) except bevacizumab or aflibercept, continuous or intermittent small molecule therapeutics, or any other investigational agents within a period of time that is ≤ 5 half-lives ($t_{1/2}$) or ≤ 4 weeks (whichever is shorter) prior to starting study treatment<br>• Bevacizumab or aflibercept therapy ≤ 3 weeks prior to starting study treatment<br>• Radiation therapy that included > 30% of the bone marrow<br>17. Residual CTCAE ≥ Grade 2 toxicity from any prior anticancer therapy, with the exception of Grade 2 alopecia or Grade 2 neuropathy<br>18. Known history of HIV infection<br>19. Active hepatitis B or hepatitis C infection<br>20. Known history of Gilbert's syndrome or is known to have any of the following genotypes: UGT1A1*6/*6, UGT1A1*28/*28, or UGT1A1*6/*28.<br>21. Known contraindication to receive cetuximab or irinotecan

	at the planned doses; refer to the most recent cetuximab and irinotecan SPC or local label as applicable.<br>22. Current treatment with a non-topical medication known to be a strong inhibitor of CYP3A4. However, patients who either discontinue this treatment or switch to another medication at least 7 days prior to starting study treatment are eligible.<br>23. Concomitant use of St. John's Wort (hypericum perforatum).<br>24. Other severe, acute or chronic medical or psychiatric condition or laboratory abnormality that may increase the risk associated with study participation or study drug administration or that may interfere with the interpretation of study results and, in the judgment of the Investigator, would make the patient an inappropriate candidate for the study.<br>25. Pregnant, confirmed by a positive human chorionic gonadotropin (hCG) laboratory test result, or nursing (lactating).<br>26. Prior enrollment into this clinical study.
**Inclusion and Exclusion Criteria for Treatment Crossover**	**Inclusion Criteria for Treatment Crossover**<br>All of the following inclusion criteria must be met within 28 days prior to initiation of crossover treatment.<br>1. Patient was randomized to the Control arm and:<br>• Discontinued Control arm treatment for any reason on or after 13 June 2019 (i.e., 4 weeks before the date of protocol version 8.0),<br>• Received no systemic anticancer therapy after discontinuation of Control arm treatment, and<br>• Remains in survival follow-up.<br>2. Provide a signed and dated Crossover informed consent document.<br>3. ECOG PS of 0 or 1.<br>4. Adequate bone marrow function characterized by the following:

	• Absolute neutrophil count (ANC) ≥ 1.5 × 10^9/L;<br>• Platelets ≥ 100 × 10^9/L;<br>• Hemoglobin ≥ 9.0 g/dL<br>Note: Transfusions will be allowed to achieve this. Transfusions will be permitted provided the patient has not received more than 2 units red blood cells in the prior 4 weeks to achieve these criteria.<br>5. Adequate renal function characterized by serum creatinine ≤ 1.5 × upper limit of normal (ULN), or calculated by Cockroft-Gault formula or directly measured creatinine clearance ≥ 50 mL/min.<br>6. Adequate electrolytes defined as serum potassium and magnesium levels within institutional normal limits (Note: replacement treatment to achieve adequate electrolytes will be allowed).<br>7. Adequate hepatic function characterized by the following:<br>• Serum total bilirubin ≤ 1.5 × ULN and < 2 mg/dL<br>Note: Patients who have a total bilirubin level > 1.5 × ULN will be allowed if their indirect bilirubin level is ≤ 1.5 × ULN<br>• Alanine aminotransferase (ALT) and/or aspartate aminotransferase (AST) ≤ 2.5 × ULN, or ≤ 5 × ULN in presence of liver metastases.<br>8. Adequate cardiac function characterized by the following:<br>• Mean triplicate QT interval corrected for heart rate using Fridericia's formula (QTcF) value ≤480 msec.<br>9. Able to take oral medications.<br>10. Willing and able to comply with scheduled visits, treatment plan, laboratory tests and other study procedures.<br>11. Female patients are either postmenopausal for at least 1 year, are surgically sterile for at least 6 weeks, or must agree to take appropriate precautions to avoid pregnancy through follow-up if of childbearing potential.<br>Note: Permitted contraceptive methods listed in Section 5.3.1 should be communicated to the patients and their understanding confirmed. For all females, the pregnancy test result must be negative at crossover screening and at

	the time of crossover.<br>12. Males must agree to take appropriate precautions to avoid fathering a child through 90 days following end of therapy. Note: Permitted contraceptive methods listed in Section 5.3.1 should be communicated to the patients and their understanding confirmed.<br>13. Patients under guardianship or partial guardianship will be eligible unless prohibited by local laws or by local/central ethic committees (e.g., France, Germany). Where allowed, all procedures prescribed by law must be followed.<br>**Exclusion Criteria for Treatment Crossover**<br>Patients randomized to the Control arm who meet any of the following criteria within 28 days prior to initiation of crossover treatment will not be eligible for crossover.<br>1. Patients for whom cetuximab was permanently discontinued due to cetuximab-related toxicity in the randomized Phase 3 portion of the study.<br>2. Symptomatic brain metastasis.<br>Notes: Patients previously treated or untreated for this condition who are asymptomatic in the absence of corticosteroid and anti-epileptic therapy are allowed. Brain metastases must be stable for ≥ 4 weeks, with imaging (e.g., MRI or CT) demonstrating no current evidence of progressive brain metastases.<br>3. Leptomeningeal disease.<br>4. Use of any herbal medications/supplements or any medications or foods that are strong inhibitors or inducers of cytochrome P450 (CYP) 3A4/5 ≤ 1 week prior to crossover.<br>5. Known history of acute pancreatitis.<br>6. History of chronic inflammatory bowel disease or Crohn's disease requiring medical intervention (immunomodulatory or immunosuppressive medications or surgery) ≤ 12 months prior to crossover.<br>7. Impaired cardiovascular function or clinically significant cardiovascular diseases, including any of the following:<br>• History of acute myocardial infarction, acute

	coronary syndromes (including unstable angina, CABG, coronary angioplasty or stenting) ≤ 6 months prior to start of crossover;<br>• Symptomatic congestive heart failure (i.e., Grade 2 or higher), history or current evidence of clinically significant cardiac arrhythmia and/or conduction abnormality ≤ 6 months prior to crossover, except atrial fibrillation and paroxysmal supraventricular tachycardia.<br>8. Impaired hepatic function, defined as Child Pugh class B or C.<br>9. Impaired gastrointestinal (GI) function or disease that may significantly alter the absorption of encorafenib or binimetinib (e.g., ulcerative diseases, uncontrolled vomiting, malabsorption syndrome, small bowel resection with decreased intestinal absorption).<br>10. Treatment with irinotecan within 3 weeks prior to Day 1 of crossover treatment.<br>11. Residual CTCAE ≥ Grade 2 toxicity from any prior anticancer therapy, with the exception of Grade 2 alopecia or Grade 2 neuropathy.<br>12. Concomitant use of St. John's Wort (hypericum perforatum).<br>13. Other severe, acute or chronic medical or psychiatric condition or laboratory abnormality that may increase the risk associated with study participation or study drug administration or that may interfere with the interpretation of study results and, in the judgment of the Investigator, would make the patient an inappropriate candidate for crossover treatment.<br>14. Pregnant, confirmed by a positive hCG laboratory test result, or nursing (lactating) at time of crossover.<br>15. Left ventricular ejection fraction (LVEF) < 50% as determined by a MUGA scan or ECHO.<br>16. Current evidence or history of RVO or current risk factors for RVO (e.g., uncontrolled glaucoma or ocular hypertension, history of hyperviscosity or

	hypercoagulability syndromes).<br>17. Uncontrolled hypertension defined as persistent systolic blood pressure ≥ 150 mmHg or diastolic blood pressure ≥ 100 mmHg despite current therapy.<br>18. History of thromboembolic or cerebrovascular events ≤ 6 months prior to crossover, including transient ischemic attacks, cerebrovascular accidents, deep vein thrombosis or pulmonary emboli.<br>19. Concurrent neuromuscular disorder that is associated with the potential of elevated CK (e.g., inflammatory myopathies, muscular dystrophy, amyotrophic lateral sclerosis, spinal muscular atrophy)
**Assessments**	Efficacy assessments include radiological imaging (e.g., CT, MRI, X-ray, different methods of whole body bone scans) and with tumor response will be determined locally by the Investigator and restrospectively by BICR according to RECIST, v1.1. Tumor assessments will be performed every 6 weeks from randomization (or from first dose in Safety Lead-in) for the first 24 weeks, then every 12 weeks until disease progression, withdrawal of consent for treatment, initiation of subsequent anticancer therapy, patient is lost to follow-up, or death. After patients have discontinued treatment for any reason, they will be followed every 3 months, or more frequently as needed, until withdrawal of consent, the patient is lost to follow-up, death or end of study as defined in Section 4.6. If patients withdraw consent, they will be asked if they are willing to be contacted via telephone for survival status. If the patient refuses to be contacted, attempts to determine survival status should be made via access to public records where permitted by local laws.<br><br>Safety assessments include monitoring of AEs, clinical laboratory tests (chemistry, hematology, coagulation and urinalysis), vital signs, physical examinations, ophthalmic examinations, dermatologic examinations, ECGs and ECHO/MUGA scans. Other assessments include ECOG PS and QoL questionnaires (EORTC QLQ-C30, FACT-C, EQ5D-5L and PGIC).<br><br>Serial blood samples for characterization of the PK of encorafenib, cetuximab, binimetinib and the active metabolite of binimetinib (AR00426032) will be collected from all patients in the Safety Lead-in and from a subset of patients (approximately the first 50 enrolled in the Triplet Arm and in the Doublet Arm from any site and approximately the first 100 enrolled in the Control Arm from

	any site).<br><br>Exploratory assessments include measurement of tumor markers (CEA and CA19-9), determination of microsatellite instability (MSI) status and investigation of potential biomarkers in blood and tumor tissue (as available). Tumor samples (fresh or archival) used to determine eligibility will also be used in the development of a diagnostic assay for *BRAF*^{V600E} CRC (i.e., companion diagnostic assay); blood samples collected at baseline may also be used for this purpose. The *BRAF*^{V600E} and *KRAS*^{wt} status of all patients' tumors will be confirmed centrally.
**Statistics**	The sample size of the randomized Phase 3 portion of the study (approximately 615 patients) is driven by the key secondary endpoint of the OS of the Doublet Arm vs. Control Arm. For this comparison, 338 OS events are required to have approximately 90% power to detect an OS hazard ratio (HR) of 0.70 with 1-sided alpha = 0.025. This corresponds to a median OS of 7.1 months in the Doublet Arm and 5 months in the Control Arm. In order to observe 338 OS events in the 2 arms, a total of 615 patients will be randomized in a 1:1:1 ratio to the Triplet Arm, the Doublet Arm or the Control Arm (i.e., approximately 205 patients per arm). This assumes the accrual rate will reach a maximum of 25 patients per month and approximately 5% will be lost to follow-up. It is anticipated that it will take approximately 25 months to enroll 615 patients.<br><br>The final OS analysis will occur once at least 268 events are observed in the Triplet Arm + Control Arm and at least 338 events are observed in the Doublet Arm + Control Arm. This is expected to occur approximately 33 months after the first patient is randomized. At the time of the final analysis, it is anticipated that approximately 333 OS events will be observed in the Triplet Arm + Control Arm, but only the first 268 OS events will be used for the final analysis. With 268 events, there will be approximately 90% power to detect a HR of 0.67 (5 versus 7.5 months) with 1-sided alpha = 0.025. The null hypothesis that the OS in the Triplet Arm is the same or worse than the Control Arm will be rejected if the HR is smaller (i.e. better) than 0.79.<br><br>An initial analysis of the study will be performed when all three of the following criteria have been met:<br><br>• approximately 9 months after randomization of the 330th patient (i.e., approximately 110 patients per arm), to allow a majority of responders among the 330 Phase 3 patients to have the opportunity to be followed for approximately 6 months or longer after their first response

	• at least 188 OS events have occurred in the Triplet and Control arms combined (i.e., approximately 70% information)<br>• at least 169 OS events have occurred in the Doublet and Control arms combined (i.e., approximately 50% information)<br><br>This analysis will be conducted by the DMC and will serve as both the primary analysis of Triplet vs Control ORR and an interim analysis of Triplet vs Control OS. The Type I error rate for the primary endpoints will be controlled using a fallback procedure described by Wiens and Dmitrienko (2005). A 1-sided alpha value of 0.005 will be assigned to the Triplet vs Control ORR endpoint. The remaining alpha of 0.020 will be assigned to the Triplet vs Control OS endpoint.<br><br>The primary endpoint of Triplet vs Control ORR (per BICR) will be formally tested first and will be based on the first 330 randomized patients. A sample size of 110 patients per arm provides 88% power to detect a 20% absolute difference in ORR, assuming an ORR of 10% in the Control Arm and an ORR of 30% in the Triplet Arm at a 1-sided alpha of 0.005.<br><br>If the Triplet vs. Control ORR comparison is positive ($p < 0.005$), then, based on the fallback procedure, the OS endpoint of Triplet vs. Control will be assigned a total 1-sided alpha = 0.025. Otherwise, the Triplet vs. Control OS will be assigned a total 1-sided alpha = 0.020.<br><br>An interim analysis for superiority or (non-binding) futility of the Triplet vs Control OS endpoint will then also be performed using data from all available patients. Futility and superiority boundaries for both the OS interim and final analyses will be determined using a Lan-DeMets spending function that approximates O'Brien-Fleming stopping boundaries.<br><br>If the p-value for the Triplet Arm vs Control Arm OS comparison exceeds the superiority boundary at the interim analysis, the following endpoints will be tested at that time in the following order:<br>1. OS of Doublet Arm vs. Control Arm<br>2. ORR (per BICR) of Doublet Arm vs. Control Arm<br>3. PFS (per BICR) of Triplet Arm vs. Control Arm<br>4. PFS (per BICR) of Doublet Arm vs. Control Arm<br><br>For each endpoint in the hierarchy, a Lan-DeMets spending

	function that approximates the O'Brien-Fleming stopping boundaries will be used with data from all available patients. The total alpha assigned to each endpoint will match the total alpha assigned to Triplet vs Control OS comparison (either 1-sided 0.025 or 1-sided 0.020). If any of the above tests is found to not be statistically significant, all subsequent comparisons will only be summarized using descriptive statistics.<br><br>If the p-value for the Triplet Arm vs Control Arm OS comparison does not exceed the superiority boundary at the OS interim analysis, OS of Triplet Arm vs. Control Arm will be tested again at the final analysis (i.e., when at least 268 OS events in Triplet and Control and at least 338 OS events in Doublet and Control have occurred). If the results are positive, the four remaining endpoints will be tested using the hierarchy and method specified above. If the results of the OS of Triplet Arm vs. Control Arm comparison are negative, no further formal testing will be conducted and all remaining endpoints will be summarized using descriptive statistics.<br><br>The OS of Triplet Arm vs. Doublet Arm will be assessed as a secondary endpoint, but this will not be included in the hierarchical sequence that controls the overall Type I error rate. If the interim analysis for OS of Triplet vs. Control exceeds the superiority boundary, patients in the Triplet and Doublet arms may continue to be followed for a more mature comparison.<br><br>Additional analyses will be performed for PFS, ORR, DOR and time to response (CR or PR).<br><br>Plasma concentrations of encorafenib, binimetinib and the active metabolite of binimetinib (AR00426032) and serum concentrations of cetuximab will be determined using validated assays. Descriptive statistics of concentrations will be reported and summarized graphically.<br><br>Pharmacokinetic parameters from the Safety Lead-in will be generated by noncompartmental and/or compartmental approaches as appropriate. No noncompartmental PK parameters will be estimated in the Phase 3 portion of the study due to sparse sampling. Phase 3 PK parameters will be estimated for encorafenib, cetuximab, binimetinib and the active metabolite of binimetinib (AR00426032) as appropriate using a population model-based approach for patients in the Safety Lead-in, Triplet, Doublet, and Control Arms, as appropriate, to determine appropriate model-based PK parameters and their respective variability, if deemed appropriate.<br><br>Assessments of potential drug-drug interactions for encorafenib,

	cetuximab, binimetinib and the active metabolite of binimetinib (AR00426032) will be conducted by comparison to historical PK results for all 3 study drugs and by covariate modeling as part of the population PK analysis, as appropriate.<br><br>Safety data will be presented in tabular and/or graphical format and summarized descriptively by treatment arm and study day, as appropriate.<br><br>Results of QoL questionnaires (EORTC QLQ-C30, FACT-C, EQ-5D-5L, PGIC) will be summarized for each visit using descriptive statistics. Additionally, changes from baseline in the domain scores at the time of each assessment will be summarized. A repeated measurement analysis model may be used to compare the treatment groups with respect to longitudinal changes over time. Additional details regarding analysis of QoL results will be included in the statistical analysis plan.
**Sponsor**	Array BioPharma Inc.

TABLE OF CONTENTS

1.0 INVESTIGATORS AND STUDY ADMINISTRATIVE STRUCTURE 43

2.0 INTRODUCTION...44

2.1 Metastatic Colorectal Cancer...44

2.2 *BRAF*^{V600E} Colorectal Cancer...44

2.2.1 Inhibition of BRAF, MEK, and EGFR in *BRAF*-V600E Colorectal Cancer Cells 45

2.2.2 Effects of Encorafenib and Cetuximab in *BRAF*-V600E Colorectal Cancer..........46

2.2.3 Effects of Encorafenib, Binimetinib, and Cetuximab in *BRAF*-V600E Colorectal Cancer 47

2.2.4 Encorafenib in Colorectal Cancer 48

2.2.5 Other BRAF Inhibitors 51

2.3 Overview of Encorafenib 52

2.3.1 Nonclinical Safety Pharmacology and Toxicology 52

2.3.2 Pharmacokinetics and Metabolism...53

2.3.3 Clinical Safety 54

2.4 Overview of Binimetinib...58

2.4.1 Nonclinical Safety Pharmacology and Toxicology 58

2.4.2 Clinical Pharmacokinetics...58

2.4.3 Clinical Experience...59

2.5 Overview of the Combination of Encorafenib and Binimetinib..................................60

2.5.1 Nonclinical Pharmacokinetics and Metabolism...60

2.5.2 Clinical Experience...61

2.6 Overview of Cetuximab 63

2.6.1 Clinical Experience...63

2.7 Overview of Irinotecan...64

2.8 Overview of FOLFIRI...64

2.9 Rationale for the Study...64

2.9.1 Study Design 65

2.9.2 Safety Lead-in 67

2.9.3 Dose Selection...68

2.9.4 Summary 69

3.0 STUDY OBJECTIVES AND ENDPOINTS...71

3.1 Study Objectives...71

3.1.1 Safety Lead-In...71

3.1.2 Randomized Phase 3...71

3.2 Study Endpoints...73

3.2.1 Safety Lead-In...73

3.2.2 Randomized Phase 3...74

4.0 STUDY DESIGN 76
4.1 Study Design Overview 76
4.2 Safety Lead-in Phase..80
4.2.1 Dose-Limiting Toxicities 80
4.2.2 Tolerability Assessment 81
4.2.3 Japanese Safety Lead-in...83
4.3 Randomized Phase 3 84
4.4 Measures to Minimize Bias 85
4.5 Duration of Treatment 86
4.6 End of Study 87
4.7 Data Review Committees 87
4.7.1 Data Monitoring Committee 87
4.7.2 Steering Committee 87

5.0 PATIENT POPULATION 88
5.1 Number of Patients 88
5.2 Selection of Patients..88
5.2.1 Eligibility Criteria for Molecular Prescreening...88
5.2.2 Patient Eligibility...90
5.2.3 Eligibility Criteria for Treatment Crossover 95
5.3 Lifestyle Guidelines 98
5.3.1 Contraception 98
5.3.2 Photosensitivity 99

6.0 STUDY TREATMENT AND CONCOMITANT MEDICATIONS/THERAPIES 100
6.1 Patient Numbering 100
6.2 Allocation to Treatment...100
6.2.1 Treatment Crossover...100
6.3 Doses and Schedule of Administration 101
6.3.1 Administration of Encorafenib or Encorafenib + Binimetinib 102
6.3.2 Administration of Cetuximab...103
6.3.3 Administration of Irinotecan 104
6.3.4 Administration of FOLFIRI 104
6.4 Dose Modifications..104
6.4.1 Dose Modifications for Encorafenib and/or Binimetinib 106
6.4.2 Dose Modifications for Cetuximab 117
6.4.3 Dose Modifications for FOLFIRI Treatment 118
6.4.4 Dose Modifications for Irinotecan When Given Without 5-FU and FA...............121
6.5 Concomitant Medications/Therapies 122
6.5.1 Permitted Concomitant Medications/Therapies 122
6.5.2 Permitted Concomitant Therapy Requiring Caution and/or Action 123

6.5.3 Prohibited Concomitant Therapy 125
6.6 Study Drug Supply..125
6.6.1 Manufacturing, Formulation, Packaging and Labeling 125
6.6.2 Shipping, Storage and Handling..126
6.6.3 Accountability and Return of Study Drug Supply 127
6.7 Treatment Compliance 127
7.0 STUDY PROCEDURES AND ASSESSMENTS..128
7.1 Prescreening/Screening Assessments and Procedures 128
7.1.1 *BRAF* Testing 128
7.1.2 Molecular Prescreening 129
7.1.3 Screening..130
7.1.4 Information Collected for Screen Failures..130
7.1.5 Patient Demographics and Other Baseline Characteristics..130
7.2 Safety Assessments 131
7.2.1 Adverse Events..131
7.2.2 Clinical Laboratory Tests 131
7.2.3 Vital Signs 134
7.2.4 Physical Examination 134
7.2.5 Ophthalmic Assessments 135
7.2.6 Dermatologic Evaluations..135
7.2.7 Electrocardiograms..136
7.2.8 Echocardiogram/Multi-gated Acquisition Scans..136
7.2.9 ECOG Performance Status..136
7.3 Pharmacokinetic and Biomarker Blood Sampling Assessments 137
7.4 Pharmacokinetics Assessment 140
7.5 Biomarker Assessments..142
7.5.1 C-reactive Protein..142
7.5.2 Tumor Markers (CEA and CA19-9)..142
7.5.3 Other Assessment (Companion Diagnostic Assay) 142
7.5.4 Microsatellite Instability..142
7.5.5 Predictive Biomarkers of Activity..142
7.5.6 *RAS* Wild-Type Status..143
7.5.7 Other Exploratory Assessments..143
7.5.8 Retention of Samples for Future Analysis 144
7.6 Efficacy Assessments 144
7.6.1 BICR Evaluation of Imaging Data 146
7.6.2 Patient-reported Outcome Assessments..146
8.0 SCHEDULE OF PROCEDURES AND ASSESSMENTS 148
8.1 Molecular Prescreening..160

8.2 Screening ... 160
8.2.1 Screening for Treatment Crossover ... 162
8.3 Cycle 1 ... 162
8.3.1 Cycle 1 Day 1 ... 162
8.3.2 Cycle 1 Day 8 ... 164
8.3.3 Cycle 1 Day 15 ... 165
8.3.4 Cycle 1 Day 22 ... 165
8.4 Subsequent Cycles ... 166
8.4.1 Subsequent Cycles Day 1 ... 166
8.4.2 Subsequent Cycles Day 8 ... 168
8.4.3 Subsequent Cycles Day 15 ... 168
8.4.4 Subsequent Cycles Day 22 ... 168
8.5 End of Treatment Visit ... 168
8.6 Follow-up Visits ... 170
8.6.1 30-Day Safety Follow-up Visit ... 170
8.6.2 Other Follow-up Visits ... 171
8.7 Collection of Data for Ongoing Patients Following Database Lock ... 172

9.0 STUDY DISCONTINUATION ... 173
9.1 Termination of the Study by the Sponsor ... 173
9.2 Treatment Discontinuation for Individual Patients ... 173
9.3 Study Discontinuation for Individual Patients ... 174
9.4 Replacement of Patients ... 175

10.0 SAFETY MONITORING: DEFINITIONS AND REPORTING ... 176
10.1 Adverse Event ... 176
10.2 Events Related to Progression of Disease ... 176
10.3 Adverse Events Related to Subsequent Anticancer Therapy ... 176
10.4 Clinical Laboratory Abnormalities ... 176
10.5 Overdose ... 176
10.6 Assessment of Severity ... 176
10.7 Assessment of Causality ... 177
10.8 Assessment of Seriousness ... 178
10.9 Reporting of Serious and Nonserious Adverse Events ... 178
10.10 Reporting of Suspected Unexpected Serious Adverse Events (SUSAR) ... 179
10.11 Pregnancy or Drug Exposure during Pregnancy ... 179
10.12 Review of Safety Data ... 180

11.0 STATISTICAL METHODS ... 181
11.1 Determination of Sample Size ... 181
11.1.1 Safety Lead-in ... 181
11.1.2 Randomized Phase 3 ... 182

11.2 Analysis Sets......184
11.2.1 Full Analysis Set......184
11.2.2 Safety Set......184
11.2.3 Dose-Determining Set......184
11.2.4 Safety Lead-in Efficacy Set......184
11.2.5 Phase 3 Response Efficacy Set......184
11.2.6 Per Protocol Set......185
11.2.7 Pharmacokinetic Set......185
11.3 Statistical Analyses and Methods......185
11.3.1 General Considerations......185
11.3.2 Patient Characteristics......185
11.3.3 Efficacy Analyses......185
11.3.4 Pharmacokinetic Analysis......191
11.3.5 Exploratory Analyses......192
11.3.6 Subgroup Analyses......192
11.3.7 Safety Analyses......192
11.3.8 Interim Safety Reviews......196
11.3.9 Initial Efficacy Analyses......196
12.0 DATA RECORDING, RETENTION AND MONITORING......198
12.1 Data Management......198
12.2 Data Monitoring......198
12.3 Quality Control and Quality Assurance......199
13.0 REGULATORY, ETHICAL AND LEGAL OBLIGATIONS......200
13.1 Good Clinical Practice......200
13.2 Ethics Committee Approval......200
13.3 Regulatory Authority Approval......200
13.4 Other Required Approvals......200
13.5 Informed Consent......201
13.6 Patient Confidentiality......201
13.7 Disclosure of Information......201
13.8 Publication of Study Data......202
14.0 ADHERENCE TO THE PROTOCOL......203
14.1 Amendments to the Protocol......203
15.0 REFERENCES......204

LIST OF TABLES

Table 1: Summary of Best Overall Response by Treatment Group for Patients with Metastatic Colorectal Cancer in CLGX818X2101 Dose Expansion......................48
Table 2: Summary of Best Overall Response per Investigator Assessment by Treatment Group in Phase 1b (Study CLGX818X2103-Dual Combination)..........................49
Table 3: Summary of Best Overall Response per Investigator Assessment by Treatment Group in Phase 2 (Study CLGX818X2103) 51
Table 4: Adverse Events (≥ 20%), Regardless of Study Drug Relationship, by Preferred Term - Study CLGX818X2101 in mCRC Patients in the Dose-Expansion Phase...55
Table 5: Adverse Events (≥ 20% of Patients) Regardless of Causality by Preferred Term in Phase 1b (Study CLGX818X2103, Safety Set - Dual Combination Treatment) .56
Table 6: Adverse Events Regardless of Causality in ≥ 15% of Patients by Preferred Term in the Phase 2 Interim Analysis (Study CLGX818X2103, Safety Set) 57
Table 7: Adverse Events Regardless of Causality in ≥ 10% of Patients by Preferred Term in Interim Phase 2 Analysis (Study CLGX818X2109)...62
Table 8: Criteria for Defining Dose-limiting Toxicities 80
Table 9: Starting Dose and Treatment Schedule 101
Table 10: Dose Levels for Dose Modification...106
Table 11: Modified NCI CTCAE, Version 4.03 Grading of Eye Disorders.........................107
Table 12: NCI CTCAE, Version 4.03 Grading of Serous Detachment of the Retina 108
Table 13: NCI CTCAE, Version 4.03 Grading of Uveitis..108
Table 14: NCI CTCAE, Version 4.03 Grading of Hand-foot Skin Reaction (HFSR)[a] 108
Table 15: Modified NCI CTCAE, Version 4.03 Grading of Diarrhea 108
Table 16: Recommended Dose Modifications for Encorafenib-related and/or Binimetinib-related Adverse Events...110
Table 17: Recommended Dose Modifications for Cetuximab-related Adverse Events.........117
Table 18: Recommended Dose Modifications for 5-FU and Irinotecan Treatment-related Adverse Events During FOLFIRI Treatment 118
Table 19: Recommended Dose Modifications for Irinotecan Treatment-related Adverse Events When Given Without 5-FU and FA..121
Table 20: Summary of Clinical Laboratory Tests 132
Table 21: Eastern Cooperative Oncology Group (ECOG) Performance Status Scale...........137
Table 22: Blood Collection Volumes for Pharmacokinetic and Biomarker Assessments......138
Table 23: Pharmacokinetic Sampling Times for the Safety Lead-in Portion of Study ARRAY-818-302 for the Safety Lead-in Patients Who Will Receive Encorafenib, Cetuximab and Binimetinib...139
Table 24: Pharmacokinetic Sampling Times for the Randomized Phase 3 Portion of Study ARRAY-818-302 for a Subset of the Triplet, Doublet, and Control Arms............140
Table 25: Schedule of Events for Prescreening and Screening 149
Table 26: Schedule of Events for Safety Lead-in, Triplet Arm, and Crossover Patients (Encorafenib + Binimetinib + Cetuximab) 151

Table 27: Schedule of Events for Doublet Arm (Encorafenib + Cetuximab)........................154
Table 28: Schedule of Events for Control Arm (Irinotecan/Cetuximab or FOLFIRI/Cetuximab) 157
Table 29: Operating Characteristics of Safety Lead-In Criteria for 9 Patients Compared to 3+3 Rules 182
Table 30: Expected Number of OS Events and Cumulative Power at Expected OS Analysis Timepoints for the Triplet vs Control Comparison 197

LIST OF FIGURES

Figure 1: Efficacy of Encorafenib and Cetuximab as Single Agents and in Combination in the HT-29 (*BRAF*^{V600E}, PIK3CαP449T) Human CRC Tumor Xenograft Model Grown in Immunocompromised Mice..46
Figure 2: Effects of Encorafenib and Binimetinib in Combination with Cetuximab in the HT-29 (BRAFV600E, PIK3CαP449T) Human CRC Tumor Xenograft Model Grown in Immunocompromised Mice..47
Figure 3: Best Percentage Change from Baseline in Sum of Diameters in Phase 1b (Study CLGX818X2103-Dual Combination) 50
Figure 4: Summary of Time on Study, by Response in Phase 1b (Study CLGX818X2103-Dual Combination) 50
Figure 5: Study Design 79
Figure 6: Safety Lead-in Schema 83
Figure 7: BRAF Testing..129
Figure 8: Graphic Representation of Pharmacokinetic Sampling Plan 141
Figure 9: Testing Strategy for Study ARRAY-818-302...187

LIST OF APPENDICES

Appendix 1: Recommended Guidelines for the Management of Cetuximab-induced, Encorafenib-induced and/or Binimetinib-induced Skin Toxicity.......................... 211
Appendix 2: Recommended Guidelines for the Management of Encorafenib-induced Hand-foot skin reactions (HFSR)... 214
Appendix 3: Recommended Guidelines for the Management of Binimetinib-induced Diarrhea.. 217
Appendix 4: Snellen Equivalence (Visual Acuity Conversion Chart)...................................... 220
Appendix 5: List of Concomitant Medications 221
Appendix 6: Response Evaluation Criteria in Solid Tumors (RECIST), Version 1.1 227
Appendix 7: Patient Global Impression of Change 238
Appendix 8: Recommended Guidelines for the Management of Binimetinib-associated Interstitial Lung Disease 239

LIST OF ABBREVIATIONS AND DEFINITION OF TERMS

The following abbreviations and special terms are used in this study protocol.

Abbreviation or special term	Explanation
aPTT	activated partial thromboplastin time
AE	adverse event
ALT	alanine aminotransferase
AMP	Association for Molecular Pathology
ANC	absolute neutrophil count
ASCO	American Society of Clinical Oncology
AST	aspartate aminotransferase
ATP	adenosine triphosphate
AUC	area under the concentration-time curve
BCRP	breast cancer resistance protein
BICR	blinded independent central review
BID	twice daily
BOR	best overall response
BRAF	B-RAF proto-oncogene, serine/threonine kinase
$BRAF^{V600E}$	B-RAF proto-oncogene, serine/threonine kinase V600E-mutant
$BRAF^{wt}$	B-RAF proto-oncogene, serine/threonine kinase wild-type
BSA	body surface area
BUN	blood urea nitrogen
CA19-9	cancer antigen 19-9
CAP	College of American Pathologists
CEA	carcinoembryonic antigen
CI	confidence interval
CK	creatine kinase
C_{max}	maximum concentration
CR	complete response
CRA	clinical research associate
CRC	colorectal cancer
CRO	contract research organization
CRP	C-reactive protein
CSR	clinical study report

Abbreviation or special term	Explanation
CT	computed tomography
CTCAE	Common Terminology Criteria for Adverse Events
CV	curriculum vita
CYP	cytochrome P450
DDS	dose-determining data set
DLT	dose-limiting toxicity
DMC	Data Monitoring Committee
DOR	duration of response
EC	ethics committee (includes institutional review board, research ethics board, and institutional ethics committee)
EC_{50}	half maximal effective concentration
ECG	electrocardiogram
ECHO	echocardiogram
ECOG	Eastern Cooperative Oncology Group
ECOG PS	Eastern Cooperative Oncology Group performance status
eCRF	electronic case report form
EDC	electronic data capture system
EGFR	epidermal growth factor receptor
EMA	European Medicines Agency
EORTC	European Organization for Research and Treatment of Cancer
EOT	end of treatment
EQ-5D-5L	EuroQol-5D-5L
ESMO	European Society of Medical Oncology
EU	European Union
FA	folinic acid
FACT-C	Functional Assessment of Cancer Therapy-Colon Cancer
FAS	full analysis set
FDA	United States Food and Drug Administration
FDG-PET	fluorodeoxyglucose positron emission tomography
FOLFIRI	5-fluorouracil/folinic acid/irinotecan
FOLFOX	5-fluorouracil/folinic acid/oxaliplatin
FOLFOXIRI	5-fluorouracil/folinic acid/oxaliplatin/irinotecan
FSH	follicle-stimulating hormone

Abbreviation or special term	Explanation
5-FU	5-fluorouracil
GCP	Good Clinical Practice
G-CSF	granulocyte colony-stimulating factor
GI	gastrointestinal
GLP	Good Laboratory Practice
GM-CSF	granulocyte-macrophage colony-stimulating factor
hCG	human chorionic gonadotropin
HDPE	high-density polyethylene
hERG	human ether-a-go-go-related gene
HFSR	rash, hand foot skin reaction
HIV	human immunodeficiency virus
HR	hazard ratio
ICF	informed consent form
IC_{50}	half maximal inhibitory concentration
ICH	International Conference on Harmonisation of Technical Requirements for Registration of Pharmaceuticals for Human Use
ITT	intent-to-treat
IV	intravenous(ly)
IWRS	interactive web response system
KA	keratoacanthoma
KM	Kaplan-Meier
KRAS	V-Ki-ras2 Kirsten rat sarcoma viral oncogene homolog
LH	luteinizing hormone
LLN	lower limit of the normal reference range
LVEF	left ventricular ejection fraction
mCRC	metastatic colorectal cancer
MedDRA	Medical Dictionary for Regulatory Activities
MRI	magnetic resonance imaging
MSI	microsatellite instability
MTD	maximum tolerated dose
MUGA	multi-gated acquisition
NaF PET	sodium fluoride positron emission tomography
NCCN	National Comprehensive Cancer Network

Abbreviation or special term	Explanation
NCI	National Cancer Institute
NGS	next generation sequencing
OAT	organic anionic transporter
OATP	organic anion-transporting peptide
OCT	optical coherence tomography
OCT	organic cationic transporter
ORR	objective response rate (overall response rate)
OS	overall survival
PCR	polymerase chain reaction
PD	progressive disease
PFS	progression-free survival
PGIC	Patient Global Impression of Change
P-gp	P-glycoprotein
pH	hydrogen ion concentration
PK	pharmacokinetic(s)
PO	oral(ly)
PPS	per-protocol set
PR	partial response
QD	once daily
QLQ-C30	Quality of Life Questionnaire for Cancer Patients
QoL	quality of life
QTc	corrected QT interval
QTcF	QT interval corrected for heart rate using Fridericia’s formula
RECIST	Response Evaluation Criteria in Solid Tumors
RPED	retinal pigment epithelial detachment
RP2D	recommended Phase 2 dose
RVO	retinal vein occlusion
SAE	serious adverse event
SAP	statistical analysis plan
SC	Steering Committee
SCC	squamous cell carcinoma
SD	stable disease

**Abbreviation or special term**	**Explanation**
SOP	standard operating procedure
SPC	summary of product characteristics
SS	safety set
$t_{1/2}$	terminal half-life
T max	Time of maximaum plasma concentration
TdP	Torsade de Pointes
UGT	UDP-glucuronosyl transferase
ULN	upper limit of normal
US	United States
v	version
wt	wild-type

1.0 INVESTIGATORS AND STUDY ADMINISTRATIVE STRUCTURE

The Principal Investigator is the person responsible for the conduct of the study at the investigational site. A subinvestigator is any member of the clinical study team designated and supervised by the Principal Investigator to perform critical study-related procedures and/or to make important study-related decisions.

Prior to study initiation, the Principal Investigator at each site must provide to Array BioPharma Inc. (Array BioPharma/Sponsor) a signed protocol signature page, a fully executed and signed United States (US) Food and Drug Administration (FDA) Form 1572 (or equivalent) and a Qualified Investigator Undertaking form for Investigators at Canadian sites, a current curriculum vita (CV), medical license and a financial disclosure form. Financial disclosure forms, current CVs and medical licenses must also be provided for all subinvestigators listed on Form 1572 who will be directly involved in the treatment or evaluation of patients in this study.

The study will be administered and monitored by employees or representatives of Array BioPharma and/or a contract research organization (CRO) in accordance with all applicable regulations. Clinical research associates (CRAs) will monitor each site on a periodic basis and perform verification of source documentation for each patient. The Array BioPharma Drug Safety Department (and/or the CRO, if applicable) will be responsible for ensuring timely reporting of expedited serious adverse event (SAE) reports to regulatory authorities and Investigators.

2.0 INTRODUCTION

2.1 Metastatic Colorectal Cancer

Metastatic colorectal cancer (mCRC) continues to be a serious, life-threatening condition. In the US, it is the fourth most common type of cancer with approximately 130,000 new cases projected in 2015, and it is the second leading cause of cancer mortality with nearly 50,000 projected deaths in 2015 (Siegel et al. 2015). It is also the second most common type of cancer in men and women in Europe. In 2012, almost 450,000 new cases were diagnosed and colorectal cancer (CRC) was responsible for 215,000 deaths. Approximately 25% of patients present with metastases and 50% of patients eventually develop metastatic disease (Van Cutsem et al. 2014). Standard therapy in patients with unresectable mCRC includes combination regimens with cytotoxic and targeted agents. In the last decade, substantial advances in the treatment of mCRC have resulted in an improvement in overall survival (OS) from 10 to 12 months to more than 20 months (Grothey and Goldberg 2004). This improvement has occurred with the addition of irinotecan, oxaliplatin, bevacizumab, cetuximab, and panitumumab to the standard treatment with 5-fluorouracil (5-FU)/folinic acid (FA).

2.2 *BRAF*^{V600E} Colorectal Cancer

BRAF mutations, which lead to constitutive activation of *BRAF* kinase and sustained RAS/RAF/MEK/ERK pathway signaling resulting in increased cell proliferation and survival (Corcoran et al. 2012), are found in up to 20% of patients with CRC with lower prevalence in more advanced patient populations (De Roock et al. 2010, Sorbye et al. 2015).

The presence of a *BRAF* mutation is considered a marker for poor prognosis in patients with mCRC and is associated with a median survival of approximately 12 to 14 months relative to 21 to 25 months for patients with *BRAF*wild-type (*BRAF*^{wt}) tumors (Van Cutsem et al. 2011, Sorbye et al. 2015). In a retrospective analysis of patients with chemo-refractory mCRC treated with chemotherapy and an anti-epidermal growth factor receptor (EGFR) agent, the median progression-free survival (PFS) and OS in patients with *BRAF*-mutant tumors was 8 weeks and 26 weeks, respectively, compared with a PFS of 26 weeks and an OS of 54 weeks in patients with *BRAF*^{wt} tumors (De Roock et al. 2010). In a study evaluating 5-FU/FA/irinotecan (FOLFIRI) plus panitumumab in a pure second-line setting, patients with *BRAF*-mutant mCRC had a median PFS of 2.5 months and an OS of 4.7 months, compared with a PFS and an OS of 6.9 and 18.7 months, respectively, in patients with *BRAF*^{wt} tumors (Peeters et al. 2014). Finally, in 71 patients with *BRAF*^{V600E} mCRC treated at MD Anderson Cancer Center between 2003 and 2012, the median PFS with second-line therapy was 10 weeks in the overall group (n=58) and 12 weeks in patients who were treated with an irinotecan-based regimen (n=39), 28 of whom had received panitumumab or cetuximab concomitantly with irinotecan. The median PFS in patients receiving 2nd- or 3rd-line therapy corresponded to the timing of the first restaging scan (Morris et al. 2014) suggesting that more efficacious therapies are urgently needed. Currently, there are no agents specifically indicated for patients with *BRAF*^{V600E} mCRC. However, since

BRAF and *KRAS* mutations are almost always mutually exclusive, patients with *BRAF*^{V600E} mCRC have typically been treated with standard-of-care regimens for *KRAS*wild-type (*KRAS*^{wt}) mCRC. Patients with *KRAS*^{wt} mCRC typically receive infusional 5-FU/FA/oxaliplatin (FOLFOX) with or without bevacizumab as initial therapy. Common regimens following progression on first-line therapy include infusional FOLFIRI or irinotecan with or without cetuximab or panitumumab. Recent data suggests that the combination of 5-FU/FA, oxaliplatin and irinotecan (FOLFOXIRI) with bevacizumab may be more efficacious than FOLFIRI plus bevacizumab in patients with *BRAF*^{V600} mCRC (Cremolini et al. 2015).

While data suggest *BRAF* mutation status has clear prognostic value in mCRC, the predictive value of *BRAF* mutation status for response and benefit from EGFR-directed treatments, such as cetuximab, remain controversial. Retrospective analyses of recent trials have suggested that *BRAF* mutations are not predictive of outcome with EGFR-directed therapies (Tol and Punt 2010, Van Cutsem et al. 2011, Bokemeyer et al. 2012, Oliner et al. 2013) in certain settings whereas other analyses have suggested that cetuximab and panitumumab are most active in patients with *BRAF*^{wt} mCRC (Di Nicolantonio et al. 2008, De Roock et al. 2010). In a recent meta-analysis of 7 randomized control trials in which patients received panitumumab or cetuximab in different lines of therapy, and with a range of background chemotherapy, no significant interaction was noted between the benefits of anti-EGFR therapy (measured as OS and PFS) and the presence of *BRAF* mutation (Rowland et al. 2015). These results suggest that anti-EGFR therapy may have benefit for patients with *BRAF*^{V600E} CRC. Consistent with this recent publication, a joint committee of the American Society for Clinical Pathology (ASCP), the College of American Pathologists (CAP), the Association for Molecular Pathology (AMP), and the American Society of Clinical Oncology (ASCO) issued draft guidelines for the evaluation of molecular markers for colorectal cancer which stated that there is insufficient evidence to recommend *BRAF*^{V600} mutation status as a predictive molecular marker for response to anti-EGFR inhibitors (ASCP/CAP/ASCO/AMP Draft Recommendations Summary 2015). Based on these data and recommendations, EGFR-directed therapies remain an appropriate component of therapy for patients with *BRAF*^{V600E} mCRC.

2.2.1 Inhibition of BRAF, MEK, and EGFR in *BRAF*-V600E Colorectal Cancer Cells

Cancer cells with *BRAF* mutations are highly dependent on MEK/ERK signaling. As demonstrated in melanoma cells, MEK-dependent activation of MAPK signaling occurs following BRAF inhibition and near-complete inhibition of phospho-ERK is required for tumor responses (Corcoran et al. 2012). The combination of a BRAF inhibitor and a MEK inhibitor has been shown to be more active than either agent alone presumably due to delaying or preventing resistance (Thakur and Stuart 2014). Nonclinical work in CRC cells has shown that BRAF inhibition causes a rapid feedback activation of EGFR that supports continued proliferation of *BRAF*^{V600E} CRC tumor cells (Corcoran et al. 2012, Prahallad et al. 2012). Activation of EGFR can be effectively prevented by the combination of vemurafenib with anti-EGFR agents such as the small-molecule kinase inhibitor, erlotinib, or the monoclonal antibody, cetuximab. These

reports suggest that activation of EGFR may partially explain the limited therapeutic effect of BRAF inhibitor monotherapy in patients with *BRAF*^{V600E} mCRC and that this can be overcome with concomitant EGFR inhibition. The dependence of *BRAF*-mutant CRC cells on MAPK signaling provides a compelling rationale for inhibiting MEK in addition to BRAF and EGFR to achieve more robust inhibition of the pathway.

2.2.2 Effects of Encorafenib and Cetuximab in *BRAF*-V600E Colorectal Cancer

Consistent with the published literature, encorafenib in combination with cetuximab has demonstrated robust and synergistic anti-proliferative effects in a human xenograft model of *BRAF*^{V600E} CRC (Figure 1).

In this experiment, encorafenib was administered 20 mg/kg BID and cetuximab was administered 20 mg/kg biweekly. The same concentration and dosing schedule was used for single agents and the combination. Dosing was stopped 29 days following the initiation of treatment. No signs of toxicity or mortality were observed.

Figure 1: Efficacy of Encorafenib and Cetuximab as Single Agents and in Combination in the HT-29 (*BRAF*^{V600E}, PIK3CαP449T) Human CRC Tumor Xenograft Model Grown in Immunocompromised Mice

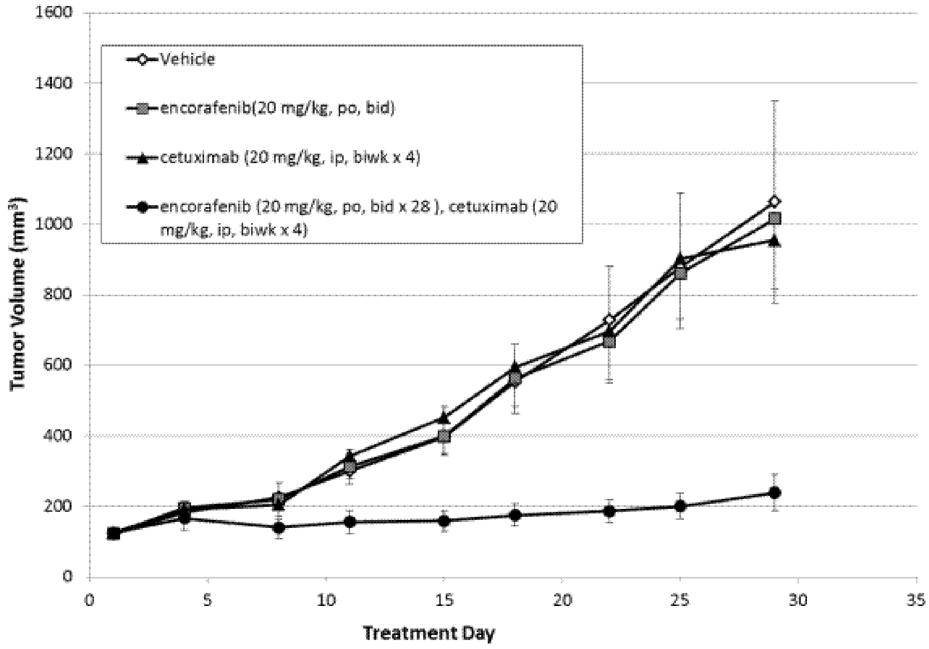

2.2.3 Effects of Encorafenib, Binimetinib, and Cetuximab in *BRAF*-V600E Colorectal Cancer

The combination of binimetinib, encorafenib, and cetuximab was tested in a *BRAF*-V600E mutant human colorectal xenograft model. The average reduction in tumor volume across all animals was better in the group that received the triplet compared to the group that received encorafenib and cetuximab (Figure 2). As seen in the lower panel, the addition of binimetinib resulted in tumor regressions in 8/9 (89%) of the animals compared with 1/8 (13%) of the animals treated with encorafenib and cetuximab without binimetinib.

In this experiment, encorafenib was administered 20 mg/kg BID for 21 days, cetuximab was administered 20 mg/kg biweekly × 10 doses intraperitoneally, and binimetinib was administered 3.5 mg/kg BID orally for 21 days. Dosing was initiated 22 days after tumor implant. The waterfall plots reflect the best response at any time point in the animals.

Figure 2: Effects of Encorafenib and Binimetinib in Combination with Cetuximab in the HT-29 (BRAFV600E, PIK3CαP449T) Human CRC Tumor Xenograft Model Grown in Immunocompromised Mice

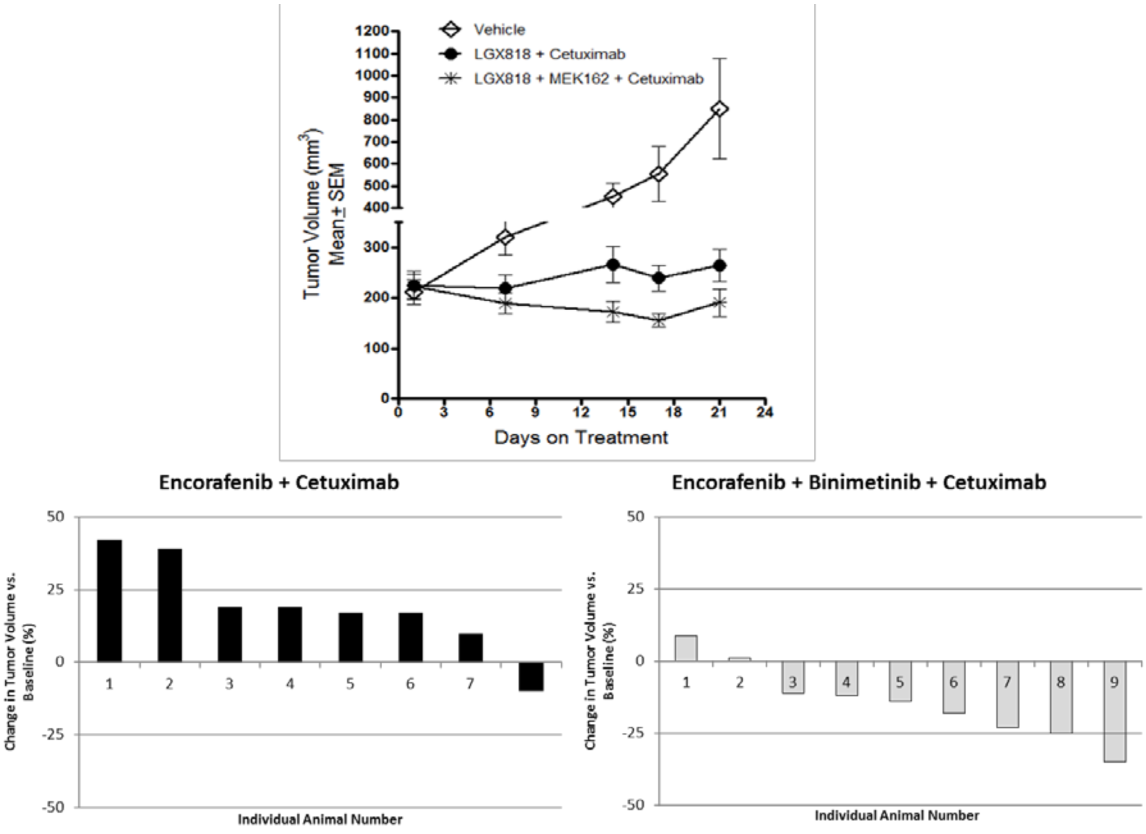

2.2.4 Encorafenib in Colorectal Cancer

2.2.4.1 Single-agent Encorafenib

Encorafenib as a single agent was evaluated in 18 patients with *BRAF*^{V600} mutant mCRC in Study CLGX818X2101. As shown in Table 1, the activity was modest with an overall response rate (ORR) of 5.6%.

Table 1: Summary of Best Overall Response by Treatment Group for Patients with Metastatic Colorectal Cancer in CLGX818X2101 Dose Expansion

	**Number (%) of Patients**		
	**Single-Agent Encorafenib**		
	**300 mg (N=6)**	**450 mg (N=12)`**	**All mCRC (N=18)**
Complete Response (CR)	0 (0.0)	0 (0.0)	0 (0.0)
Partial Response (PR)	1 (16.7)	0 (0.0)	1 (5.6)
Stable Disease (SD)	3 (50)	8 (66.7)	11 (61.1)
Progressive Disease (PD)	1 (16.7)	3 (25.0)	4 (22.2)
Unknown	1 (16.7)	1 (8.3)	2 (11.1)
Overall response rate (CR + PR), 95% CI	1 (16.7) [0.0; 64.1]	0 (0.0) [0.0; 26.5]	1 (5.6) [0.1; 27.3]
Disease control rate (CR + PR + SD), 95% CI	4 (66.7) [22.3; 95.7]	8 (66.7) [34.9; 90.1]	12 (66.7), [41.0; 86.7]

2.2.4.2 Encorafenib Plus Cetuximab

Based on the nonclinical evidence as previously discussed, the combination of encorafenib and cetuximab is being evaluated in patients with *BRAF*^{V600E} mCRC in Study CLGX818X2103. This is an ongoing Phase 1b/2, open-label, multicenter, dose-escalation and dose-expansion study of orally (PO) administered encorafenib and intravenously (IV) administered cetuximab (400 mg/m^2 first dose followed by weekly 250 mg/m^2 IV) with or without alpelisib (a PI3Kα inhibitor) in adult patients with *BRAF*^{V600E} mCRC (1 patient with a *BRAF* non-V600E mutation was also treated). All patients must have had disease progression on at least 1 prior standard-of-care regimen for mCRC or were intolerant to irinotecan-based regimens. Patients treated with prior cetuximab or panitumumab were included only in the dose-escalation phase. A total of 156 patients have been enrolled and treated with at least 1 dose of encorafenib + cetuximab with or without alpelisib, 54 patients in the dose-escalation phase and 102 patients in the dose-expansion phase.

2.2.4.2.1 Preliminary Efficacy in Study CLGX818X2103

Efficacy results are available for the 26 patients treated with encorafenib in combination with cetuximab in the Phase 1b portion of Study CLGX818X2103 and for the 50 patients in the Phase 2 portion of the study.

Phase 1b

Table 2 shows the best overall response (BOR) for patients treated with the dual combination in the Phase 1b portion of the study. Four dose levels of encorafenib QD in combination with cetuximab (400 mg/m^2 initial and 250 mg/m^2 weekly) were evaluated; 100 mg QD (n=2), 200 mg QD (n=7), 400 QD (n=9), and 450 QD (n=8).

Table 2: Summary of Best Overall Response per Investigator Assessment by Treatment Group in Phase 1b (Study CLGX818X2103-Dual Combination)

Response	Number (%) of Patients<br>Dual combination (All doses)<br>(N=26)
Complete Response (CR)	1 (3.8)
Partial Response (PR)	5 (19.2)[a]
Stable Disease (SD)	14 (53.8)
Progressive Disease (PD)	4 (15.4)
Unknown	2 (7.7)
Overall response rate (CR+PR)[a]	6 (23.1)[a]
Disease control rate (CR+PR+SD)	20 (76.9)

[a] Includes 1 unconfirmed PR

In these patients, the median duration of response (DOR) was 34.7 weeks (range 4.1-73.3 weeks). As shown in Figure 3, of the 14 patients with stable disease (SD) as best radiological response, tumor regression was observed in 7 (27%). The median PFS was 3.7 months (95% CI, 2.8-10.6 months) which may underestimate the true effect in the second setting as it includes patients who had received up to 5 prior regimens.

Figure 3: Best Percentage Change from Baseline in Sum of Diameters in Phase 1b (Study CLGX818X2103-Dual Combination)

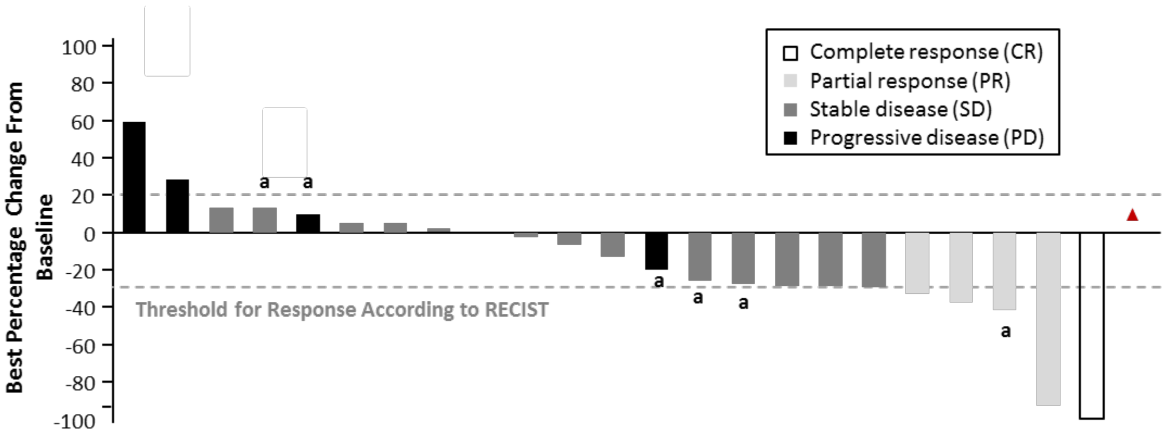

Data cutoff date: February 1, 2015
a Patients treated at the RP2D

The activity of the dual combination appeared durable with 8 (30.7%) patients remaining on treatment for greater than 50 weeks and the longest duration being > 88 weeks (Figure 4).

Figure 4: Summary of Time on Study, by Response in Phase 1b (Study CLGX818X2103-Dual Combination)

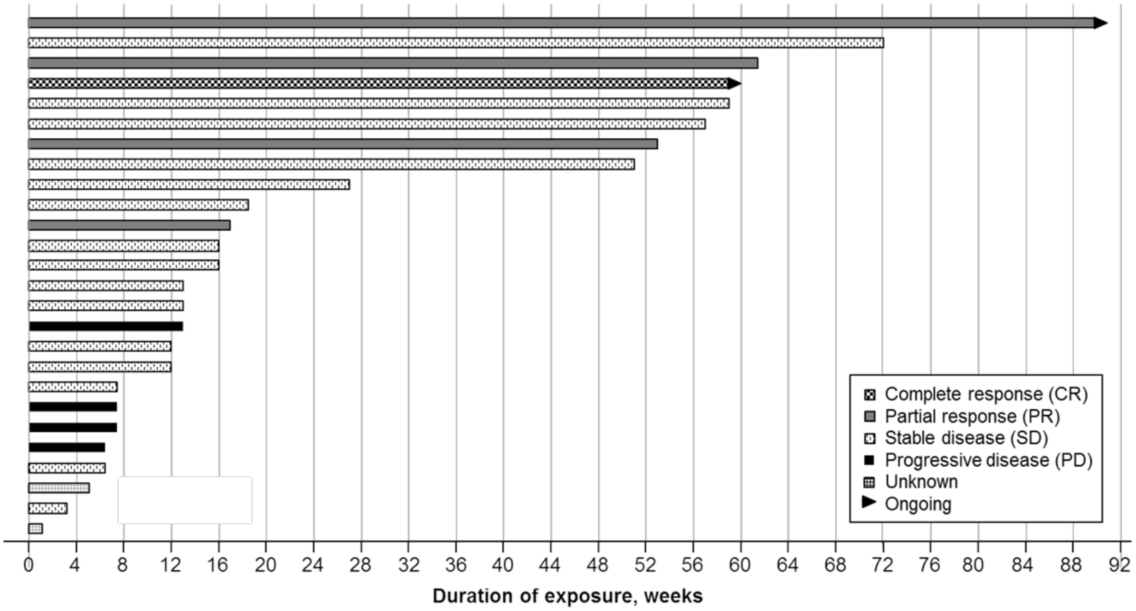

Phase 2

A total of 102 patients have been enrolled in the Phase 2 dose expansion portion of the study, 50 patients in the dual combination treatment group (encorafenib 200 mg QD + cetuximab 400 mg/m^2 followed by 250 mg/m^2 IV weekly) and 52 patients in the triple combination treatment group (encorafenib 200 mg QD + alpelisib 300 mg QD + cetuximab 400 mg/m^2 followed by 250 mg/m^2 IV weekly). Inclusion and exclusion criteria for the Phase 1b and 2 portions of the study were similar except that patients who previously received EGFR-targeted treatments were allowed in Phase 1b, while in Phase 2, patients with prior exposure to EGFR, PI3K, MEK or RAF inhibitors were excluded. In both the Phase 1b and Phase 2 portions, patients must have failed at least 1 prior therapy or have been intolerant of previous treatment with irinotecan. Preliminary results (Table 3) indicate that the level of efficacy demonstrated in the encorafenib plus cetuximab group in Phase 2 was similar to that in the Phase 1b portion (Table 2). The median OS was greater than a year and had not been reached at the time of the analysis.

Table 3: Summary of Best Overall Response per Investigator Assessment by Treatment Group in Phase 2 (Study CLGX818X2103)

	**Number (%) of patients**
	**Encorafenib + cetuximab (N=50)**
Complete Response (CR)	0
Partial Response (PR)	11 (22.0)
Stable Disease (SD)	28 (56.0)
Progressive Disease (PD)	5 (10.0)
Unknown	6 (12.0)
Overall Response Rate (CR + PR)	11 (22.0)
Disease Control Rate (CR + PR + SD)	39 (78.0)

2.2.5 Other BRAF Inhibitors

Preliminary data are available from an ongoing clinical trial in patients with previously treated *BRAF*^{V600E} mCRC which suggest that combined inhibition of MEK, BRAF and EGFR may be more effective than the combination of a BRAF inhibitor and an EGFR inhibitor. As reported by Van Cutsem and colleagues, responses were observed in 2/10 patients (20%) and a PFS of 3.4 months was observed in patients treated with the *BRAF* inhibitor, dabrafenib, plus the anti-EGFR inhibitor, panitumumab. In patients receiving the MEK inhibitor, trametinib, in addition to dabrafenib and panitumumab, responses were observed in 9/35 patients (26%) with a PFS of 4.1 months. The combination of trametinib and panitumumab was evaluated in 19

patients and was marked with no activity (0 CR or PR) and increased toxicity, especially skin toxicity (Van Cutsem et al. 2015). In a separate trial, the combination of dabrafenib plus trametinib without an EGFR inhibitor yielded a response rate of 12% (Corcoran et al. 2015).

Vemurafenib was initially tested as a single agent in a total of 21 patients, 19 of whom were evaluable for response. Among these 19 patients, there was 1 partial response (PR) (5%) and the median PFS was 3.7 months (Kopetz et al. 2010). When combined with panitumumab in 15 total patients of whom 12 were evaluable for response, PRs were observed in 2 (13%) patients (Yeager et al. 2015). In another Phase 1b/2 study (n=19), vemurafenib at doses of 480 mg BID, 720 mg BID, and 960 mg BID was also combined with panitumumab and irinotecan. Of 17 response-evaluable patients, responses were observed in 6 (35%) patients with a median DOR of 8.8 months and median PFS of 7.7 months. The most common adverse events (AEs) (≥ 30%) observed in 19 patients included fatigue 17 (89%), diarrhea 16 (84%), rash 15 (74%), nausea 15 (74%), anemia 14 (74%), myalgia 10 (53%), leukopenia 9 (47%), arthralgia 8 (42%), vomiting 6 (32%), and neuropathy 6 (32%) (Hong et al. 2015).

2.3 Overview of Encorafenib

Encorafenib is a highly selective adenosine triphosphate (ATP)-competitive small-molecule RAF kinase inhibitor, which suppresses the RAS/RAF/MEK/ERK pathway in tumor cells expressing *BRAF*^{V600E}. Similar to other selective small-molecule RAF kinase inhibitors, encorafenib inhibits CRAF (half maximal inhibitory concentration [IC_{50}]=0.30 nM), BRAF (IC_{50}=0.47 nM), as well as BRAF^{V600E} (IC_{50}=0.35 nM) in cell-free assays. However, this class of inhibitor does not inhibit RAS/RAF/MEK/ERK signaling in cells expressing *BRAF*^{wt}. In the human melanoma cell line A375, which expresses *BRAF*^{V600E}, encorafenib potently inhibits phospho-MEK (half maximal effective concentration [EC_{50}]=2 nM), phospho-ERK (EC_{50}=3 nM), and proliferation (EC_{50}=4 nM), resulting in cell cycle arrest and apoptosis. Given the high-degree of selectivity against other kinases, encorafenib has no antiproliferative activity in tumor cell lines that express *BRAF*^{wt} and is highly selective for *BRAF* mutations in cell lines containing V600E/D/K, with the greatest sensitivity observed in *BRAF*^{V600E} melanoma and CRC lineages.

Encorafenib has been evaluated in multiple human tumor xenograft models grown in nude mice. Similar to the in vitro profile, the antitumor activity was restricted to tumors expressing *BRAF*^{V600E}, while there was no antitumor effect in xenograft models expressing *BRAF*^{wt} or *CRAF*^{wt}. Nonclinical in vivo data suggest that encorafenib has a wide therapeutic index and that regression of *BRAF*^{V600E} human melanoma tumor xenografts is associated with a strong and sustained inhibition of the RAF/MEK/ERK pathway.

2.3.1 Nonclinical Safety Pharmacology and Toxicology

In 1- to 4-week toxicology studies in rats and cynomolgus monkeys, encorafenib was well tolerated at systemic exposures which result in tumor regression in mouse xenograft studies. Findings included hyperplasia and hyperkeratosis in the skin (plantar surface of feet) and

non-glandular stomach in the rat. An absence of the later stages of spermatid maturation in male rats was also observed. Significant mortality/morbidity was observed mostly in female rats at the highest dose of 400 mg/kg/day, a dose well above the maximum tolerated dose (MTD) in rats.

In the 13-week toxicology study in monkeys, the only test article-related finding was blister-like lesions identified over the macular region of the retina, observed in 2 monkeys treated at a dose of 60 mg/kg/day. Exposure (maximum AUC_{0-24} achieved in the study at any time point) in the 2 affected monkeys was 5 to 8-fold and 4 to 6-fold that achieved at the 300 and 450 mg QD dose levels, respectively, in humans at steady state in Clinical Study CLGX818X2101. One of the animals with this finding showed evidence of recovery. Histopathology examination of the affected eyes suggested that the findings were similar to the retinopathy associated with MEK inhibitors.

Nonclinical safety pharmacology results did not indicate a risk for corrected QT interval (QTc) prolongation based on: the findings of the Good Laboratory Practice (GLP) human ether-a-go-go-related gene (hERG) assay, on electrocardiogram (ECG) evaluations in the GLP monkey cardiovascular telemetry study, and the GLP 4- and 13-week toxicology studies in monkeys.). Also, there were no clinical signs in the 4-or 13-week GLP-compliant studies in the rat and monkey that would indicate an effect on the central nervous system or respiratory system.

The GLP Ames and chromosomal aberration assays as well as a rat micronucleus study indicated that encorafenib is not genotoxic.

Encorafenib showed a potential for phototoxicity in a screen 3T3 neutral red uptake (NRU) in vitro assay. In patients treated with encorafenib, as a single-agent, in the range of doses of 300 to 450 mg QD, the incidence of photosensitivity was 2.9% and 5.6% in the melanoma and colorectal cohorts, respectively (Study CLGX818X2101).

2.3.2 Pharmacokinetics and Metabolism

Encorafenib is a relatively potent reversible inhibitor of cytochrome P450 (CYP)2B6, CYP2C9, and CYP3A4/5 and a weak ($IC_{50} \geq 20$ μM) reversible inhibitor of CYP1A2, CYP2C8, CYP2C19, and CYP2D6. Encorafenib is also a time-dependent inhibitor and at high concentrations (≥ 10 to 50 µM), a potential inducer of CYP3A4 based on an in vitro pregnane X receptor (PXR) reporter gene assay. Furthermore, encorafenib inhibits UDP-glucuronosyl transferase (UGT)1A1 and is a substrate of P-glycoprotein with high apparent passive permeability. It is also an inhibitor of breast cancer resistance protein (BCRP) (IC_{50}=10 to 25 µM). Finally, encorafenib inhibits the renal transporters OAT1, OAT3, and OCT2 and the hepatic transporters, organic anion-transporting polypeptide (OATP)1B1 and OATP1B3.

Encorafenib is primarily metabolized by CYP3A4 (> 50%) and to lesser degrees by CYP2D6 and CYP2C19.

In a Phase 1 study in patients with locally advanced or metastatic *BRAF*^{V600E} melanoma (Study CLGX818X2101, dose escalation phase), encorafenib exposures on Cycle 1 Day 15 were consistently decreased by 30% to 60% compared with those at Day 1, probably due to induction of CYP450 enzymes. Area under the concentration-time curve (AUC) and maximum concentration (C_{max}) ratios at steady-state concentrations (Day 15) relative to Day 1 did not appear to change with dose. The trough concentration on and after Cycle 2 Day 1 did not show a trend of further decline, suggesting that Cycle 1 Day 15 was close to or at the time of steady-state concentration. At doses tested in the study (50, 100, 150, 200, 300, 450, 550 or 700 mg once daily [QD] and 75, 100, and 150 mg BID), the average concentrations of encorafenib were above the predicted efficacious concentrations based on nonclinical xenograft models (0.135 µg/mL). The terminal half-life of encorafenib is approximately 4 hours.

Additional details are provided in the encorafenib Investigator's Brochure.

2.3.3 Clinical Safety

2.3.3.1 Single-agent Encorafenib

Single-agent encorafenib was evaluated in a first-in-human, Phase 1 dose-escalation/dose-expansion study (Clinical Study CLGX818X2101) in adult patients with *BRAF*^{V600E} locally advanced or metastatic melanoma (dose-escalation and dose-expansion phases) or mCRC (dose-expansion phase only). A total of 107 patients were treated at doses ranging from 50 mg to 700 mg QD.

In the dose-expansion phase of Study CLGX818X2101, the first 34 patients received encorafenib 450 mg QD (i.e., MTD). Nine of these patients experienced a dose-limiting toxicity (DLT) during Cycle 1, leading to a dose reduction to 300 mg QD in 6 patients and discontinuation of encorafenib for the remaining 3 patients. Among the patients who did not have a DLT, the encorafenib dose was reduced to 300 mg QD in 8 patients, and 1 patient discontinued due to an AE. Based on these findings, 300 mg QD was identified as the recommended Phase 2 dose (RP2D).

Eighteen patients with mCRC received encorafenib in the dose-expansion phase of the study. Twelve patients received encorafenib 450 mg QD and 6 patients received 300 mg QD. Dose-limiting toxicities were reported in 3 patients treated at the 450 mg QD dose and in none of the patients treated at the 300 mg QD dose. The most common (occurring in ≥ 20% of patients) AEs, regardless of causality, observed at the MTD of 450 mg QD and the RP2D of 300 mg QD in the mCRC patients in the dose-expansion phase are shown in Table 4.

Table 4: Adverse Events (≥ 20%), Regardless of Study Drug Relationship, by Preferred Term - Study CLGX818X2101 in mCRC Patients in the Dose-Expansion Phase

	Number (%) of Patients					
	Encorafenib					
	300 mg QD (N=6)		450 mg QD (N=12)		Total (N=18)	
Preferred Term	All grades	Grade 3/4	All grades	Grade 3/4	All grades	Grade 3/4
Palmar-plantar erythrodysaesthesia syndrome	4 (66.7)	0	8 (66.7)	3 (25.0)	12 (66.7)	3 (16.7)
Myalgia	1 (16.7)	0	7 (58.3)	2 (16.7)	8 (44.4)	2 (11.1)
Pruritus	3 (50.0)	0	5 (41.7)	0	8 (44.4)	0
Vomiting	5 (83.3)	0	3 (25.0)	2 (16.7)	8 (44.4)	2 (11.1)
Arthralgia	2 (33.3)	0	5 (41.7)	2 (16.7)	7 (38.9)	2 (11.1)
Hyperkeratosis	3 (50.0)	0	4 (33.3)	0	7 (38.9)	0
Decreased appetite	1 (16.7)	0	6 (50.0)	1 (8.3)	7 (38.9)	1 (5.6)
Dry skin	1 (16.7)	0	6 (50.0)	0	7 (38.9)	0
Insomnia	1 (16.7)	0	5 (41.7)	1 (8.3)	6 (33.3)	1 (5.6)
Rash	2 (33.3)	0	4 (33.3)	0	6 (33.3)	0
Weight decreased	2 (33.3)	1 (16.7)	4 (33.3)	0	6 (33.3)	1 (5.6)
Alopecia	1 (16.7)	0	4 (33.3)	0	5 (27.8)	0
Asthenia	0	0	5 (41.7)	2 (16.7)	5 (27.8)	2 (11.1)
Fatigue	2 (33.3)	0	2 (16.7)	0	4 (22.2)	0
Keratosis pilaris	1 (16.7)	0	3 (25.0)	0	4 (22.2)	0
Constipation	1 (16.7)	0	3 (25.0)	0	4 (22.2)	0
Melanocytic naevus	1 (16.7)	0	3 (25.0)	0	4 (22.2)	0
Edema peripheral	3 (50.0)	0	1 (8.3)	0	4 (22.2)	0
Abdominal pain	2 (33.3)	1 (16.7)	2 (16.7)	1 (8.3)	4 (22.2)	2 (11.1)

2.3.3.2 Combination Treatment with Encorafenib plus Cetuximab

Study CLGX818X2103 is a Phase1b/2 study evaluating the safety /tolerability and preliminary efficacy of encorafenib plus cetuximab (400 mg/m^2 first dose followed by weekly 250 mg/m^2 IV) and encorafenib and the PI3K inhibitor alpelisib (BYL719) in combination with cetuximab at the same dose.

2.3.3.2.1 CLGX818X2103 Phase 1b Dose Escalation

Data are available from the first 26 patients with locally determined *BRAF*^{V600E} mCRC who were treated with encorafenib and cetuximab during the Phase 1b portion of the previously described Study CLGX818X2103 (see Section 2.2.4.2). Four dose levels of encorafenib QD in combination with cetuximab (400 mg/m^2 initial and 250 mg/m^2 weekly) were evaluated in the dual combination treatment arm: 100 mg QD (n=2), 200 mg QD (n=7), 400 QD (n=9), and 450 QD (n=8), respectively. Three DLTs were observed, Grade 3 arthralgia (200 mg), Grade 3 vomiting (400 mg) and Grade 3 QT prolongation (450 mg).

The most frequently reported AEs (≥ 20% of patients), regardless of causality, observed in patients treated with the dual combination are shown in Table 5.

Table 5: Adverse Events (≥ 20% of Patients) Regardless of Causality by Preferred Term in Phase 1b (Study CLGX818X2103, Safety Set - Dual Combination Treatment)

	Number (%) of Patients	
	Encorafenib + Cetuximab (All dose levels combined: N=26)	
Preferred Term	All grades	Grade 3/4
**Total**	**26 (100)**	**18 (69.2)**
Fatigue	13 (50.0)	3 (11.5)
Vomiting	12 (46.2)	2 (7.7)
Dyspnea	9 (34.6)	1 (3.8)
Abdominal pain	8 (30.8)	3 (11.5)
Nausea	8 (30.8)	0
Back pain	7 (26.9)	1 (3.8)
Constipation	7 (26.9)	1 (3.8)
Decreased appetite	7 (26.9)	0
Hypophosphatemia	7 (26.9)	5 (19.2)
Infusion related reaction	7 (26.9)	0
Weight decreased	7 (26.9)	0
Cough	6 (23.1)	0
Headache	6 (23.1)	0
Myalgia	6 (23.1)	0
Pain in extremity	6 (23.1)	0
Stomatitis	6 (23.1)	0

In the triple combination cohort, dose levels included encorafenib 200 mg/alpelisib 300 mg and encorafenib 300 mg/alpelisib 200 mg in combination with the same cetuximab regimen. An MTD was not identified for either the double combination arm (i.e., encorafenib at doses up to 450 mg in combination with cetuximab) or the triple combination arm (i.e., encorafenib at doses up to 300 mg in combination with alpelisib 200 mg and cetuximab). Although doses of encorafenib up to 450 mg were tolerated in the dual combination, based on the overall toxicity of the triple combination, and in order to determine the contribution of alpelisib to the efficacy of the triple combination, the same dose of encorafenib, 200 mg QD, was selected for both the dual combination and the triple combination arms of the Phase 2 portion of the study.

2.3.3.2.2 CLGX818X2103 Phase 2 Dose Expansion

Preliminary data are available from the ongoing Phase 2 portion of the study. The interim data are generally consistent with the observations from the Phase 1b portion of the study (Table 6).

Table 6: Adverse Events Regardless of Causality in ≥ 15% of Patients by Preferred Term in the Phase 2 Interim Analysis (Study CLGX818X2103, Safety Set)

	Number (%) of Patients	
	Encorafenib + Cetuximab (N=50)	
**Preferred Term**	**All grades**	**Grade 3/4**
**Total**	**49 (98.0)**	**29 (58.0)**
Fatigue	25 (50.0)	2 (4.0)
Nausea	23 (46.0)	0
Abdominal pain	21 (42.0)	4 (8.0)
Decreased appetite	17 (34.0)	1 (2.0)
Arthralgia	17 (34.0)	0
Vomiting	16 (32.0)	0
Headache	16 (32.0)	0
Diarrhea	14 (28.0)	1 (2.0)
Lipase increased	14 (28.0)	9 (18.0)
Pyrexia	13 (26.0)	0
Constipation	13 (26.0)	2 (4.0)
Back pain	12 (24.0)	1 (2.0)
Dermatitis acneiform	9 (18.0)	0
Dyspnea	8 (16.0)	0
Rash	8 (16.0)	0
Anemia	8 (16.0)	3 (6.0)
Dry skin	8 (16.0)	0
Pruritus	8 (16.0)	0
Myalgia	8 (16.0)	1 (2.0)

2.4 Overview of Binimetinib

Binimetinib (also known as MEK162 or ARRY-438162) is a potent and selective allosteric, ATP-uncompetitive inhibitor of MEK1/2 that is active in inhibiting pERK and growth of *BRAF*-mutant cancer cells in the low nanomolar range. In oncology settings, binimetinib is currently being investigated both as a single agent and in combination with RAF or PI3K or inhibitors in patients with selected advanced or metastatic solid tumors, including biliary cancer, CRC and melanoma.

2.4.1 Nonclinical Safety Pharmacology and Toxicology

Acute, subchronic, chronic and reproductive toxicity, genotoxicity and phototoxicity studies were completed in rats and monkeys to support the chronic administration of binimetinib to adult patients. There was no evidence of a genotoxic potential in vitro or in vivo. The adverse effects of MEK inhibitors in humans are similar to those observed in rats and monkeys, with the exception of ocular findings. These adverse effects include gastro-intestinal intolerance and diarrhea, rash (skin findings in rats only), retinal events (only seen in humans) and retinal vein occlusion (rarely seen in humans). In vitro and in vivo phototoxicity studies conducted in mice indicate that binimetinib has a low risk of weak phototoxic potential at therapeutic doses. Furthermore, there has been no evidence of phototoxicity or photosensitivity in humans being treated with binimetinib for cancer or for rheumatoid arthritis.

Given the embryo-lethal effects seen in rats and rabbits and the teratogenic effects seen in rabbits, binimetinib should not be used in pregnant women. Women of child-bearing potential must be advised to use highly effective contraception methods.

For further details, please refer to the current binimetinib Investigator's Brochure.

2.4.2 Clinical Pharmacokinetics

Nonclinical in vitro and in vivo data indicated that binimetinib is metabolized by multiple routes but primarily by glucuronidation pathways (mainly via UGT1A1, 1A3 and 1A9) and to a lesser extent by oxidation pathways (mainly via CYP1A2 and 2C19). In vitro, binimetinib has the potential to inhibit CYP2B6. It is not considered a time dependent inhibitor of CYP1A2, CYP2C9, CYP2D6 and CYP3A.

Binimetinib is rapidly absorbed after oral administration with a median time (T_{max}) to reach C_{max} around 1.5 hours at steady-state. The mean terminal half-life ($t_{1/2}$) in healthy subjects is approximately 10 hours.

For further details on non-clinical pharmacokinetics (PK) and metabolism, please refer to the current binimetinib Investigator's Brochure.

2.4.3 Clinical Experience

2.4.3.1 Clinical Safety

As of 20 January 2016, a total of 2555 healthy subjects and patients have received at least 1 dose of binimetinib and are therefore eligible for inclusion in the overall safety population of binimetinib, which comprises 220 healthy subjects, 164 patients with rheumatoid arthritis, 12 patients with hepatic dysfunction and 2159 patients with advanced cancer.

For further details, please refer to the current binimetinib Investigator's Brochure.

2.4.3.1.1 Single-agent Binimetinib

The single agent safety of binimetinib in oncology patients is based on data from 884 patients across 7 clinical trials. The recommended single-agent dose is 45 mg PO BID. The most frequently reported AEs considered related to single-agent binimetinib, regardless of grade and binimetinib dose, have been dermatologic events (rash, dermatitis acneiform), gastrointestinal (GI) events (nausea, vomiting, diarrhea), edema peripheral, fatigue and increased CK. The majority of these AEs have been Grade 1 or 2, with the exception of increased blood CK, which was reported as Grade 3/Grade 4 for 24% of patients in Clinical Study CMEK162X2201 and for 17.2% and 16.6% of patients in the dose-escalation and dose-expansion portions of Clinical Study CMEK162X1101.

Additional AEs which require monitoring include ejection fraction decreases and retinal toxicities. Cardiac events, in particular events related to asymptomatic ejection fraction decrease as detected by protocol-specified echocardiogram (ECHO)/multi-gated acquisition (MUGA) scans and cardiac failure, are a class effect of MEK inhibitors. In Clinical Study CMEK162X2201 of binimetinib as a single agent, at the recommended dose of 45 mg BID (N = 158 patients), the incidence of left ventricular ejection fraction (LVEF) decreases reported as AEs were observed in 10 patients (6.3%). Nine of these patients presented with an LVEF above 50% at Baseline, and 1 patient had an LVEF of 48% at Baseline. These AEs were reversible in 6 patients (60.0%) and were ongoing in 4 patients at the time of cutoff. Five patients (3.2%) had Grade 3 events; all considered related to study treatment. No Grade 4 events were observed. Four patients discontinued the study treatment due to decreased LVEF, of whom 2 patients experienced Grade 3 events.

Retinopathy is also a well-known MEK inhibitor-associated class effect. The most common retinal events that have been reported are retinal detachment, detachment of retinal pigment epithelium and chorioretinopathy. In Clinical Study CMEK162X2201 of binimetinib as a single agent, at the recommended dose of 45 mg BID (N = 158), observations related to retinal events (retinopathy, retinal detachment, detachment of retinal pigment epithelium, chorioretinopathy, retinal disorder, retinal edema, macular edema, maculopathy, and retinal cyst) were as follows. Forty-nine of the 158 patients (31%) reported retinal events. The majority of patients presented

with low severity retinal events; maximum severity was Grade 1 (n = 31, 19.6%), Grade 2 (n = 15, 9.5%), Grade 3 (n = 3, 1.9%). All Grade 3 retinal events were considered related to the study drug. No retinal events observed in patients receiving 45 mg BID led to study drug discontinuation and no Grade 4 retinal events were reported. Events occurred early in treatment, with a median time to onset of 15 days.

Among the preferred terms reported by the 49 patients with retinal events, chorioretinopathy (n = 6; 10.1%) was the most commonly observed, followed by retinopathy and retinal detachment (n = 13 each, 8.2%). In the majority of patients, retinal events were transient with a median duration of 29 days. Most patients continued the planned treatment without treatment modification. Sixteen patients (10.1%) had either dose reduction or temporary study drug interruption.

Analyses of retinal events confirmed by optical coherence tomography (OCT) in cancer patients treated with binimetinib as a single-agent monotherapy at the recommended dose of 45 mg BID, revealed that most of the events were bilateral, early onset, mild or moderate (Grade 1/2) in severity, transient and reversible, and in most of the cases, did not lead to treatment discontinuation.

2.4.3.1.2 Binimetinib in Combination with Anti-EGFR Agents

The combination of binimetinib and panitumumab was evaluated in patients with mCRC in the Phase 1b/2 Clinical Study CMEK162X2116. Ten patients were treated in the Phase 1b portion of the study with binimetinib (45 mg BID) and the labeled dose of panitumumab (6 mg/kg IV biweekly) which was declared the MTD/RP2D. An additional 40 patients were enrolled in the Phase 2 portion of the study. In the Phase 2 portion, AEs, regardless of causality, reported in ≥ 15% of patients included all grade (%)/Grade 3-4 (%): diarrhea (70.0/12.5), vomiting (55.0/2.5), rash (50.0/12.5), nausea (47.5/5.0), fatigue (35.0/5.0), abdominal pain (32.5/2.5), dermatitis acneiform (32.5/5.0), blood creatine kinase (CK) increased (27.5/7.5), dry skin (25.0/5.0), anemia (20.0/10.0), asthenia (20.0/2.5), constipation (20.0/0), hypokalemia (20.0/12.5), pyrexia (20.0/0), stomatitis (20.0/0), AST increased (17.5/5.0), blood creatinine increased (15.0/2.5), chills (15.0/0) and hypomagnesemia (15.0/0).

2.5 Overview of the Combination of Encorafenib and Binimetinib

2.5.1 Nonclinical Pharmacokinetics and Metabolism

Although the in vitro data suggest a potential for encorafenib to affect the PK of binimetinib-based inhibition of UGT1A1, no significant drug-drug interaction was observed up to the highest doses tested in Clinical Study CMEK162X2110 (800 mg QD encorafenib in combination with 45 mg BID of binimetinib).

2.5.2 Clinical Experience

The ongoing Clinical Study CMEK162X2110 is an open label, dose finding, Phase 1b/2 study to determine the MTD(s) and/or RP2D(s) of encorafenib in combination with binimetinib (dual combination), and in combination with encorafenib and LEE011 (triple combination), in selected patient populations (locally advanced or metastatic melanoma, mCRC or any other solid tumor positive for either BRAF-V600 mutation). Forty-seven patients have been enrolled (with different tumor types, including melanoma, mCRC, papillary thyroid cancer (PTC) and non-small cell lung cancer (NSCLC) and treated with at least 1 dose of encorafenib and binimetinib across several dose levels in the Phase 1b dose escalation, and 79 patients with either melanoma or mCRC have been enrolled and treated with at least 1 dose of encorafenib and binimetinib in the Phase 2 portion of the study.

In Phase 1b, 47 patients were treated with binimetinib 45 mg BID and encorafenib at 7 dose levels [Dose levels; 50 (n=6), 100 (n=5), 200 (n=4), 400 (n=5), 450 (n=13), 600 (n=8) and 800 (n=6) mg QD)]. The MTD was not reached up to the highest tested dose of 800 mg + 45 mg (encorafenib QD + binimetinib BID). Initially, two RP2Ds were declared for the combinations at 450 mg + 45 mg and 600 mg + 45 mg dose levels. Based on the occurrence of 3 cases of Grade 3 creatinine increase suspected to be drug related in the Phase 2 portion of the study at 600 mg + 45 mg, the confirmed RP2Ds of the combination is 450 mg + 45 mg of encorafenib QD plus binimetinib BID, respectively.

Among the 47 patients treated with the dual combination in Phase 1b, 18 patients were treated at an encorafenib starting dose of 400 or 450 mg QD and 14 patients were treated with ≥ 600 mg QD. The most common AEs (≥ 20%, all grades) in all patients treated in Phase 1b, regardless of causality, were nausea, diarrhea, fatigue, constipation, abdominal pain, vomiting, headache, cough, alanine aminotransferase (ALT) increased, and pain in extremity. The most common National Cancer Institute [NCI] Common Terminology Criteria for Adverse Events (CTCAE) Grade 3 or 4 AEs (≥ 3.0%), regardless of causality, were increases in serum lipase, liver enzymes (ALT, AST), and CK, fatigue, anemia, abdominal pain, and cancer pain. Hypertension of any grade was observed in 6/47 (12.8%) patients and a Grade 3/4 hypertension event was documented in 1 (2.1%) patient.

Among the 79 patients treated with the dual combination in Phase 2, 15 patients were treated at an encorafenib starting dose of 400 or 450 mg QD and 64 were treated with ≥ 600 mg QD. The most common AEs (≥ 20%, all grades), regardless of causality, were diarrhea, nausea, vomiting, arthralgia, fatigue, pyrexia, constipation, aspartate aminotransferase (AST) increased, blood CK increased, ALT increased, retinopathy, and cough. The most common CTCAE Grade 3 or 4 AEs (≥ 3.0 %), regardless of causality, were increases in serum lipase, liver enzymes (ALT, AST), and CK, diarrhea, nausea, vomiting, and anemia. As compared to the respective single-agent therapies, there was a decreased occurrence of skin toxicities in the combination. Among the 29 patients treated with encorafenib 400 or 450 mg, the most common AEs (≥ 20% all grades),

regardless of causality, were diarrhea, nausea, vomiting, pyrexia, arthralgia, fatigue and anemia. Hypertension of any grade was observed in 9/79 (11.4%) patients, and Grade 3/4 hypertension was documented in 2 (2.5%) patients. These data indicate an acceptable safety of the combination of encorafenib and binimetinib (Sullivan et al. 2015).

The combination of binimetinib + encorafenib is also being evaluated in an ongoing 2-part Phase 2 study, CLGX818X2109, in patients with advanced/unresectable *BRAF*^{V600} mutant melanoma. Safety data are available in 88 patients treated in Part 1 of the study in patients treated with binimetinib 45 mg BID + encorafenib 450 mg QD. The preliminary AE findings are described in Table 7 below.

Table 7: Adverse Events Regardless of Causality in ≥ 10% of Patients by Preferred Term in Interim Phase 2 Analysis (Study CLGX818X2109)

	Number (%) of Patients Treated with Encorafenib 450 mg QD + Binimetinib 45 mg BID	
	Total (N=88)	
**Preferred Term**	**All grades**	**Grade 3/4**
**Total**	81 (92.0)	37 (42.0)
Nausea	24 (27.2)	5 (5.7)
Diarrhea	22 (25.0)	0
Fatigue	21 (23.9)	3 (3.4)
Retinopathy	21 (23.9)	0
Vomiting	14 (15.9)	1 (1.1)
Blood creatine phosphokinase increased	14 (15.9)	1 (1.1)
Pyrexia	11 (12.5)	1 (1.1)
Abdominal pain	10 (11.4)	0
Anaemia	10 (11.4)	4 (4.5)
Vision blurred	9 (10.2)	0

Hypertension of any grade and of Grade 3/4 was observed in 5 (5.7%) and 3 (3.4%) patients, respectively.

The combination of encorafenib and binimetinib is being evaluated in the ongoing study, CMEK162B2301: A 2-part phase III randomized, open label, multicenter study of encorafenib and binimetinib versus vemurafenib and encorafenib monotherapy in patients with unresectable or metastatic *BRAF*^{V600} melanoma. The trial has completed enrollment of over 920 patients including approximately 430 randomized to the encorafenib plus binimetinib combination arms.

The study has been monitored by an independent Data Monitoring Committee (DMC) which has not raised any concerns regarding safety/tolerability.

2.6 Overview of Cetuximab

Cetuximab is a recombinant, human/mouse chimeric monoclonal antibody that binds specifically to the extracellular domain of human EGFR on both normal and tumor cells, and inhibits receptor activation by competing with epidermal growth factor and other ligands. In vitro and in vivo assays have shown that binding of cetuximab to EGFR blocks its dimerization and phosphorylation and its consequent activation, resulting in inhibition of cell growth, induction of apoptosis, and decreased matrix metalloproteinase and vascular endothelial factor production. Cetuximab exhibits clinical activity as monotherapy or in combination with chemotherapy and/or radiation in head and neck cancer and mCRC (Cunningham et al. 2004, Baselga et al. 2005, Bonner et al. 2006, Mendelsohn and Baselga 2006, Vermorken et al. 2008).

Cetuximab is approved in several countries for the treatment of patients with CRC or head and neck squamous cell carcinomas. Cetuximab is approved for the treatment of EGFR-expressing, *RAS*^{wt} mCRC in combination with chemotherapeutic agents or as a single agent, in previously untreated patients and in patients who have failed irinotecan- and oxaliplatin-based regimens or who are intolerant to irinotecan-based regimens (see the locally applicable cetuximab label).

2.6.1 Clinical Experience

2.6.1.1 Clinical Pharmacokinetics

Following the recommended dose regimen (400 mg/m^2 initial dose; 250 mg/m^2 weekly dose), concentrations of cetuximab reach steady-state levels by the third weekly infusion. Although the US label for Erbitux® indicates that US-licensed cetuximab (Erbitux) provides approximately 22% greater exposure relative to cetuximab which is approved in the European Union (EU), the dosing and administration guidelines are consistent between the regions.

2.6.1.2 Clinical Efficacy

In patients with mCRC whose disease had progressed during or within 3 months after treatment with an irinotecan-based regimen, treatment with single-agent cetuximab resulted in an objective response rate of 11% and a median time to progression of 1.5 months (Cunningham et al. 2004). In patients with EGFR-expressing mCRC treated with cetuximab in combination with irinotecan in the second-line setting, the median PFS was 4.0 months (95% confidence interval [CI], 3.2-4.1 months) and OS was 10.7 months (95% CI, 9.6 -11.3 months) (Sobrero et al. 2008).

2.6.1.3 Clinical Safety

Across all studies, cetuximab was discontinued in 3% to 10% of patients because of AEs. The main undesirable effects of cetuximab are as follows:

- Skin reaction occurring in 76% to 88% of patients, with severe acneiform rash occurring in 1% to 17% of patients
- Severe (Grade 3-4 by NCI CTCAE) hypomagnesemia in 6% to 17% of patients
- Infusion reactions, with mild to moderate symptoms in 15% to 21% of patients, and severe in 1% to 5% of patients
- Cardiopulmonary arrest and/or sudden death in 2% of patients receiving cetuximab in combination with radiation therapy
- A case of tumor lysis syndrome reported with single-agent cetuximab within 24 hours of administration of the first dose (Krishnan et al. 2008).

More details are provided in the locally approved cetuximab label.

2.7 Overview of Irinotecan

Irinotecan is a topoisomerase-I inhibitor which has been used in patients with mCRC since the late 1990s. In patients with mCRC, it is indicated as a component of first-line therapy or as a single agent following progression on an initial fluorouracil-based regimen (see locally approved irinotecan label).

Irinotecan treatment is most commonly associated with GI toxicity and myelosuppression. The biweekly regimen is associated with Grade 3 or 4 diarrhea in 10% of patients, Grade 2 nausea and Grade 2 vomiting in 10% of patients, and Grade 4 neutropenia in approximately 8% of patients (Martín-Martorell et al. 2008, Pfeiffer et al. 2008).

2.8 Overview of FOLFIRI

Irinotecan combined with infusional 5-FU and FA administered biweekly (FOLFIRI) has been shown to be more effective than when irinotecan is combined with bolus 5-FU (Fuchs et al. 2007). The regimen is considered one of the standard-of-care regimens in 2nd line mCRC (National Comprehensive Cancer Network [NCCN] Guidelines Version 2, 2016), and has also been shown to be effective in previously untreated patients (i.e., 1st line) (Bekaii-Saab and Wu 2014). The combination of FOLFIRI and cetuximab has been shown to be an active regimen in patients with *RAS*^{wt} mCRC (Heinemann et al. 2014, Venook et al. 2014). Adverse events most commonly associated with FOLFIRI are typically GI toxicity and myelosuppression with Grade 3-4 diarrhea and mucositis occurring in approximately 10% and 3% patients, respectively, and Grade 3-4 neutropenia occurring in approximately 20% of patients (Peeters et al. 2010).

2.9 Rationale for the Study

BRAF mutations are found in up to 20% of mCRC cases with lower rates of prevalence in patients in later lines of therapy. Patients with *BRAF*^{V600E} mCRC have a poor prognosis with shorter PFS and OS compared with patients with wild-type tumors (Van Cutsem et al. 2011,

Modest et al. 2012, Sorbye et al. 2015). There are no specific therapies targeting *BRAF*^{V600E} mCRC, and these patients are treated with combination chemotherapy with or without anti-EGFR agents.

Nonclinical experiments have provided compelling evidence that combining a BRAF inhibitor with an EGFR inhibitor results in greater anti-tumor effects than either agent alone in *BRAF*^{V600E} CRC cells (Corcoran et al. 2012, Prahallad et al. 2012, Yang et al. 2012). Consistent with these observations, the combination of the selective BRAF inhibitor, encorafenib and cetuximab resulted in a strong synergistic antitumor activity (Section 2.2.2).

There is convincing non-clinical evidence that robust inhibition of MAPK signaling is needed to more effectively treat *BRAF*-mutated tumors (Larkin et al. 2014, Long et al. 2015). This is consistent with the observations of the effects of the combination of binimetinib, encorafenib, and cetuximab, generated in human colon cancer xenograft model.

Early clinical data suggests that the combination of encorafenib and cetuximab is a well-tolerated regimen in patients with *BRAF*^{V600E} mCRC with encouraging response rates which are durable (Section 2.2.4). Preliminary clinical data with other *BRAF* and MEK inhibitor combinations have also suggested greater activity of the triple combination of a MEK inhibitor, BRAF inhibitor, and an anti-EGFR antibody relative to the dual combination of a BRAF and EGFR inhibitor. This data also suggests that the combination of a MEK inhibitor and an EGFR inhibitor (without a BRAF inhibitor) is only minimally active and associated with increased toxicity in patients with *BRAF*^{V600E} mCRC (Van Cutsem et al. 2015).

The current study is designed in light of the promising non-clinical and early clinical data of the combination of binimetinib, encorafenib, and cetuximab, and of encorafenib and cetuximab in patients with *BRAF*^{V600E} mCRC who have a poor clinical outcome, and for whom no targeted therapeutic strategies are effective after failure of standard chemotherapeutic regimens.

2.9.1 Study Design

This is a prospective, randomized, open-label, multi-center, parallel group, 3-arm Phase 3 study in patients with previously-treated *BRAF*^{V600E} mCRC comparing the efficacy and safety of binimetinib + encorafenib + cetuximab (Triplet Arm), and encorafenib + cetuximab (Doublet Arm) to Control Arm (irinotecan/cetuximab or FOLFIRI/cetuximab per Investigator's choice of control treatment), with a Safety Lead-in to assess the tolerability of binimetinib plus encorafenib plus cetuximab. The purpose of the study is to initially evaluate the safety/tolerability of the triplet combination in the Safety Lead-in and then to assess the efficacy of the triplet combination and the doublet combination relative to the control treatment in the randomized Phase 3 portion of the study.

Several lines of evidence suggest that *BRAF*^{V600E} mCRC is a distinct histological subtype associated with a unique clinical phenotype. The predominant BRAF mutations in CRC affect

V600E and result in a constitutively active BRAF protein. In the current study, patients may be enrolled using local laboratory assessment of *BRAF* status. All samples will also be tested by the central laboratory using the assay being developed as a companion diagnostic in the trial. Lack of $BRAF^{V600E}$ confirmation by the central laboratory may be due to discordance between the local assay and central laboratory results (potential false positive local assay results), or due to inadequate or poor sample condition for central testing (indeterminate results). If at any time there is lack of $BRAF^{V600E}$ confirmation by the central laboratory in a total of 37 patients (6% of the total planned enrollment of the randomized portion of the trial) or discordance between the local assay and the central laboratory in 18 patients (3% of the total planned enrollment), all subsequent patients will only be enrolled using central laboratory results. The actual discordance rate of 3% reflects the accuracy assessment observed between the central laboratory assay and an orthogonal PCR method, and minimizes the number of patients without $BRAF^{V600E}$ mCRC who would be exposed to study therapy.

The trial targets the population of patients whose disease has progressed on 1 or 2 prior regimens for mCRC. The number of 3rd-line patients (those having received 2 prior regimens) randomized to the Phase 3 portion of the study will be limited to 215 (35% of the total randomized) in order to ensure generalizability of the study. Patients with 2 prior regimens who have entered Screening at the time that the limit has been reached will be permitted to continue into the study if they are otherwise determined to be eligible. Permitting up to 35% of the randomized patients to have received 2 prior regimens ensures a sufficient sample size to adequately generalize the results across subgroups when incorporating other relevant stratification factors.

In order to allow for an early assessment of efficacy, an initial analysis for efficacy and futility will be conducted on the primary endpoints of OS (interim analysis) and ORR (primary analysis) of the Triplet Arm vs the Control Arm. While OS is considered the gold standard for demonstrating clinical benefit (Amir et al. 2012), ORR allows for a more rapid assessment of potential clinical benefit. Doublet vs Control OS is the key secondary endpoint of the study and will be tested in a hierarchical manner if the OS primary endpoint is observed to be statistically significant. Other secondary endpoints included in the testing hierarchy will be ORR of Doublet vs. Control, PFS of Triplet vs. Control, and PFS of Doublet vs. Control. In addition, a comparison of the OS in the Triplet Arm and the Doublet Arm will be a secondary endpoint in order to help demonstrate the contribution of binimetinib when added to the combination of encorafenib and cetuximab.

A number of therapeutic options are available following progression of initial therapy for mCRC. The combination of cetuximab plus irinotecan is one of the options recommended by the NCCN for patients who have previously received irinotecan- or oxaliplatin-based combination regimens and its use in this setting is consistent with current labeling of cetuximab. The use of FOLFIRI is consistent with European Society of Medical Oncology (ESMO) guidelines which recommend the use of cytotoxic doublets containing 5-FU with an EGFR inhibitor in patients with mCRC which is RAS^{wt} whose disease has progressed on 1 prior regimen (Van Cutsem et al. 2014). It has

been used in the control arm of several recent Phase 3 studies in the pure 2nd line setting in patients with mCRC (Peeters et al. 2014, Tabernero et al. 2015, Van Cutsem et al. 2015). The choice of whether to treat with FOLFIRI or irinotecan in the control arm will be at the discretion of the treating Investigator. In one of the few head-to-head comparisons of irinotecan and FOLFIRI found in the literature, mCRC patients without specific molecular characterization of their disease, the treatment groups did not differ significantly in overall quality of life (QoL) changes, response rate or PFS. The authors reported these findings to be consistent with a meta-analysis of 29 Phase 2 trials (Clarke et al. 2011).

2.9.2 Safety Lead-in

The safety of binimetinib/encorafenib (Section 2.5.2), cetuximab/encorafenib (Section 2.3.3.2), and binimetinib/panitumumab (Section 2.4.3.1.2) combinations have already been assessed in previous trials. Since the safety of the combination of binimetinib, encorafenib and cetuximab has not previously been evaluated, the current study proposes to expeditiously evaluate the tolerability of binimetinib/encorafenib/cetuximab in a Safety Lead-in phase to identify the dose to be used in the randomized Phase 3 portion of the study. The Safety Lead-in will be conducted at a limited number of sites where safety will be assessed by (approximately) weekly communication with Investigators to monitor AEs on a continuous basis. Dose-limiting toxicities will be evaluated. A DMC will determine tolerability after 9 and 15 patients have been treated at the starting doses of binimetinib 45 mg BID, encorafenib 300 mg QD and cetuximab 400 mg/m^2 followed by 250 mg/m^2 weekly. The DMC will also assess the tolerability at the end of the Safety Lead-in when a total of 25-30 patients have been treated. If it is determined that the MTD has been exceeded at any point during the Safety Lead-in, lower doses of binimetinib (30 mg BID), or encorafenib (225 mg QD) or both (depending on the observed toxicity), in combination with the same dose of cetuximab will be tested in a separate cohort. In considering whether it is appropriate to proceed at the recommended dose, the DMC will also have the opportunity to review toxicities observed over time, in cycles of treatment beyond the first cycle.

The proposed design for the run-in in conjunction with additional measures taken to ensure patient safety both during and after the Safety Lead-in will provide sufficient information to proceed with the randomized portion of the trial while maintaining patient safety within the trial.

2.9.2.1 Japanese Safety Lead-in

Prior studies in Japanese patients of binimetinib single agent (clinical trial CMEK162X1101) suggested that exposure was increased 1.5 to 2-fold in Japanese patients compared with non-Japanese patients. This increase in binimetinib exposure did not however appear to result in clinically meaningful differences in tolerability relative to non-Japanese patients. Encorafenib exposures, as assessed in clinical trials CLGX818X2101 and CLGX818X2103, appear to be similar in Japanese and non-Japanese patients. The safety and tolerability of the combination of encorafenib with cetuximab is expected to be consistent with the non-Japanese patients based on findings in clinical trial CLGX818X2103. The combination of binimetinib and encorafenib in

Japanese patients is currently being evaluated in an ongoing Phase 3 study (CMEK162B2301) and no significant differences in safety and tolerability have been observed between the Japanese and non-Japanese patients in that study.

In order to confirm the triplet dose is tolerable in Japanese patients, a separate Japanese Safety Lead-in cohort will be conducted in 6 patients. The starting dose in Japanese patients will be the dose assessed to be tolerable in the non-Japanese Safety Lead-in. The tolerability assessments will be based upon occurrence of DLTs as described in Section 4.2.1. As in the non-Japanese Safety Lead-in, the safety data will be reviewed by the DMC.

2.9.3 Dose Selection

2.9.3.1 Encorafenib/Cetuximab

In order to optimize the potential for benefit, while limiting the potential for toxicity, the starting encorafenib dose in the current study is 300 mg QD, corresponding to its single-agent RP2D.

As described in Section 2.3.3.2, the combination of encorafenib and cetuximab are currently being evaluated in patients with *BRAF*^{V600E} mCRC the Phase 1b/2 study CLGX818X2103. In the dose exploration of encorafenib and cetuximab, the MTD was not reached through the highest dose tested (encorafenib 450 mg QD plus cetuximab 400 mg/m^2 initial dose followed by 250 mg/m^2 weekly). In the dose exploration of the triple combination including encorafenib, cetuximab and the PI3K inhibitor alpelisib, although an MTD was not formally declared, review of safety and tolerability data suggested RP2D regimen of the triplet of encorafenib 200 mg QD, alpelisib 300 mg QD and standard doses of cetuximab. Although higher doses of encorafenib were tolerated in the doublet combination with cetuximab alone, the 200 mg QD encorafenib dose was chosen and is being evaluated in the Phase 2 portion of the study to maintain consistency between the dual combination and triple combination arms in order to isolate the effect of adding the PI3K inhibitor.

Given the available safety data up to 450 mg QD for encorafenib combined with standard doses of cetuximab, there are no clear safety concerns that would require initiating this study at a dose of 225 mg, approximating the Phase 2 dose which was chosen in Clinical Study CLGX818X2103 for practical reasons related to a specific scientific question rather than to maximize the benefit/risk of the doublet combination of encorafenib and cetuximab. It is not surprising that doses even higher than the single agent RP2D of 300 mg would be tolerated in combination with cetuximab as reactivation of the RAS/RAF/MEK pathway (i.e., RAS and CRAF), observed with single agent encorafenib, which is mediated by EGFR, is effectively modulated by the addition of cetuximab.

The proposed 300 mg starting dose represents an active dose of encorafenib that has been shown in clinical studies to lead to tumor regressions and clinical responses as a single agent (Dummer et al. 2013, Gomez-Roca et al. 2014). Nonclinical in vitro and in vivo studies using

BRAF^{V600E} human melanoma and colon cancer cell lines and xenograft models exploring the relationship between dose and efficacy also provide evidence in support of 300 mg being an appropriate starting dose in patients. Using the human melanoma xenograft model A375, which expresses *BRAF*^{V600E}, a 20 mg/kg dose was estimated to reduce tumor volume by 85% compared to untreated animals. Based on relative free fraction exposure, the 20 mg/kg steady-state mouse exposure is equivalent to the average exposure for patients receiving 300 mg QD dosing. This is also consistent with the observed inhibitory effects of encorafenib in combination with cetuximab using a *BRAF*^{V600E} CRC cell based system. Inhibition was 70% when studied at a free encorafenib concentration that was equivalent to the average daily concentration in patients receiving 300 mg QD dosing.

2.9.3.2 Binimetinib/Encorafenib/Cetuximab

The safety and tolerability of the relevant doublets of encorafenib plus cetuximab, binimetinib plus encorafenib, and binimetinib plus the EGFR inhibitor, panitumumab, have been previously evaluated. In these trials, all the agents were tolerated at or above their single agent MTD. These data are reviewed in detail in Section 2.2.4, Section 2.4.3.1.2 and Section 2.5.2.

The Safety Lead-in in this study will assess the safety/tolerability of binimetinib 45 mg BID and encorafenib 300 mg QD in combination with the standard weekly cetuximab regimen (400 mg/m^2 initial dose followed by 250 mg/m^2 weekly) for use in the randomized Phase 3 portion of the study. The binimetinib dose of 45 mg BID is below its single-agent MTD of 60 mg BID and represents the dose evaluated in the recently completed Phase 3 registrational trial in patients with advanced unresectable or metastatic *NRAS* mutation-positive melanoma (Clinical Study CMEK162A2301).

The combination of encorafenib and binimetinib was evaluated in Clinical Study CMEK162X2110 in which encorafenib 450 mg QD (in combination with binimetinib 45 mg BID) was identified as the RP2D (see Section 2.5.2) and is currently being evaluated in the ongoing Phase 3 trial (CMEK162XB2301) which has completed enrollment. The finding that encorafenib doses above the single agent RP2D are tolerated when combined with binimetinib is consistent with previous observations that MEK inhibition may ameliorate BRAF-inhibitor mediated toxicities (Menzies and Long 2014). As described below, when combined with standard doses of cetuximab, encorafenib doses up to 450 mg QD were tolerated without reaching MTD. As described in Section 2.4.3.1.2, Clinical Study CMEK162X2116 has demonstrated that binimetinib 45 mg BID plus the standard dose of panitumumab (6 mg/kg IV biweekly) was tolerated in patients with mCRC.

2.9.4 Summary

In summary, Phase 1/2 clinical data obtained to date have demonstrated that the combinations of encorafenib/cetuximab, encorafenib/binimetinib, and binimetinib/an EGFR-inhibitor have acceptable safety profiles. In addition, encouraging clinical activity has been observed when

MAPK signaling is partially inhibited with the combination of encorafenib and cetuximab in patients with previously treated *BRAF*^{V600E}-mutated mCRC. Preclinical data in-vivo models of CRC indicate the addition of a MEK inhibitor to the combination of a BRAF inhibitor and an EGFR-directed therapy could improve clinical activity and this has also been observed in preliminary clinical data sets.

3.0 STUDY OBJECTIVES AND ENDPOINTS

3.1 Study Objectives

3.1.1 Safety Lead-In

In patients with BRAFV600E mCRC:

3.1.1.1 Primary Objective

- Assess the safety/tolerability of the combination of encorafenib + binimetinib + cetuximab

3.1.1.2 Secondary Objectives

- Assess the activity of encorafenib + binimetinib + cetuximab as measured by blinded independent central review (BICR)-determined and Investigator-determined ORR, DOR, PFS and time to response

- Characterize the PK of encorafenib, cetuximab, binimetinib and the active metabolite of binimetinib (AR00426032)

3.1.1.3 Exploratory Objective

- Assess the activity of encorafenib + binimetinib + cetuximab as measured by OS

3.1.2 Randomized Phase 3

In patients with BRAFV600E mCRC:

3.1.2.1 Primary Objectives

- Compare the activity of encorafenib + binimetinib + cetuximab (Triplet Arm) vs. irinotecan/cetuximab or 5-FU/FA/irinotecan (FOLFIRI)/cetuximab (Control Arm) as measured by OS

- Compare the activity of encorafenib + binimetinib + cetuximab (Triplet Arm) vs. irinotecan/cetuximab or FOLFIRI/cetuximab (Control Arm) as measured by ORR per BICR

3.1.2.2 Key Secondary Objectives

- Compare the activity of encorafenib + cetuximab (Doublet Arm) vs. irinotecan/cetuximab or FOLFIRI/cetuximab (Control Arm) as measured by OS

3.1.2.3 Other Secondary Objectives

- Compare the Investigator-determined ORR of encorafenib + binimetinib + cetuximab (Triplet Arm) vs. irinotecan/cetuximab or FOLFIRI/cetuximab (Control Arm)
- Compare the BICR-determined and Investigator-determined ORR of encorafenib + cetuximab (Doublet Arm) vs. irinotecan/cetuximab or FOLFIRI/cetuximab (Control Arm)
- Compare the BICR-determined and Investigator-determined PFS of encorafenib + binimetinib + cetuximab (Triplet Arm) vs. irinotecan/cetuximab or FOLFIRI/cetuximab (Control Arm)
- Compare the BICR-determined and Investigator-determined PFS of encorafenib + cetuximab (Doublet Arm) vs. irinotecan/cetuximab or FOLFIRI/cetuximab (Control Arm)
- Compare the activity of Triplet Arm vs. Doublet Arm as measured by OS
- Compare the BICR-determined and Investigator-determined ORR of Triplet Arm vs. Doublet Arm
- Compare the BICR-determined and Investigator-determined PFS of Triplet Arm vs. Doublet Arm
- Compare BICR-determined and Investigator-determined DOR of Triplet Arm vs. Control Arm, of Doublet Arm vs. Control Arm and of Triplet Arm vs. Doublet Arm
- Compare BICR-determined and Investigator-determined time to response of Triplet Arm vs. Control Arm, of Doublet Arm vs. Control Arm and of Triplet Arm vs. Doublet Arm
- Assess the safety/tolerability of Triplet Arm, of Doublet Arm and of Control Arm
- Compare the effect on QoL of Triplet Arm vs. Control Arm, of Doublet Arm vs. Control Arm and of Triplet Arm vs. Doublet Arm
- Characterize the PK of encorafenib, cetuximab, binimetinib and the active metabolite of binimetinib (AR00426032)
- Assess for drug interactions between encorafenib, cetuximab, binimetinib and the active metabolite of binimetinib (AR00426032) based on PK modeling

3.1.2.4 Exploratory Objectives

- Assess the relationship between changes in tumor markers (carcinoembryonic antigen [CEA] and carbohydrate antigen 19-9 [CA19-9]) and radiographic response to treatment
- Assess blood- and tissue-based predictive biomarkers of activity

3.2 Study Endpoints

3.2.1 Safety Lead-In

3.2.1.1 Primary Endpoints

- Incidence of DLTs
- Incidence and severity of AEs, graded according to the NCI CTCAE, version 4.03 (v.4.03), and changes in clinical laboratory parameters, vital signs, ECGs, ECHO/MUGA scans and ophthalmic examinations
- Incidence of dose interruptions, dose modifications and discontinuations due to AEs

3.2.1.2 Secondary Endpoints

- ORR (by BICR and Investigator) per the Response Evaluation Criteria in Solid Tumors (RECIST), version 1.1 (v1.1), defined as the number of patients achieving an overall best response of complete response (CR) or PR divided by the total number of patients
- DOR (by BICR and Investigator), defined as the time from first radiographic evidence of response to the earliest documented disease progression or death due to underlying disease
- PFS (by BICR and Investigator), defined as the time from first dose to the earliest documented disease progression or death due to any cause
- Time to response (by BICR and Investigator), defined as the time from first dose to first radiographic evidence of response
- PK parameters of encorafenib, cetuximab, binimetinib and the active metabolite of binimetinib (AR00426032)

3.2.1.3 Exploratory Endpoint

- OS, defined as the time from first dose to death due to any cause

3.2.2 Randomized Phase 3

3.2.2.1 Primary Endpoints

- OS, defined as the time from randomization to death due to any cause, of Triplet Arm vs. Control Arm
- Confirmed ORR (by BICR) per RECIST, v1.1 of Triplet Arm vs. Control Arm

3.2.2.2 Key Secondary Endpoint

- OS of Doublet Arm vs. Control Arm

3.2.2.3 Other Secondary Endpoints

- Confirmed ORR (by Investigator) per RECIST, v1.1 of Triplet Arm vs. Control Arm
- Confirmed ORR (by BICR and Investigator) per RECIST, v1.1 of Doublet Arm vs. Control Arm
- PFS (by BICR and Investigator), defined as the time from randomization to the earliest documented disease progression or death due to any cause, of Triplet Arm vs. Control Arm
- PFS (by BICR and Investigator) of Doublet Arm vs. Control Arm
- OS of Triplet Arm vs. Doublet Arm
- Confirmed ORR (by BICR and Investigator) per RECIST, v1.1 of Triplet Arm vs. Doublet Arm
- PFS (by BICR and Investigator) of Triplet Arm vs. Doublet Arm
- DOR (by BICR and Investigator) of Triplet Arm vs. Control Arm, of Doublet Arm vs. Control Arm and of Triplet Arm vs. Doublet Arm
- Time to response (by BICR and Investigator), defined as the time from randomization to first radiographic evidence of response, of Triplet Arm vs. Control Arm, of Doublet Arm vs. Control Arm and of Triplet Arm vs. Doublet Arm
- Incidence and severity of AEs, graded according to NCI CTCAE, v.4.03, and changes in clinical laboratory parameters, vital signs, ECGs, ECHO/MUGA scans and ophthalmic examinations

- Change from baseline in the European Organization for Research and Treatment of Cancer (EORTC) Quality of Life Questionnaire for Cancer Patients (QLQ-C30), Functional Assessment of Cancer Therapy-Colon Cancer (FACT-C), EuroQol-5D-5L (EQ-5D-5L), and Patient Global Impression of Change (PGIC) of Triplet Arm vs. Control Arm, of Doublet Arm vs. Control Arm and of Triplet Arm vs. Doublet Arm

- Model-based PK parameters of encorafenib, cetuximab, binimetinib and the active metabolite of binimetinib (AR00426032)

- Model-based PK assessment of drug-drug interactions between encorafenib, cetuximab, binimetinib and the active metabolite of binimetinib (AR00426032)

3.2.2.4 Exploratory Endpoints

- Changes in CEA and CA19-9

- Genomic and proteomic analysis of blood and tissue samples at baseline and at end of treatment (optional for tumor samples at end of treatment).

4.0 STUDY DESIGN

4.1 Study Design Overview

This is a multicenter, randomized, open-label, 3-arm, Phase 3 study to evaluate encorafenib + cetuximab plus or minus binimetinib versus Investigator's choice of either irinotecan/cetuximab or FOLFIRI/cetuximab, as controls, in patients with *BRAF*^{V600E} mCRC whose disease has progressed after 1 or 2 prior regimens in the metastatic setting. The study contains a Safety Lead-in Phase in which the safety and tolerability of encorafenib + binimetinib + cetuximab will be assessed prior to the Phase 3 portion of the study.

BRAF Testing

Patients will be eligible for the study based on identification of a *BRAF*^{V600E} mutation in the tumor as determined by the central laboratory as part of Molecular Prescreening for the trial or by a local assay result obtained any time prior to Screening. Only polymerase chain reaction (PCR) and next generation sequencing (NGS)-based local assays results will be acceptable. If the patient is enrolled based on local assay results, the *BRAF* mutation status must be confirmed by the central laboratory no later than 30 days from first dose of study treatment.

In cases where there is discordance between the local assay and central laboratory results, or if the central laboratory is not able to confirm presence of a *BRAF*^{V600E} mutation due to inadequate or poor sample condition within 30 days of initiating study therapy, patients may only continue treatment if there is no clinical indication of deterioration or disease progression and the investigator determines that the patient is deriving benefit. In such instances, patients must be informed that the *BRAF* mutation status is unconfirmed and must sign a separate informed consent form (ICF) that includes this information and describes alternative treatment options.

Central laboratory *BRAF* mutation tests with a definitive result (positive or negative) cannot be repeated to resolve a discordant result. Patients whose sample is determined to be inadequate or who have an indeterminate result on central testing may have samples resubmitted for testing. Lack of *BRAF*^{V600E} confirmation by the central laboratory may be due to discordance between the local assay and central laboratory results (potential false positive local assay results), or due to inadequate or poor sample condition for central testing (indeterminate results). If at any time in the study there is lack of *BRAF*^{V600E} confirmation in a total of 37 patients (6% of the total planned enrollment of the randomized portion of the trial) or discordance between the local assay and the central laboratory in 18 patients (3% of the total planned enrollment), all subsequent patients will be required to have *BRAF*^{V600E} determined by the central laboratory for enrollment (i.e., local *BRAF* testing will no longer be accepted for trial eligibility). Information regarding sites and laboratories associated with discordant results will be maintained and results from laboratories with more than 1 prior discordant result will not be accepted for patient enrollment. Sites with more than 2 randomized patients having indeterminate results after initiation of

protocol version 6 will be required to enroll all subsequent patients based only on central laboratory assay results.

Molecular Prescreening

Prior to eligibility assessment for study randomization, patients may undergo molecular tumor prescreening with the central laboratory *BRAF* mutation assay at any time prior to Screening as long as they meet all the Molecular Prescreening inclusion/exclusion criteria. Note that tumor samples previously determined to be $BRAF^{wt}$ by local assessment may be submitted to the central laboratory. In particular, tumors with clinicopathological features of *BRAF* mutations, may be considered for testing by central laboratory regardless of the results of prior local *BRAF* mutation testing (see Section 7.1.1).

Safety Lead-in

The Safety Lead-in will be conducted at a limited number of sites. Dose-limiting toxicities will be evaluated (see Section 4.2.1) and the tolerability of the binimetinib, encorafenib, and cetuximab combination will be assessed (see Section 4.2.2) by the Sponsor and the Investigator in approximately weekly communications. The DMC will evaluate the safety data at pre-specified intervals and at additional points during the conduct of the Safety Lead-in, if necessary. The first 9 evaluable patients will be enrolled on a rolling basis in a single cohort to evaluate the combination of encorafenib 300 mg QD + binimetinib 45 mg BID + cetuximab 400 mg/m^2 followed by 250 mg/m^2 IV weekly. Additional patients will be enrolled based on assessments of the safety data by the DMC during the Safety Lead-in. The doses for the Triplet Arm in the randomized Phase 3 portion of the study will be determined after a total of 25-30 patients have been treated at the proposed doses and their data evaluated by the DMC (see Section 4.2).

In the Japanese Safety Lead-in portion of the study, a separate cohort of patients will be evaluated at a limited number of sites in Japan. Six patients will be enrolled on a rolling basis in a single cohort to evaluate the triplet dose. The starting dose in Japanese patients will be the dose assessed to be tolerable in the Safety Lead-in for non-Japanese patients. Similar to the Safety Lead-in for non-Japanese patients, the DMC will evaluate the safety data to confirm tolerability. The Phase 3 portion of the trial outside of Japan may be initiated before the completion of the Japanese Safety Lead-in (see Section 4.2.3).

Following study treatment discontinuation, patients who provide informed consent for survival follow-up will continue to be assessed to determine survival status until withdrawal of consent, patient is lost to follow-up, death or defined end of study (see Section 8.6.2.2).

Phase 3

Once the tolerability of the proposed doses for the Triplet Arm has been established, the Phase 3 portion of the study will begin and eligible patients will be randomized in a 1:1:1 ratio to the

Triplet Arm, Doublet Arm or Control Arm. The number of 3rd-line patients (those having received 2 prior regimens) will be limited to 215 (35% of the total randomized) after which only patients with 1 prior regimen will be randomized. Patients with 2 prior regimens who have entered Screening at the time that the limit has been reached will be permitted to continue into the study if they are otherwise determined to be eligible. Patients randomized to the Control Arm may be treated with either irinotecan + cetuximab or FOLFIRI + cetuximab as per Investigator's choice. The choice of irinotecan or FOLFIRI must be declared prior to randomization.

Randomization will be stratified by baseline Eastern Cooperative Oncology Group performance status (ECOG PS; 0 vs. 1), prior use of irinotecan (yes vs. no) and cetuximab source (US-licensed vs. EU-approved). The DMC will review the available safety information after the first 30 patients in the randomized Phase 3 portion of the study (i.e., approximately 10 patients in each arm) have had the opportunity to complete at least 1 cycle of treatment to confirm tolerability.

Treatment will be administered in 28-day cycles until disease progression, unacceptable toxicity, withdrawal of consent, initiation of subsequent anticancer therapy or death. In special circumstances (defined in Section 4.5), continuation of treatment beyond disease progression may be allowed. After treatment is discontinued, randomized Phase 3 patients will continue to be followed for OS unless consent for survival follow-up is withdrawn (see Section 8.6.2.3).

Upon implementation of protocol version 8, patients randomized to the Control arm who meet crossover eligibility criteria (Section 5.2.3) may cross over to receive the triplet regimen. Treatment in crossover patients will be administered in 28-day cycles until disease progression, unacceptable toxicity, withdrawal of consent, initiation of subsequent anticancer therapy or death. In special circumstances (defined in Section 4.5), continuation of treatment beyond disease progression may be allowed. After treatment is discontinued, patients will continue to be followed for OS unless consent for survival follow-up is withdrawn.

End of Study

End of study will be defined as the point when all patients have the opportunity to be followed for at least 1 year after the randomization date of the last patient enrolled **and** at least 80% of patients have an OS event (or are lost to follow-up). Any patients still receiving study drugs at the end of the study will be allowed to continue at the discretion of the Investigator and as long as none of the treatment discontinuation criteria are met (see Section 4.6). After the end of the study, access to study drugs will be provided in accordance with local regulations and requirements.

A schematic of the study design is presented in Figure 5.

Figure 5: Study Design

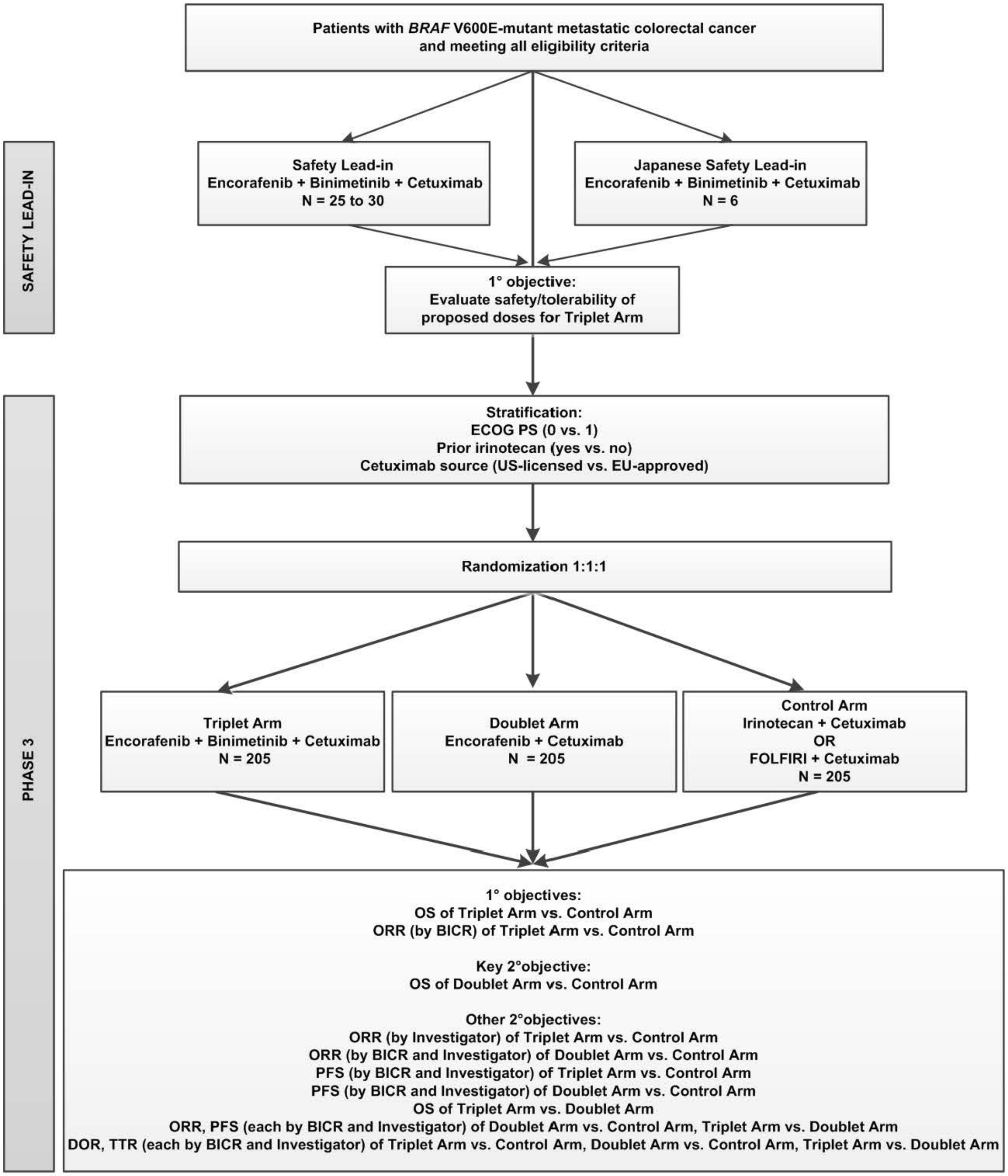

Note: Upon implementation of protocol version 8, patients randomized to the Control arm who meet crossover eligibility criteria (Section 5.2.3) may cross over to receive the triplet regimen.

4.2 Safety Lead-in Phase

The Safety Lead-in portion of the study will evaluate the safety and tolerability of the combination of encorafenib + binimetinib + cetuximab for 1 cycle (the first 28 days of treatment).

4.2.1 Dose-Limiting Toxicities

For purposes of tolerability decisions, a DLT is defined as any AE or abnormal laboratory value assessed as unrelated to disease, disease progression, intercurrent illness or concomitant medications/therapies occurring within the first 28 days of treatment that satisfies at least 1 of the criteria listed in Table 8.

Table 8: Criteria for Defining Dose-limiting Toxicities

**DLT Criteria**
• Any AE or laboratory value considered unrelated to underlying disease, disease progression, intercurrent illness or concomitant medications/therapies resulting in the inability to tolerate at least 75% dose intensity [(administered dose in mg/planned dose in mg) x 100] of binimetinib, encorafenib or cetuximab during Cycle 1
**Cardiac disorders**<br>• Absolute decrease of LVEF > 10% compared to Baseline and the LVEF is below the institution's LLN<br>• Left ventricular systolic dysfunction Grade ≥ 3<br>• Other cardiac disorders Grade ≥ 3
**Vascular disorders**<br>• Grade 3 hypertension for > 14 consecutive days<br>• Grade 4 hypertension
**General disorders and administration site conditions**<br>• Fatigue Grade 3 for > 14 consecutive days
**Respiratory disorders**<br>• Interstitial lung disease/pneumonitis Grade ≥ 2
**Skin and subcutaneous tissue disorders**[a]<br>• Rash, hand foot skin reaction (HFSR), or photosensitivity CTCAE Grade 3 for > 14 consecutive days despite maximal skin toxicity treatment (as per local practice)<br>• Rash, HFSR, or photosensitivity CTCAE Grade 4
**Gastrointestinal disorders**[a]<br>• Diarrhea Grade 3 for ≥ 48 hours despite optimal use of antidiarrheal therapy<br>• Diarrhea Grade 4<br>• Nausea/vomiting Grade 3 for ≥ 48 hours despite optimal use of antiemetic therapy<br>• Nausea/vomiting Grade 4

DLT Criteria
**Investigations**<br>• Total bilirubin Grade ≥ 3<br>• AST or ALT Grade ≥ 3 in conjunction with total bilirubin Grade ≥ 2 of any duration<br>• AST or ALT Grade 3 for > 7 consecutive days<br>• AST or ALT Grade 4<br>• Serum creatinine Grade ≥ 3<br>• CK elevation ≥ Grade 3 associated with an increase in creatinine ≥ 1.5 × the patient's Baseline screening creatinine<br>• ANC Grade 4 for > 7 consecutive days<br>• Platelet count Grade 3 with signs of clinically significant bleeding<br>• Platelet count Grade 4<br>• ECG QTcF prolonged ≥ Grade 3[b]
**Eye disorders-Retinal**<br>• Retinopathy or retinal detachment Grade ≥ 3, confirmed by ophthalmic examination<br>• Retinal vascular disorder including retinal vein occlusion (RVO), confirmed by ophthalmic examination
**Eye disorders –Visual disturbances without ocular (retinal) changes**<br>• Blurred vision, flashing lights, floaters: Grade ≥ 3
**Eye disorders – (other specify)**<br>• Grade ≥ 3 for > 21 consecutive days<br>• Grade 4 confirmed by ophthalmic examination
**Other hematologic and nonhematologic toxicities[c]**<br>• Any other Grade ≥ 3 AE except:<br>o Lymphocyte count decreased (lymphopenia) Grade ≥ 3 unless clinically significant

Abbreviations: AE = adverse event; ALT = alanine aminotransferase; ANC = absolute neutrophil count; AST = aspartate aminotransferase; CK = creatine kinase; DLT = dose-limiting toxicities; ECG = electrocardiogram; LLN = lower limit of normal; LVEF = left ventricular ejection fraction; QTcF = QT interval corrected for heart rate using Fridericia's formula.

[a] Prophylactic treatment for nausea/vomiting or skin AEs is not required with binimetinib. However, antiemetics and treatments for skin AEs should be used at the discretion of the Investigator if the patient experiences nausea/vomiting and/or skin AEs Grade ≥ 1.

[b] QTc must be prolonged on two separate ECGs.

[c] Isolated laboratory changes (e.g. alkaline phosphatase, cholesterol, lipase, serum amylase) or those due to sampling or laboratory errors without associated clinical signs or symptoms may be determined to not be DLTs upon review and agreement by the Investigator and Sponsor's Medical Monitor.

4.2.2 Tolerability Assessment

For the Safety Lead-in, the first 9 patients will be enrolled on a rolling basis in a single cohort to evaluate the combination of encorafenib 300 mg QD + binimetinib 45 mg BID + cetuximab

400 mg/m^2 followed by 250 mg/m^2 weekly (see Figure 6). The tolerability assessment will be based upon occurrence of DLTs (see Section 4.2.1) in all patients. Enrollment will be put on hold if 3 patients experience DLTs until a discussion with the DMC can occur. If it is determined that the MTD has been exceeded, dosing at the current dose will be suspended and the next 9 patients will be enrolled on a rolling basis in a separate cohort to test lower doses of binimetinib (30 mg BID) or encorafenib (225 mg QD), corresponding to the doses of binimetinib, and encorafenib in Dose Level -1 in Table 10, in combination with the standard cetuximab dose. It is possible that doses of binimetinib, encorafenib, or both may be reduced depending on the observed pattern of toxicity and based on the known AE profiles of both drugs.

If DLTs are observed in fewer than 3 of 9 evaluable patients, the DMC will review data after the 9th patient has been followed for at least one 28-day cycle to confirm tolerability of the doses. As the initial 9 patients will be enrolled over time, the DMC will have available information beyond the first 28 days of treatment on several patients, and this information will also be reviewed and incorporated into decisions regarding tolerability of the regimen. If the DMC determines the doses to be tolerable in the first 9 evaluable patients based on observing DLTs in < 33% of patients and evaluation of the overall toxicity profile, the Safety Lead-in will be expanded by at least an additional 16 up to a maximum of 21 patients to account for patients at various stages of screening at the time.

Once the first 6 evaluable patients in this Safety Lead-in expansion have been treated for at least 1 cycle, the DMC will again review all the available data. The overall safety profile of the evaluable 15 patients, including data from all cycles of treatment, will be considered in the evaluation.

At the completion of the Safety Lead-in when all 25-30 patients (the initial 9 plus the additional 16-21 patients in the expansion) have been followed for a minimum of 1 cycle, the DMC will again review all accumulated safety data to confirm that the doses are acceptable for use in the Triplet Arm (encorafenib + binimetinib + cetuximab) of the randomized Phase 3 portion of the study. Following their review of the cumulative data after the first 15 patients treated and / or at the end of the Safety Lead-in, the DMC will either confirm the tolerability of the current dose or may recommend de-escalation.

In the event that the initial doses are not tolerated and Dose Level -1 is evaluated, it will be conducted as described above with the DMC reviewing the data after the first 9 patients, during the Safety Lead-in expansion (first 15 patients), and at the end of the Safety Lead-in (25-30 patients) (see Figure 6). Inclusion of the Triplet Arm in the Phase 3 portion of the trial will be reconsidered if it is determined that Dose Level -1 is not tolerable.

Patients who require a dose interruption or reduction during the initial 28-day treatment period (Cycle 1) will remain evaluable for tolerability decisions if the reason for the reduction and/or interruption represents a DLT. Patients will be replaced if they have received less than 75% dose intensity [(administered dose in mg/planned dose in mg) x 100] of binimetinib, encorafenib or

cetuximab for any reason other than an AE or abnormal laboratory value that is not related to disease, disease progression, intercurrent illness or concomitant medications/therapies before completing Cycle 1. The DMC will review all accumulated safety data after all Safety Lead-in patients complete 1 cycle of treatment.

If an acceptable combination dose cannot be identified, following discussions with the DMC and the study Steering Committee, the protocol may be amended to remove the Triplet Arm and allow comparison of the Doublet Arm to the Control Arm with the appropriate modifications to study conduct, endpoints and analysis. If the study is conducted under a Special Protocol Assessment (SPA), any amendment must be approved by the FDA.

Figure 6: Safety Lead-in Schema

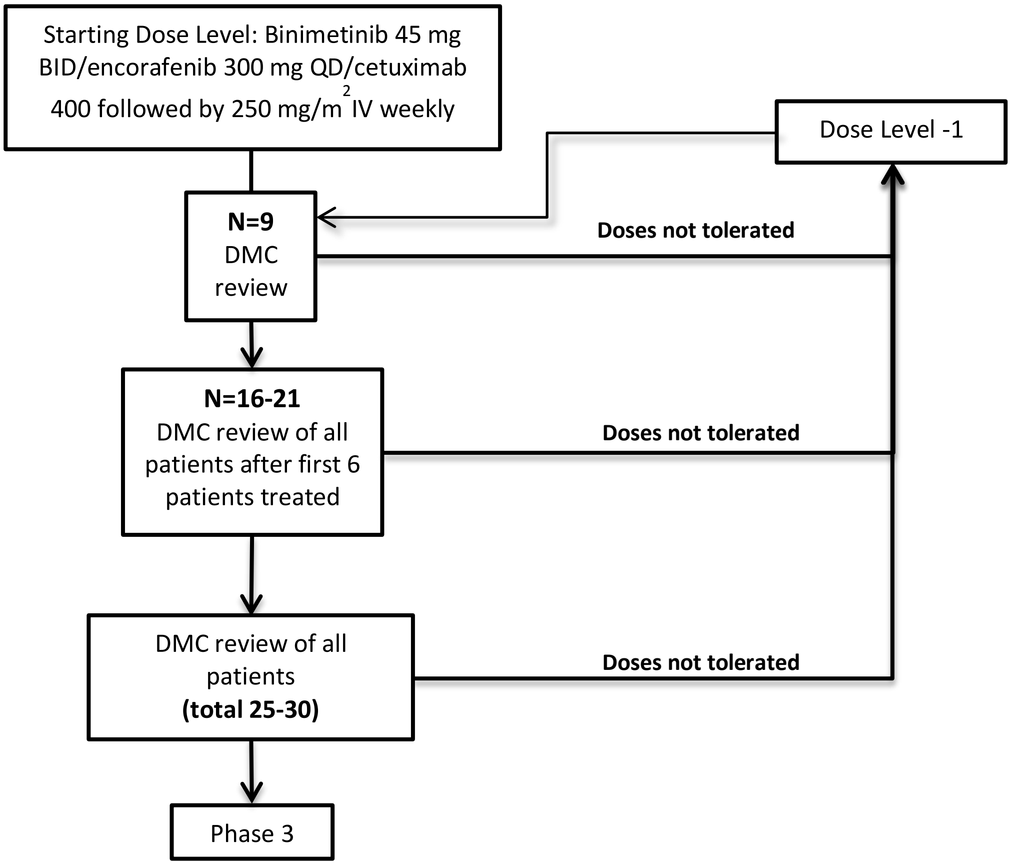

4.2.3 Japanese Safety Lead-in

The first 6 patients will be enrolled on a rolling basis in a single cohort to evaluate the combination of encorafenib + binimetinib + cetuximab at doses which are being evaluated in the non-Japanese Safety Lead-in Expansion at the time the Japanese Safety Lead-in is initiated or at the Phase 3 doses if established in non-Japanese patients. During Cycle 1, patients must be

hospitalized for the first 2 weeks of treatment. If the treating physician determines the patient is tolerating therapy well, the patient may be treated as an outpatient for the remainder of the cycle. If multiple patients are ready to start treatment in the Japanese Safety Lead-in, then initiation of study therapy for each patient (i.e., Cycle 1 Day 1) must be separated by at least 24 hours. The tolerability assessment will be based upon occurrence of DLTs (see Section 4.2.1) in all patients. Enrollment will be put on hold if 2 patients experience DLTs until a discussion with the DMC can occur. If it is determined that the MTD has been exceeded, dosing at the current dose will be suspended and the next 6 patients will be enrolled on a rolling basis in a separate cohort to test lower doses of binimetinib or encorafenib, in combination with the standard cetuximab dose. It is possible that doses of binimetinib, encorafenib, or both may be reduced depending on the observed pattern of toxicity and based on the known AE profiles of both drugs.

If DLTs are observed in fewer than 2 of the 6 evaluable patients, the DMC will review data after the 6th patient has been followed for at least one 28-day cycle to confirm tolerability of the doses (the DMC review may also be conducted after the 5th patient has been followed for at least one 28-day cycle if no DLTs have been observed). Patients who require a dose interruption or reduction during the initial 28-day treatment period (Cycle 1) will remain evaluable for tolerability decisions if the reason for the reduction and/or interruption represents a DLT. Patients will be replaced if they have received less than 75% dose intensity [(administered dose in mg/planned dose in mg) × 100] of binimetinib, encorafenib or cetuximab for any reason other than an AE or abnormal laboratory value that is not related to disease, disease progression, intercurrent illness or concomitant medications/therapies before completing Cycle 1.

As the initial 6 patients will be enrolled over time, the DMC will have available information beyond the first 28 days of treatment on several patients, and this information will also be reviewed and incorporated into decisions regarding tolerability of the regimen. If the DMC determines the doses to be tolerable in the first 6 evaluable patients based on observing DLTs in < 33% of patients and evaluation of the overall toxicity profile in Japanese patients, the Japanese Safety Lead-in will be considered complete.

4.3 Randomized Phase 3

A total of 615 eligible patients with *BRAF*^{V600E} mutant mCRC who have progressed on 1 or 2 prior metastatic regimens will be randomized 1:1:1 to the 3 treatment arms (Triplet, Doublet or Control). The number of 3rd-line patients (those having received 2 prior regimens) will be limited to 215 after which only patients with 1 prior regimen will be randomized. Patients with 2 prior regimens who have entered Screening at the time that the limit has been reached will be permitted to continue into the study if they are otherwise determined to be eligible. Patients randomized to the Control Arm may be treated with irinotecan + cetuximab or FOLFIRI + cetuximab as per Investigator’s choice. The choice of irinotecan or FOLFIRI must be declared prior to randomization.

Randomization will be stratified by baseline ECOG PS (0 vs. 1), prior use of irinotecan (yes vs. no) and cetuximab source (US-licensed vs. EU-approved). The DMC will review all available safety information after the first 30 patients in the randomized Phase 3 portion of the study (i.e., approximately 10 patients in each arm) have had the opportunity to complete at least 1 cycle of treatment to confirm tolerability. During the remainder of the study, the DMC will review safety data at regular intervals.

An initial analysis of the study will be performed when all three of the following criteria have been met:

- approximately 9 months after randomization of the 330th patient (i.e., approximately 110 patients per arm), to allow a majority of responders among the 330 Phase 3 patients to have the opportunity to be followed for approximately 6 months or longer after their first response
- at least 188 OS events have occurred in the Triplet and Control arms combined (i.e., approximately 70% information)
- at least 169 OS events have occurred in the Doublet and Control arms combined (i.e., approximately 50% information)

The primary analysis of Triplet vs. Control ORR by BICR will occur at this time and will be based on the first 330 randomized patients. An interim analysis for superiority or (non-binding) futility of the Triplet vs Control OS endpoint will also be performed at the time of the primary ORR analysis based on all available data (Section 11.3.9). The DMC will perform the analyses for both of the comparisons at this time point. If the interim analysis for OS of Triplet vs. Control exceeds the superiority boundary, patients in the Triplet and Doublet arms may continue to be followed for a more mature comparison.

If the OS interim analysis results do not cross the superiority boundary, the OS final analysis will occur once at least 268 events are observed in the Triplet Arm + Control Arm and at least 338 events are observed in the Doublet Arm + Control Arm.

4.4 Measures to Minimize Bias

This is an open-label study, so the following steps will be taken during the randomized Phase 3 portion of the study to minimize any possible bias:

- The randomization schedule will be created and managed by a third-party vendor, and treatments will be assigned according to a computerized central randomization list using an interactive web response system (IWRS) (see Section 6.2).
- The Sponsor (and their designee trial team) will be blinded to treatment group assignments presented in aggregate data summaries.

- A limited number of study team members will be unblinded to individual treatment assignments for purposes of study conduct (e.g., site monitoring, data management, patient emergencies or for regulatory reporting purposes). The names and roles of these team members will be documented by the Sponsor, and they will not have access to unblinded aggregate summaries.

- Patients with $BRAF^{wt}$ tumors as determined by a local laboratory test are not excluded from molecular prescreening or screening. Screening using the central laboratory test is encouraged particularly where clinicopathological features are consistent with $BRAF^{V600E}$ mutation.

These steps will remain in place until a database lock supporting a clinical study report occurs. The Sponsor will remain blinded to aggregate OS results until the Triplet vs Control OS endpoint exceeds the superiority boundary or the study stops for futility.

4.5 Duration of Treatment

Patients may continue receiving study drug as long as none of the treatment discontinuation criteria are met (see Section 9.2). Continuing study treatment beyond disease progression for any patient is only to be considered under special circumstances when it is believed that the patient may clinically benefit from continued treatment beyond progression. If it is judged by the Investigator in consultation with the Sponsor, to be in the best interest of the patient, the patient may remain on study treatment as long the patient continues to benefit from the study treatment per Investigator assessment. Special circumstances can be defined by e.g., mixed responses and appearance of new brain metastases (only) which is treatable with stereotactic radiotherapy or surgery but does not require whole brain radiotherapy.

Dosing beyond progression is not allowed in the following cases:

- Patients with clear evidence of disease progression at multiple sites or clear evidence of new lesions outside the central nervous system (CNS)

- Patients with rapid progression of disease at critical anatomical sites (e.g., cord compression) requiring urgent alternative medical intervention cannot be dosed beyond progression

- Patients who have clinically relevant worsening of laboratory values

- Patients who have a clinically significant decline in performance status at time of progression.

After patients have discontinued treatment for any reason, they will be followed every 3 months, or more frequently as needed, until withdrawal of consent, the patient is lost to follow-up, death or defined end of study (Section 4.6). If patients withdraw consent, they will be asked if they are

willing to be contacted via telephone for survival status. If the patient refuses to be contacted (or, for patients in the Safety Lead-in, where informed consent cannot be obtained due to the patient being lost to follow-up or previous withdrawal of consent), attempts to determine survival status should be made via access to public records where permitted by local laws.

4.6 End of Study

End of study will be defined as the point when all patients have the opportunity to be followed for at least 1 year after the randomization date of the last patient enrolled **and** at least 80% of patients have an OS event (or are lost to follow-up). Any patients still receiving study drugs at the end of the study will be allowed to continue at the discretion of the Investigator and as long as none of the treatment discontinuation criteria are met (see Section 9.2). The Sponsor will notify all applicable regulatory agencies in accordance with local requirements when the study has ended. After the end of the study, access to study drugs will be provided only in accordance with local regulations and requirements.

4.7 Data Review Committees

4.7.1 Data Monitoring Committee

The DMC will be responsible for reviewing safety data in the non-Japanese and Japanese Safety Lead-in portions of the study in order to evaluate the safety and tolerability of the combination of encorafenib + binimetinib + cetuximab. Once the Phase 3 portion has been initiated, the DMC will confirm the safety and tolerability of the triple combination after the first 30 patients have been randomized and then will be responsible for reviewing safety data at regular intervals as well as performing the efficacy analyses for Triplet vs Control OS (interim analysis for superiority and [non-binding] futility) and Triplet vs Control ORR (primary analysis) (Section 11.3.9). A DMC Charter that outlines DMC membership, precisely the data that will be reviewed and the timing and frequency of the reviews will be established. DMC recommendations will be provided to the Sponsor in compliance with the DMC Charter.

4.7.2 Steering Committee

The Steering Committee (SC) will be appointed by the Sponsor prior to the initiation of the study. The SC will include Investigators participating in the study, leading experts in colorectal cancer and Sponsor representatives. The SC will be involved in the oversight of the study and will ensure transparent management of the study according to the protocol. Details on the role of the SC and working procedures will be defined in the SC Charter.

5.0 PATIENT POPULATION

5.1 Number of Patients

Approximately 646 to 651 patients at approximately 300 study centers are planned (includes 31 to 36 patients for the non-Japanese and Japanese Safety Lead-in study portions; additional patients may be included if lower dose levels are evaluated during the Safety Lead-in).

5.2 Selection of Patients

The eligibility criteria described in this study protocol are designed to identify patients for whom study treatment is considered appropriate. All relevant medical conditions should be considered when deciding whether a patient is suitable for enrollment in the study.

Eligibility will be determined separately for molecular tumor testing during a Prescreening Phase and for enrollment/randomization in the study during a Screening Phase. Questions regarding patient eligibility should be addressed to the Sponsor or delegate prior to enrollment/randomization. Patients will be considered enrolled in the study once they have signed the Screening ICF.

5.2.1 Eligibility Criteria for Molecular Prescreening

Patients may undergo molecular tumor prescreening with the central laboratory *BRAF* mutation assay at any time prior to Screening as long as they meet all the Molecular Prescreening inclusion/exclusion criteria. Note that tumor samples previously determined to be $BRAF^{wt}$ by local assessment may be submitted to the central laboratory. In particular, tumors with clinicopathological features of *BRAF* mutations such as right colon tumors, poorly differentiated, mucinous, or signet-ring carcinomas, or tumors metastasized to the peritoneum (Yokota et al. 2011), may be considered for testing by central laboratory regardless of the results of prior local BRAF mutation testing (see Section 7.1.1).

5.2.1.1 Inclusion Criteria for Molecular Prescreening

All the following inclusion criteria at Prescreening must be met for a patient to be eligible to undergo molecular tumor prescreen:

1. Provide a signed and dated Prescreening informed consent document
2. Age ≥ 18 years at time of informed consent
3. Histologically- or cytologically-confirmed CRC that is metastatic
4. Eligible to receive cetuximab per locally approved label with regard to tumor *RAS* status

5. Able to provide a sufficient amount of representative tumor specimen (primary or metastatic, archival or newly obtained) for central laboratory testing of *BRAF* and *KRAS* mutation status (minimum of 6 slides; optimally up to 15 slides).

5.2.1.2 Exclusion Criteria for Molecular Prescreening

Patients meeting any of the following criteria at Prescreening are not eligible to undergo molecular tumor prescreen:

1. Leptomeningeal disease
2. History or current evidence of RVO or current risk factors for RVO (e.g., uncontrolled glaucoma or ocular hypertension, history of hyperviscosity or hypercoagulability syndromes)
3. Known history of acute or chronic pancreatitis
4. History of chronic inflammatory bowel disease or Crohn’s disease requiring medical intervention (immunomodulatory or immunosuppressive medications or surgery) ≤ 12 months prior to randomization
5. Concurrent neuromuscular disorder that is associated with the potential of elevated CK (e.g., inflammatory myopathies, muscular dystrophy, amyotrophic lateral sclerosis, spinal muscular atrophy)
6. Known history of human immunodeficiency virus (HIV) infection
7. Known history of Gilbert's syndrome or is known to have any of the following genotypes: UGT1A1*6/*6, UGT1A1*28/*28, or UGT1A1*6/*28
8. Known contraindication to receive cetuximab or irinotecan at the planned doses; refer to the most recent cetuximab and irinotecan summary of product characteristics (SPC) or local label as applicable
9. Prior anti-EGFR treatment
10. More than 2 prior regimens in the metastatic setting
    Notes:
    a. Disease relapse during treatment or within 6 months following adjuvant therapy will be considered metastatic disease.
    b. Maintenance therapy given in the metastatic setting will not be considered a separate regimen.

5.2.2 Patient Eligibility

Patients must fulfill all of the following inclusion criteria and none of the exclusion criteria to be included in this study.

5.2.2.1 Inclusion Criteria

All the following inclusion criteria must be met for a patient to be eligible to be included in this study:

1. Provide a signed and dated Screening informed consent document
2. Age ≥ 18 years at time of informed consent
3. Histologically- or cytologically-confirmed CRC that is metastatic
4. Presence of BRAFV600E in tumor tissue as previously determined by a local assay at any time prior to Screening **or** by the central laboratory (see Section 7.1.1)
   Notes:
   a. Only PCR and NGS-based local assays results will be acceptable
   b. If at any time in the Phase 3 portion of the study there is lack of *BRAF*^{V600E} confirmation by the central laboratory (for any reason including discordance and inadequate available tissue) in 37 total patients or discordance (a valid result of "no *BRAF*^{V600E} mutation" as determined by the central laboratory) between the local assay and the central laboratory in 18 patients, all subsequent patients will be required to have *BRAF*^{V600E} determined by the central laboratory prior to enrollment (see Section 7.1.1).
   c. Central testing cannot be repeated to resolve discordances with a local result once the central laboratory delivers a definitive result (positive or negative).
   d. If the result from the central laboratory is indeterminate or the sample is deemed is inadequate for testing, additional samples may be submitted.
   e. Results from local laboratories with more than 1 discordant result leading to patient enrollment will not be accepted for further patient enrollment.
   f. Sites with more than 2 randomized patients having indeterminate results after initiation of protocol version 6 will be required to enroll all subsequent patients based only on central laboratory assay results.

5. Able to provide a sufficient amount of representative tumor specimen (primary or metastatic, archival or newly obtained) for confirmatory central laboratory testing of *BRAF* and *KRAS* mutation status (minimum of 6 slides; optimally up to 15 slides)
Note: For patients enrolled on the basis of a local *BRAF* mutation assay, tumor samples must be submitted to the central laboratory for *BRAF* testing as soon as possible following the signing of the Molecular Prescreening informed consent. The *BRAF* status must be confirmed no later than 30 days following first dose of study drug. If the central laboratory determines that the sample is inadequate for analysis, a second sample may be submitted (see Section 7.1.1).

6. Eligible to receive cetuximab per locally approved label with regard to tumor *RAS* status

7. Progression of disease after 1 or 2 prior regimens in the metastatic setting.
Notes:
    a. Disease relapse during treatment or within 6 months following adjuvant therapy will be considered metastatic disease.
    b. Patients who have received 2 prior regimens (i.e., those entering the study in the 3rd line setting), must have received or have been offered and refused prior oxaliplatin unless it was contraindicated due to underlying conditions.
    c. Maintenance therapy given in the metastatic setting will not be considered a separate regimen.
    d. In the Phase 3 portion of study, the number of patients having received 2 prior regimens will be limited to 215 (35% of the total randomized). Patients with 2 prior regimens who have entered Screening at the time that the limit has been reached will be permitted to continue into the study if they are otherwise determined to be eligible.

8. Evidence of measurable or evaluable non-measurable disease per RECIST, v1.1

9. ECOG PS of 0 or 1

10. Adequate bone marrow function characterized by the following at screening:
    a. Absolute neutrophil count (ANC) $\geq 1.5 \times 10^9$/L;
    b. Platelets $\geq 100 \times 10^9$/L;
    c. Hemoglobin ≥ 9.0 g/dL.
    Note: Transfusions will be allowed to achieve this. Transfusions will be permitted provided the patient has not received more than 2 units red blood cells in the prior 4 weeks to achieve this criteria.

11. Adequate renal function characterized by serum creatinine ≤ 1.5 × upper limit of normal (ULN), or calculated by Cockroft-Gault formula, or directly measured creatinine clearance ≥ 50 mL/min at screening

12. Adequate electrolytes at Baseline, defined as serum potassium and magnesium levels within institutional normal limits (Note: replacement treatment to achieve adequate electrolytes will be allowed).

13. Adequate hepatic function characterized by the following at screening:

    a. Serum total bilirubin ≤ 1.5 × ULN and < 2 mg/dL
    Note: Patients who have a total bilirubin level > 1.5 × ULN will be allowed if their indirect bilirubin level is ≤ 1.5 × ULN.

    b. Alanine aminotransferase (ALT) and/or aspartate aminotransferase (AST) ≤ 2.5 × ULN, or ≤ 5 × ULN in presence of liver metastases

14. Adequate cardiac function characterized by the following at screening:

    a. Left ventricular ejection fraction (LVEF) ≥ 50% as determined by a MUGA scan or ECHO;

    b. Mean triplicate QT interval corrected for heart rate using Fridericia's formula (QTcF) value ≤480 msec

15. Able to take oral medications

16. Willing and able to comply with scheduled visits, treatment plan, laboratory tests and other study procedures

17. Female patients are either postmenopausal for at least 1 year, are surgically sterile for at least 6 weeks, or must agree to take appropriate precautions to avoid pregnancy from screening through follow-up if of childbearing potential
    Note: Permitted contraceptive methods listed in Section 5.3.1 should be communicated to the patients and their understanding confirmed. For all females, the pregnancy test result must be negative at screening.

18. Males must agree to take appropriate precautions to avoid fathering a child from screening through 90 days following end of therapy.
    Note: Permitted contraceptive methods listed in Section 5.3.1 should be communicated to the patients and their understanding confirmed.

19. Patients under guardianship or partial guardianship will be eligible unless prohibited by local laws or by local/central ethic committees (e.g., France, Germany). Where allowed, all procedures prescribed by law must be followed.

5.2.2.2 Exclusion Criteria

Patients meeting any of the following criteria at screening will not be included in the study:

1. Prior treatment with any RAF inhibitor, MEK inhibitor, cetuximab, panitumumab or other EGFR inhibitors
2. Prior irinotecan hypersensitivity or toxicity that would suggest an inability to tolerate irinotecan 180 mg/m^2 every 2 weeks
3. Symptomatic brain metastasis
   Notes: Patients previously treated or untreated for this condition who are asymptomatic in the absence of corticosteroid and anti-epileptic therapy are allowed. Brain metastases must be stable for ≥ 4 weeks, with imaging (e.g., magnetic resonance imaging [MRI] or computed tomography [CT]) demonstrating no current evidence of progressive brain metastases at screening.
4. Leptomeningeal disease
5. History or current evidence of RVO or current risk factors for RVO (e.g., uncontrolled glaucoma or ocular hypertension, history of hyperviscosity or hypercoagulability syndromes)
6. Use of any herbal medications/supplements or any medications or foods that are strong inhibitors or inducers of cytochrome P450 (CYP) 3A4/5 ≤ 1 week prior to the start of study treatment
7. Known history of acute or chronic pancreatitis
8. History of chronic inflammatory bowel disease or Crohn’s disease requiring medical intervention (immunomodulatory or immunosuppressive medications or surgery) ≤ 12 months prior to randomization
9. Impaired cardiovascular function or clinically significant cardiovascular diseases, including any of the following:
   a. History of acute myocardial infarction, acute coronary syndromes (including unstable angina, coronary artery bypass graft [CABG], coronary angioplasty or stenting) ≤ 6 months prior to start of study treatment;
   b. Symptomatic congestive heart failure (i.e., Grade 2 or higher), history or current evidence of clinically significant cardiac arrhythmia and/or conduction abnormality ≤ 6 months prior to start of study treatment, except atrial fibrillation and paroxysmal supraventricular tachycardia.

10. Uncontrolled hypertension defined as persistent elevation of systolic blood pressure ≥ 150 mmHg or diastolic blood pressure ≥ 100 mmHg despite current therapy

11. Impaired hepatic function, defined as Child-Pugh class B or C

12. Impaired GI function or disease that may significantly alter the absorption of encorafenib or binimetinib (e.g., ulcerative diseases, uncontrolled vomiting, malabsorption syndrome, small bowel resection with decreased intestinal absorption)

13. Concurrent or previous other malignancy within 5 years of study entry, except cured basal or squamous cell skin cancer, superficial bladder cancer, prostate intraepithelial neoplasm, carcinoma in-situ of the cervix, or other noninvasive or indolent malignancy without Sponsor approval

14. History of thromboembolic or cerebrovascular events ≤ 6 months prior to starting study treatment, including transient ischemic attacks, cerebrovascular accidents, deep vein thrombosis or pulmonary emboli

15. Concurrent neuromuscular disorder that is associated with the potential of elevated CK (e.g., inflammatory myopathies, muscular dystrophy, amyotrophic lateral sclerosis, spinal muscular atrophy)

16. Treatment with any of the following:

    a. Cyclical chemotherapy within a period of time that was shorter than the cycle length used for that treatment (e.g., 6 weeks for nitrosourea, mitomycin-C) prior to starting study treatment

    b. Biologic therapy (e.g., antibodies) except bevacizumab or aflibercept, continuous or intermittent small molecule therapeutics, or any other investigational agents within a period of time that is ≤ 5 half-lives ($t_{1/2}$) or ≤ 4 weeks (whichever is shorter) prior to starting study treatment

    c. Bevacizumab or aflibercept therapy ≤ 3 weeks prior to starting study treatment

    d. Radiation therapy that included > 30% of the bone marrow

17. Residual CTCAE ≥ Grade 2 toxicity from any prior anticancer therapy, with the exception of Grade 2 alopecia or Grade 2 neuropathy

18. Known history of HIV infection

19. Active hepatitis B or hepatitis C infection

20. Known history of Gilbert's syndrome or is known to have any of the following genotypes: UGT1A1*6/*6, UGT1A1*28/*28, or UGT1A1*6/*28

21. Known contraindication to receive cetuximab or irinotecan at the planned doses; refer to the most recent cetuximab and irinotecan SPC or local label as applicable

22. Current treatment with a non-topical medication known to be a strong inhibitor of CYP3A4. However, patients who either discontinue this treatment or switch to another medication at least 7 days prior to starting study treatment are eligible.

23. Concomitant use of St. John’s Wort (hypericum perforatum)

24. Other severe, acute or chronic medical or psychiatric condition or laboratory abnormality that may increase the risk associated with study participation or study drug administration or that may interfere with the interpretation of study results and, in the judgment of the Investigator, would make the patient an inappropriate candidate for the study

25. Pregnant, confirmed by a positive human chorionic gonadotropin (hCG) laboratory test result, or nursing (lactating)

26. Prior enrollment into this clinical study.

5.2.3 Eligibility Criteria for Treatment Crossover

5.2.3.1 Inclusion Criteria for Treatment Crossover

All of the following inclusion criteria must be met within 28 days prior to initiation of crossover treatment.

1. Patient was randomized to the Control arm and:
   - Discontinued Control arm treatment for any reason on or after 13 June 2019 (i.e., 4 weeks before the date of protocol version 8.0),
   - Received no systemic anticancer therapy after discontinuation of Control arm treatment, and
   - Remains in survival follow-up.
2. Provide a signed and dated Crossover informed consent document.
3. ECOG PS of 0 or 1.
4. Adequate bone marrow function characterized by the following:
   - Absolute neutrophil count (ANC) $\geq 1.5 \times 10^9$/L;
   - Platelets $\geq 100 \times 10^9$/L;
   - Hemoglobin $\geq$ 9.0 g/dL

Note: Transfusions will be allowed to achieve this. Transfusions will be permitted provided the patient has not received more than 2 units red blood cells in the prior 4 weeks to achieve these criteria.

5. Adequate renal function characterized by serum creatinine ≤ 1.5 × upper limit of normal (ULN), or calculated by Cockroft-Gault formula or directly measured creatinine clearance ≥ 50 mL/min.
6. Adequate electrolytes defined as serum potassium and magnesium levels within institutional normal limits (Note: replacement treatment to achieve adequate electrolytes will be allowed).
7. Adequate hepatic function characterized by the following:
    - Serum total bilirubin ≤ 1.5 × ULN and < 2 mg/dL

    Note: Patients who have a total bilirubin level > 1.5 × ULN will be allowed if their indirect bilirubin level is ≤ 1.5 × ULN

    - Alanine aminotransferase (ALT) and/or aspartate aminotransferase (AST) ≤ 2.5 × ULN, or ≤ 5 × ULN in presence of liver metastases.
8. Adequate cardiac function characterized by the following:
    - Mean triplicate QT interval corrected for heart rate using Fridericia's formula (QTcF) value ≤480 msec.
9. Able to take oral medications.
10. Willing and able to comply with scheduled visits, treatment plan, laboratory tests and other study procedures.
11. Female patients are either postmenopausal for at least 1 year, are surgically sterile for at least 6 weeks, or must agree to take appropriate precautions to avoid pregnancy through follow-up if of childbearing potential.
    Note: Permitted contraceptive methods listed in Section 5.3.1 should be communicated to the patients and their understanding confirmed. For all females, the pregnancy test result must be negative at crossover screening and at the time of crossover.
12. Males must agree to take appropriate precautions to avoid fathering a child through 90 days following end of therapy.
    Note: Permitted contraceptive methods listed in Section 5.3.1 should be communicated to the patients and their understanding confirmed.
13. Patients under guardianship or partial guardianship will be eligible unless prohibited by local laws or by local/central ethic committees (e.g., France, Germany). Where allowed, all procedures prescribed by law must be followed.

5.2.3.2 Exclusion Criteria for Treatment Crossover

Patients randomized to the Control arm who meet any of the following criteria within 28 days prior to initiation of crossover treatment will not be eligible for crossover.

1. Patients for whom cetuximab was permanently discontinued due to cetuximab-related toxicity in the randomized Phase 3 portion of the study.
2. Symptomatic brain metastasis.
   Notes: Patients previously treated or untreated for this condition who are asymptomatic in the absence of corticosteroid and anti-epileptic therapy are allowed. Brain metastases must be stable for ≥ 4 weeks, with imaging (e.g., MRI or CT) demonstrating no current evidence of progressive brain metastases.
3. Leptomeningeal disease.
4. Use of any herbal medications/supplements or any medications or foods that are strong inhibitors or inducers of cytochrome P450 (CYP) 3A4/5 ≤ 1 week prior to crossover.
5. Known history of acute pancreatitis.
6. History of chronic inflammatory bowel disease or Crohn's disease requiring medical intervention (immunomodulatory or immunosuppressive medications or surgery) ≤ 12 months prior to crossover.
7. Impaired cardiovascular function or clinically significant cardiovascular diseases, including any of the following:
   - History of acute myocardial infarction, acute coronary syndromes (including unstable angina, CABG, coronary angioplasty or stenting) ≤ 6 months prior to start of crossover;
   - Symptomatic congestive heart failure (i.e., Grade 2 or higher), history or current evidence of clinically significant cardiac arrhythmia and/or conduction abnormality ≤ 6 months prior to crossover, except atrial fibrillation and paroxysmal supraventricular tachycardia.
8. Impaired hepatic function, defined as Child Pugh class B or C.
9. Impaired gastrointestinal (GI) function or disease that may significantly alter the absorption of encorafenib or binimetinib (e.g., ulcerative diseases, uncontrolled vomiting, malabsorption syndrome, small bowel resection with decreased intestinal absorption).
10. Treatment with irinotecan within 3 weeks prior to Day 1 of crossover treatment.
11. Residual CTCAE ≥ Grade 2 toxicity from any prior anticancer therapy, with the exception of Grade 2 alopecia or Grade 2 neuropathy.
12. Concomitant use of St. John's Wort (hypericum perforatum).
13. Other severe, acute or chronic medical or psychiatric condition or laboratory abnormality that may increase the risk associated with study participation or study drug administration or that may interfere with the interpretation of study results and, in the judgment of the Investigator, would make the patient an inappropriate candidate for crossover treatment.

14. Pregnant, confirmed by a positive hCG laboratory test result, or nursing (lactating) at time of crossover.
15. Left ventricular ejection fraction (LVEF) < 50% as determined by a MUGA scan or ECHO.
16. Current evidence or history of RVO or current risk factors for RVO (e.g., uncontrolled glaucoma or ocular hypertension, history of hyperviscosity or hypercoagulability syndromes).
17. Uncontrolled hypertension defined as persistent systolic blood pressure ≥ 150 mmHg or diastolic blood pressure ≥ 100 mmHg despite current therapy.
18. History of thromboembolic or cerebrovascular events ≤ 6 months prior to crossover, including transient ischemic attacks, cerebrovascular accidents, deep vein thrombosis or pulmonary emboli.
19. Concurrent neuromuscular disorder that is associated with the potential of elevated CK (e.g., inflammatory myopathies, muscular dystrophy, amyotrophic lateral sclerosis, spinal muscular atrophy)

5.3 Lifestyle Guidelines

5.3.1 Contraception

Female patients are either postmenopausal for at least 1 year, are surgically sterile for at least 6 weeks, or must agree to take appropriate precautions to avoid pregnancy from screening through follow-up in any treatment arm if of childbearing potential. Males must agree to take appropriate precautions to avoid fathering a child from screening through 90 days following end of therapy.

Patients should also be referred to the locally approved prescribing information for cetuximab, irinotecan, fluorouracil and folinic acid for additional guidance (as applicable). The following methods have been determined to be effective and acceptable and are permitted under this protocol for use by the patient and his/her partner (Clinical Trials Facilitation Group Guidelines 2014):

- Complete abstinence from sexual intercourse when this is in line with the preferred and usual lifestyle of the patient
- Double barrier method (condom with spermicide in conjunction with use of an intrauterine device)
- Due to the potential of encorafenib to induce CYP3A4, hormonal agents (including but not limited to birth control patch, vaginal ring, oral, injectable, or implanted contraceptives) are permissible only when combined with other highly effective or acceptable methods

- Surgical sterilization (bilateral oophorectomy with or without hysterectomy, tubal ligation or vasectomy) at least 6 weeks prior to taking study treatment. In the case of oophorectomy alone, only when the reproductive status of the woman has been confirmed by follow-up levels of luteinizing hormone (LH), follicle-stimulating hormone (FSH), and/or estradiol.
- It is recommended that patients experiencing vomiting or diarrhea should be counseled to use non-oral methods of contraception.

5.3.2 Photosensitivity

Patients treated with encorafenib, cetuximab and/or 5-FU should avoid extended exposure to ultraviolet light and when outdoors, should wear occlusive clothing, sunscreen and sunglasses while receiving study drugs and for 2 months following the last dose of encorafenib, cetuximab and/or 5-FU.

6.0 STUDY TREATMENT AND CONCOMITANT MEDICATIONS/THERAPIES

The term “study drug” is used to refer to encorafenib, binimetinib, cetuximab, irinotecan, and 5-FU and FA, collectively. In addition to the information provided in this protocol, refer to the SPC for cetuximab, irinotecan, fluorouracil, and folinic acid for the management of patients, concerning contraindications, duration of contraception, special warnings and precautions, and medications that are contraindicated or that must be used with caution. Patients with known contraindications to either 5-FU or FA may be included in the study, and if randomized to the control arm, will be treated with irinotecan and cetuximab.

6.1 Patient Numbering

Patients who sign informed consent to participate in Molecular Prescreening will be assigned a unique number by IWRS. This number will identify the patient if they decide to participate in screening and if they are eventually enrolled into the study. Once a patient is in screening or is enrolled in the study, that patient will be identified only by the assigned patient number. Once assigned, the patient number must not be reused for any other patient.

Patients who sign informed consent to enter Screening based on molecular characterization of their tumor based on a prior test will be assigned a unique number by IWRS which will identify the patient if they are eventually enrolled into the study. Once assigned, the patient number must not be reused for any other patient.

6.2 Allocation to Treatment

In Phase 3, patients will be randomized in a 1:1:1 ratio to receive encorafenib + binimetinib + cetuximab (Triplet Arm), encorafenib + cetuximab (Doublet Arm) or to the Control Arm (irinotecan/cetuximab or FOLFIRI/cetuximab, per Investigator’s choice).

The number of patients with 2 prior regimens will be limited to 215 after which only patients with 1 prior regimen will be randomized. Patients with 2 prior regimens who have entered Screening at the time that the limit has been reached will be permitted to continue into the study if they are otherwise determined to be eligible.

Randomization will be stratified by ECOG PS (0 or 1), prior use of irinotecan (yes or no), and cetuximab source (US-licensed vs. EU-approved). The randomization schedule will be created and managed by an independent statistician not assigned to support the study and treatments will be assigned according to a computerized central randomization list using an IWRS.

6.2.1 Treatment Crossover

Upon implementation of protocol version 8, patients randomized to the Control Arm who meet crossover eligibility criteria (Section 5.2.3) may cross over to receive the triplet regimen.

6.3 Doses and Schedule of Administration

The investigational products in this study are encorafenib and binimetinib, which will be administered PO in combination with cetuximab (i.e., encorafenib + binimetinib + cetuximab [Safety Lead-in and Triplet Arm] and encorafenib + cetuximab [Doublet Arm]). The comparator combination treatment will be the Investigator's choice of either irinotecan/cetuximab or FOLFIRI/cetuximab administered IV (Control Arm) (Table 9).

Table 9: Starting Dose and Treatment Schedule

Study Treatments	Pharmaceutical Form and Route of Administration	Dose	Frequency
**Safety Lead-in and Triplet Arm**			
Encorafenib	4 × 75 mg oral capsule	300 mg	QD
Binimetinib	3 × 15 mg oral film-coated tablet	45 mg	BID
Cetuximab	IV infusion	400 mg/m^2 initial dose (120-minute infusion), then 250 mg/m^2 (60-minute infusion) thereafter	once weekly
**Doublet Arm**			
Encorafenib	4 × 75 mg oral capsule	300 mg	QD
Cetuximab	IV infusion	400 mg/m^2 initial dose (120-minute infusion), then 250 mg/m^2 (60-minute infusion) thereafter	once weekly
**Control Arm**			
Irinotecan/Cetuximab			
Irinotecan	IV infusion	180 mg/m^2 (90-minute infusion or as per institutional standards)	every 2 weeks
Cetuximab	IV infusion	400 mg/m^2 initial dose (120-minute infusion), then 250 mg/m^2 (60-minute infusion) thereafter	once weekly
FOLFIRI/Cetuximab			
Irinotecan	IV infusion	180 mg/m^2 (90-minute infusion or as per institutional standards)	every 2 weeks
Folinic acid[a]	IV infusion	400 mg/m^2 (120-minute infusion or as per institutional standards) or maximal dose tolerated in a prior regimen	every 2 weeks
5-FU[a]	IV bolus/IV infusion	400 mg/m^2 initial dose bolus (not to exceed 15 minutes), then 1200 mg/m^2/day x 2 days (total 2400 mg/m^2 over 46-48 hours) continuous infusion or maximal dose tolerated in a prior regimen	every 2 weeks
Cetuximab	IV infusion	400 mg/m^2 initial dose (120-minute infusion), then 250 mg/m^2 (60-minute infusion) thereafter	once weekly
**Crossover Triplet Regimen**			
Encorafenib	4 × 75 mg oral capsule	300 mg	QD
Binimetinib	3 × 15 mg oral film-coated tablet	45 mg	BID
Cetuximab	IV infusion	250 mg/m^2 (60-minute infusion) or the dose level the patient was receiving in the Control arm prior to crossover	once weekly

Patients who experienced unacceptable toxicities requiring 5-FU and FA dose reductions in prior regimens (e.g. as part of FOLFOX or FOLFOXIRI regimens) may be initiated at the highest doses which were previously tolerated.

6.3.1 Administration of Encorafenib or Encorafenib + Binimetinib

Encorafenib will be administered on a QD schedule and binimetinib will be administered on a BID schedule, both PO as a flat-fixed dose, and not by body weight or body surface area (BSA) (Table 9). Binimetinib and encorafenib should be taken without regard to food. Patients should be instructed to swallow the capsules/tablets whole and not to chew or crush them.

- **QD Dosing**: Patients should be instructed to take encorafenib capsules daily with a large glass of water (~250 mL) in the morning at approximately the same time every day. Doses of encorafenib that are omitted for AEs or any other reason can be taken up to 12 hours prior to the next dose.

- **BID Dosing**: Patients should be instructed to take binimetinib tablets 12 ± 2 hours apart with a large glass of water (~250 mL) in the morning and in the evening at approximately the same times every day. Doses of binimetinib that are omitted for AEs or any other reason should not be made up later in the day, or at the end of the dosing period.

In the Safety Lead-in and Triplet Arm and in Crossover patients, both oral study drugs (encorafenib + binimetinib) are to be instructed to be taken together in the morning and only the BID administered drug (binimetinib) is to be taken in the evening without regard to food.

On days when a blood collection is scheduled at the investigational site, patients will take the morning dose of encorafenib and binimetinib (as applicable) at the site, after the collection, under the supervision of the Investigator or designee. On the evening of the visit day, patients will take binimetinib (as applicable) at home. On all other days, patients will take encorafenib and binimetinib (as applicable) at home. Predose PK samples for encorafenib and binimetinib analysis should be collected just prior to intake of encorafenib and binimetinib (as applicable).

If a patient vomits at any time after dosing, the dose of study drug should not be re-administered.

Patients must avoid consumption of grapefruit, pomegranates, star fruits, Seville oranges or products containing the juice of each during the entire study and preferably 7 days before the first dose of study drugs, due to potential CYP3A4 interaction with the study drugs. Orange juice is allowed.

Encorafenib and binimetinib (if applicable) will be administered at least 30 minutes prior to cetuximab.

The pharmacist or study nurse will ensure that the appropriate dose is dispensed and will provide the patient with at least the appropriate number of encorafenib capsules and binimetinib tablets (if applicable) for the number of doses to be taken prior to the next scheduled visit. The site personnel will train the patient and/or the patient's caregiver on dosing procedures for the study drug.

Patients will receive a diary to document self-administered dosing of encorafenib and binimetinib (if applicable) in each cycle to include the dose of study drug taken, the date of dosing (and times if applicable), and if any doses were missed and the reason for the missed dose. One diary will be provided per cycle. Patients will be instructed to return unused encorafenib and binimetinib and the patient diary to the site at the end of each cycle. Drug accountability must be performed on a regular basis.

The Investigator or responsible site personnel should instruct the patient to take encorafenib and binimetinib (if applicable) as per protocol (promote compliance). The dosage prescribed and dispensed to the patient and all dose changes and all missed doses during the study must be recorded in the electronic case report form (eCRF).

6.3.2 Administration of Cetuximab

Cetuximab will be administered IV weekly on Days 1, 8, 15 and 22 (±3 days) of every 28-day cycle at the study site (Table 9) according to institutional standards. The initial cetuximab dose (Cycle 1 Day 1) is 400 mg/m^2 administered as a 120-minute IV infusion followed thereafter by a 250 mg/m^2 dose administered as a 60-minute IV infusion. The infusion rate should be consistant with the local label but should not exceed 10 mg/min. Close monitoring is required during the infusion and for at least 1 hour after the end of the infusion. If an infusion reaction occurs while cetuximab is being administered, the infusion should be stopped immediately, and the patients should be closely monitored and treated in line with institutional standards. Any rechallenge with cetuximab following an infusion reaction should be first discussed with the Sponsor. Cetuximab administration should be completed 1 hour prior to the start of FOLFIRI or irinotecan infusion for Control Arm patients. For patients who cross over from the Control arm, the first dose after crossover will be 250 mg/m^2 or the dose level the patient was receiving prior to crossover.

Premedications for routine cetuximab infusions may be used in accordance with the label and with the national and/or institutional standards, but preferably be based on a combination of an H1 antagonist (e.g., diphenhydramine) and dexamethasone (10 mg IV). Premedications should be administered approximately 30 minutes prior to cetuximab infusion. Following cetuximab label instructions, medications such as corticosteroids and antihistamines may be administered at the discretion of the Investigator to treat an existing infusion reaction, or as premedication for a patient who has previously experienced an infusion reaction. Predose PK samples for cetuximab analysis should be collected just prior to the beginning of the infusion of cetuximab.

Doses of cetuximab that are omitted for AEs or any other reason should not be made up. If cetuximab is discontinued, the frequency of study visits may be decreased after discussion with Sponsor.

6.3.3 Administration of Irinotecan

Irinotecan will be administered IV biweekly on Days 1 and 15 (±3 days) of every 28-day cycle at the study site (Table 9) according to institutional standards. The initial irinotecan dose (Cycle 1 Day 1) is 180 mg/m^2 administered as a 90-minute IV infusion or as per institutional standards.

Doses of irinotecan that are omitted for AEs or any other reason should not be made up.

6.3.4 Administration of FOLFIRI

The starting doses of 5-FU, FA and irinotecan are provided in Table 9. Patients who required 5-FU and FA dose reductions in prior regimens (e.g., as part of FOLFOX or FOLFOXIRI regimens) should be initiated at the doses listed in Table 9 which most closely approximate, without exceeding, the previously tolerated dose. In these circumstances, the starting irinotecan dose should not be reduced. See Section 6.3.3 for information on the administration of irinotecan.

6.3.4.1 Folinic Acid

Folinic acid will be administered IV biweekly on Days 1 and 15 (±3 days) of every 28-day cycle at the study site (Table 9) according to institutional standards. The initial FA dose is 400 mg/m^2 administered as a 120-minute IV infusion or as per institutional standards. Alternatively FA may be administered (via separate infusion lines) concurrently with irinotecan.

Doses of FA that are omitted for AEs or any other reason should not be made up.

6.3.4.2 5-Fluorouracil

5-Fluorouracil will be administered IV biweekly on Days 1 and 15 (±3 days) of every 28-day cycle at the study site (Table 9) immediately following completion of the FA infusion according to institutional standards. The initial 5-FU dose is 400 mg/m^2 bolus (not to exceed 15 minutes) administered IV on Days 1 and 15, followed by 1200 mg/m^2/day x 2 days (total 2400 mg/m^2 over 46-48 hours) continuous IV infusion or as per institutional standards.

Doses of 5-FU that are omitted for AEs or any other reason should not be made up.

6.4 Dose Modifications

Patients will be monitored for AEs on an ongoing basis. The severity of AEs will be evaluated using the NCI CTCAE, v.4.03. If a patient develops a toxicity, the dose may be modified as outlined in Table 16, Table 17, Table 18 and Table 19, which include criteria for interruption and reduction of encorafenib and/or binimetinib, cetuximab, FOLFIRI and irinotecan when given without 5-FU and FA, respectively. All dose modifications should be based on the worst preceding toxicity. All dosing interruptions and modifications must be recorded in the eCRF.

In the Safety Lead-in and Triplet Arms and in Crossover patients, if a patient permanently discontinues binimetinib due to an AE or clinically significant laboratory value, they may continue to receive encorafenib in combination with cetuximab. Due to the lack of efficacy of binimetinib, encorafenib, or cetuximab when used as single agents in patients with *BRAF*-mutant mCRC, patients who cannot tolerate these agents in combination with at least one other agent, should discontinue study treatment altogether, complete the end of treatment visit and continue to be followed for survival (and disease progression, if applicable).

Cetuximab may be reduced 2 dose levels to a minimum of 150 mg/m^2 for AEs or laboratory abnormalities (Table 10). When a dose reduction is required because of an AE, no subsequent dose re-escalation of cetuximab will be permitted for that patient for the duration of study treatment. If after resolution of an AE, treatment is resumed at the same dose, and the same toxicity reoccurs with the same severity, any re-initiation of treatment must be at the next lower dose level irrespective of duration, with some exceptions for skin toxicity. In addition, a patient must discontinue study treatment if, after treatment is resumed at a lower dose of cetuximab, the same toxicity reoccurs with the same or worse severity.

In the Control Arm, irinotecan may be reduced up to 3 dose levels to a minimum dose of 100 mg/m^2 (Table 10). When a dose reduction is required because of an AE, no subsequent dose re-escalation will be permitted for the duration of study treatment. If after resolution of an AE, treatment is resumed at the same dose and the same toxicity reoccurs with the same severity, any re-initiation of treatment must be at the next lower dose level irrespective of duration, with some exceptions for skin toxicity. In addition, a patient must discontinue study treatment if, after treatment is resumed at a lower dose of irinotecan, the same toxicity reoccurs with the same or worse severity. Due to the lack of efficacy of single-agent cetuximab or 5-FU/FA in patients with previously treated *BRAF*-mutant mCRC, patients who cannot tolerate irinotecan either alone or as a component of FOLFIRI should discontinue study treatment altogether, complete the end of treatment visit and continue to be followed for survival (and disease progression, if applicable).

For patients in the control arm receiving FOLFIRI an individual patient may have their doses of 5-FU reduced to the dose levels specified in Table 10. For FOLFIRI dose modification, dose modifications of irinotecan, 5-FU bolus or 5-FU infusion are to be made independently, based on the specific types of toxicity observed. In general, if a dose is reduced because of toxicity, it is not to be re-escalated to the starting level. However, patients who require multiple dose reductions during a cycle for a Grade 2 toxicity could, at the investigator's discretion, begin the following cycle at 1 dose level higher than the final dose level during that cycle.

Table 10: Dose Levels for Dose Modification

	Encorafenib (mg QD)	Binimetinib (mg BID)	Cetuximab (mg/m^2 once weekly)	Irinotecan (mg/m^2 every 2 weeks)	5-FU	
					5-FU Bolus (mg/m^2 every 2 weeks)	5-FU Infusion (mg/m^2 over 46-48 hours every 2 weeks)
**Starting Dose**	**300**	**45**	**400 initial dose then 250 thereafter[a]**	**180**	**400**	**2400**
**Dose level -1**	225	30	200	150	200	2000
**Dose level -2**	150	15	150	120	0	1600
**Dose level -3**				100	0	1200

Abbreviations: BID = twice daily; 5-FU = 5-fluorouracil; QD = once daily.

[a] 400 mg/m^2 initial dose (120-minute infusion), then 250 mg/m^2 (60-minute infusion) thereafter. For patients who cross over from the Control arm, the first dose after crossover will be 250 mg/m^2 or the dose level the patient was receiving prior to crossover.

If a patient misses > 28 consecutive days of encorafenib, or binimetinib, > 4 consecutive cetuximab doses, or 2 consecutive irinotecan, 5-FU or FA doses, as the result of an AE or clinically significant laboratory abnormality, then the respective agent should be discontinued. As previously noted, due to the lack of efficacy in patients with *BRAF*-mutant mCRC, patients will not be permitted to continue on single agent binimetinib, encorafenib or cetuximab and will be discontinued from study treatment altogether, and following completion of the end of treatment visit, will continue to be followed for survival (and disease progression, if applicable).

Patients receiving FOLFIRI may only continue in the study if they are able to continue with irinotecan.

Patients who discontinue study treatment for a study-related AE or an abnormal laboratory value must be followed as described in Section 8.6.

Under special circumstances, when according to the assessment of the Investigator, a patient with progression of disease may benefit from continuation of study treatment, treatment beyond progression can be considered following discussion with the Sponsor (see Section 4.5).

6.4.1 Dose Modifications for Encorafenib and/or Binimetinib

Doses of encorafenib and/or binimetinib should be adjusted for AEs throughout the study (Table 16). In general, doses should not be reduced or interrupted for Grade 1 AEs unless the AE is a specific ocular AE referred to in Table 16 but treatment to control symptoms should be provided as appropriate, if applicable.

An individual patient may have their dose of encorafenib and/or binimetinib reduced to the dose levels specified in Table 10. The lowest recommended dose level of encorafenib is 150 mg QD and the lowest recommended dose level of binimetinib is 15 mg BID. When the AE that resulted in a dose reduction improves to and remains stable at the patient's Baseline for a minimum of 14 days, the dose can be re-escalated to the next dose level at the discretion of the Investigator, provided there are no other concomitant toxicities that would prevent drug re-escalation. There is no limit to the number of times the patient can have their dose reduced or re-escalated (in increments specified in Table 10); however:

- No dose re-escalation of encorafenib is allowed after a dose reduction due to prolonged QTcF ≥ 501 msec
- No dose re-escalation of binimetinib is allowed after a dose reduction due to LVEF dysfunction or prolonged QTcF ≥ 501 msec
- No dose re-escalation of binimetinib or encorafenib is allowed after a dose reduction due to retinal toxicity ≥ Grade 2.

Please refer to Table 16 for recommended dose modifications for encorafenib and/or binimetinib, if applicable, based on the occurrence of encorafenib and/or binimetinib treatment-related AEs.

Eye disorders that cannot be specifically graded according to NCI CTCAE, v.4.03 should be graded according to Table 11. Serous detachment of the retina should be graded according to Table 12. Uveitis should be graded according to NCI CTCAE, v.4.03 as described in Table 13. Hand-foot skin reaction should be graded according to NCI CTCAE, v.4.03 as described in Table 14. Diarrhea should be graded according to modified NCI CTCAE, v.4.03 as described in Table 15. Furthermore, please refer to the following appendices for additional supportive care recommended guidelines for the management of cetuximab-induced, encorafenib-induced and/or binimetinib-induced skin toxicity (Appendix 1), encorafenib-induced HFSR (Appendix 2), binimetinib-induced diarrhea (Appendix 3) and binimetinib-associated interstitial lung disease/pneumonitis (Appendix 8).

Table 11: Modified NCI CTCAE, Version 4.03 Grading of Eye Disorders

Grade	Description
1	Asymptomatic or mild symptoms; clinical or diagnostic observations only; intervention not indicated
2	Moderate; minimal, local or noninvasive intervention indicated; limiting age-appropriate instrumental activities of daily living
3	Severe or medically significant but not immediately sight threatening; hospitalization or prolongation of existing hospitalization indicated; disabling; limiting self-care activities of daily living
4	Sight-threatening consequences; urgent intervention indicated; blindness (20/200 or worse) in the affected eye

Table 12: NCI CTCAE, Version 4.03 Grading of Serous Detachment of the Retina

Grade	Description
1	Asymptomatic (but with findings in ocular coherence tomography, fundoscopy and/or slit lamp biomicroscopy)
2	Symptomatic with moderate decrease in visual acuity (20/40[a] or better); limiting instrumental activities of daily living
3	Symptomatic with marked decrease in visual acuity (worse than 20/40[a]); limiting self-care activities of daily living
4	Blindness (20/200[a] or worse) in the affected eye

Note: For rhegmatogenous retinal detachment, grade according to NCI CTCAE v.4.03 Retinal Detachment.

[a] Please refer to Appendix 4 for Snellen Equivalence (Visual Acuity Conversion Chart).

Table 13: NCI CTCAE, Version 4.03 Grading of Uveitis

Grade	Description
1	Asymptomatic; clinical or diagnostic observations only
2	Anterior uveitis; medical intervention indicated
3	Posterior or pan-uveitis
4	Blindness (20/200[a] or worse) in the affected eye

[a] Please refer to Appendix 4 for Snellen Equivalence (Visual Acuity Conversion Chart).

Table 14: NCI CTCAE, Version 4.03 Grading of Hand-foot Skin Reaction (HFSR)[a]

Grade	Description[b]
1	Minimal skin changes or dermatitis (e.g., erythema, edema, numbness, dysesthesia, paresthesia, tingling or hyperkeratosis) without pain
2	Skin changes (e.g., peeling, blisters, bleeding, edema, or hyperkeratosis) with pain; limiting instrumental ADL
3	Severe skin changes (e.g., peeling, ulceration, blisters, bleeding, edema, or hyperkeratosis) with pain; limiting self-care ADL

Abbreviations: ADL=activities of daily living.

[a] HFSR or palmar-plantar erythrodysesthesia syndrome, a disorder characterized by redness, marked discomfort, swelling, and tingling in the palms of the hands or the soles of the feet;

[b] More specifics examples to Grade 1 and Grade 3 are added to facilitate proper grading [from the sorafenib package insert (West Haven, CT: Bayer Pharmaceuticals Corporation; 2007).

Table 15: Modified NCI CTCAE, Version 4.03 Grading of Diarrhea

Grade	Description
1	Increase of < 4 stools per day over Baseline; mild increase in ostomy output compared to Baseline
2	Increase of 4-6 stools per day over Baseline; moderate increase in ostomy output compared to Baseline

Grade	Description
1/2 Complicated	Definition as above (Grade 1/2) with the following complicating signs/symptoms:<br>• Moderate to severe cramping<br>• Grade ≥ 2 nausea/vomiting<br>• Decreased performance status<br>• Fever<br>• Sepsis<br>• Neutropenia<br>• Frank bleeding<br>• Dehydration<br>• Unresolved diarrhea after 48 hours of treatment with loperamide (including high-dose administration) and initiation of second-line treatment
3	Increase of ≥ 7 stools per day over Baseline; incontinence; hospitalization indicated; severe increase in ostomy output compared to Baseline; limiting self-care activities of daily living
4	Life threatening consequences; urgent intervention indicated

Table 16: Recommended Dose Modifications for Encorafenib-related and/or Binimetinib-related Adverse Events

**Worst toxicity CTCAE, v.4.03 Grade (unless otherwise specified[a])**	**Dose Modification for Encorafenib (Doublet Arm, Triplet Arm and Crossover Patients) and for Binimetinib (Triplet Arm and Crossover Patients)**
**Eye Disorders - Retinal Events (including serous detachment of the retina), Posterior Uveitis[c]** **Note: Results and images of ocular coherence tomography (OCT) must be made available upon request. Any visual acuity impairment at screening should be documented and should be considered as baseline.**	
Grade 1	Maintain dose levels of encorafenib and binimetinib and repeat ophthalmic monitoring including visual acuity assessment and OCT within 10 days<br>• If patient remains asymptomatic (Grade 1), maintain dose level of encorafenib and binimetinib and continue the schedule of visual assessments established per protocol<br>• If patient becomes symptomatic (blurred vision, photophobia, etc.) or visual acuity assessment shows Grade 2, follow Grade 2 dose guidelines below
Grade 2	Interrupt dosing of encorafenib and binimetinib and repeat ophthalmic monitoring including visual acuity assessment and OCT within 10 days<br>• If resolved to baseline or Grade ≤ 1, resume treatment at current dose level of encorafenib and binimetinib and continue the schedule of visual assessments established per protocol<br>• If not resolved to baseline or Grade ≤ 1, resume treatment at 1 reduced dose level[b] of encorafenib and binimetinib and continue the schedule of visual assessments established per protocol<br>• If posterior uveitis lasts > 6 weeks, permanently discontinue binimetinib and encorafenib.
Grade 3	Interrupt dosing of encorafenib and binimetinib and repeat ophthalmic monitoring including visual acuity assessment and OCT within 10 days:<br>• If posterior uveitis resolves to Baseline or Grade ≤ 1 in < 6 weeks, resume treatment at 1 reduced dose level[b] of encorafenib and binimetinib and continue the schedule of visual assessments established per protocol<br>• If posterior uveitis does not resolve to Baseline or Grade ≤ 1 in < 6 weeks, permanently discontinue binimetinib and encorafenib<br>• If resolved to baseline or Grade ≤ 2, resume treatment at 1 reduced dose level[b] of encorafenib and binimetinib and continue the schedule of visual assessments established per protocol<br>• If not resolved to baseline or Grade ≤ 2, continue the interruption and repeat the ophthalmic assessment in 10 days.<br>o If resolved to baseline or Grade ≤ 2, resume treatment at 1 reduced dose level[b] of encorafenib and binimetinib and continue the schedule of visual assessments established per protocol<br>o If remains Grade 3, permanently discontinue encorafenib and binimetinib
Grade 4	Permanently discontinue encorafenib and binimetinib and immediate follow-up with ophthalmic monitoring[c]

Worst toxicity CTCAE, v.4.03 Grade (unless otherwise specified[a])	Dose Modification for Encorafenib (Doublet Arm, Triplet Arm and Crossover Patients) and for Binimetinib (Triplet Arm and Crossover Patients)
**Eye Disorder - RVO[c]** **Note: Results and images of ophthalmic examinations should be made available upon request. This includes scans/images of fluorescein angiography should a patient be assessed using this technique.**	
RVO of any grade	Permanently discontinue encorafenib and binimetinib and immediately follow-up with ophthalmic monitoring[c]
**Other Eye Disorders (i.e., Non-retinal Events)**	
Grade 1 – 2	Maintain dose level of encorafenib and binimetinib and increase frequency of ophthalmic monitoring to at least every 14 days until stabilization or resolution
Grade 3	Interrupt dosing of encorafenib and binimetinib and refer patient to ophthalmologist within 7 days[c]:<br>• If resolved to Grade ≤ 1 in ≤ 21 days, resume treatment at 1 reduced dose level[b] of encorafenib and binimetinib<br>• If not resolved to Grade ≤ 1 in ≤ 21 days, permanently discontinue encorafenib and binimetinib and close follow-up with ophthalmic monitoring until stabilization or resolution[c]
Grade 4	Permanently discontinue encorafenib and binimetinib and immediate follow-up with ophthalmic monitoring until stabilization or resolution[c]
**Liver-related Adverse Events**	
Grade 1<br>AST or ALT<br>> ULN to 3 × ULN	Maintain dose level of encorafenib and binimetinib
Grade 2<br>AST or ALT<br>> 3 to 5.0 × ULN or 3 × baseline value[d]<br>**AND**<br>**blood bilirubin[g]**<br>**≤ 2.0 × ULN**	Maintain dose level of encorafenib and interrupt dosing of binimetinib until resolved to Grade ≤ 1 (or Grade ≤ 2 in case of liver metastasis), then:<br>• If resolved in ≤ 14 days, maintain dose level of encorafenib and binimetinib<br>• If not resolved in ≤ 14 days, interrupt dose of encorafenib (in addition to prior binimetinib) until resolved to Grade ≤ 1 (or Grade ≤ 2 in case of liver metastasis), then resume treatment at current dose level of encorafenib and 1 reduced dose level[b] of binimetinib<br>If additional occurrence:<br>• Interrupt dosing of encorafenib and binimetinib until resolved to Grade ≤ 1 (or Grade ≤ 2 in case of liver metastasis), then resume treatment at 1 reduced dose level[b] of encorafenib and binimetinib<br>Treatment with encorafenib and binimetinib may be resumed sequentially at the Investigator's discretion, with encorafenib being resumed alone for one week before resuming binimetinib treatment
AST or ALT<br>> 3.0 to 5.0 × ULN<br>**AND**<br>**blood bilirubin[g]**<br>**> 2.0 × ULN**	Interrupt dosing of encorafenib and binimetinib until resolved to Grade ≤ 1, then:<br>• If resolved in ≤ 7 days, resume treatment at 1 reduced dose level[b] of encorafenib and binimetinib<br>• If not resolved in ≤ 7 days, permanently discontinue encorafenib and binimetinib<br>Treatment with encorafenib and binimetinib may be resumed sequentially at the investigator's discretion, with encorafenib being resumed alone for one week before

**Worst toxicity CTCAE, v.4.03 Grade (unless otherwise specified[a])**	**Dose Modification for Encorafenib (Doublet Arm, Triplet Arm and Crossover Patients)<br>and for Binimetinib (Triplet Arm and Crossover Patients)**
	resuming binimetinib treatment
Grade 3<br>AST or ALT<br>> 5.0 to 8.0 × ULN)<br>**AND**<br>**blood bilirubin[g]**<br>**≤ 2.0 × ULN**	Interrupt dosing of encorafenib and binimetinib until resolved to Grade ≤ 1 (or Grade ≤ 2 in case of liver metastasis), then:<br>• If resolved in ≤ 14 days, resume treatment at current dose level of encorafenib and binimetinib<br>• If not resolved in ≤ 14 days, resume treatment at 1 reduced dose level[b] of encorafenib and binimetinib<br>Treatment with encorafenib and binimetinib may be resumed sequentially at the investigator's discretion, with encorafenib being resumed alone for one week before resuming binimetinib treatment<br><br>If additional occurrence:<br>• Interrupt dosing of encorafenib and binimetinib until resolved to Grade ≤ 1 (or Grade ≤ 2 in case of liver metastasis), then resume treatment at 1 reduced dose level[b] of encorafenib and binimetinib
AST or ALT<br>>8 × ULN<br>**AND blood bilirubin[g]**<br>**≤ 2.0 × ULN**	Permanently discontinue encorafenib and binimetinib
AST or ALT<br>> 5.0 × ULN<br>**AND**<br>**blood bilirubin[g]**<br>**> 2.0 × ULN**	Permanently discontinue encorafenib and binimetinib
Grade 4<br>AST or ALT<br>> 20.0 × ULN	Permanently discontinue encorafenib and binimetinib
**Cardiac Disorders - Left Ventricular Systolic Dysfunction[a] (Dose Adjustment for Binimetinib ONLY)**	
Asymptomatic absolute decrease of > 10% in LVEF compared to baseline and the LVEF is below the institution's LLN (e.g., a decrease of 60% to 48% is an absolute decrease of 12%)	Interrupt dosing of binimetinib and repeat evaluation of LVEF within 2 weeks<br>• If the LVEF recovers (defined as LVEF ≥ 50% or ≥ LLN and absolute decrease ≤ 10% compared to baseline) ≤ 21 days, resume treatment at 1 reduced dose level[b] of binimetinib after approval of the Sponsor Medical Monitor. Monitor LVEF 2 weeks after resuming binimetinib, every 4 weeks for 12 weeks and subsequently as per protocol<br>• If the LVEF does not recover in ≤ 21 days, permanently discontinue binimetinib. Closely monitor LVEF until resolution or for up to 16 weeks

**Worst toxicity CTCAE, v.4.03 Grade (unless otherwise specified[a])**	**Dose Modification for Encorafenib (Doublet Arm, Triplet Arm and Crossover Patients) and for Binimetinib (Triplet Arm and Crossover Patients)**
Grade 3 – 4	Permanently discontinue binimetinib. Closely monitor LVEF until resolution or up to 16 weeks<br>Note: Copies of ECHO and/or MUGA scans could be requested for patients to be available to the Sponsor for patients with absolute decrease of >10% in LVEF compared to baseline and LVEF < 50% or LLN
**CK Elevation**	
Grade 1-2	Maintain dose of encorafenib and binimetinib. Ensure patient is adequately hydrated. Closely monitor CK and serum creatinine<br>• If total CK ≥ 3 × ULN, measure CK isoenzymes and myoglobin in blood or urine
Grade 3<br>> 5.0 - 10.0 x ULN<br>**without**<br>renal impairment (i.e., serum creatinine < 1.5 × ULN or 1.5 × baseline)	If asymptomatic, maintain dosing of encorafenib and binimetinib. Ensure patient is adequately hydrated. Monitor and measure isoenzymes and myoglobin in blood or urine and serum creatinine<br><br>If symptomatic (muscle pain/spasms/muscle weakness), maintain dosing of encorafenib and interrupt dosing of binimetinib until resolved to CTCAE Grade ≤ 1 and monitor closely, then:<br>• If resolved in ≤ 21 days, maintain dose of encorafenib and resume treatment at 1 reduced dose level[b] of binimetinib<br>• If not resolved in ≤ 21 days, maintain dose of encorafenib and permanently discontinue binimetinib
Grade 4<br>**without**<br>renal impairment (i.e., serum creatinine < 1.5 × ULN or 1.5 × baseline)	If asymptomatic, maintain dose of encorafenib and interrupt dosing of binimetinib. Ensure patient is adequately hydrated. Monitor and measure isoenzymes and myoglobin in blood or urine and serum creatinine<br>• If resolved in ≤ 21 days, maintain dose of encorafenib and resume treatment at 1 reduced dose level[b] of binimetinib<br>• If not resolved in ≤ 21 days, maintain dose of encorafenib and permanently discontinue binimetinib<br>If symptomatic (muscle pain/spasms/muscle weakness), maintain dose of encorafenib and permanently discontinue binimetinib
Grade 3 or 4<br>**with**<br>renal impairment (i.e., serum creatinine ≥ 1.5 × ULN or 1.5 × baseline)	Interrupt dosing of encorafenib and binimetinib until resolved to CTCAE Grade < 1 or baseline level. Ensure patient is adequately hydrated. Monitor closely and measure isoenzymes and myoglobin in blood or urine and serum creatinine, then:<br>• If resolved in ≤ 21 days, consider resuming treatment at 1 reduced dose level[b] of encorafenib and binimetinib<br>• If not resolved in ≤ 21 days, permanently discontinue encorafenib and binimetinib<br>2nd occurrence:<br>• Permanently discontinue encorafenib and binimetinib
**Cardiac Investigation – Prolongation of the QT interval QTcF value**	
QTcF > 500 ms during treatment and change from pre-treatment value remains ≤ 60 ms	Patients should have regular ECG monitoring (continuous where appropriate) until an adequately trained physician (such as a cardiologist or internist) has reviewed the data. Electrolyte abnormalities including magnesium should be corrected and cardiac risk factors for QT prolongation (e.g., congestive heart failure, bradyarrhythmias) should be controlled.<br>1st occurrence:

**Worst toxicity CTCAE, v.4.03 Grade (unless otherwise specified[a])**	**Dose Modification for Encorafenib (Doublet Arm, Triplet Arm and Crossover Patients) and for Binimetinib (Triplet Arm and Crossover Patients)**
	• Temporarily interrupt dosing of encorafenib and binimetinib until QTcF < 500 ms. Then resume treatment at 1 reduced dose level[b] of encorafenib and binimetinib<br>2nd occurrence:<br>• Temporarily interrupt dosing of encorafenib and binimetinib treatment until QTcF < 500 ms. Then resume treatment at 1 reduced dose level[b] of encorafenib and binimetinib. If a patient restarts binimetinib and encorafenib following resolution of Grade 3 QTcF prolongation event, the patient should be evaluated with triplicate predose ECGs on Day 1 of the next cycle, followed by a single postdose ECG and a single predose ECG on Day 15, as well as triplicate predose ECGs and a single postdose ECG on Days 1 and 2 of the subsequent cycle (2nd cycle after the Grade 3 QT prolongation event).<br>3rd occurrence:<br>• Permanently discontinue encorafenib and binimetinib
QTcF increase during treatment is both > 500 ms and > 60 ms change from pre-treatment values	Patients should have regular ECG monitoring (continuous where appropriate) until an adequately trained physician (such as a cardiologist or internist) has reviewed the data. Electrolyte abnormalities including magnesium should be corrected and cardiac risk factors for QT prolongation (e.g., congestive heart failure, bradyarrhythmias) should be controlled.<br>• Permanently discontinue encorafenib and binimetinib
**Rash [see cetuximab dose modifications (Table 17) and Appendix 1]**	
Grade 1	Maintain dose level of encorafenib and binimetinib<br>Initiate Initial Rash Treatment Regimen if it was not already started and rash should be closely monitored
Grade 2	1st occurrence:<br>• Maintain dose level of encorafenib and binimetinib<br>• Initiate Initial Rash Treatment Regimen if it was not already started and rash should be closely monitored<br>• Reassess within ≤ 14 days. If rash worsens or does not improve, interrupt dosing of encorafenib and binimetinib until resolved to Grade ≤ 1. Then resume treatment at current dose level of encorafenib and binimetinib. For dermatitis acneiform, treatment with encorafenib may be maintained if, in the judgment of the investigator, the rash is considered to be unrelated to encorafenib. If treatment with encorafenib was maintained and no improvement within 8 days, interrupt dosing of encorafenib<br>2nd occurrence:<br>• Reassess within ≤ 14 days. If rash worsens or does not improve, interrupt dosing of encorafenib and binimetinib until resolved to Grade ≤ 1. Then resume treatment at current dose level of encorafenib and 1 reduced dose level[b] of binimetinib. For dermatitis acneiform rash, treatment with encorafenib may be maintained if, in the judgment of the investigator, the rash is considered to be unrelated to encorafenib. If treatment with encorafenib was maintained and no improvement within 8 days, interrupt dosing of encorafenib

**Worst toxicity CTCAE, v.4.03 Grade (unless otherwise specified[a])**	**Dose Modification for Encorafenib (Doublet Arm, Triplet Arm and Crossover Patients) and for Binimetinib (Triplet Arm and Crossover Patients)**
Grade 3	1st occurrence:<br>• Interrupt dosing of encorafenib and binimetinib until resolved to Grade ≤ 1. Reassess weekly. Then resume treatment at current dose level of encorafenib and binimetinib.<br>• Consider referral to dermatologist and manage rash per dermatologist's recommendation.<br>2nd occurrence:<br>• Interrupt dosing of encorafenib and binimetinib until resolved to Grade ≤ 1. Then resume treatment at 1 reduced dose level[b] of encorafenib and binimetinib. Resume treatment with encorafenib at the same dose level if, in the judgment of the Investigator, the rash is considered to be unrelated to encorafenib<br>• Consider referral to dermatologist and manage rash per dermatologist's recommendation
Grade 4	Permanently discontinue encorafenib and binimetinib[f]
**Hand-foot Skin Reaction (HFSR)/Palmar-plantar Erythrodysesthesia Syndrome[e] (Dose Adjustment for Encorafenib ONLY) (See Appendix 2)**	
Grade 1	Maintain dose of encorafenib. Promptly institute supportive measures, such as topical therapy, for symptomatic relief. Give instruction on life-style modifications.
Grade 2	1st occurrence:<br>• Maintain dose of encorafenib and HFSR should be closely monitored. Promptly institute supportive measures, such as topical therapy, for symptomatic relief. Give instruction on life-style modifications.<br>• If no improvement ≤ 14 days, interrupt dosing of encorafenib until resolved to Grade ≤ 1. Resume treatment with encorafenib at current dose level. Continue supportive measures, such as topical therapy, for symptomatic relief. Give instruction on life-style modifications.<br>Additional occurrence:<br>• Treatment with encorafenib may be maintained or interrupted based upon the Investigator's discretion. Continue supportive measures, such as topical therapy, for symptomatic relief. Give instruction on life-style modifications.<br>• If interrupted dosing of encorafenib per investigator's judgment, interrupt until resolved to Grade ≤ 1. Resume treatment with encorafenib at the same dose level or 1 reduced dose level[b] based upon the Investigator's discretion.
Grade 3	1st or additional occurrence:<br>• Interrupt dosing of encorafenib until resolved to Grade ≤ 1. Promptly initiate supportive measures, such as topical therapy, for symptomatic relief. Give instruction on life-style modifications. Reassess the patient weekly. Then resume treatment at one reduced dose level[b] of encorafenib<br>• Consider referral to dermatologist and manage HFSR per dermatologist's recommendation<br>> 3nd occurrence:<br>• Interrupt dosing of encorafenib until resolved to Grade ≤ 1, decision to resume treatment with encorafenib at one reduced dose level[b] or permanently discontinue encorafenib should be based upon the Investigator's discretion.

**Worst toxicity CTCAE, v.4.03 Grade (unless otherwise specified[a])**	**Dose Modification for Encorafenib (Doublet Arm, Triplet Arm and Crossover Patients) and for Binimetinib (Triplet Arm and Crossover Patients)**
**SCC, KA and any Other Suspicious Skin Lesion (Dose Adjustment for Encorafenib ONLY)**	
Grade ≤ 3	Maintain dose of encorafenib (dose interruptions or modifications are not required). Treatment of SCC, KA, and any other suspicious skin lesion (eg. new primary melanoma) should occur based upon institutional practice.
**Diarrhea (See Appendix 3)**	
Uncomplicated Grade 1-2	Maintain dose of encorafenib. Consider temporary interruption of binimetinib until resolved to Grade ≤ 1. Then resume treatment at current dose level of binimetinib
Complicated Grade 1-2	Consider temporary interruption of encorafenib until resolved to Grade ≤ 1. Then resume treatment at current dose level of encorafenib<br>Interrupt dosing of binimetinib until resolved to Grade ≤ 1. Then resume treatment at 1 reduced dose level[b] of binimetinib
Grade 3-4	Interrupt dosing of encorafenib and binimetinib until resolved to Grade ≤ 1. Then resume treatment at current dose level of encorafenib if, in the judgment of the Investigator, the toxicity is considered to be unrelated to encorafenib, or at one reduced dose levelb. Resume treatment at 1 reduced dose level[b] of binimetinib
**Nausea/Vomiting**	
Grade 1-2	Maintain dose level of encorafenib and binimetinib. Promptly institute antiemetic measure.
Grade 3	Interrupt dosing of encorafenib and binimetinib until resolved to Grade ≤ 1. Then resume treatment at 1 reduced dose level[b] of encorafenib. Resume treatment with binimetinib at the current dose if, in the judgment of the Investigator, the toxicity is considered to be unrelated to binimetinib, or at 1 reduced dose level[b].<br>Note: Interrupt dosing of encorafenib and binimetinib for ≥ Grade 3 vomiting or Grade 3 nausea only if the vomiting or nausea cannot be controlled with optimal antiemetics (as per local practice)
Grade 4	Permanently discontinue encorafenib and binimetinib[f]
**Interstitial lung disease/pneumonitis (See Appendix 8)**	
Grade 1	Maintain dose level of encorafenib and binimetinib. Monitor weekly.
Grade 2	Maintain dose of encorafenib. Withhold binimetinib for up to 3 weeks.<br>If improved to Grade 0 or 1, resume treatment at 1 reduced dose level of binimetinib.<br>If not resolved within 3 weeks, permanently discontinue binimetinib.
Grade 3-4	Permanently discontinue binimetinib.
**All Other Adverse Events (Suspected To Be Related To Encorafenib and/or Binimetinib)**	
Grade 1-2	If the event is a persistent Grade 2 AE not responsive to a specific therapy, consider interruption or reduction of encorafenib and binimetinib, as applicable
Grade 3	Interrupt dosing of encorafenib and binimetinib until resolved to Grade ≤1 or to pretreatment/baseline level. If the event resolves ≤ 21 days, then study drug may be resumed at 1 reduced dose level[b] based upon the Investigator's discretion.
Grade 4	Permanently discontinue encorafenib and binimetinib[f]

[a] Not according to NCI CTCAE.

[b] Dose reduction below 150 mg QD for encorafenib, and below 15 mg BID for binimetinib is not allowed.

[c] Ophthalmic monitoring mandated for retinal events, posterior uveitis, RVO: further evaluation with specialized retinal imaging (e.g. ocular coherence tomography, fluorescein angiography). Any diagnosis of retinal events must

Worst toxicity CTCAE, v.4.03 Grade (unless otherwise specified[a])	Dose Modification for Encorafenib (Doublet Arm, Triplet Arm and Crossover Patients) and for Binimetinib (Triplet Arm and Crossover Patients)

be supported by presence or absence of symptoms, visual acuity assessment and findings in OCT.

[d] For patients enrolled with liver metastases and baseline LFT elevations.

[e] Disorder characterized by redness, marked discomfort, swelling, and tingling in the palms of the hands or the soles of the feet.

[f] A patient with a Grade 4 AE may resume treatment at the lower dose level if the AE recovers to Grade ≤ 1 within 28 days of discontinuing drug and, if in the opinion of the Investigator and Sponsor Medical Monitor, the event is not life-threatening and the patient can be managed and monitored for recurrence of AE. Any patients requiring a treatment interruption of duration > 28 days must discontinue study drug permanently.

[g] Refers to total bilirubin.

6.4.2 Dose Modifications for Cetuximab

Recommended dose modifications for cetuximab based on the occurrence of cetuximab treatment-related AEs are summarized in Table 17. Appendix 1 contains recommended guidelines for prophylactic and symptomatic treatment of cetuximab-induced rash.

Table 17: Recommended Dose Modifications for Cetuximab-related Adverse Events

Worst toxicity CTCAE, v.4.03 Grade	Dose Modification for Cetuximab During a Cycle of Therapy
**Infusion Reaction**	If an infusion reaction occurs while cetuximab is being infused, the infusion should be stopped immediately and the patient should be evaluated.
Grade 1 or 2	Restart and complete the disrupted infusion at the discretion of the Investigator. The infusion must be restarted at a reduced rate. Additional pre-medications such as antihistamines or low-dose systemic corticosteroids may be administered when the infusion is restarted per instutional standards.<br>All subsequent infusions must also be administered at the reduced rate.
Grade 3 or 4	Permanently discontinue cetuximab
**Rash [see encorafenib and/or binimetinib dose modifications (Table 16)]**	
Grade 1 or 2	Maintain dose level; consider initiating appropriate therapy (such as antihistamines, topical corticosteroids, and low-dose systemic corticosteroids)
Grade 3, despite therapy	Omit dose until resolved to ≤ Grade 2, then:<br>• If resolved in ≤ 7 days (or ≤ 14 days for acneiform rash), then maintain dose level<br>• If not resolved in ≤ 7 days despite appropriate skin toxicity therapy (or ≤ 14 days for acneiform rash), then permanently discontinue cetuximab

Worst toxicity CTCAE, v.4.03 Grade	Dose Modification for Cetuximab During a Cycle of Therapy
Grade 3 recurrent	Omit dose until resolved to ≤ Grade 2, then:<br>• If resolved in ≤ 7 days (or ≤ 14 days for acneiform rash), then decrease 1 dose level<br>• If not resolved in ≤ 7 days despite appropriate skin toxicity therapy (or ≤ 14 days for acneiform rash), then permanently discontinue cetuximab<br>• Permanently discontinue cetuximab after 3rd recurrence (upon 4th occurrence)
Grade 4, despite skin toxicity therapy	Permanently discontinue cetuximab

6.4.3 Dose Modifications for FOLFIRI Treatment

Recommended dose modifications for 5-FU and irinotecan based on the occurrence of FOLFIRI treatment-related AEs are summarized in Table 18. No dose modifications are implemented for FA. If 5-FU is held or omitted, then FA should also be held or omitted, accordingly.

Table 18: Recommended Dose Modifications for 5-FU and Irinotecan Treatment-related Adverse Events During FOLFIRI Treatment

Worst toxicity CTCAE, v.4.03 Grade (value)	Dose Modification for 5-FU and Irinotecan During a Cycle of FOLFIRI Therapy	At Start of Next Cycles of FOLFIRI Therapy (After Adequate Recovery), Compared with Starting Dose in Previous Cycle
**Dose modifications below are for both 5-FU and irinotecan unless otherwise noted.**		
**Hematologic Toxicities**		
A new cycle of therapy should not begin until the absolute neutrophil count (ANC) has recovered to ≥ 1500/mm^3 and the platelet count has recovered to ≥ 100,000/mm^3. Treatment should be delayed 1-2 weeks to allow for recovery from treatment-related toxicities, unless otherwise noted. If the patient has not recovered after a 28-day delay, consideration should be given to discontinuing FOLFIRI.		
**Neutropenia**		
Grade 1 (1500 to 1999/mm^3)	Maintain dose level	Maintain dose level
Grade 2 (1000 to 1499/mm^3)	Decrease 1 dose level	Maintain dose level
Grade 3 (500 to 999/mm^3)	Omit dose until resolved to ≤ Grade 1[a], then decrease 1 dose level	Decrease 1 dose level
Grade 4 (< 500/mm^3)	Omit dose until resolved to ≤ Grade 1[a], then decrease 2 dose levels	Decrease 2 dose levels
**Thrombocytopenia**		
Grade 1 (< LLN to 75,000/mm^3)	Maintain dose level	Maintain dose level
Grade 2 (75,000 to 50,000/mm^3)	Decrease 1 dose level	Maintain dose level
Grade 3 (50,000 to 25,000/mm^3)	Omit dose until resolved to ≤ Grade 2,	Decrease 1 dose level

Worst toxicity<br>CTCAE, v.4.03 Grade (value)	Dose Modification for 5-FU and Irinotecan During a Cycle of FOLFIRI Therapy	At Start of Next Cycles of FOLFIRI Therapy (After Adequate Recovery), Compared with Starting Dose in Previous Cycle
	then decrease 1 dose level	
Grade 4 (< 25,000/mm^3)	Omit dose until resolved to ≤ Grade 2, then decrease 2 dose levels	Decrease 2 dose levels
**Neutropenic Fever**	Omit dose until resolved to ≤ Grade 1[a] ANC and platelet count (for a maximum time of two, 2-week treatments [approx 28 days]), then decrease 2 dose levels	Decrease 2 dose levels
**Other Hematologic Toxicities**	Dose modifications for leukopenia and anemia during a cycle of therapy and at the start of subsequent cycles of therapy are also based on CTCAE toxicity criteria and are the same as those recommended for neutropenia as above.	
**Diarrhea**		
A new cycle of therapy should not begin until the treatment-related diarrhea has recovered to ≤ Grade 1 diarrhea. Treatment should be delayed 1 to 2 weeks to allow for recovery from treatment-related toxicities. If the patient has not recovered after a 14-day delay, consideration should be given to discontinuing FOLFIRI.		
Grade 1 (2-3 stools/day > pretx)	Maintain dose level	**Both -** Maintain dose level
Grade 2 (4-6 stools/day > pretx)	Decrease 1 dose level	**5-FU -** Decrease 1 dose<br>**Irinotecan -** Maintain dose level
Recurrent Grade 2 (4-6 stools /day > pretx)	Decrease 1 dose level	**Both -** Decrease 1 dose level
Grade 3 (7-9 stools/day > pretx)	Omit dose until resolved to ≤ Grade 1, then decrease 1 dose level	**Both -** Decrease 1 dose level
Grade 4 (≥ 10 stools/day > pretx)	Omit dose until resolved to ≤ Grade 1 (for a maximum time of two, 2-week treatments [approx 28 days]), then decrease 2 dose levels	**Both -** Decrease 2 dose levels
**Mucositis (Dose Adjustment for 5-FU ONLY)**		
Grade 1	Maintain dose level	Maintain dose level
Grade 2	Decrease 1 dose level	Decrease 1 dose level
Recurrent Grade 2	Decrease 1 dose level	Decrease 2 dose levels
Grade 3	Omit dose until resolved to ≤ Grade 1, then decrease 1 dose level	If unresolved to ≤ Grade 1, omit dose for maximum of 28 days, then decrease 2 dose levels
Grade 4	Omit dose until resolved to ≤ Grade 1 (for a maximum time of two, 2-week treatments [approx 28 days]), then decrease 2 dose levels	If unresolved to ≤ Grade 1, omit dose for maximum of 28 days, then decrease 2 dose levels

Worst toxicity<br>CTCAE, v.4.03 Grade (value)	Dose Modification for 5-FU and Irinotecan During a Cycle of FOLFIRI Therapy	At Start of Next Cycles of FOLFIRI Therapy (After Adequate Recovery), Compared with Starting Dose in Previous Cycle
**Nausea/Vomiting (Dose reductions should occur only if symptoms persist despite 2 treatments with adequate [combination] antiemetic treatment.)**		
Grade 1	Maintain dose level	Maintain dose level
Grade 2	Maintain dose level	Maintain dose level
Grade 3	**5-FU -** Maintain dose level<br>**Irinotecan -** Decrease 1 dose level	**5-FU -** Maintain dose level<br>**Irinotecan -** Decrease 1 dose level
Grade 4	**5-FU -** Decrease 1 dose level<br>**Irinotecan -** Decrease 1 dose level	**5-FU -** Decrease 1 dose level<br>**Irinotecan -** Decrease 1 dose level
**Skin Toxicity (Including Palmar Plantar Erythrodysesthesia) (Dose Adjustment for 5-FU ONLY)**		
Grade 1	Maintain dose level	Maintain dose level
Grade 2	Maintain dose level	Maintain dose level
Grade 3	Omit dose until resolved to ≤ Grade 1 (for a maximum time of two, 2-week treatments [approx 28 days]), then decrease 1 dose level	Decrease 1 dose level
Grade 4	Omit dose until resolved to ≤ Grade 1 (for a maximum time of two, 2-week treatments [approx 28 days]), then decrease 2 dose levels	Decrease 2 dose levels
**Cardiac Toxicity (Dose Adjustment for 5-FU ONLY)**		
Grade ≥ 2	Discontinue 5-FU	
**Neurocerebellar Toxicity (Dose Adjustment for 5-FU ONLY)**		
Any grade	Discontinue 5-FU	
**Other Nonhematologic[b] Toxicities**		
Grade 1	Maintain dose level	Maintain dose level
Grade 2	Maintain dose level	Maintain dose level
Grade 3	Omit dose until resolved to ≤ Grade 1, then decrease 1 dose level	Decrease 1 dose level
Grade 4	Omit dose until resolved to ≤ Grade 1, then decrease 1 dose level	Decrease 1 dose level

Abbreviations: approx = approximately, ANC = absolute neutrophil count, LLN = lower limit of normal, pretx = pretreatment.

[a] If a dose delay is required due to any grade of neutropenia prophylactic use of granulocyte colony-stimulating factor (G-CSF) and granulocyte-macrophage colony stimulating factor (GM-CSF) prior to the next administration of FOLFIRI is permitted at the Investigator's discretion. (Smith et al. 2015 ASCO)

[b] Excludes alopecia, anorexia, or asthenia.

6.4.4 Dose Modifications for Irinotecan When Given Without 5-FU and FA

Recommended dose modifications for irinotecan when given without 5-FU or FA based on the occurrence of irinotecan treatment-related AEs are summarized in Table 19.

Table 19: Recommended Dose Modifications for Irinotecan Treatment-related Adverse Events When Given Without 5-FU and FA

Worst toxicity<br>CTCAE, v.4.03 Grade (value)	Dose Modification for Irinotecan During a Cycle of Therapy	At Start of Next Cycles of Therapy (After Adequate Recovery) Compared with Starting Dose in Previous Cycle
**Hematologic Toxicities**		
A new cycle of therapy should not begin until the granulocyte count has recovered to ≥ 1500/mm^3 and the platelet count has recovered to ≥ 100,000/mm^3. Treatment should be delayed 1 to 2 weeks to allow for recovery from treatment-related toxicities. If the patient has not recovered after a 2-week delay, consideration should be given to discontinuing irinotecan.		
**Neutropenia**		
Grade 1 (1500 to 1999/mm^3)<br>Grade 2 (1000 to 1499/mm^3)<br>Grade 3 (500 to 999/mm^3)<br><br>Grade 4 (< 500/mm^3)	Maintain dose level<br>Decrease 1 dose level<br>Omit dose until resolved to ≤ Grade 2[a], then decrease 1 dose level<br>Omit dose until resolved to ≤ Grade 2[a], then decrease 2 dose levels	Maintain dose level<br>Maintain dose level<br>Decrease 1 dose level<br><br>Decrease 2 dose levels
**Neutropenic Fever**	Omit dose until resolved, then decrease 2 dose levels	Decrease 2 dose levels
**Other Hematologic Toxicities**	Dose modifications for leukopenia, thrombocytopenia, and anemia during a cycle of therapy and at the start of subsequent cycles of therapy are also based on CTCAE toxicity criteria and are the same as those recommended for neutropenia as above.	
**Diarrhea**		
A new cycle of therapy should not begin until the treatment-related diarrhea has recovered to ≤ Grade 1 diarrhea. Treatment should be delayed 1 to 2 weeks to allow for recovery from treatment-related toxicities. If the patient has not recovered after a 14-day delay, consideration should be given to discontinuing irinotecan.		
Grade 1 (2-3 stools/day > pretx)<br>Grade 2 (4-6 stools/day > pretx)<br>Recurrent Grade 2 (4-6 stools /day > pretx)	Maintain dose level<br>Decrease 1 dose level<br>Decrease 1 dose level	Maintain dose level<br>Maintain dose level<br>Decrease 1 dose level
Grade 3 (7-9 stools/day > pretx)<br><br>Grade 4 (≥ 10 stools/day > pretx)	Omit dose until resolved to ≤ Grade 2, then decrease 1 dose level<br>Omit dose until resolved to ≤ Grade 2 then decrease 2 dose levels	Decrease 1 dose level<br><br>Decrease 2 dose levels

**Worst toxicity CTCAE, v.4.03 Grade (value)**	**Dose Modification for Irinotecan During a Cycle of Therapy**	**At Start of Next Cycles of Therapy (After Adequate Recovery) Compared with Starting Dose in Previous Cycle**
**Other Nonhematologic[b] Toxicities**		
Grade 1	Maintain dose level	Maintain dose level
Grade 2	Decrease 1 dose level	Decrease 1 dose level
Grade 3	Omit dose until resolved to ≤ Grade 2, then decrease 1 dose level	Decrease 1 dose level
Grade 4	Omit dose until resolved to ≤ Grade 2, then decrease 2 dose levels	Decrease 2 dose levels

Abbreviations: approx = approximately; pretx = pretreatment.

[a] If a dose delay is required due to any grade of neutropenia prophylactic use of granulocyte colony-stimulating factor (G-CSF) and granulocyte-macrophage colony stimulating factor (GM-CSF) prior to the next administration of FOLFIRI is permitted at the Investigator's discretion. (Smith et al. 2015 ASCO)

[b] Excludes alopecia, anorexia, or asthenia.

6.5 Concomitant Medications/Therapies

6.5.1 Permitted Concomitant Medications/Therapies

In general, the use of any concomitant medication/therapies deemed necessary for the care of the patient is permitted, unless otherwise specified. Additional information regarding concomitant medications/therapies is provided in the Investigator's Brochures for encorafenib and binimetinib and the cetuximab and irinotecan locally approved labels.

Patients receiving medications outlined below must be carefully monitored for potentiating of toxicity due to any individual concomitant medication and may require dose titration of the drug substance. Investigators should use caution when prescribing concomitant medications, as clinical experience with these compounds in patients with cancer is often limited. Investigators should contact the Sponsor when they are unsure whether a drug should be prescribed to a patient in the clinical study. All concomitant medications/therapies, transfusions, procedures and dietary supplements must be documented on the eCRF. Refer to Appendix 5 for a list of medications to be used with caution as mentioned above.

6.5.1.1 Skin Toxicity Treatments

Patients should be treated for cetuximab-induced, encorafenib-induced and/or binimetinib-induced skin toxicity following the supportive care recommended guidelines for the management of these toxicities (Appendix 1). Prophylactic measures may be initiated as outlined in Appendix 1.

6.5.1.2 Hand-foot Skin Reaction Treatments

Because HFSR has been reported for encorafenib, it is recommended that patients are educated prior to starting study treatment to avoid activities that can cause friction on hands and feet. In addition, supportive measures for prevention and/or management of HFSR should be instituted. Clinical judgment and experience of the treating physician should guide the management plan of each patient. Patients receiving encorafenib should be treated for encorafenib-induced HFSR following the supportive care recommended guidelines for the management of these toxicities (Appendix 2).

6.5.1.3 Antidiarrheals

Patients should be treated for diarrhea as per institutional guidelines, and/or as indicated in the locally approved prescribing information, or for patients receiving binimetinib, per the supportive care recommended guidelines for the management of binimetinib-induced diarrhea (Appendix 3). For patients in the Control Arm, lacrimation, rhinorrhea, miosis, diaphoresis, hot flashes, flushing, abdominal cramping, diarrhea, or other symptoms of early cholinergic syndrome may occur during or shortly after receiving irinotecan. Atropine, 0.25 to 1.0 mg IV or subcutaneously, may be used (unless clinically contraindicated to treat these symptoms at the discretion of the treating physician. Combination anticholinergic medications containing barbiturates or other agents should not be used because these may affect irinotecan metabolism. Anticholinergics should be used in caution in patients with potential contraindications (e.g., obstructive uropathy, glaucoma and tachycardia). Late diarrhea (e.g., developing more than 24 hours after irinotecan) should be managed with loperamide.

6.5.1.4 Antiemetics

Prophylactic antiemetics should be started only once the patient experiences nausea or vomiting and at the discretion of the Investigator. It is recommended that patients use drugs that do not cause QT prolongation. Note that some antiemetics have a known risk for Torsade de Pointes (TdP) (Appendix 5).

6.5.2 Permitted Concomitant Therapy Requiring Caution and/or Action

6.5.2.1 CYP and UGT Substrates and Inhibitors

Encorafenib is a reversible inhibitor of CYP2B6, CYP2C9, CYP3A4 and UGT1A1. It is also a time-dependent inhibitor of CYP3A4. Binimetinib is also a reversible inhibitor of CYP2B6. Permitted medications to be used with caution in this study include those that are sensitive substrates of CYP2B6, CYP2C8, CYP2C9, CYP2C19, CYP3A4, and UGT1A1 or those substrates that have a narrow therapeutic index (NTI). Substrates of CYP3A4 are allowed based on a lack of interaction with midazolam (Appendix 5).

There is a potential for encorafenib to induce CYP3A4, which may reduce the effectiveness of hormonal contraception methods. Therefore, the use of at least 1 form of non-hormonal contraception is required during participation in this study. See Section 5.3.1 for use of contraception methods required for this study. Caution should be used in patients receiving concomitant treatment with other drugs that are substrates of CYP3A4 as the efficacy of these drugs could be reduced when administered with encorafenib.

Encorafenib has been identified to be metabolized by CYP3A4 and to a lesser extent by CYP2C19 in vitro. The use of strong inhibitors of CYP3A4 is prohibited (see Section 2.3.2). Moderate inhibitors of CYP3A4 and strong inhibitors of CYP2C19 should be used with caution when co-administered with encorafenib.

Binimetinib has been identified to be primarily metabolized by glucuronidation. It is advised that strong inhibitors of UGT1A1 should be taken with caution when co-administered with binimetinib (Appendix 5).

Patients should be closely monitored for the occurrence of AEs.

6.5.2.2 Transporter Substrates and Inhibitors

In vitro data showed that both encorafenib and binimetinib are substrates of the transporter P-glycoprotein (P-gp). Binimetinib is also a substrate of BCRP. Thus, the use of drugs that are known to inhibit or induce P-gp or BCRP should be used with caution. Encorafenib is a BCRP inhibitor. Encorafenib is also a potent inhibitor of the renal transporters, OAT1, OAT3 and OCT 2, and the hepatic transporter OATP1B1 and OATP1B3. The co-administration of drugs that are known to be sensitive or narrow therapeutic index (NTI) substrates of BCRP, P-gp, OAT1, OAT3, OCT 2, OATP1B1 and OATP1B3 should be used with caution (Appendix 5).

6.5.2.3 Hematopoietic Growth Factors

Hematopoietic growth factors (e.g., erythropoietin, granulocyte colony-stimulating factor [G-CSF] and granulocyte-macrophage colony stimulating factor [GM-CSF]) are not to be administered prior to first dose of study treatment. After irinotecan or FOLFIRI treatment, if a dose delay is required due to any grade of neutropenia, prophylactic use of G-CSF and GM-CSF prior to the next administration of FOLFIRI is permitted at the Investigator’s discretion. Use of these drugs should be reserved for patients requiring this therapy as per the labeling of these agents or as dictated by local practice (see also the ASCO guidelines; Smith et al. 2015).

6.5.2.4 Drugs with a Conditional or Possible Risk to Prolong the QT Interval and/or Induce Torsade de Pointes

Investigators should use caution when administering encorafenib or binimetinib with concomitant medications with a known, conditional or possible risk to prolong the QT interval and/or induce TdP (Appendix 5). Patients receiving such medications must be carefully

monitored for potentiating of toxicity due to any individual concomitant medication, and may require dose titration of the concomitant medication.

6.5.3 Prohibited Concomitant Therapy

6.5.3.1 Anticancer Therapy

No additional anticancer agents such as cytotoxic chemotherapy, small-molecule targeted agents, biological agents, immune response modifiers or hormonal therapy are to be administered to patients while they are receiving study drug.

6.5.3.2 Other Prohibited Therapies

The following therapies are prohibited during the study (unless otherwise noted):

- Investigational drugs and devices
- Radiation therapy (not including palliative radiotherapy at focal sites that covers ≤ 10% of the bone marrow reserve)
- Herbal preparations/medications. Patients should stop using herbal medications 7 days prior to first dose of study treatment.
- Concomitant strong systemic CYP3A4 inhibitors. These would likely significantly increase the exposure of encorafenib and thus should not be used during this study (see Appendix 5).

6.6 Study Drug Supply

6.6.1 Manufacturing, Formulation, Packaging and Labeling

Encorafenib is manufactured by Catalant Pharma Solutions and supplied by the Sponsor as capsules for PO administration in dosage strength of 75 mg and packaged into high-density polyethylene (HDPE) bottles with an induction seal, child-resistant cap and a desiccant. Encorafenib capsules consist of encorafenib drug substance and the following excipients: copovidone, poloxamer 188, succinic acid, microcrystalline cellulose, colloid silicon dioxide, crospovidone, and magnesium stearate of vegetable origin. The capsule shell is commercially available and contains gelatin and titanium dioxide as well as iron oxide red, yellow or black depending on the particular strength.

Binimetinib is manufactured by Almac Pharma Services Limited and Novartis Pharma AG and supplied by the Sponsor as film-coated tablets for PO administration in a dosage strength of 15 mg and packaged into HDPE bottles with an induction seal and child-resistant cap. Binimetinib film-coated tablets consist of binimetinib drug substance; colloidal silicon dioxide/silica colloidal anhydrous; croscarmellose sodium; lactose monohydrate; magnesium

stearate; microcrystalline cellulose/cellulose, microcrystalline; and a commercial film coating. The capsule-shaped tablets are yellow to dark yellow.

For both encorafenib and binimetinib, each bottle will be labelled, at a minimum, with a unique identifier (medication number), the lot number, contents (number of tablets), dosage strength, storage conditions and the name and address of the Sponsor. Both encorafenib and binimetinib labels will be in the local language and comply with the legal requirements of each country. For patients randomized to the Triplet Arm or the Doublet Arm, responsible site personnel will identify the study treatment bottle(s) to dispense to the patient by using the IWRS which will provide the medication number(s) at each dispensing visit. Site personnel will add the patient number to the label.

Cetuximab will be supplied either locally or by the Sponsor. A description of cetuximab for infusion is included in the locally approved prescribing information. Commercially available cetuximab vials are packaged in boxes and will be used in this study according to local regulations in each participating country. Cetuximab preparation must be in accordance with the label and with local institutional guidelines.

Irinotecan will be supplied either locally or by the Sponsor. A description of irinotecan for infusion is included in the locally approved prescribing information. Commercially available irinotecan vials are packaged in boxes and will be used in this study according to local regulations in each participating country. Irinotecan preparation must be in accordance with the label and with local institutional guidelines.

5-Fluoruracil and FA will be supplied either locally or by the Sponsor. A description of 5-FU and FA for IV administration is included in the locally approved prescribing information. Commercially available 5-FU and FA vials are packaged in boxes and will be used in this study according to local regulations in each participating country. 5-Fluoruracil and FA preparation must be in accordance with the label and with local institutional guidelines.

6.6.2 Shipping, Storage and Handling

Labeled, packaged encorafenib and binimetinib will be shipped to each site by the Sponsor or designee, as described in the Pharmacy Manual. The Investigator or an approved representative (e.g., registered pharmacist) will ensure that all encorafenib and binimetinib is stored as outlined in the Pharmacy Manual and in accordance with applicable regulatory requirements. The drug storage area at the site must be secure, with access limited to authorized personnel.

Encorafenib capsules should not be stored above 25°C and should be protected from moisture. Binimetinib film-coated tablets should not be stored above 25°C and should be protected from light. Storage conditions will be described on the medication label.

Stability studies to support drug storage conditions have been conducted by the Manufacturer or an affiliate. The Manufacturer will continue to monitor the stability of encorafenib and binimetinib and the Sponsor will alert the site if a lot is nearing the end of its anticipated shelf life.

Detailed instructions for storage and handling of encorafenib and binimetinib will be provided in the Pharmacy Manual.

Irinotecan, cetuximab, 5-FU and FA should be stored according to the locally approved prescribing information.

6.6.3 Accountability and Return of Study Drug Supply

The Investigator or an approved representative (e.g., pharmacist) must maintain accurate records of dates and quantities of study drug received, to whom study drug is dispensed (patient-by-patient accounting), and accounts of any study drug accidentally or deliberately destroyed. The Investigator must retain all unused or expired study drug supplies until the study monitor has confirmed the accountability records. If site policy prohibits holding study drug supplies for monitor review, then a copy of the standard operating procedure (SOP) for processing drug returns must be provided to the Sponsor.

To ensure adequate records, all study drugs (encorafenib, binimetinib, cetuximab, irinotecan and FOLFIRI) will be accounted for on a drug accountability inventory form as instructed by the Sponsor. Refer to the Pharmacy Manual for details on how to process all unused or expired study supplies.

6.7 Treatment Compliance

Patients receiving encorafenib and binimetinib, if applicable, should be instructed to bring their study drug supply and bottles, if applicable, to the site at each study visit. Compliance will be evaluated at each visit by review of patient diary entries, an accounting of returned study drug (i.e., encorafenib and binimetinib, if applicable) and patient interviews.

Cetuximab, irinotecan and FOLFIRI will be administered per protocol in the clinic by study personnel. Information regarding individual study drug infusions is to be documented as described in Section 6.3.2, Section 6.3.3 and Section 6.3.4, respectively.

7.0 STUDY PROCEDURES AND ASSESSMENTS

The procedures and assessments that will be conducted during this study are described in this section in narrative form and are presented by study visit in Section 8.0 and summarized in Table 25 (Prescreening and Screening Phases), Table 26 (Safety Lead-in and Triplet Arm), Table 27 (Doublet Arm) and Table 28 (Control Arm).

Patients in the Control arm who are determined to be eligible for treatment crossover (see Sections 5.2.3 and 8.2.1) will initiate study activities and assessments such that the first day of the crossover treatment regimen equates to Cycle 1 Day 1 on the Schedule of Events for the triplet regimen (Table 26).

Written informed consent (Molecular Prescreening, Screening and Crossover informed consents) must be granted by each patient prior to the initiation of any study procedure or assessment (other than those considered standard of care).

7.1 Prescreening/Screening Assessments and Procedures

7.1.1 *BRAF* Testing

Patients will be eligible for the study based on identification of a *BRAF*^{V600E} mutation in the tumor as determined by the central laboratory as part of Molecular Prescreening for the trial or by a local assay result obtained any time prior to Screening (see Figure 7). Only polymerase chain reaction (PCR) and next generation sequencing (NGS)-based local assays results will be acceptable. If the patient is enrolled based on local assay results, the *BRAF* mutation status must be confirmed by the central laboratory no later than 30 days from first dose of study treatment.

In cases where there is discordance between the local assay and central laboratory results, or if the central laboratory is not able to confirm presence of a *BRAF*^{V600E} due to inadequate or poor sample condition within 30 days of initiating study therapy, patients may only continue treatment if there is no clinical indication of deterioration or disease progression and the investigator determines that the patient is deriving benefit. In such instances, patients must be informed that the *BRAF* mutation status is unconfirmed and must sign a separate ICF that includes this information and describes alternative treatment options.

Central laboratory *BRAF* mutation tests with a definitive result (positive or negative) cannot be repeated to resolve a discordant result. Patients whose sample is determined to be inadequate or who have an indeterminate result on central testing may have samples resubmitted for testing. Lack of *BRAF*^{V600E} confirmation by the central laboratory may be due to discordance between the local assay and central laboratory results (potential false positive local assay results), or due to inadequate or poor sample condition for central testing (indeterminate results). If at any time in the study there is lack of *BRAF*^{V600E} confirmation in a total of 37 patients (6% of the total planned enrollment of the randomized portion of the trial) or discordance between the local assay

and the central laboratory in 18 patients (3% of the total planned enrollment), all subsequent patients will be required to have *BRAF*^{V600E} determined by the central laboratory for enrollment (i.e., local *BRAF* testing will no longer be accepted for trial eligibility). Information regarding sites and laboratories associated with discordant results will be maintained and results from laboratories with more than 1 prior discordant result will not be accepted for patient enrollment. Sites with more than 2 randomized patients having indeterminate results after initiation of protocol version 6 will be required to enroll all subsequent patients based only on central laboratory assay results.

Tumor samples previously determined to be *BRAF*^{wt} by local assessment may be submitted to the central laboratory. In particular, tumors with clinicopathological features of *BRAF* mutations such as right colon tumors, poorly differentiated, mucinous, or signet-ring carcinomas, or tumors metastasized to the peritoneum (Yokota et al. 2011), should be considered for testing by central laboratory regardless of the results of prior local *BRAF* mutation testing.

Figure 7: BRAF Testing

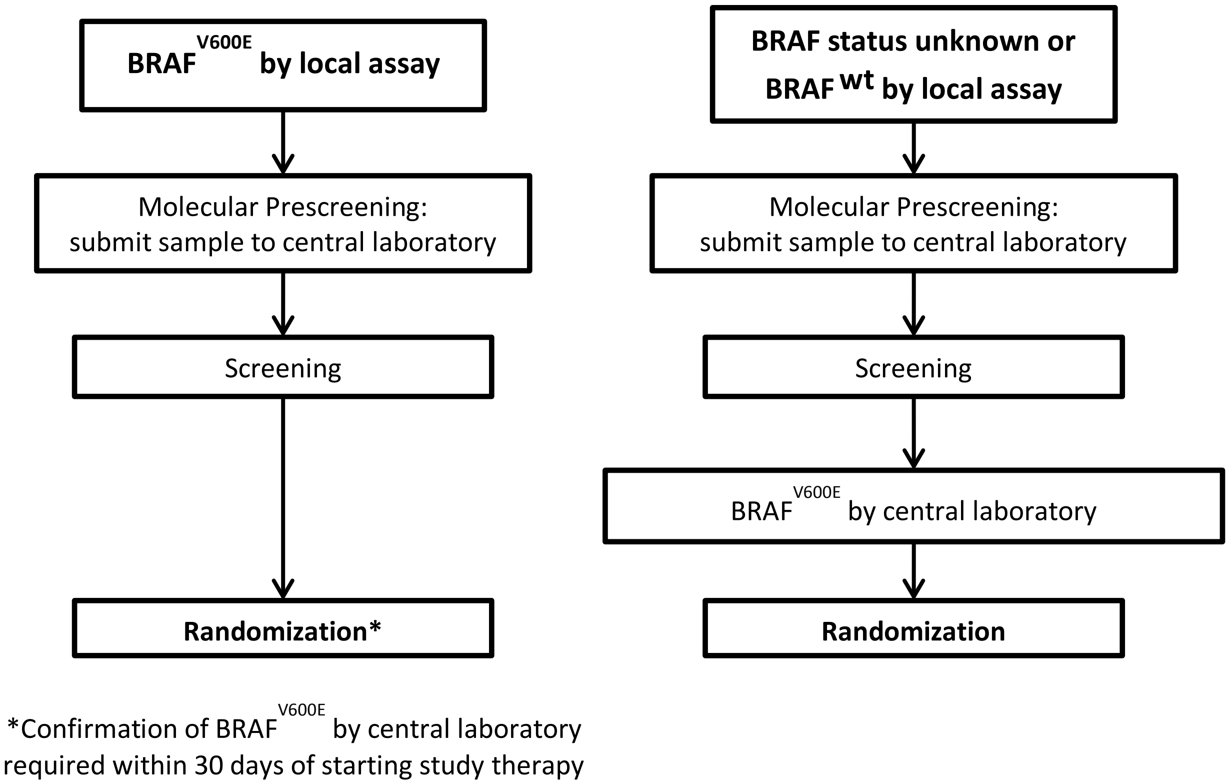

7.1.2 Molecular Prescreening

The molecular prescreening can be performed at any time prior to Screening (see Section 5.2.1). An informed consent must be signed prior to any molecular prescreening procedure for

confirmation of *BRAF*^{V600E} status. If an archival tumor sample is not available, a fresh tumor biopsy must be obtained. In patients with documented *BRAF*^{V600E} status determined by local assay prior to the study, when possible, the same tissue source should be submitted to the central laboratory in order to minimize the potential for discordance. Information regarding tissue specimen requirements, sample handling and shipment will be provided in the Laboratory Manual.

Patients will be registered for the study using IWRS after the prescreening informed consent is obtained. Refer and comply with detailed guidelines in the IWRS manual.

7.1.3 Screening

Patients, with documented *BRAF*^{V600E} status determined by the central laboratory during Molecular Prescreening or by local assay prior to this study, must sign a Screening ICF before additional screening procedures to determine eligibility for participation in the study are performed. A copy of the ICF will be given to the patient or their legal representative. The date that informed consent was obtained must be documented in source documents.

Patient eligibility will be verified against the inclusion and exclusion criteria once all screening procedures are completed. The eligibility check will be embedded in the IWRS system.

7.1.4 Information Collected for Screen Failures

Patients who provide informed consent (i.e., via the Prescreening ICF or the Screening ICF) but are not enrolled/randomized for any reason will be considered screen failures.

Date of informed consent and review of inclusion/exclusion criteria will be collected for screen failures as well as any AEs or SAEs possibly related to a study procedure during the screening period and any medications used to treat those AEs or SAEs (see Section 10.9 for SAE reporting details). For molecular prescreening failures, results of local *BRAF* assay available at Prescreening or Screening, AEs or SAEs possibly related to a study procedure will be reported.

7.1.5 Patient Demographics and Other Baseline Characteristics

Demographics, prior medications/therapies/procedures that were administered/conducted within 28 days prior to Day 1, current medications, diagnosis and extent of tumor, baseline tumor mutation status including site of disease, and details of prior antineoplastic treatments including number of prior metastatic regimens and prior irinotecan will be recorded. Past and present medical history considered by the Investigator to be significant will also be recorded.

7.2 Safety Assessments

7.2.1 Adverse Events

Adverse events will be assessed by direct observation and patient interviews on an ongoing basis. Patients should be questioned using non-leading questions. The severity of AEs will be evaluated using the NCI CTCAE, v.4.03. All AEs that occur from initiation of study drug through 30 days after the last dose of study drug must be recorded on the AE eCRF, regardless of causal assessment.

Definitions and reporting of AEs are described in detail in Section 10.0.

7.2.2 Clinical Laboratory Tests

Laboratory parameters to be assessed are listed in Table 20.

Table 20: Summary of Clinical Laboratory Tests

Hematology	Chemistry	Urinalysis	Coagulation
Basophils<br>Eosinophils<br>Hematocrit<br>Hemoglobin<br>Lymphocytes<br>Monocytes<br>Neutrophils/ANC<br>Platelets<br>RBC<br>WBC	ALT<br>AST<br>Bilirubin (total and direct)[a]<br>Albumin<br>Alkaline phosphatase<br>Bicarbonate (CO_2)<br>BUN/urea<br>CA19-9<br>Calcium<br>Chloride<br>CK[b]<br>CEA<br>Creatinine<br>Glucose<br>LDH<br>Magnesium<br>Potassium<br>Sodium<br>Total protein<br>Troponin<br>Uric acid	Blood<br>Glucose<br>Ketones<br>Leukocytes<br>pH<br>Protein	PT or INR<br>aPTT<br><br>**Others**<br><br>At Screening only:<br>• Hepatitis B surface antigen<br>• Hepatitis C antibody<br>• CRP<br><br>If applicable:<br>• Serum pregnancy test<br>• LH, FSH and/or estradiol<br>• Serum CK isoenzymes[b]<br>• Myoglobin

Abbreviations: ALT = alanine aminotransferase; ANC = absolute neutrophil count; AST = aspartate aminotransferase; BUN = blood urea nitrogen; CA19-9 = cancer antigen 19-9; CEA = carcinoembryonic antigen; CK = creatine kinase; CRP = C-reactive protein; FSH = follicle-stimulating hormone; INR = International Normalized Ratio; LDH = lactate dehydrogenase; LH = luteinizing hormone; pH = hydrogen ion concentration; PT = prothrombin time; aPPT = activated partial thromboplastin time; RBC = red blood cell(s); WBC = white blood cell(s).

[a] Direct bilirubin will be measured at screening only if total bilirubin values are abnormal; the result will be used to calculate indirect bilirubin level for purposes of determining eligibility to participate in the study.

[b] For Grade 2 total CK that is also $\geq$ 3 × ULN or asymptomatic Grade 3 total CK: measure CK, CK isoenzymes and myoglobin in blood or urine weekly for 3 weeks. If total CK remains above the grade that led to the increased monitoring, continue to assess CK, CK isoenzymes and myoglobin, along with regularly scheduled clinical chemistry assessments, until normalization or improvement to Grade 1. When CK is elevated, ensure patient is adequately hydrated.

A central laboratory will be used to analyze all blood and urine samples collected throughout the study for hematology, coagulation, clinical chemistry and urinalysis safety laboratory tests. Details regarding the collection of blood and urine samples and shipment to the central laboratory will be outlined in the Laboratory Manual.

At Screening, laboratory results from the central laboratory should be used by the Investigator to determine the patient's eligibility for the study. If a particular central laboratory result is not available, a local laboratory test result for that particular analyte may be utilized to determine a patient's eligibility to participate in the study. Local laboratory test results may also be used when results are time-sensitive (e.g., for patient safety or adjustment of study drug dose or schedule). Part of the sample obtained for local laboratory analysis should be sent to the central laboratory for analysis. Local laboratory results obtained during the study will not be captured in the eCRF unless they are needed to clarify why a treatment decision was made or an AE was recorded.

Additional clinical laboratory tests may be obtained at any time during the study at the Investigator's discretion.

7.2.2.1 Hematology, Coagulation and Clinical Chemistry

Blood samples for the hematology, coagulation and chemistry laboratory tests listed in Table 20 will be collected at the time points specified in Table 25 (Screening Phase), Table 26 (Safety Lead-in and Triplet Arm), Table 27 (Doublet Arm) and Table 28 (Control Arm).

Blood sample collections occurring on dosing days must be performed prior to study drug administration. Laboratory test results required to make decisions regarding potential dose modifications (as specified in Section 6.4.1, Section 6.4.2, Section 6.4.3 and Section 6.4.4, respectively) should be reviewed prior to study treatment administration.

7.2.2.2 Urinalysis

Urine samples for the laboratory tests listed in Table 20 will be collected at the time points specified in Table 25 (Screening Phase), Table 26 (Safety Lead-in and Triplet Arm), Table 27 (Doublet Arm) and Table 28 (Control Arm).

All urine collections occurring on dosing days must be performed prior to study treatment administration.

7.2.2.3 Pregnancy and Assessments of Fertility

All females of childbearing potential are required to undergo a serum pregnancy assessment at Screening and EOT and local urine pregnancy assessments at time points specified in Table 25 (Screening Phase), Table 26 (Safety Lead-in and Triplet Arm), Table 27 (Doublet Arm) and Table 28 (Control Arm). Female patients of nonchildbearing potential (as defined in Section 5.3.1) do not require pregnancy tests. Any positive pregnancy test will result in immediate cessation of study treatment administration. The serum pregnancy assessments conducted at Screening and EOT should be submitted to the central laboratory.

Female patients who have undergone female sterilization using oophorectomy alone will have a blood sample for measurement of LH, FSH, and/or estradiol as described in Section 5.3.1.

All urine collections for pregnancy tests occurring on dosing days must be performed and assessed prior to study treatment administration.

7.2.3 Vital Signs

Vital signs (blood pressure, pulse and temperature, as appropriate) will be measured per institutional standards at the time points specified in Table 25 (Screening Phase), Table 26 (Safety Lead-in and Triplet Arm), Table 27 (Doublet Arm) and Table 28 (Control Arm).

All vital sign measurements occurring on dosing days must be performed prior to study treatment administration. Any treatment-emergent abnormal findings will be recorded as AEs.

7.2.4 Physical Examination

Physical examinations will be performed by trained medical personnel at the time points specified in Table 25 (Screening Phase), Table 26 (Safety Lead-in and Triplet Arm), Table 27 (Doublet Arm) and Table 28 (Control Arm).

At Screening, the physical examination should include the examination of general appearance, skin, neck (including thyroid), eyes, ears, nose, throat, lungs, heart, abdomen, back, lymph nodes, extremities, vascular and neurological. If indicated based on medical history and/or symptoms, rectal, external genitalia, breast and pelvic examinations will be performed.

For subsequent visits, the physical examinations should be targeted as clinically indicated.

Body weight will be measured as part of the physical examination. Height will be measured only at Screening. All physical examinations occurring on dosing days must be performed prior to study treatment administration. Any treatment-emergent abnormal findings will be recorded as AEs.

7.2.4.1 Body Surface Area

For cetuximab, irinotecan and FOLFIRI dosing, patients' BSA will be calculated at the start of each cycle. The most recent patient weight available should be used to calculate BSA in order to determine the appropriate dose for that cycle. If the patient's weight at the beginning of each cycle varies by >10% from the previous cycle, the dose must be recalculated.

In calculating the BSA, actual height and weight should be used. In patients with BSA > 2.2 m^2, either actual BSA or 2.2 m^2 may be used. As per ASCO guidelines, any of the established formulas may be used to calculate BSA (Griggs et al. 2012).

7.2.5 Ophthalmic Assessments

7.2.5.1 Routine Testing

Full ophthalmic examination, including best corrected visual acuity for distance testing, automated visual field testing, slit lamp examination, intraocular pressure and dilated fundoscopy with attention to retinal abnormalities, especially retinal pigment epithelial detachment (RPED), serous detachment of the retina and RVO, will be performed by an ophthalmologist at the time points specified in Table 25 (Screening Phase), Table 26 (Safety Lead-in and Triplet Arm) and Table 27 (Doublet Arm). Patients randomized to the Control Arm will not have routine ophthalmic examinations. For all patients, ophthalmic assessments may be performed more frequently per standard of care or if clinically indicated for evaluation of any visual signs or symptoms.

7.2.5.2 Additional Testing

Patients with clinical suspicion of retinal abnormalities (i.e., RPED, serous detachment of the retina, RVO, photopsia, metamorphopsia, impairment of visual acuity, etc.), **must** complete at least one of the following additional assessments:

- For non-vascular abnormalities: OCT of the macula (spectral domain OCT recommended)
- For vascular abnormalities: fluorescein angiography of the central 30 degrees.

Images/results of the ophthalmic examinations (at a minimum, OCT and/or fluorescein angiography) should be sent to the study site and be maintained in the patient's source document file. These images/results may be requested to be sent to the Sponsor or designee.

7.2.6 Dermatologic Evaluations

For patients receiving encorafenib or encorafenib + binimetinib, dermatologic evaluations will be performed at the site by the Investigator to monitor for the possible development of keratoacanthoma (KA) and/or squamous cell carcinoma (SCC), as these have been reported to occur with selective BRAF inhibitor treatment (Flaherty et al. 2010, Kefford et al. 2010, Robert et al. 2011). This assessment can be done predose or postdose and will be performed at the time points specified in Table 25 (Screening Phase), Table 26 (Safety Lead-in and Triplet Arm) and Table 27 (Doublet Arm).

In case of occurrence of KA or SCC, patients will undergo complete surgical excision of the skin lesion following institutional standards. Dermatologic evaluations should be performed by a dermatologist as clinically indicated.

7.2.7 Electrocardiograms

Standard single 12-lead ECGs will be performed at the time points specified in Table 25 (Screening Phase), Table 26 (Safety Lead-in and Triplet Arm), Table 27 (Doublet Arm) and Table 28 (Control Arm). A triplicate ECG (3 serial ECGs conducted within approximately 5 to 10 minutes total time) will only be performed predose on Cycle 1 Day 1.

Prior to performing the 12-lead ECG, patients should rest in the supine position for at least 5 minutes. The ECG measurement performed at the Screening Visit will be used to determine eligibility. The mean of the triplicate ECG measurements recorded pre-morning dose on Cycle 1 Day 1 will serve as the patient's Baseline value for all postdose comparisons. The ECG measurement at any time point should be used for AE grading and recommended dose modifications.

When an ECG is to be performed at the same time point as a blood collection, the ECG is to be performed first.

Interpretation of the tracing should be made by a qualified physician and documented in the eCRF. Clinically significant abnormalities present when the patient signed the Screening informed consent should be reported in the eCRF. New or worsened clinically significant findings occurring after the Screening informed consent must be recorded in the eCRF.

7.2.8 Echocardiogram/Multi-gated Acquisition Scans

Cardiac ejection fraction will be assessed by transthoracic ECHO or MUGA scans at the time points specified in Table 25 (Screening Phase) and Table 26 (Safety Lead-in and Triplet Arm). The same method should be used throughout the study. Patients who develop signs/symptoms of congestive heart failure at any point during the study are required to have an evaluation of LVEF measurement by ECHO or MUGA.

7.2.9 ECOG Performance Status

Assessment of ECOG PS (Table 21) will be performed at the time points specified in Table 25 (Screening Phase), Table 26 (Safety Lead-in and Triplet Arm), Table 27 (Doublet Arm) and Table 28 (Control Arm).

ECOG PS should be obtained on the scheduled day, even if study treatment is being held.

Table 21: Eastern Cooperative Oncology Group (ECOG) Performance Status Scale

0	Fully active, able to carry on all predisease performance without restriction.
1	Restricted in physically strenuous activity but ambulatory and able to carry out work of a light or sedentary nature, e.g., light housework, office work.
2	Ambulatory and capable of all self-care but unable to carry out any work activities. Up and about more than 50% of waking hours.
3	Capable of only limited self-care, confined to bed or chair more than 50% of waking hours.
4	Completely disabled. Cannot carry on any self-care. Totally confined to bed or chair.
5	Dead

7.3 Pharmacokinetic and Biomarker Blood Sampling Assessments

During the course of the study, blood sampling for safety monitoring will be consistent with standard-of-care. Additional blood samples that will be collected over the course of the study for PK and biomarker analyses are shown in Table 22. If a patient receiving any of the treatments defined in this protocol experiences an AE that results in an unscheduled visit or meets the criteria for an SAE (Section 10.0), a blood sample for measurement of plasma concentrations of drug-related analytes should be collected, if feasible, if less than 24 hours have elapsed since the last dose of study drug.

Table 22: Blood Collection Volumes for Pharmacokinetic and Biomarker Assessments

Sample Type	Sample Volume (mL)	Number of Samples						
		Screening	Safety Lead-in Portion	Phase 3 Portion		Follow-up (EOT)	
				Baseline	During treatment		
**PK**							**Total Volume (mL) for PK for Each Arm**
**Safety Lead-in[a]**	8	0	10	-	-	0	**80**
**Triplet and Doublet Arms[b]**	8	0	-	0	4	0	**32**
**Control Arm[c]**	4	0	-	0	4	0	**16**
**Biomarkers**	10	2[d]	0	3[e]	1 sample every cycle for as long as patient is in study	2[f]	**70 plus 10 per treatment cycle**
**MSI Biomarker**	5	0	0	1	0	0	**5**
					**Total Volume (mL) for Biomarkers /Arm**		
							**75 plus 10 per treatment cycle**
**TOTAL**					**Total Blood Volume in Study (mL)**		
					**Safety Lead-in**		**155 plus 10 per treatment cycle**
					**Triplet and Doublet Arms**		**107 plus 10 per treatment cycle**
					**Control Arm**		**91 plus 10 per treatment cycle**

**Sample Type**	**Sample Volume (mL)**	**Number of Samples**					
		**Screening**	**Safety Lead-in Portion**	**Phase 3 Portion**		**Follow-up (EOT)**	
				**Baseline**	**During treatment**		

Abbreviations: EOT = end of treatment; MSI = microsatellite instability; PK = pharmacokinetic.

[a] PK for patients in the Safety Lead-in: at each timepoint, 2 blood samples (~4 mL) will be collected. The first sample will be processed to plasma for analysis of encorafenib, binimetinib and the metabolite of binimetinib. The second sample will be processed to serum for analysis of cetuximab.

[b] PK for the Triplet and Doublet Arms, PK samples will be collected from the first ~50 patients enrolled. At each timepoint, 2 blood samples (~4 mL) will be collected. The first sample will be processed to plasma for analysis of encorafenib, binimetinib and the metabolite of binimetinib. The second sample will be processed to serum for analysis of cetuximab.

[c] PK for the Control Arm, PK samples will be collected from the first ~100 patients enrolled. At each timepoint 1 sample (~4 mL) will be collected and processed to serum for analysis of cetuximab.

[d] Blood collections for biomarker assessments obtained during Screening will include C-reactive protein and tumor markers.

[e] Blood collections (~10 mL) for biomarker assessments obtained at Baseline (predose Cycle 1 Day 1) will include tumor markers, companion diagnostic assay and predictive markers of activity.

[f] Blood collections for biomarker assessments obtained at End of Treatment will include tumor markers (~10 mL) and markers of resistance (at the time of disease progression) (~10 mL).

Blood sample collection times for the Safety Lead-in portion of the study are presented in Table 23 and for the Randomized Phase 3 portion of the study in Table 24. See Section 7.4 for additional information on sample collection.

Table 23: Pharmacokinetic Sampling Times for the Safety Lead-in Portion of Study ARRAY-818-302 for the Safety Lead-in Patients Who Will Receive Encorafenib, Cetuximab and Binimetinib

	**Cycle 1 Day 1**					**Cycle 2 Day 1**				
Time after dosing on designated dosing days (h)	0 (predose)	1	2	4	6	0 (predose)	1	2	4	6
PK Sample[a]	X	X	X	X	X	X	X	X	X	X

Abbreviations: h = hours; PK = pharmacokinetic.

[a] PK samples will be collected from all Safety Lead-in patients on Cycle 1 Day 1 and Cycle 2 Day 1 only at the following time points: predose (just prior to dose of encorafenib/binimetinib) and pre-infusion (just prior to infusion of cetuximab), post dose/post-infusion at 1 h (± 10 min), 2 h (± 10 min), 4 h (± 30 min), 6 h (± 30 min).

[b] Blood samples for encorafenib/binimetinib PK will be collected predose and postdose at the times indicated above and processed to plasma. Blood samples for cetuximab PK will be collected pre-infusion and post-infusion at the times indicated above and processed to serum.

Table 24: Pharmacokinetic Sampling Times for the Randomized Phase 3 Portion of Study ARRAY-818-302 for a Subset of the Triplet, Doublet, and Control Arms

	Cycle 1 Day 1		Cycle 2 Day 1	
Time after dosing on designated dosing days (h)	2	6	0 (predose)	2
PK Sample<br>Triplet Arm[a]<br>Doublet Arm[b]<br>Control Arm[c]	X	X	X	X

Abbreviations: h = hours; PK = pharmacokinetic.

[a] Triplet Arm: PK samples will be collected for the first ~50 patients enrolled in the Triplet Arm on Cycle 1 Day 1 postdose (encorafenib/binimetinib) and post-infusion (cetuximab) at 2 h (± 10 min) and 6 h (± 30 min). PK samples will be collected on Cycle 2 Day 1 predose (just prior to encorafenib/binimetinib dose) and pre-infusion (just prior to infusion of cetuximab) and postdose/post-infusion at 2 h (± 10 min). Blood samples for encorafenib/binimetinib PK will be processed to plasma.
Blood samples for cetuximab PK will be processed to serum.

[b] Doublet Arm: PK samples will be collected from the first ~50 patients enrolled in the Doublet Arm on Cycle 1 Day 1 postdose of encorafenib and post-infusion of cetuximab at 2 h (± 10 min) and 6 h (± 30 min). PK samples will be collected on Cycle 2 Day 1 predose of encorafenib and pre-infusion of cetuximab, and postdose/post-infusion at 2 h (± 10 min). Blood samples for encorafenib PK will be processed to plasma.Blood samples for cetuximab PK will be processed to serum.

[c] Control Arm: Cetuximab PK samples will be collected from the first ~100 patients enrolled in the Control Arm on Cycle 1 Day 1 post-infusion at 2 h (± 10 min) and 6 h (± 30 min). PK samples will be collected on Cycle 2 Day 1 just prior to infusion and post-infusion at 2 h (± 10 min). Blood samples for cetuximab PK will be processed to serum.

7.4 Pharmacokinetics Assessment

Blood samples (~4 mL each) for plasma PK analysis of encorafenib, binimetinib and/or the active metabolite of binimetinib (AR00426032), as appropriate, will be collected from all patients in the Safety Lead-in and from a subset of approximately 50 patients who are first to enroll in any site in the Triplet and Doublet Arms of the Phase 3 portion of the study, as specified in Table 26 (Safety Lead-in and Triplet Arm) and Table 27 (Doublet Arm).

A second blood sample (~4 mL) will be collected from these patients for serum PK analysis of cetuximab from the Safety Lead-in, Triplet Arm and Doublet Arm and from approximately 100 patients who are first to enroll in the Control Arm, as specified in Table 26 (Safety Lead-in and Triplet Arm), Table 27 (Doublet Arm) and Table 28 (Control Arm).

Study visits for PK sampling should be scheduled in the morning so that a proper predose and postdose PK blood samples can be collected. On the PK visit days, the morning doses of encorafenib and binimetinib, if applicable, should be taken at the study site, only after collecting the predose PK sample for plasma. Predose sample collection for serum concentration of cetuximab should occur before the beginning of cetuximab infusion. Predose sampling

information should include the dose amount taken and the date and approximate time of the most recent previous dose of binimetinib and encorafenib, or cetuximab, if applicable (except Day 1). Postdose sampling information should include the date and approximate time of the morning dose, including the dose amount taken. Postdose infusion information for cetuximab should include dose, start time, and infusion duration. Infusion interruptions should also be documented. Except for the Cycle 1 Day 1 PK samples, which have to be obtained on the scheduled day, other PK samples may be obtained ±1 day from the scheduled date. A graphic representation of blood PK sampling for plasma and serum is provided in Figure 8.

Figure 8: Graphic Representation of Pharmacokinetic Sampling Plan

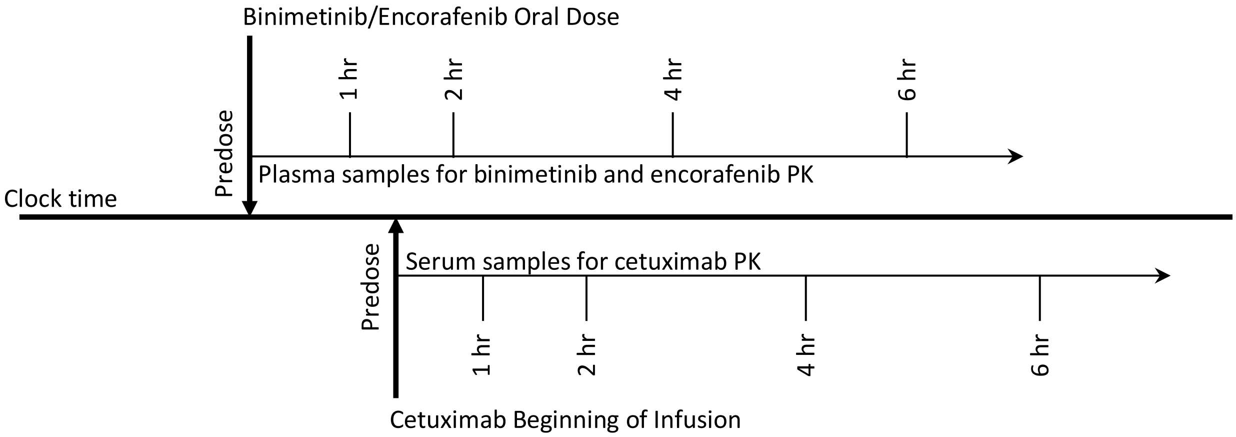

If vomiting occurs within 4 hours following oral study drug administration on the day of PK sampling, no additional study treatment should be taken in an effort to replace the material that has been vomited.

Blood should be collected in accordance with institutional guidelines. Any sampling problems should be noted in the eCRF and on appropriate source documentation. Complete instructions for sample processing, handling and shipment will be provided in the Laboratory Manual.

Compartmental, noncompartmental and population PK analyses will be performed, as appropriate, based on analyte plasma concentration data from blood samples from all patients. In addition, exploratory analyses on remaining plasma samples for analytes related to administered drugs or their effects may be performed using a non-validated, semi-quantitative, or qualitative liquid chromatography tandem mass spectrometry (LC-MS/MS) method, if deemed appropriate.

7.5 Biomarker Assessments

7.5.1 C-reactive Protein

A blood sample (up to ~10 mL) for analysis of C-reactive protein (CRP) will be collected during Screening (Table 25).

Complete instructions for sample collection, processing, handling and shipment to the central laboratory will be provided in the Laboratory Manual.

7.5.2 Tumor Markers (CEA and CA19-9)

Blood samples (up to ~10 mL) for analysis of tumor markers CEA and CA 19-9 will be collected at the time points specified in Table 25 (Screening Phase), Table 26 (Safety Lead-in and Triplet Arm), Table 27 (Doublet Arm) and Table 28 (Control Arm).

Complete instructions for sample collection, processing, handling and shipment to the central laboratory will be provided in the Laboratory Manual.

7.5.3 Other Assessment (Companion Diagnostic Assay)

An in vitro diagnostic assay will be developed in accordance with FDA's Quality System Regulations and Design Control requirements. Any tumor samples remaining after testing for *BRAF*^{V600E} and *RAS*^{wt} status will be used to assess the concordance between the results for *BRAF*^{V600E} status obtained using the central laboratory assay to determine eligibility for the study and the companion diagnostic assay which will be submitted for post-marketing approval. A blood sample (up to ~10 mL) obtained predose at baseline may also be used for this purpose.

7.5.4 Microsatellite Instability

Tumor samples and blood samples (~5 mL) will be collected predose at baseline and analyzed for microsatellite instability (MSI) status by immunohistochemistry. The tumor sample for MSI testing will be the same sample (fresh or archival) used for determination of *BRAF* mutation status for patient eligibility purposes.

Complete instructions for sample collection, processing, handling and shipment to the central laboratory will be provided in the Laboratory Manual.

If necessary, MSI status will be confirmed by PCR analysis.

7.5.5 Predictive Biomarkers of Activity

Blood samples (~10 mL) will be collected at baseline and analyzed for potential genomic or proteomic predictive markers of activity.

Complete instructions for sample collection, processing, handling and shipment to the central laboratory will be provided in the Laboratory Manual.

Further exploratory biomarker research may be conducted on collected blood (including PK) samples. These studies would extend the search for other potential biomarkers relevant to the effects of the drugs given in combination in this study, and/or prediction of these effects, and/or resistance to the treatment, and/or safety, and these additional investigations would be dependent upon clinical outcome, reagent and sample availability.

7.5.6 *RAS* Wild-Type Status

Tumor formalin-fixed paraffin embedded (FFPE) sample will be collected from all patients at screening for the determination of *KRAS* mutation status by a central laboratory.

7.5.7 Other Exploratory Assessments

7.5.7.1 Other Biomarkers

Any remaining PK plasma samples may be used for detecting CYP3A4 inhibition by measuring beta-hydroxycholesterol levels as a biomarker.

7.5.7.2 Markers of Resistance

Blood samples (~10 mL) will be taken at the time of disease progression for all patients. These samples will be stored for possible analysis of circulating tumor deoxyribonucleic acid (ctDNA) to assess possible markers of resistance.

Complete instructions for sample collection, processing, handling and shipment to the central laboratory will be provided in the Laboratory Manual.

7.5.7.3 Optional Tumor Sample Collection at Disease Progression

Patients will be offered the option to provide fresh tumor samples at the time of progression. Patients will be asked to sign a separate ICF prior to the procedure. These optional biopsy collections should be offered to patients with accessible lesions. Accessible lesions are defined as tumor lesions which are easily biopsied. Lesions with the greatest change in dimensional size are the recommended lesions to be excised at the time of progressive disease (PD). Whenever possible, biopsies at progression should be performed within 3 days of study drug discontinuation. The tissue from these biopsies will be used for potential pharmacodynamic biomarker assessments and to determine possible mechanisms of resistance. Possible pharmacodynamic biomarkers that will be investigated include phosphorylated ERK (pERK), p27, DUSP6, CyclinD1, and the cell proliferation marker Ki67 among possible others. Resistance mechanisms to be explored could include the acquisition of genetic alterations that result in the activation of MAPK pathway signaling or cause the activation of compensatory

growth pathways (e.g., the loss of PTEN, or activating mutations in PIK3a). Alternatively, methods to examine possible epigenetic causes of resistance (e.g., increased expression of specific kinases that allow MAPK bypass) could also be explored.

7.5.8 Retention of Samples for Future Analysis

If the patient agrees, and in accordance with local laws, any tumor (archival or fresh) samples remaining after determination of *BRAF*^{V600E} status and use for companion diagnostic assay development may be stored for up to 2 years after the completion of the study. The samples may be further analyzed to address scientific questions and/or development of biological tests related to administration of encorafenib + cetuximab ± binimetinib and/or cancer. The decision to perform such exploratory biomarker research studies would be based on outcome data from this study or from new scientific findings related to the drug class or disease, as well as reagent and assay availability.

The Sponsor will be the exclusive owner of any data and discoveries resulting from this study.

7.6 Efficacy Assessments

Tumor response will be evaluated locally by the Investigator and retrospectively by BICR according to RECIST, v1.1 (Appendix 6).

All potential sites of tumor lesions will be assessed at the time points specified in Table 25 (Screening Phase), Table 26 (Safety Lead-in and Triplet Arm), Table 27 (Doublet Arm) and Table 28 (Control Arm):

At screening, the following should be performed:

- A CT scan with IV contrast of chest, abdomen and pelvis is the preferred technique. If there is concern about radiation exposure, an MRI may be used instead of a CT.
- In patients with a history of asymptomatic brain metastases, a brain MRI or CT scan
- If clinically indicated, a whole body bone scan (i.e., if bone metastases are suspected or known at baseline). Sites may use a whole body bone imaging method per their local standard of care (e.g., Tc99m bone scan, fluorodeoxyglucose-positron emission tomography [FDG-PET], sodium fluoride-positron emission tomography [NaF PET] scan or whole-body bone MRI). Skeletal lesions identified on a whole body bone scan at baseline, which are not visible on the chest, abdomen, or pelvis CT (or MRI) scan should be imaged at baseline using localized CT, MRI, or X-ray.

Every effort must be made to assess each lesion that is measured at screening by the same method throughout the study so that the comparison is consistent.

All post-screening assessments should be performed every 6 weeks (±7 days) from the date of randomization (or from first dose for Safety Lead-in) until disease progression for the first 24 weeks of treatment, then every 12 weeks thereafter until disease progression, withdrawal of consent, initiation of subsequent anticancer therapy, patient is lost to follow-up, death or defined end of study (Section 4.6). The interval should remain unchanged from study start for patients who cross over from the Control arm to receive the triplet regimen.

Regardless of whether study treatment is discontinued, the following should be performed:

- Chest, abdomen, and pelvis CT (or MRI) scans
- Brain MRI or CT scan, if metastases were documented at baseline
- Skeletal lesions identified at baseline should continue to be imaged at subsequent scheduled visits using localized CT, MRI, or X-ray (using the same method used at baseline for all visits for any given lesion). After baseline, whole body bone scans need not be repeated, unless clinically indicated.
- Additional imaging evaluations may be performed if there is symptomatic evidence suggesting the possibility of disease progression based on clinical symptoms or physical examination at any time.

If off-schedule imaging evaluations are performed or if progression is suspected, every effort should be made to perform subsequent imaging evaluations in accordance with the original imaging schedule.

If a patient has disease progression during the first 5 weeks of study treatment, that patient's BOR is PD. The BOR in patients who have been participating in the study for at least 5 weeks and who have not experienced a CR or PR per RECIST will be SD.

All CT scans should be performed with IV contrast. If a patient is known to have a medical contraindication to the contrast agent or develops a contraindication during the study, a CT scan without IV contrast of the chest and MRI with IV contrast, if possible, of the abdomen and pelvis may be performed. A CT scan of the brain, preferably with IV contrast, may be performed if MRI is contra-indicated.

Chest X-ray or ultrasound should not be used for tumor response assessments in this study.

Any lesions that have been subjected to loco-regional therapies (e.g., radiotherapy, ablation, etc.) should not be considered measurable, unless they have clearly progressed since the therapy. Previously treated lesions that have not progressed should be considered non-measurable and therefore, assessed as non-target lesions.

While FDG-PET scans are not required for this study, sites may perform combined PET/CT scans per their local standard of care, provided the CT is of similar diagnostic quality as CT performed without PET, including the use of oral and IV contrast media. If acquired according to local standard of care, FDG-PET may be relied upon to document PD in accordance with RECIST.

When possible, each center should have a designated radiologist responsible for the interpretation of scans and response evaluations for study patients. At a minimum, a single radiologist should perform all evaluations for an individual patient.

For patients who cross over from the Control arm to receive the triplet regimen, the last tumor assessment prior to treatment crossover will be considered the baseline assessment for determination of disease progression.

7.6.1 BICR Evaluation of Imaging Data

All imaging data acquired for efficacy purposes (e.g., CT/MRI scans) will be transmitted to an imaging vendor for BICR review. Image transmission to the imaging vendor should be performed according to the imaging vendor manual.

The images will be read by readers who are blinded to treatment assignment and to other clinical data as specified in the BICR charter. BICR review of imaging data will be performed retrospectively and will not be provided to Investigators for decisions regarding patient treatment.

Once patients cross over from the Control Arm to the triplet regimen, images for those patients will no longer be submitted for BICR review.

7.6.2 Patient-reported Outcome Assessments

Patient-reported outcome (PRO) assessments will be collected using the QoL questionnaires EORTC QLQ-C30, FACT-C, EQ-5D-5L and PGIC (Appendix 7) at the time points specified in Table 25 (Screening Phase), Table 26 (Safety Lead-in and Triplet Arm), Table 27 (Doublet Arm) and Table 28 (Control Arm). These questionnaires will be used to explore patient-reported outcome measures of health-related quality-of-life, functioning, cancer symptoms, and treatment-related side effects. The QLQ-C30, FACT-C, EQ-5D-5L and PGIC are recognized reliable and valid measures in the treatment of cancer patients (Aaronson et al, 1993, Rabin and de Charro 2001, and Mesa et al. 2013).

PRO assessments will not be collected in patients after treatment crossover.

The questionnaires should be administered to patients in the patients’ local language at the beginning of the study visit prior to receiving any study treatment, prior to any other study

assessment or consultation with the Investigator, and prior to being informed of their current disease status.

Attempts should be made to collect all questionnaires for all patients, including those who discontinue prior to the End of Treatment visit. However, if the patient refuses to complete the questionnaires, this should be documented in study source records. Patient refusal to complete study questionnaires is not a protocol deviation.

Patients should be given sufficient space and time to complete all study questionnaires, and all administered questionnaires should be reviewed for completeness. If missing responses are noted, patients should be encouraged to complete any missing responses.

Completed questionnaires, including both responses to the questions and any unsolicited comments written by the patient, should be reviewed by the Investigator or designee to ensure every question has been answered and that there is only 1 response for each question. If omissions or double responses occur, they should be brought to the attention of the patient. Investigators must not encourage the patient to change responses reported in questionnaires.

In addition, the completed questionnaires should be reviewed and assessed by the Investigator for responses which may indicate potential AEs or SAEs. This review should be documented in study source records.

8.0 SCHEDULE OF PROCEDURES AND ASSESSMENTS

The procedures and assessments that will be conducted during this study are described in this section by study visit, described in narrative form in Section 7.0, and presented in separate tables for: Prescreening and Screening Phases (Table 25), Safety Lead-in and Triplet Arm (Table 26), Doublet Arm (Table 27) and Control Arm (Table 28).

Patients in the Control arm who are determined to be eligible for treatment crossover (see Section 8.2.1) will initiate study activities and assessments such that the first day of the crossover treatment regimen equates to Cycle 1 Day 1 on the Schedule of Events for the triplet regimen (Table 26).

For all visits, there is a general ±3-day window on assessments to take into account scheduling over public or religious holidays, if not explicitly specified otherwise. For PK sampling, the samples may be obtained ±1 day from the scheduled date (except for PK sampling on Cycle 1 Day 1). For imaging assessments, a ±7-day window is allowed. The first post-baseline tumor assessment should be performed at 6 weeks (±7-day window permitted) from date of randomization (or from first dose for Safety Lead-in).

Table 25: Schedule of Events for Prescreening and Screening

Procedure/Assessment	Prescreening Phase<br>Any time prior to Screening	Screening Phase<br>Day<br>–28 to–1	Randomization[b]
Molecular Prescreening informed consent	X		
Molecular prescreen inclusion/exclusion criteria	X		
Tumor sample (archival or fresh) for *BRAF*^{V600E} and *RAS*^{wt} status, companion diagnostic assay and MSI testing to be submitted for central laboratory testing	X		
Register in IWRS	X		
Screening informed consent		X	
Screening inclusion/exclusion criteria		X	
Demographics		X	
Medical history		X	
Prior medications/therapies/procedures		X	
Height		X	
Weight		X	
Vital signs		X	
Physical examination		X	
ECOG PS		X	
EORTC QLQ-C30, FACT-C, EQ-5D-5L, PGIC[b]		X	
ECG		X	
Ophthalmic examination		X	
Dermatologic examination		X	
ECHO/MUGA		X	

Procedure/Assessment	Prescreening Phase	Screening Phase	Randomization[b]
	Any time prior to Screening	Day –28 to–1	
Pregnancy test[a]		X	
LH, FSH and/or estradiol[a]		X	
Hepatitis B surface antigen, Hepatitis C antibody		X	
Hematology		X	
Clinical chemistry		X	
Coagulation		X	
Urinalysis		X	
Tumor evaluation (CT scan, MRI)		X	
Blood sample for CRP		X	
Blood sample for tumor markers (CEA, CA19-9)		X	
Concomitant medications/therapies		X	
Randomize patient via IWRS[c]			X
Determination of study drug for patients in the Control Arm[d]			X

Abbreviations: CA19-9 = carbohydrate antigen 19-9; CEA = carcinoembryonic antigen; CRP = C-reactive protein; CT = computed tomography; ECG = electrocardiogram; ECHO = echocardiogram; ECOG PS = Eastern Cooperative Oncology Group Performance Status; EORTC QLQ-C30 = European Organization for Research and Treatment of Cancer Quality of Life Questionnaire; EQ-5D-5L = EuroQol-5D-5L; FACT-C = Functional Assessment of Cancer Therapy-Colon Cancer; FSH = follicle-stimulating hormone; IWRS = interactive web response system; LH = luteinizing hormone; MRI = magnetic resonance imaging; MSI = microsatellite instability; MUGA = multi-gated acquisition; PGIC = Patient Global Impression of Change.

[a] Serum test for women of childbearing potential only (LH, FSH and/or estradiol measurements, if applicable, as described in Section 5.3.1).

[b] Does not apply to patients participating in Safety Lead-in. Only for randomized Phase 3 patients.

[c] Patients must receive their first dose of study drug within 5 days of randomization.

[d] For the Phase 3 Control Arm, the Investigator will determine the best treatment option for the patient (irinotecan/cetuximab or FOLFIRI/cetuximab). The IWRS will be notified of the treatment decision.

Table 26: Schedule of Events for Safety Lead-in, Triplet Arm, and Crossover Patients (Encorafenib + Binimetinib + Cetuximab)

Procedure/Assessment (± 3-day window for procedures/assessments)	Treatment Phase								Follow-up Phase		
	Cycle 1				Subsequent Cycles						
	Day 1	Day 8	Day 15	Day 22	Day 1	Day 8	Day 15	Day 22	End of Treatment	30-Day Follow-up[m]	Survival Follow-up
Verify inclusion/exclusion criteria	X[b,n]										
Medical history from screening to Day 1	X										
Weight	X[c]				X				X	X	
BSA	X[c]				X						
Vital signs	X[n]	X	X	X	X	X	X	X	X	X	
Physical examination	X[c]				X				X	X	
ECOG PS	X[c,n]				X				X	X	
EORTC QLQ-C30, FACT-C, EQ-5D-5L, PGIC[a,o]	X				X				X	X	
ECG	X[d,n]		X		X[d]				X	X	
Ophthalmic examination	X[n]				X[e]				X	X[e]	
Dermatologic examination	X[n]				X[f]				X	X	
ECHO/MUGA	X[n]				X[g]				X		
Pregnancy test[h]	X[c,n]				X				X[h]	X	
Hematology	X[c,n]		X	X	X				X	X	
Clinical chemistry	X[c,n]		X		X				X	X	
Coagulation	X[c,n]				X				X	X	
Urinalysis	X[c]				X				X	X	
Tumor evaluation (CT scan, MRI)	Every 6 weeks (±7 days) from randomization date (or from first dose for Safety Lead-in) for the first 24 weeks, then every 12 weeks[i] (±7 days)										

Procedure/Assessment (± 3-day window for procedures/assessments)	Treatment Phase								Follow-up Phase		
	Cycle 1				Subsequent Cycles						
	Day 1	Day 8	Day 15	Day 22	Day 1	Day 8	Day 15	Day 22	End of Treatment	30-Day Follow-up[m]	Survival Follow-up
PK blood samples	X[j]				X[j]						
Blood sample for companion diagnostic assay development	X										
Blood sample for tumor markers (CEA, CA19-9)	X				X				X		
Blood sample for predictive biomarkers	X										
Blood sample for MSI testing	X										
Blood sample for markers of resistance									X[k]		
Tumor biopsy (optional)									X[k]		
**Encorafenib** dispense (plus dosing diary)	X				X						
Assess encorafenib compliance					X				X		
**Binimetinib** dispense (plus dosing diary)	X				X						
Assess binimetinib compliance					X				X		
**Cetuximab** IV infusion	X	X	X	X	X	X	X	X			
Adverse Events	Assess continuously										
Concomitant medications/therapies	Assess continuously										
Survival status										X	Every 3 months[l]
Documentation of subsequent anticancer therapy										X	Every 3 months[l]
Documentation of PD after subsequent anticancer therapy[a]											Every 3 months[l]

Abbreviations: BSA = body surface area; CA19-9 = carbohydrate antigen 19-9; CEA = carcinoembryonic antigen; CT = computed tomography; ECG = electrocardiogram; ECHO = echocardiogram; ECOG PS = Eastern Cooperative Oncology Group Performance Status; EORTC QLQ-C30 = European

Organization for Research and Treatment of Cancer Quality of Life Questionnaire; EQ-5D-5L = EuroQol-5D-5L; FACT-C = Functional Assessment of Cancer Therapy-Colon Cancer; IV = intravenous(ly); MRI = magnetic resonance imaging; MSI = microsatellite instability; MUGA = multi-gated acquisition; PD = progressive disease; PGIC = Patient Global Impression of Change; PK = pharmacokinetic(s).

[a] Does not apply to patients participating in Safety Lead-in; only for randomized Phase 3 patients.

[b] Inclusion/exclusion criteria are to be verified before the first dose of study drug (including crossover treatment).

[c] Procedure does not have to be repeated if performed within 72 hours prior to Cycle 1 Day 1 (i.e., first day of dosing).

[d] Electrocardiograms are to be performed in triplicate predose on Cycle 1 Day 1 (conducted within approximately 5 to 10 minutes total time), followed by a single ECG at 2.0 (± 0.5) hours after administration of encorafenib and binimetinib and before the start of the cetuximab infusion. Single ECGs are to be performed on Cycle 2 Day 1 predose and at 2.0 (+ 0.5) hours after administration of encorafenib and binimetinib and before the start of the cetuximab infusion. Single ECGs are to be performed predose at remaining time points. Electrocardiograms should be performed prior to PK and PD blood collection at equivalent nominal timepoints.

[e] Ophthalmic examinations are to be performed on Cycle 2 Day 1 and then every 8 weeks from Cycle 2 Day 1 (i.e., on Day 1 of Cycles 4, 6, 8…) and EOT. An ophthalmic examinations at the 30-day follow up is only required if there was a clinically significant abnormality noted at EOT.

[f] Dermatologic examinations are to be performed every 8 weeks from Cycle 1 Day 1 (i.e., on Day 1 of Cycles 3, 5, 7…).

[g] ECHO/MUGA scans are to be performed on Cycle 2 Day 1 and Cycle 5 Day 1, then every 12 weeks and EOT

[h] Local urine test for women of childbearing potential only, except for EOT visit which will be a central laboratory serum pregnancy test.

[i] Tumor evaluations are to be performed every 6 weeks (±7 days) from randomization (or from first dose for Safety Lead-in) for the first 24 weeks, then every 12 weeks (±7 days) until PD is determined. For crossover patients, the schedule of tumor evaluations should remain unchanged from study start.

[j] PK samples will be collected during Cycle 1 and Cycle 2 only. See Table 23 for the PK sampling times for the Safety Lead-in and Table 24 for the Phase 3 portion of the study.

[k] Blood biomarker sample and optional tumor sample will be requested only for patients that discontinue study due to disease progression.

[l] Patients will be contacted by telephone for collection of information during the survival follow-up period. This may be conducted more frequently as needed.

[m] Following the 30-day follow up, when clinically appropriate, it is recommended patients be monitored with physical examinations, dermatological examinations and chest CT scans for cutaneous and non-cutaneous secondary malignancies for up to 6 months after the last encorafenib dose or until initiation of another antineoplastic therapy.

[n] In crossover patients, must be performed within 28 days prior to starting the triplet regimen. Assessments performed during the treatment or follow-up period within 28 days prior to treatment crossover may serve as screening assessments and need not be repeated.

[o] Does not apply to crossover patients.

Table 27: Schedule of Events for Doublet Arm (Encorafenib + Cetuximab)

Procedure/Assessment (± 3-day window for procedures/assessments)	Treatment Phase								Follow-up Phase		
	Cycle 1				Subsequent Cycles						
	Day 1	Day 8	Day 15	Day 22	Day 1	Day 8	Day 15	Day 22	End of Treatment	30-Day Follow-up[k]	Survival Follow-up
Verify inclusion/exclusion criteria	X[a]										
Medical history from screening to Day 1	X										
Weight	X[b]				X				X	X	
BSA	X[b]				X						
Vital signs	X	X	X	X	X	X	X	X	X	X	
Physical examination	X[b]				X				X	X	
ECOG PS	X[b]				X				X	X	
EORTC QLQ-C30, FACT-C, EQ-5D-5L, PGIC	X				X				X	X	
ECG	X[c]		X		X[c]				X	X	
Ophthalmic exam[d]									X		
Dermatologic exam	X				X[e]				X	X	
Pregnancy test[f]	X[b]				X				X[f]	X	
Hematology	X[b]	-	X	X	X				X	X	
Clinical chemistry	X[b]		X		X				X	X	
Coagulation	X[b]				X				X	X	
Urinalysis	X[b]				X				X	X	
Tumor evaluation (CT scan, MRI)	Every 6 weeks (±7 days) from randomization date for the first 24 weeks, then every 12 weeks[g] (±7 days)										
PK blood samples	X[h]				X[h]						
Blood sample for companion diagnostic assay	X										

Procedure/Assessment (± 3-day window for procedures/assessments)	Treatment Phase								Follow-up Phase		
	Cycle 1				Subsequent Cycles				End of Treatment	30-Day Follow-up[k]	Survival Follow-up
	Day 1	Day 8	Day 15	Day 22	Day 1	Day 8	Day 15	Day 22			
development											
Blood sample for tumor markers (CEA, CA19-9)	X				X				X		
Blood sample for predictive biomarkers	X										
Blood sample for MSI testing	X										
Blood sample for markers of resistance									X[i]		
Tumor biopsy (optional)									X[i]		
**Encorafenib** dispense (plus dosing diary)	X				X						
Assess encorafenib compliance					X				X		
**Cetuximab** IV infusion	X	X	X	X	X	X	X	X			
Adverse Events	Assess continuously										
Concomitant medications/therapies	Assess continuously										
Survival status										X	Every 3 months[j]
Documentation of subsequent anticancer therapy										X	Every 3 months[j]
Documentation of PD after subsequent anticancer therapy											Every 3 months[j]

Abbreviations: BSA = body surface area; CA19-9 = carbohydrate antigen 19-9; CEA = carcinoembryonic antigen; CT = computed tomography; ECG = electrocardiogram; ECOG PS = Eastern Cooperative Oncology Group Performance Status; EORTC QLQ-C30 = European Organization for Research and Treatment of Cancer Quality of Life Questionnaire;EQ-5D-5L = EuroQol-5D-5L; FACT-C = Functional Assessment of Cancer Therapy-Colon Cancer; IV = intravenous(ly); MRI = magnetic resonance imaging; MSI = microsatellite instability; PD = progressive disease; PGIC = Patient Global Impression of Change; PK = pharmacokinetic(s).

[a] Inclusion/exclusion criteria are to be verified before the first dose of study drug.

[b] Procedure does not have to be repeated if performed within 72 hours prior to Cycle 1 Day 1 (i.e., first day of dosing).

[c] Electrocardiograms are to be performed in triplicate predose on Cycle 1 Day 1 (conducted within approximately 5 to 10 minutes total time), followed by a single ECG at
2.0 (± 0.5) hours after administration of encorafenib and before the start of the cetuximab infusion. Single ECGs are to be performed on Cycle 2 Day 1 predose and at
2.0 (+ 0.5) hours after administration of encorafenib and before the start of the cetuximab infusion. Single ECGs are to be performed predose at remaining time points. Electrocardiograms should be performed prior to PK and PD blood collection at equivalent nominal timepoints.

[d] Ophthalmic examinations are to be performed as needed during on-study treatment phase and at EOT

[e] Dermatologic examinations are to be performed every 8 weeks from Cycle 1 Day 1 (i.e., on Day 1 of Cycles 3, 5, 7…).

[f] Local urine test for women of childbearing potential only, except for EOT visit which will be a central laboratory serum pregnancy test.

[g] Tumor evaluations are to be performed every 6 weeks (±7 days) from randomization for the first 24 weeks, then every 12 weeks (±7 days) until PD is determined

[h] PK samples will be collected during Cycle 1 and Cycle 2 only. See Table 24 for the PK sampling times for the Phase 3 portion of the study.

[i] Blood biomarker sample and optional tumor sample will be requested only for patients that discontinue study due to disease progression.

[j] Patients will be contacted by telephone for collection of information during the survival follow-up period. This may be conducted more frequently as needed.
Following the 30-day follow up, when clinically appropriate, it is recommended patients be monitored with physical examinations, dermatological examinations and chest CT scans for cutaneous and non-cutaneous secondary malignancies for up to 6 months after the last encorafenib dose or until initiation of another antineoplastic therapy.

Table 28: Schedule of Events for Control Arm (Irinotecan/Cetuximab or FOLFIRI/Cetuximab)

Procedure/Assessment (± 3-day window for procedures/assessments)	Treatment Phase								Follow-up Phase		
	Cycle 1				Subsequent Cycles						
	Day 1	Day 8	Day 15	Day 22	Day 1	Day 8	Day 15	Day 22	End of Treatment[l]	30-Day Follow-up[l]	Survival Follow-up
Verify inclusion/exclusion criteria	X[a]										
Medical history from screening to Day 1	X										
Weight	X[b]				X				X	X	
BSA	X[b]				X						
Vital signs	X	X	X	X	X	X	X	X	X	X	
Physical examination	X[b]				X				X	X	
ECOG PS	X[b]				X				X	X	
EORTC QLQ-C30, FACT-C, EQ-5D-5L, PGIC	X				X				X	X	
ECG	X[c]		X		X[c]				X	X	
Pregnancy test[d]	X[b]				X				X[d]	X	
Hematology	X[b]		X	X	X		X[e]		X	X	
Clinical chemistry	X[b]		X		X				X	X	
Coagulation	X[b]				X				X	X	
Urinalysis	X[b]				X				X	X	
Tumor evaluation (CT scan, MRI)	Every 6 weeks (±7 days) from randomization date for the first 24 weeks, then every 12 weeks[f] (±7 days)										
PK blood sample	X[g]				X[g]						
Blood sample for companion diagnostic assay development	X										
Blood sample for tumor markers (CEA, CA19-9)	X				X				X		

Procedure/Assessment (± 3-day window for procedures/assessments)	Treatment Phase								Follow-up Phase		
	Cycle 1				Subsequent Cycles				End of Treatment[l]	30-Day Follow-up[l]	Survival Follow-up
	Day 1	Day 8	Day 15	Day 22	Day 1	Day 8	Day 15	Day 22			
Blood sample for predictive biomarkers	X										
Blood sample for MSI testing	X										
Blood sample for markers of resistance									X[h]		
Tumor biopsy (optional)									X[h]		
**Irinotecan** IV infusion[i]	X		X		X		X				
**5-FU and FA** IV infusion[j]	X		X		X		X				
**Cetuximab** IV infusion	X	X	X	X	X	X	X	X			
Adverse Events	Assess continuously										
Concomitant medications/therapies	Assess continuously										
Survival status										X	Every 3 months[k]
Documentation of subsequent anticancer therapy										X	Every 3 months[k]
Documentation of PD after subsequent anticancer therapy											Every 3 months[k]

Abbreviations: BSA = body surface area; CA19-9 = carbohydrate antigen 19-9; CEA = carcinoembryonic antigen; CT = computed tomography; ECG = electrocardiogram; ECOG PS = Eastern Cooperative Oncology Group Performance Status; EORTC QLQ-C30 = European Organization for Research and Treatment of Cancer Quality of Life Questionnaire; EQ-5D-5L = EuroQol-5D-5L; FA = folinic acid; FACT-C = Functional Assessment of Cancer Therapy-Colon Cancer; 5-FU = 5-fluorouracil; IV = intravenous(ly); MRI =-magnetic resonance imaging; MSI = microsatellite instability; PD = progressive disease; PGIC = Patient Global Impression of Change.

[a] Inclusion/exclusion criteria are to be verified before the first dose of study drug.

[b] Procedure does not have to be repeated if performed within 72 hours prior to Cycle 1 Day 1 (i.e., first day of dosing).

[c] Electrocardiograms are to be performed in triplicate predose on Cycle 1 Day 1 (conducted within approximately 5 to 10 minutes total time). Single ECGs are to be performed predose at remaining time points. Electrocardiograms should be performed prior to predose PK and PD blood collection at equivalent nominal timepoints.

[d] Local urine test for women of childbearing potential only, except for EOT visit which will be a central laboratory serum pregnancy test.

[e] A blood sample for hematology is to be drawn on Day 15 of cycles subsequent to Cycle 1 for patients only while they are receiving irinotecan.

[f] Tumor evaluations are to be performed every 6 weeks (±7 days) from randomization for the first 24 weeks, then every 12 weeks (±7 days) until PD is determined.

[g] PK samples will be collected during Cycle 1 and Cycle 2 only. See Table 24 for the PK sampling times for the Phase 3 portion of the study.

[h] Blood biomarker sample and optional tumor sample will be requested only for patients that discontinue study due to disease progression.

[i] Irinotecan is administered in both control groups (irinotecan plus cetuximab or FOLFIRI).

[j] 5-FU and FA are administered in the FOLFIRI plus cetuximab control group. The initial 5-FU dose is given as a bolus (not to exceed 15 minutes) on Days 1 and 15, followed by continuous IV infusion over 46-48 hours (2 days) or as per institutional standards.

[k] Patients will be contacted by telephone for collection of information during the survival follow-up period. This may be conducted more frequently as needed.

[l] These visits are not required for patients crossing over to the triplet regimen.

8.1 Molecular Prescreening

Patient may undergo molecular prescreening at any time prior to randomization based on eligibility criteria described in Section 5.2.1.

- Obtain written informed consent for molecular testing
- Register the patient into the IWRS for screening and obtain a patient study number to be used throughout the study
- If tumor has previously been determined to be *BRAF*^{V600E} mutant by local assay, confirm that it was done using PCR or NGS
- Obtain and send archival tumor specimen to the central laboratory, as soon as possible following the signing of the Molecular Prescreening informed consent for confirmation of histology and *BRAF*^{V600E} (see Section 7.1.1). If an adequate archival tumor sample (minimum of 6 slides; optimally up to 15 slides) is not available, a tumor biopsy is required. The same tumor sample will be used for the companion diagnostic assay and MSI testing. If the central laboratory determines that the sample is inadequate for analysis, a second sample may be submitted.

8.2 Screening

All screening procedures to determine eligibility for study participation must be performed within specific time windows before the first dose of study treatment. Eligibility is determined using results of screening assessments performed before the first dose of study treatment and up to and including Day 1. Patients may be re-screened once at the discretion of the Investigator and/or individual assessments may be repeated, as appropriate. If a particular assessment is repeated, the results obtained closest to the first dose of study treatment should be used to assess eligibility.

At the site, the Investigator will maintain a log for all screened patients (including patients who fail screening after providing written informed consent) and all enrolled/randomized patients. If the patient has not been molecularly prescreened, obtain written informed consent for molecular testing.

Screening: Within 28 days prior to randomization (or from first dose for Safety Lead-in):

- Obtain Screening informed consent (must be obtained prior to performance of any study-specific tests or evaluations that are not considered standard of care)
- Record demographic information (age, race, ethnicity).
- Administer QOL questionnaires (EORTC QLQ-C30, FACT-C, EQ-5D-5L, PGIC)

- Record current and past medical history
- Document prior anticancer treatments
- Record all medications/treatments that were administered/conducted within 28 days prior to Day 1
- Complete physical examination including measured height and body weight
- Obtain full ophthalmic examination
- Obtain full dermatologic examination
- Assess vital signs (blood pressure, pulse and temperature)
- Obtain single ECG
- ECHO/MUGA
- Collection of blood samples for the following:
    - Hematology, coagulation, chemistry
    - Serum pregnancy test (females of childbearing potential only)
    - LH, FSH and/or estradiol (females who are post-menopausal)
    - Hepatitis B surface antigen
    - Hepatitis C antibody
    - CRP
    - Tumor markers (CEA and CA19-9)
- Obtain urine sample for urinalysis
- Complete tumor assessments with appropriate radiological scans to document all suspected sites of disease, measurable or non-measurable, as defined by RECIST
- Assess ECOG PS
- Verify all inclusion/exclusion criteria
- In the Phase 3 portion of the study, declare whether the patient will receive irinotecan or FOLFIRI if randomized to Control Arm

- Randomize patient via IWRS (or enroll for Safety Lead-in).

8.2.1 Screening for Treatment Crossover

Within 28 days prior to initiation of crossover treatment:

- Obtain crossover informed consent (must be obtained prior to performance of any study-specific tests or evaluations that are not considered standard of care)
- Obtain full ophthalmic examination
- Obtain full dermatologic examination
- Assess vital signs (blood pressure, pulse and temperature)
- Obtain single ECG
- ECHO/MUGA
- Collection of blood samples for the following:
  - Hematology, coagulation, chemistry
  - Serum pregnancy test (females of childbearing potential only)
- Assess ECOG PS
- Verify all Crossover inclusion/exclusion criteria
- Enroll for Crossover treatment

Note: Assessments performed during the treatment or follow-up period within 28 days prior to treatment crossover may serve as screening assessments and need not be repeated.

8.3 Cycle 1

Following determination of eligibility and enrollment in treatment crossover, patients will initiate study activities and assessments such that the first day of the crossover treatment regimen equates to Cycle 1 Day 1 on the Schedule of Events for the triplet regimen (Table 26).

8.3.1 Cycle 1 Day 1

In the Phase 3 portion of the study, patients must receive the first dose of study drug within 5 days of the randomization date.

If the following study procedures were not performed within 3 days before Day 1, they must be repeated on Cycle 1 Day 1 (i.e., first day of dosing):

- Complete physical examination, including measurement of body weight
- Calculate BSA
- Obtain blood samples for hematology, coagulation, clinical chemistry
- Obtain urine sample for urinalysis and for pregnancy test for females of childbearing potential
- Assess ECOG PS

All of the following study procedures are to be performed on Cycle 1 Day 1:

- Randomized Phase 3 patients only: Administer QOL questionnaires (EORTC QLQ-C30, FACT-C, EQ-5D-5L, PGIC)
- Record medical history from Screening to Day 1
- Assess vital signs (blood pressure, pulse and temperature)
- Obtain 3 serial, resting and supine 12-lead ECGs conducted within approximately 5 to 10 minutes total time
- Safety Lead-in/Triplet Arm and Doublet Arm only: dermatologic examination
- Verify inclusion/exclusion criteria
- Collect blood samples for the following:
    - Tumor markers (CEA and CA19-9)
    - Predictive biomarker assessment
    - Companion diagnostic assay development
    - MSI testing
    - Plasma PK samples:
        - Safety Lead-in (all patients): Predose and 1, 2, 4, and 6 hours following dosing of encorafenib and binimetinib

        - Phase 3 (first ~50 patients enrolled in the Doublet and Triplet Arms): obtain at 2 and 6 hours following administration of encorafenib and/or binimetinib
    - Serum PK samples:
        - Safety Lead-in: Predose and 1, 2, 4 and 6 hours after the beginning of the cetuximab infusion
        - Phase 3 (all Arms, first ~50 patients enrolled in the Doublet and Triplet Arms and the first ~100 patients enrolled in the Control Arm): obtain at 2 and 6 hours following after the beginning of the cetuximab infusion
- Safety Lead-in/Triplet Arm only: administer dose of encorafenib and binimetinib with water
- Doublet Arm only: administer dose of encorafenib with water
- All Arms: administer cetuximab premedication according to institutional standards
- All Arms: administer cetuximab as an IV infusion
- Control Arm only: administer irinotecan as an IV infusion either alone or in combination with 5-FU as an IV bolus and infusion and FA as an IV infusion
- Safety Lead-in/Triplet Arm and Doublet Arm only: obtain a single ECG 2.0 (+0.5) hours following administration of encorafenib or encorafenib + binimetinib and before the start of the cetuximab infusion. Electrocardiograms should be performed prior to the 2-hour postdose PK blood collection.
- Document concomitant medications/therapies
- Assess AEs
- Safety Lead-in/Triplet Arm only: dispense a 4-week supply of encorafenib and binimetinib along with a monthly diary. Review dosing instructions with patient.
- Doublet Arm only: dispense a 4-week supply of encorafenib along with a monthly diary. Review dosing instructions with patient

8.3.2 Cycle 1 Day 8

- Assess vital signs (blood pressure, pulse and temperature)
- All Arms: administer cetuximab premedication according to institutional standards

- All Arms: administer cetuximab as an IV infusion
- Review concomitant medications/therapies
- Assess AEs since previous visit

8.3.3 Cycle 1 Day 15

- Assess vital signs (blood pressure, pulse and temperature)
- Obtain blood sample for hematology and chemistry
- Obtain single ECG (predose)
- Safety Lead-in/Triplet Arm only: administer dose of encorafenib and binimetinib with water
- Doublet Arm only: administer dose of encorafenib with water
- All Arms: administer cetuximab premedication according to institutional standards
- All Arms: administer cetuximab as an IV infusion
- Control Arm only: administer irinotecan as an IV infusion either alone or in combination with 5-FU as an IV bolus and infusion and FA as an IV infusion
- Review concomitant medications/therapies
- Assess AEs since previous visit

8.3.4 Cycle 1 Day 22

- Assess vital signs (blood pressure, pulse and temperature)
- Obtain blood sample for hematology
- Safety Lead-in/Triplet Arm only: administer dose of encorafenib and binimetinib with water
- Doublet Arm only: administer dose of encorafenib with water
- All Arms: administer cetuximab premedication according to institutional standards
- All Arms: administer cetuximab as an IV infusion
- Review concomitant medications/therapies

- Assess AEs since previous visit

8.4 Subsequent Cycles

Tumor assessments (i.e., appropriate radiological scans to document all suspected sites of disease) are to be performed (Section 7.6) every 6 weeks (±7 days) from the date of randomization (or from first dose for Safety Lead-in) for the first 24 weeks of treatment, then every 12 weeks thereafter until disease progression, withdrawal of consent, initiation of subsequent anticancer therapy, patient is lost to follow-up, or death, regardless of whether study treatment is discontinued.

8.4.1 Subsequent Cycles Day 1

- Administer QOL questionnaires (EORTC QLQ-C30, FACT-C, EQ-5D-5L, PGIC)
- Complete physical examination including weight
- Calculate BSA
- Assess vital signs (blood pressure, pulse and temperature)
- Assess ECOG PS
- Safety Lead-in/Triplet Arm and Doublet Arm only: Cycle 2 only: obtain a single ECG predose and 2.0 hours (+0.5 hour) after administration of encorafenib or encorafenib + binimetinib and before the start of the cetuximab infusion
- Control Arm: obtain single ECG
- Safety Lead-in/Triplet Arm only: ophthalmic examination at Cycle 2 Day 1 and then every 8 weeks (Day 1 Cycles 4, 6, 8 …)
- Doublet Arm only: ophthalmic examination only as needed while on study
- Safety Lead-in/Triplet Arm and Doublet Arm only: dermatologic examination every 8 weeks (i.e., Day 1 of Cycles 3, 5, 7 …)
- Safety Lead-in/Triplet Arm only: ECHO/MUGA Cycle 2 Day 1 and Cycle 5 Day 1, then every 12 weeks
- Collection of blood samples for the following:

- PK : **Cycle 2 only**:
  - Plasma PK samples:
    - Safety Lead-in (all patients): Predose and 1, 2, 4 and 6 hours following dose of encorafenib and binimetinib
    - Phase 3 (first ~50 patients enrolled in the Doublet and Triplet Arms): predose and 2 hours following administration of encorafenib and/or binimetinib
  - Serum PK samples:
    - Safety Lead-in (all patients): Predose and 1, 2, 4 and 6 hours after the beginning of the cetuximab infusion
    - Phase 3 (all Arms, first ~50 patients enrolled in the Doublet and Triplet Arms and the first ~100 patients enrolled in the Control Arm): predose and 2 hours following after the beginning of the cetuximab infusion
- Hematology, coagulation, and clinical chemistry
- Tumor markers (CEA and CA19-9)

- Obtain a urine sample for urinalysis and/or urine pregnancy testing for women of childbearing potential
- Safety Lead-in/Triplet Arm only: administer dose of encorafenib and binimetinib with water
- Doublet Arm only: administer dose of encorafenib with water
- All Arms: administer cetuximab premedication according to institutional standards
- All Arms: administer cetuximab as an IV infusion
- Control Arm only: administer irinotecan as an IV infusion either alone or in combination with 5-FU as an IV bolus and infusion and FA as an IV infusion
- Safety Lead-in/Triplet Arm and Doublet Arm only: assess compliance of encorafenib and/or binimetinib dosing and use of diary. Dispense a 4-week supply of encorafenib and/or binimetinib along with a monthly diary. Review patient instructions.
- Review concomitant medications/therapies
- Assess AEs since previous visit

8.4.2 Subsequent Cycles Day 8

- Assess vital signs (blood pressure, pulse and temperature)
- All Arms: administer dose of cetuximab premedication according to institutional standards
- All Arms: administer cetuximab as an IV infusion
- Review concomitant medications/therapies
- Assess AEs since previous visit

8.4.3 Subsequent Cycles Day 15

- Assess vital signs (blood pressure, pulse and temperature)
- Control Arm only: obtain blood sample for hematology
- All Arms: administer cetuximab premedication according to institutional standards
- All Arms: administer cetuximab as an IV infusion
- Control Arm only: administer irinotecan as an IV infusion either alone or in combination with 5-FU as an IV bolus and infusion and FA as an IV infusion
- Review concomitant medications/therapies
- Assess AEs since previous visit

8.4.4 Subsequent Cycles Day 22

- Assess vital signs (blood pressure, pulse and temperature)
- All Arms: administer cetuximab premedication according to institutional standards
- All Arms: administer cetuximab as an IV infusion
- Review concomitant medications/therapies
- Assess AEs since previous visit

8.5 End of Treatment Visit

At the time of study treatment discontinuation, the End of Treatment Visit should be completed for all patients as soon as possible after the last dose of study drug, and every effort should be made to perform the procedures listed below. This visit should take place as soon as possible and

≤ 14 days after the last dose of study treatment. An eCRF should be completed, giving the date and reason for stopping the study treatment. End of treatment visit is not considered as the end of the study. All patients will enter the follow-up period. If study drug is discontinued due to patient decision, the patient may then elect to continue with additional tumor assessments, if applicable.

- Administer QOL questionnaires (EORTC QLQ-C30, FACT-C, EQ-5D-5L, PGIC)
- Assess vital signs (blood pressure, pulse and temperature)
- Complete physical examination including measurement of body weight
- Safety Lead-in/Triplet Arm and Doublet Arm only: obtain full ophthalmic examination
- Safety Lead-in/Triplet Arm and Doublet Arm only: complete full dermatologic examination
- Assess ECOG PS
- Obtain single ECG
- Safety Lead-in/Triplet Arm only: ECHO/MUGA

Collection of blood samples for the following:

  - Tumor markers (CEA and CA19-9)
  - Hematology, coagulation, clinical chemistry
  - Serum pregnancy test for females of childbearing potential
  - Markers of resistance (for patients who discontinued study treatment due to disease progression only)

- Obtain urine sample for urinalysis
- Optional tumor biopsy (for patients who discontinued study treatment due to disease progression)
- Safety Lead-in/Triplet Arm and Doublet Arm only: assess compliance with encorafenib and/or binimetinib and use of diary.
- Document concomitant medications/therapies through last dose of study drug
- Assess AEs
- Safety Lead-in/Triplet Arm and Doublet Arm only: collect all unused study medication

- If patients withdraw consent, they should be asked if they are willing to be contacted via telephone for survival status. If patients refuse to be contacted by telephone (or, for patients in the Safety Lead-in, where informed consent cannot be obtained due to the patient being lost to follow-up or previous withdrawal of consent), attempts to determine survival status should be made via access to public records.

Note: this visit is not required for Control patients prior to crossing over to the triplet regimen.

8.6 Follow-up Visits

Tumor assessments (i.e., appropriate radiological scans to document all suspected sites of disease) are to be performed every 6 weeks (±7 days) from the date of randomization (or from first dose for Safety Lead-in) for the first 24 weeks of treatment, then every 12 weeks thereafter until disease progression, withdrawal of consent, initiation of subsequent anticancer therapy, patient is lost to follow-up, or death.

8.6.1 30-Day Safety Follow-up Visit

All patients will return for a 30-Day Safety Follow-up Visit approximately 30 days after the last dose of study drug, or prior to the initiation of subsequent anticancer therapy, whichever occurs first. Information related to AEs (including concomitant medication taken for ongoing AEs) and ongoing antineoplastic treatments will be collected for 30 days after the last dose of study drug. All AEs suspected to be related to study treatment should be followed up weekly, or as clinically indicated, until resolution or stabilization.

- Administer QOL questionnaires (EORTC QLQ-C30, FACT-C, EQ-5D-5L, PGIC)
- Assess vital signs (blood pressure, pulse and temperature)
- Complete physical examination including measurement of body weight
- Assess ECOG PS
- Obtain a single ECG
- Obtain blood samples for hematology, coagulation, clinical chemistry
- Obtain urine sample for urinalysis and for pregnancy test for females of childbearing potential
- Review concomitant medications/therapies
- Assess AEs

- Safety Lead-in/Triplet Arm only: Full ophthalmic examination, only if clinically significant abnormality noted at EOT
- Safety Lead-in/Triplet Arm and Doublet Arm only: Full dermatologic examination
- Determine survival status
- Document subsequent anticancer therapy

Note: this visit is not required for Control patients prior to crossing over to the triplet regimen.

8.6.2 Other Follow-up Visits

8.6.2.1 Visits for Tumor Assessments

Tumor assessments (i.e., appropriate radiological scans to document all suspected sites of disease) are to be performed (Section 7.6) every 6 weeks (±7 days) from the date of randomization (or from first dose for Safety Lead-in) for the first 24 weeks of treatment, then every 12 weeks thereafter until disease progression, withdrawal of consent, initiation of subsequent anticancer therapy, patient is lost to follow-up, or death, regardless of whether study treatment is discontinued.

After the 30-day safety follow-up visit, only new SAEs that are considered related to study drug should also be assessed and recorded.

8.6.2.2 Survival Follow-up (Safety Lead-in Patients)

After the 30-day safety follow-up visit, patients who provide informed consent for survival follow-up will undergo the following assessments every 3 months, or more frequently as needed, until withdrawal of consent, patient is lost to follow-up, death or defined end of study (Section 4.6):

- Record new SAEs that are considered related to study drug
- Record all subsequent anticancer therapies
- Determine survival status

If informed consent cannot be obtained due to the patient being lost to follow-up or previous withdrawal of consent, attempts to determine survival status should be made via access to public records as permitted by local laws.

8.6.2.3 Survival Follow-up (Randomized Phase 3 Patients)

After the 30-day safety follow-up visit, the assessments listed below should be performed every 3 months, or more frequently as needed, until withdrawal of consent, patient is lost to follow-up, death or defined end of study (Section 4.6):

- Record new SAEs that are considered related to study drug
- Record all subsequent anticancer therapies
- Document the date of disease progression following the initiation of subsequent therapies
- Determine survival status

If patients withdraw consent, they should be asked if they are willing to be contacted via telephone for survival status. If patients refuse to be contacted by telephone, attempts to determine survival status should be made via access to public records as permitted by local laws.

8.7 Collection of Data for Ongoing Patients Following Database Lock

If the primary objectives of the study have been met or the Sponsor decides to stop the study early, the database may be locked for the purpose of analyzing and reporting data. Patients may continue to receive study treatment per protocol beyond database lock if the Investigator and the Sponsor agree that patients' best interests are served by continuing to receive study treatment.

In such cases, appropriate safety information will continue to be captured per protocol and submitted to the Sponsor. The information to be collected may include treatment-related AEs and SAEs, laboratory results of special interest, study drug administration, patient status and date and reason(s) for study withdrawal.

9.0 STUDY DISCONTINUATION

9.1 Termination of the Study by the Sponsor

This study may be discontinued at any time due to safety concerns, failure to meet expected enrollment goals, administrative reasons or at the discretion of the Sponsor. Should the study be terminated prematurely, the Sponsor will provide written notification to all Investigators and regulatory authorities and will specify the reason(s) for early termination. The Investigator must inform the IRB promptly and provide the reason(s) for the termination.

9.2 Treatment Discontinuation for Individual Patients

Patients may withdraw their consent to participate in the study at any time for any reason without prejudice to their future medical care by the physician or at the institution. However, patients should be asked if they are willing to be contacted by telephone for monitoring of survival. If a patient withdraws consent, the date, stated reason for and level of consent withdrawal should be documented. Patient data collected up to the date of consent withdrawal will be included in the analyses. Any blood or tissue samples collected up to the date of withdrawal of consent will be analyzed.

Wherever possible, the tests and evaluations listed for the End of Treatment visit should be carried out and an effort should be made to continue follow-up. The Sponsor should be notified of all study withdrawals through the designated eCRFs in a timely manner.

Patients meeting any of the following criteria must discontinue study drug treatment:

- Withdrawal of consent (no further participation) - request patients to provide consent for further survival follow-up
- Patient decision to discontinue study treatment (but request agreement to return for end of treatment assessments, safety follow-up assessments and/or survival follow-up)
- Unacceptable AEs or failure to tolerate study drug
- Dose interruption of > 28 consecutive days in administration of encorafenib or binimetinib, or noncompliance with study drug administration consisting of 2 missed consecutive doses of irinotecan, 5-FU, or FA or > 4 missed consecutive doses of cetuximab due to an AE or clinically significant laboratory abnormality, unless judged by the Investigator and Sponsor Medical Monitor or designee to be in the best interest of the patient to continue treatment
- Changes in the patient’s condition or development of an intercurrent illness which renders the patient unacceptable for further treatment in the judgment of the Investigator

- Disease progression as defined by RECIST, v1.1 (continuation of treatment beyond progression permitted in special circumstances (defined in Section 4.5)
- Receipt of non-protocol-specified anticancer therapy for study indication (chemotherapy, biological therapy or radiation therapy that includes > 30% of the bone marrow reserve)
- Patient becomes pregnant or begins breastfeeding
- Significant protocol deviation that, in the opinion of the Investigator and/or Sponsor, renders the patient unsuitable for further study drug administration
- Lost to follow-up
- Death
- Termination of the study by the Sponsor (described in Section 9.1).

Patients meeting any of the following criteria may be discontinued from study drug treatment if, during the course of the study:

- Is found not to have met eligibility criteria; the patient would be discontinued if the investigator determines that the patient would not benefit from participation in the study due to eligibility deviation.
- Is found to have a tumor that is BRAFwt by the central laboratory
- Is noncompliant with study procedures or study drug administration in the opinion of the investigator (See Section 6.7).

9.3 Study Discontinuation for Individual Patients

The patient may be considered discontinued from the study for the following reasons:

- Withdrawal of consent for survival follow-up
- Lost to follow-up
- Death
- Termination of the study by the Sponsor (described in Section 9.1).
- Patients discontinuing treatment in Safety Lead-in

9.4 Replacement of Patients

In the Safety Lead-in phase of the study, patients who terminate participation in the study for any reason other than an AE or abnormal laboratory value unrelated to disease, disease progression, intercurrent illness or concomitant medications/therapies before completing at least 75% dose intensity (administered dose in mg/planned dose in mg) of the binimetinib, encorafenib or cetuximab doses in Cycle 1 will be considered ineligible for the safety assessment required for tolerability and will be replaced.

In the Phase 3 part of the study, randomized patients who discontinue prior to study completion will not be replaced.

All patients receiving at least 1 dose of study drug will be evaluated for safety.

10.0 SAFETY MONITORING: DEFINITIONS AND REPORTING

10.1 Adverse Event

An AE is any untoward medical occurrence, including the exacerbation of a pre-existing condition, in a patient administered a pharmaceutical product regardless of causality.

10.2 Events Related to Progression of Disease

Progression of malignancy (including fatal outcomes), if documented by use of appropriate method (for example, as per RECIST criteria for solid tumors) will be designated as progression of disease in the eCRF and should not be reported as a AEs or SAEs unless a causal relationship to study drug is suspected.

10.3 Adverse Events Related to Subsequent Anticancer Therapy

Adverse events specifically related to subsequent anticancer therapy are excluded from AE reporting, as are hospitalizations necessary for the administration of such therapy.

10.4 Clinical Laboratory Abnormalities

An abnormal laboratory value that is not associated with an already reported AE is to be recorded as an AE only if an action on the study drug is made as a result of the abnormality, if intervention for management of the abnormality is required, or at the discretion of the Investigator.

Laboratory abnormalities that meet the criteria for AEs should be followed until they have returned to normal or baseline, or per Investigator discretion.

10.5 Overdose

An overdose of study drug (whether symptomatic or asymptomatic) will be reported as an AE.

10.6 Assessment of Severity

The severity rating of an AE refers to its intensity. The severity of each AE will be categorized using the NCI CTCAE, v4.03. For any term that is not specifically listed in the CTCAE scale, intensity should be assigned a Grade of 1 through 5 using the following CTCAE guidelines:

Grade 1: Mild; asymptomatic or mild symptoms; clinical or diagnostic observations only; intervention not indicated

Grade 2: Moderate; minimal, local or noninvasive intervention indicated; limiting age-appropriate instrumental activities of daily living

Grade 3: Severe or medically significant but not immediately life-threatening; hospitalization or prolongation of hospitalization indicated; disabling; limiting self-care activities of daily living

Grade 4: Life-threatening consequences; urgent intervention indicated

Grade 5: Death related to AE

10.7 Assessment of Causality

Medical judgment should be used to determine the cause of the AE, considering all relevant factors such as (but not limited to) the underlying study indication, coexisting disease, concomitant medication, relevant history, pattern of the AE, temporal relationship to the study medication and de-challenge or re-challenge.

Yes (possibly, probably or definitely related): there is a reasonable possibility that the study drug caused the event; one or more of the following criteria apply:

- The event follows a reasonable temporal sequence from administration of study drug.
- The event could not be reasonably attributed to the known characteristics of the patient's clinical state, environmental or toxic factors or other modes of therapy administered to the patient.
- The event follows a known pattern of response to study drug.
- The event disappears or decreases on cessation or reduction in dose of the study drug. (It should be noted that in some situations an AE will not disappear or decrease in intensity upon discontinuation of study drug despite other clear indications of relatedness).
- The event reappears or worsens when the study drug is re-administered.

No (unlikely, probably not related or definitely not related): there is no reasonable possibility that the study drug caused the event; one or more of the following criteria apply:

- The event does not follow a reasonable temporal sequence from administration of study drug.
- The event could be reasonably attributed to the known characteristics of the patient's clinical state, concurrent illness, environment or toxic factors or other modes of therapy administered to the patient.
- The event does not follow a known pattern of response to study drug.

- The event does not disappear or decrease on cessation or reduction in dose of the study drug, and it does not reappear or worsen when the study drug is re-administered.

10.8 Assessment of Seriousness

An AE is considered "serious" if it results in any of the following outcomes:

- Results in death.
    - Death is an outcome of an SAE and not an SAE in itself. Death should only be reported as an SAE term when no additional information is known about a fatal event. When death is an outcome, the event(s) resulting in death should be reported (e.g., "pulmonary embolism" with a fatal outcome) and assigned severity Grade 5.
- Is immediately life-threatening (its occurrence places the patient at immediate risk of death. It does not include an event that, had it occurred in a more severe form, might have caused death)
- Requires inpatient hospitalization or prolongation of existing hospitalization.
    - Hospitalization includes any hospital admission, even if for less than 24 hours. The following do not meet hospitalization serious criteria: Preplanned or elective hospitalizations including social and/or convenience situations (e.g., respite care), emergency room visits or outpatient observation.
- Results in a persistent or significant incapacity or substantial disruption of the ability to conduct normal life functions.
- Is a congenital anomaly/birth defect.
- Based upon appropriate medical judgment, represents an important medical event that may jeopardize the patient or may require intervention to prevent one of the outcomes described above.

10.9 Reporting of Serious and Nonserious Adverse Events

All AEs, serious and nonserious and regardless of causality to study drug (including the exacerbation of a pre-existing condition), will be fully recorded on the appropriate eCRF. For each AE, the Investigator must provide its duration (start and end dates or ongoing), severity (intensity), assessment of causality and whether specific action or therapy was required and whether action was taken with regard to study drug treatment.

Any AE that occurs from the signing of the ICF until the first dose of study drug should be recorded on the AE eCRF page only if the event was related to a study procedure; all other

AEs/findings prior to the first dose of study drug should be recorded as medical history on the applicable eCRF page(s). All AEs occurring from the first dose of study drug until 30 days after the last dose of study drug must be recorded on the AE eCRF.

All SAEs must additionally be reported to the Sponsor within 24 hours of the Investigator's knowledge by faxing the completed SAE form to the Sponsor at the number provided on the SAE form or fax cover sheet.

After the 30-day Safety Follow-up, only new SAEs that are considered related to study drug will be captured on the eCRF and reported to the Sponsor or designee using the SAE form.

Investigators must follow patients with AEs/SAEs until the event has resolved, the condition has stabilized, withdrawal of consent, initiation of subsequent anticancer therapy, the patient is lost to follow-up or death OR until the 30-day Safety Follow-up, whichever occurs first. Ongoing treatment-related AEs/SAEs may be followed beyond the 30-day Safety Follow-up if clinically indicated.

If a patient is lost to follow-up, this should be captured accordingly within the eCRF and on a follow-up SAE report.

10.10 Reporting of Suspected Unexpected Serious Adverse Events (SUSAR)

Suspected Unexpected Serious Adverse Events (SUSAR) will be collected and reported by the Sponsor and/or designee to the competent authorities and relevant ethics committees in accordance with Directive 2001/20/EC or as per national regulatory requirements in participating countries.

10.11 Pregnancy or Drug Exposure during Pregnancy

If a patient becomes pregnant during the study, administration of study drug is to be discontinued immediately.

Pregnancies (both those of female patients and female partners of male patients) must be reported to the Sponsor or designee within 24 hours of the Investigator's knowledge using the Clinical Pregnancy Report. All pregnancies will be followed through to outcome and the outcome must be reported to the Sponsor or designee using the Clinical Pregnancy Outcome Report.

Pregnancies themselves are not considered AEs or SAEs. However, any AEs or SAEs occurring during pregnancy are to be reported following AE and SAE reporting guidelines.

10.12 Review of Safety Data

The Medical Monitor or designee, and the Sponsor's Drug Safety Department will be responsible for the ongoing review and evaluation of safety data, including AEs, laboratory data, and any other safety evaluations, throughout the duration of the study.

Additionally, an independent DMC with a defined charter will be used to determine if the study should be stopped or suspended at any time to allow additional review of safety data.

11.0 STATISTICAL METHODS

The study contains a Safety Lead-in and a Japan Safety Lead-in Phase in which the safety and tolerability of encorafenib + binimetinib + cetuximab will be assessed. Once the safety profile has been assessed by the DMC, subsequent patients will be randomized to the Phase 3 portion of the study. The primary endpoints of the study are OS of Triplet Arm vs Control Arm and ORR (per BICR) of Triplet Arm vs Control Arm.

An initial analysis conducted for the study will serve as the primary analysis for the ORR primary endpoint based on the first 330 patients randomized. At the same time, an interim analysis for superiority or (non-binding) futility of the OS primary endpoint will be performed. If the OS interim analysis results do not cross the superiority boundary, the OS primary endpoint will be analyzed again at the final analysis. If the interim analysis for OS of Triplet vs. Control exceeds the superiority boundary, patients in the Triplet and Doublet arms may continue to be followed for a more mature comparison.

11.1 Determination of Sample Size

The planned sample size is approximately 646-651 patients (includes 31-36 patients for the non-Japanese and Japanese Safety Lead-in study portions and approximately 615 in the randomized Phase 3 portion of the study). Additional patients may be included in Safety Lead-in if lower dose levels are evaluated.

11.1.1 Safety Lead-in

During the Safety Lead-In, the first 9 evaluable patients will be enrolled to the starting dose of the Triplet Arm. This Triplet dose will be presented to the DMC as tolerable if the observed Cycle 1 DLT rate is < 33% (i.e., < 3 patients with DLTs out of 9 patients). Table 29 provides a comparison of the operating characteristics for this dose-escalation rule with 9 patients and traditional 3+3 rules. The results illustrate the benefit of the additional patients as the probability of falsely declaring a dose to be toxic is lower with a 9-patient cohort when the true DLT rate is ≤ 20%. Similarly the probability of correctly declaring a dose to be toxic is higher with the 9-patient cohort when the true DLT rate is ≥40%. In addition, observing no Cycle 1 DLTs in 9 patients would be expected to occur with probability 0.040 if the true DLT rate is 30%.

Table 29: Operating Characteristics of Safety Lead-In Criteria for 9 Patients Compared to 3+3 Rules

True Cycle 1 DLT Rate	Probability of dose declared toxic using 3+3 rules	Probability of observed Cycle 1 DLT rate ≥ 33% in 9 patients
10%	0.094	0.053
20%	0.291	0.262
30%	0.506	0.537
40%	0.691	0.768
50%	0.828	0.910

If the DMC determines the doses to be tolerable in the first 9 evaluable patients based on observing DLTs in < 33% of patients and evaluation of the overall toxicity profile, the Safety Lead-in will be expanded by an additional 16-21 patients. A total of 25-30 patients will complete the Safety Lead-in (the initial 9 plus the additional patients in the expansion) at the doses proposed for the randomized Phase 3 portion of the study. If dose de-escalation is required during the Safety Lead-in, additional patients may be necessary.

A separate safety evaluation of Japanese patients will be conducted once the Japanese Safety Lead-In cohort completes 1 cycle of therapy. For this cohort, 6 patients will be enrolled, resulting in a total of 31-36 patients for the entire Safety Lead-In study portion.

11.1.2 Randomized Phase 3

Overall Survival

During the randomized Phase 3 portion of the study, eligible patients will be randomized 1:1:1 such that approximately 205 patients will receive binimetinib + encorafenib + cetuximab (Triplet Arm), approximately 205 patients will receive encorafenib + cetuximab (Doublet Arm) and approximately 205 patients will receive irinotecan/cetuximab or FOLFIRI/cetuximab (Control Arm). The number of 3rd-line patients (those having received 2 prior regimens) randomized to the Phase 3 portion of the study will be limited to 215 (35% of the total randomized) in order to ensure generalizability of the study. Patients with 2 prior regimens who have entered Screening at the time that the limit has been reached will be permitted to continue into the study if they are otherwise determined to be eligible. Permitting up to 35% of the randomized patients to have received 2 prior regimens ensures a sufficient sample size to adequately generalize the results across subgroups when incorporating other relevant stratification factors. Randomization will be stratified by ECOG PS (0 vs. 1), prior use of irinotecan (yes vs. no), and cetuximab source (US-licensed vs. EU-approved).

The primary endpoints are OS and ORR (per BICR) of the Triplet Arm vs. Control Arm. Historical evidence (Peeters et al. 2014) suggests that the median OS of FOLFIRI combined with

another EGFR inhibitor (panitumumab) will be approximately 5 months in *BRAF*-mutant mCRC. Existing published data suggest only modest differences between irinotecan single-agent and FOLFIRI efficacy (Clarke et al. 2011). In addition, available data suggests that treatment-eligible patients in the 2nd and 3rd-line settings and with or without prior irinotecan have similar PFS and OS with standard therapy approaches (DeRoock et al. 2010, Morris et al. 2014, Peeters et al. 2014). Based on these findings, it is assumed that both control arm options will have an approximate median OS of 5 months.

The number of patients required for the randomized Phase 3 portion of the study is driven by the key secondary endpoint of the OS of the Doublet Arm vs. Control Arm. For this comparison, the study is powered to detect an improvement of 2.1 months (7.1 months vs. 5 months; HR=0.70). With 338 OS events, the study will have approximately 90% power to detect this improvement using a group-sequential design and 1-sided α=0.025. Assuming accrual to the randomized Phase 3 portion of the study increases over a period of time before reaching a maximum of 25 patients per month (for an accrual duration of approximately 25 months) and 5% loss to follow-up, approximately 615 patients will be randomized to reach 338 events. With 338 events, the null hypothesis that the OS in the Doublet Arm is the same or worse than the Control Arm will be rejected if the hazard ration (HR) is smaller (i.e., better) than 0.81.

The final analysis for OS will occur once at least 268 events are observed in the Triplet Arm + Control Arm and at least 338 events are observed in the Doublet Arm + Control Arm. This is expected to occur approximately 33 months after the first patient is randomized (i.e., approximately 8 months after randomization is complete). At the time of the final analysis, it is anticipated that approximately 333 OS events will be observed in the Triplet Arm + Control Arm, but only the first 268 OS events will be included in the final analysis. With 268 events, there will be approximately 90% power to detect a HR=0.67 (median OS of 7.5 months vs. 5 months) with 1-sided alpha = 0.025. The null hypothesis that the OS in the Triplet Arm is the same or worse than the Control Arm will be rejected if the HR is smaller (i.e., better) than 0.79.

Overall Response

During the initial analysis of the study, the primary analysis of Triplet vs. Control ORR by BICR will be conducted based on the first 330 randomized patients. Based on historical data, the ORR in the control arm is expected to be approximately 10% (Kopetz et al. 2017, Seymour et al. 2013). A sample size of 110 patients per arm provides 88% power to detect a 20% absolute difference in ORR, assuming an ORR of 10% in the Control Arm and an ORR of 30% in the Triplet Arm at a 1-sided alpha of 0.005.

11.2 Analysis Sets

11.2.1 Full Analysis Set

For patients in the Safety Lead-in, the Full Analysis Set (FAS) will consist of all patients who receive at least 1 dose of study drug and have at least 1 post-treatment assessment, which may include death.

For patients in the Phase 3 portion, the FAS will consist of all randomized Phase 3 patients. Patients will be analyzed according to the treatment arm and stratum they were assigned to at randomization.

11.2.2 Safety Set

The Safety Set (SS) will consist of all patients who receive at least 1 dose of study drug and have at least 1 post-treatment assessment, which may include death. Patients will be analyzed according to treatment received.

11.2.3 Dose-Determining Set

The dose-determining set (DDS) includes all Safety Lead-In patients from the SS who either completed a minimum exposure requirement and have sufficient safety evaluations or experienced a DLT.

A patient is considered to have met the minimum exposure requirement if having received at least 75% dose intensity ([Cumulative administered dose in mg in Cycle 1/Cumulative planned dose in mg in Cycle 1] x 100) of the planned dose for each of the 3 agents; binimetinib (i.e., 1890 of the planned 2520 mg dose), encorafenib (i.e., 6300 of the planned 8400 mg dose) or cetuximab doses (i.e., 750 of the planned 1000 mg/m^2 dose) within the first Cycle of dosing. The length of a cycle is 28 days.

Patients who do not experience DLT during the first cycle will be considered to have sufficient safety evaluations if they have been observed for ≥ 28 days following the first dose, and are considered by both the Sponsor and Investigators to have enough safety data to conclude that a DLT did not occur.

11.2.4 Safety Lead-in Efficacy Set

The Safety Lead-in Efficacy Set will consist of all Safety Lead-in patients in the FAS who were identified at screening as having a *BRAF*^{V600E} mutation (per local or central testing).

11.2.5 Phase 3 Response Efficacy Set

The Phase 3 Response Efficacy Set will consist of the first 330 patients randomized into the Phase 3 portion of the study.

11.2.6 Per Protocol Set

The Per-protocol Set (PPS) will consist of all Phase 3 patients in the FAS who are sufficiently compliant with the protocol requirements. A precise definition of the criteria required for inclusion in the PPS will be provided in the statistical analysis plan (SAP).

11.2.7 Pharmacokinetic Set

The PK set will consist of all patients who receive at least 1 dose of encorafenib, binimetinib or cetuximab, and who have at least 1 post-dose PK blood collection with associated bioanalytical results.

11.3 Statistical Analyses and Methods

11.3.1 General Considerations

A detailed SAP will be prepared by the Sponsor. This plan may modify the statistical methods outlined in the protocol; however, any major modifications of the primary endpoints or key secondary endpoint definition or analysis will also be described in a protocol amendment.

Data analyses from this study will be performed by the Sponsor or designee.

The primary clinical study report (CSR) will be based on the data generated prior to the data cutoff date (see Section 11.3.9). Addendums to the CSR will be written once the end of the study has occurred. Additional data cutoff dates for other analyses may be established if requested per Health Authorities.

11.3.2 Patient Characteristics

The following baseline patient characteristics will be summarized descriptively by treatment arm: stratification factors (ECOG PS, prior use of irinotecan, cetuximab source); demographics (age, gender and race); histology; prior anticancer agents (chemotherapy, biologics, targeted small molecules); best response to prior irinotecan-based therapy; best response to prior chemotherapy; prior radiotherapy, prior surgery; primary site of disease; medical history; concomitant medication usage; various genes’ mutation status.

11.3.3 Efficacy Analyses

The primary efficacy endpoints are OS (Triplet Arm vs. Control Arm) and ORR by BICR (Triplet Arm vs. Control Arm) (see Sections 11.3.3.1 and 11.3.3.2).

The key secondary efficacy endpoint is OS (Doublet Arm vs. Control Arm) and other secondary endpoints include ORR (Triplet Arm vs. Control Arm and Doublet Arm vs. Control Arm), PFS (Triplet Arm vs. Control Arm and Doublet Arm vs. Control Arm), OS (Triplet Arm vs. Doublet Arm), ORR (Triplet Arm vs. Doublet Arm), DOR (Triplet Arm vs. Control Arm, Doublet Arm

vs. Control Arm, Triplet Arm vs. Doublet Arm), and time to response (Triplet Arm vs. Control Arm, Doublet Arm vs. Control Arm, Triplet Arm vs. Doublet Arm).

Unless otherwise stated, efficacy analyses will be conducted using the FAS. A supportive analysis of the primary endpoints will be conducted using the PPS. For endpoints related to tumor evaluations (PFS, ORR, DOR, time to response), the primary analysis will be based on the assessments per BICR. The secondary endpoints based on Investigator assessment will each be summarized as a supportive analysis.

The analyses described below for ORR, DOR, and PFS secondary endpoints may also be conducted using descriptive statistics if the primary endpoint of ORR is statistically significant at the primary analysis. The analyses for OS described below will be conducted only when the primary endpoint of OS exceeds the superiority boundary or the study stops for futility.

The Type I error rate for the primary endpoints will be controlled using a fallback procedure described by Wiens and Dmitrienko (2005). One-sided alpha of 0.005 will be assigned to the Triplet vs Control ORR endpoint. The remaining 0.020 will be assigned to the Triplet vs Control OS endpoint. If the p-value of the Triplet vs Control comparison of ORR at the primary analysis is <0.005, then the Triplet vs Control OS comparison will be assigned a total 1-sided alpha of 0.025. Otherwise, it will remain at 1-sided 0.020.

The key secondary endpoint and several secondary endpoints may also be formally tested. To control the overall Type I error rate, a gatekeeping procedure using hierarchical testing will be performed. If the OS of the Triplet Arm vs. Control Arm is found to be significant at either the OS interim analysis (Section 11.3.9) or the final analysis, the following tests will then be formally conducted sequentially, each at the same total alpha assigned to the Triplet vs Control OS endpoint:

1. OS of Doublet Arm vs. Control Arm
2. ORR (per BICR) of Doublet Arm vs. Control Arm
3. PFS (per BICR) of Triplet Arm vs. Control Arm
4. PFS (per BICR) of Doublet Arm vs. Control Arm

If any of the above tests is found to not be statistically significant, all subsequent comparisons will only be summarized using descriptive statistics.

The overall testing strategy of the study is summarized in Figure 9.

Figure 9: Testing Strategy for Study ARRAY-818-302

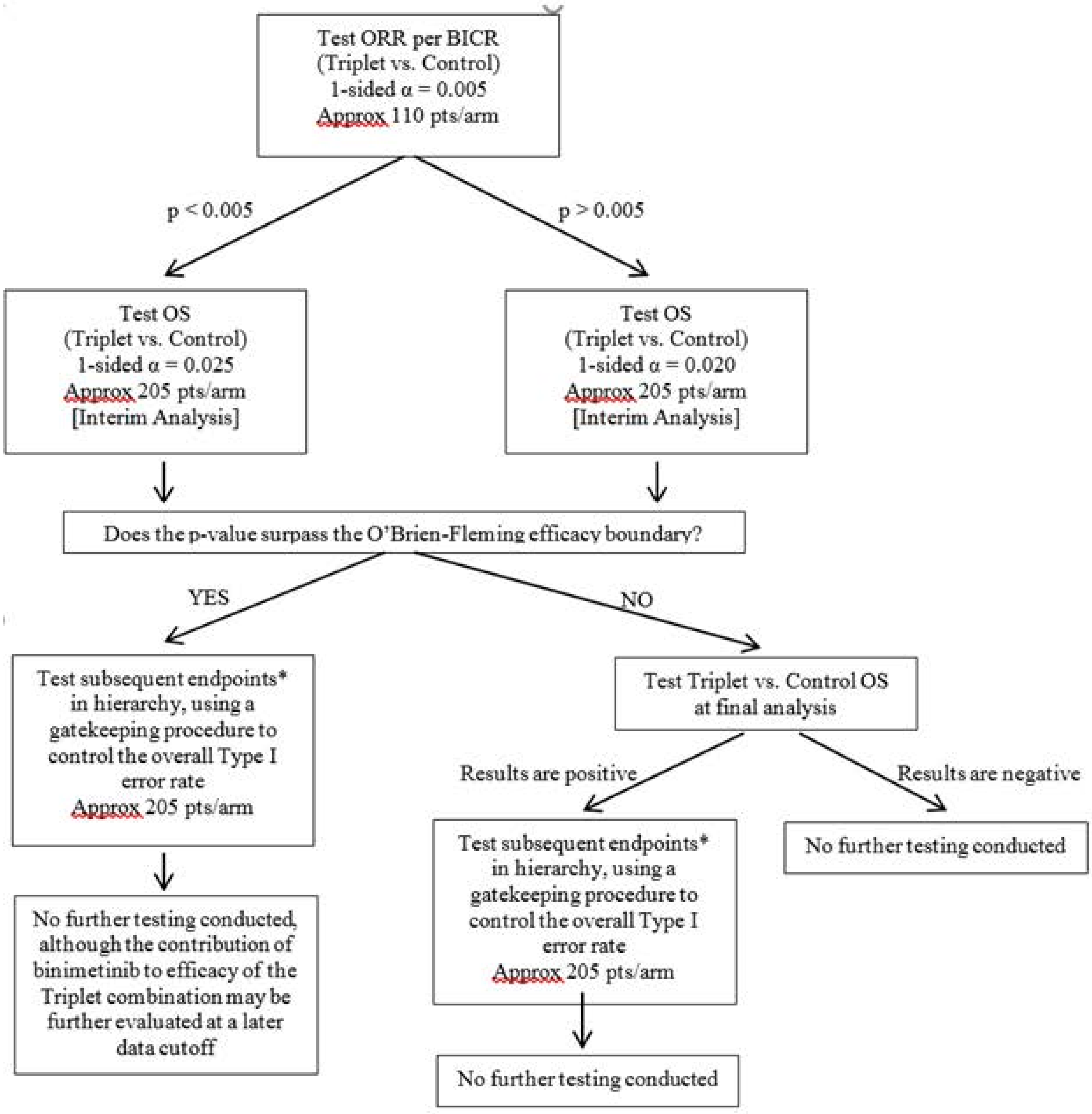

* Subsequent endpoints would be tested in the following order: Doublet vs. Control OS, Doublet vs. Control ORR per BICR, Triplet vs. Control PFS per BICR, and then Doublet vs. Control PFS per BICR.

11.3.3.1 Overall Survival

Overall survival for Phase 3 is defined as the time from randomization to death due to any cause. Patients who do not die by the data cutoff date will be censored for OS at their last contact date. Overall survival will be calculated for all patients and summarized by treatment arm using the Kaplan-Meier (KM) method. Overall treatment arm estimates as well as treatment arm estimates by stratum will be provided.

For the OS primary endpoint, the null hypothesis is that the OS for the Triplet Arm is less than or equal to the OS of the Control Arm, i.e.,

$$H_0\text{: } S_{OS,A}\ (t) \leq S_{OS,C}\ (t)$$

where $S_{OS,A}$ (t) is the OS survival distribution function for the Triplet Arm and $S_{OS,C}$ (t) is the OS survival distribution function for the Control Arm. The null hypothesis will be tested using a stratified log-rank test against the alpha assigned to the endpoint based on the fallback approach. The stratification factors used in the test will be those used for randomization, and will be based on the actual randomization (IWRS) information. A stratified Cox proportional hazard model will be fit with treatment arm as the covariate to obtain a HR estimate of the treatment effect along with 95% CI

The primary analysis of OS will be based on the intent-to-treat (ITT) principle, with patients in the treatment group assigned at randomization. Additional details will be provided in the SAP.

A similar hypothesis and testing approach will be used for the comparison of the Doublet Arm OS to the Control Arm and for the secondary objective of comparing the Triplet Arm OS to that of the Doublet Arm.

At the initial analysis of the study, an interim analysis for superiority or (non-binding) futility of the Triplet vs Control OS endpoint will be performed based on all available data (i.e., using the FAS). Futility and superiority boundaries for both the OS interim and final analyses will be determined using a Lan-DeMets spending function (Lan and DeMets 1983) that approximates O’Brien-Fleming stopping boundaries.

11.3.3.2 Objective Response Rate

The overall best response (i.e., CR or PR) as assessed by BICR per RECIST v1.1 will be determined for each patient. The ORR will be calculated within each treatment arm, where ORR is defined as the number of patients achieving an overall best response of CR or PR divided by the total number of patients in that treatment arm.

The ORR will be tested for the primary endpoint Triplet Arm vs. Control Arm comparison using the Cochran-Maentel-Haenszel test at a one-sided alpha of 0.005 using the Phase 3 Response Efficacy Set. Both confirmed and unconfirmed ORR will be summarized but, for purposes of

formal testing, the analysis of the confirmed responses will be used. The stratification factors used in the test will be those used for randomization and will be based on the actual randomization (IWRS) information. For the primary analysis, ORR will be presented by arm and stratum, along with a 95% and 99% CI.

11.3.3.3 Efficacy Analyses of Secondary Endpoints

11.3.3.3.1 Progression Free Survival (PFS)

Progression-free survival is defined as the time from randomization to the earliest documented date of disease progression, per RECIST v1.1 and as determined by Investigator, or death due to any cause. Progression-free survival will be calculated for all patients and summarized by treatment arm using the KM method. The Safety Lead-in Efficacy Set will be used to summarize the PFS in Safety Lead-in patients, and the FAS will be used for the randomized Phase 3 patients.

Progressive disease and death (from any cause) will be considered as events. If death or PD is not observed, the PFS will be censored at the date of last adequate tumor assessment (i.e., at the date of last tumor assessment of CR, PR or SD) prior to cutoff date or date a subsequent anticancer therapy for mCRC is started (e.g., systemic therapy, non-palliative radiotherapy). However, if a PFS event is observed after more than 1 missing or inadequate tumor assessment, PFS will be censored at the last adequate tumor assessment. If a PFS event is observed after a single missing or non-adequate tumor assessment, the actual date of event will be used.

When a patient discontinues treatment for “disease progression” but without documentation of radiological and/or pathological evidence of progression based on RECIST it will not be counted as a PFS event. The patient will continue to be followed for PFS until disease progression, withdrawal of consent, initiation of subsequent anticancer therapy, the patient is lost to follow-up or until death. If a PFS event occurs during this follow-up, the date of the event will be included in the analysis. If the patient does not have a PFS event during this time, the patient will be censored at the last assessment date.

Progression-free survival of the arms will be compared using a stratified log-rank test. The stratification factors used in the test will be those used for randomization, and will be based on the actual randomization (IWRS) information. A stratified Cox model will be fit with treatment arm as the covariate to obtain an HR estimate of the treatment effect along with 95% CI.

Additional sensitivity analyses examining alternative censoring rules will be described in the SAP.

11.3.3.3.2 Objective Response Rate

The overall best response (i.e., CR or PR) as assessed by the Investigator and retrospectively by BICR per RECIST v1.1 will be determined for each patient. The ORR will be calculated within

each treatment arm, where ORR is defined as the number of patients achieving an overall best response of CR or PR divided by the total number of patients in that treatment arm. The Safety Lead-in Efficacy Set will be used to summarize the ORR in Safety Lead-in patients, and both the Phase 3 Response Efficacy Set and FAS will be used for the randomized Phase 3 patients.

The ORR will be compared between treatment arms using the Cochran-Maentel-Haenszel test. Both confirmed and unconfirmed ORR will be summarized, but for purposes of the hierarchical testing, the analysis of the confirmed responses will be used. The stratification factors used in the test will be those used for randomization and will be based on the actual randomization (IWRS) information. The ORR along with a 95% CI will be presented by arm.

11.3.3.3.3 Duration of Response

Duration of response is defined as the time from first radiographic evidence of response to the earliest documented PD or death and is calculated for responders only. Responders who do not have a PD or death date by the data cutoff date will be censored for DOR at their last adequate radiological assessment (i.e., at the date of last tumor assessment of CR, PR or SD) prior to cutoff date or date a subsequent anticancer therapy for mCRC is started. The Safety Lead-in Efficacy Set will be used to summarize the DOR in Safety Lead-in patients, and both the Phase 3 Response Efficacy Set and FAS will be used for the randomized Phase 3 patients. Duration of response will be summarized by arm using the KM method.

11.3.3.3.4 Time to Response

Time to response (CR or PR) is defined as the time from date of randomization to date of first radiographic evidence of response. Patients who do not have a CR or PR by the data cutoff date will be censored for time to response at their last radiological assessment. Patients who receive subsequent anticancer therapy prior to response will be censored at their last radiological assessment prior to initiation of subsequent anticancer therapy. The Safety Lead-in Efficacy Set will be used to summarize the time to response in Safety Lead-in patients, and both the Phase 3 Response Efficacy Set and FAS will be used for the randomized Phase 3 patients. Time to response will be summarized by arm using the KM method.

11.3.3.4 Assessment of the Binimetinib Contribution to Efficacy

To adequately assess the efficacy benefit that binimetinib contributes to the triplet combination, comparisons of the Triplet Arm to the Doublet Arm will be conducted for each efficacy endpoint using the FAS.

A comparison of the OS distributions for the Triplet Arm and the Doublet Arm will be described in tabular and graphical format by treatment arm using KM methods. A Cox regression model stratified by randomization stratification factors will be also used to estimate the HR of OS, along with 95% CI based on the Wald test. Nominal p-values will be provided.

In addition to the response rates reported by treatment arms with exact binomial confidence intervals, the Cochran-Mantel-Haenszel test will be used to compare ORR between the Triplet Arm and the Doublet Arm. The stratification factors used in the test will be those used for randomization, and will be based on the actual randomization (IWRS) information. Nominal p-values will be provided. Separate summaries for confirmed responses and all responses (confirmed and unconfirmed) will be generated.

The PFS of the Triplet Arm and the Doublet Arm will be summarized using the KM method. Nominal p-values will be provided. Time to response and DOR of the two arms will also be summarized using the KM method.

11.3.4 Pharmacokinetic Analysis

Plasma concentrations of encorafenib, binimetinib and the active metabolite of binimetinib (AR00426032) and serum concentrations of cetuximab will be determined using validated assays. Descriptive statistics of concentrations will be reported and summarized graphically.

Pharmacokinetic parameters from the Safety Lead-in will be generated by noncompartmental and/or compartmental approaches as appropriate. No noncompartmental PK parameters will be estimated in the Phase 3 portion of the study due to sparse sampling. Phase 3 PK parameters will be estimated for encorafenib, cetuximab, binimetinib and the active metabolite of binimetinib (AR00426032) as appropriate using a population model-based approach for patients in the Safety Lead-in, Triplet, Doublet, and Control Arms, as appropriate, to determine appropriate model-based PK parameters and their respective variability, if deemed appropriate. Pharmacokinetic data may be analyzed in at least 2 ways:

- Data from this study will be analyzed alone based on the previously described population PK model. The structure of the model will remain similar but parameters (fixed and random effects) will be estimated based on the current population.
- If needed, prior knowledge from other studies with intensive sampling may be introduced in the analysis by either fixing the population parameters to the value estimated previously or by adding data from the previous studies to the dataset to estimate population parameters and derive Empirical Bayes Estimates of PK parameters for patients in this study.

Assessments for potential drug interactions between encorafenib, cetuximab, binimetinib and the active metabolite (AR00426032) will be conducted by comparison to historical results for all 3 study drugs and by covariate modeling as part of the population PK analysis, as appropriate. The following effects will be assessed: encorafenib exposure affecting cetuximab exposure, binimetinib exposure affecting cetuximab exposure and encorafenib exposure affecting binimetinib exposure. The effect of clinical source of cetuximab study drug on cetuximab PK may also be assessed. Additional assessments may be performed as appropriate.

Details of these analyses and of the incorporation of prior information will be provided in a specific standalone modeling plan. Analyses will be provided in a separate report.

Relationships between PK and biomarkers, clinical response and/or safety will be explored if appropriate and data quality is adequate. Details of analyses will be included in statistical analysis plans and results will be reported separately.

11.3.5 Exploratory Analyses

In the Safety Lead-in portion of the study, OS will be analyzed as an exploratory endpoint and is defined as the time from first dose of study drug to death due to any cause. Patients who are alive as of the data cutoff date will be censored for OS at their last contact date. Overall survival will be calculated for all Safety Lead-in patients and summarized using the KM method.

In the Phase 3 portion of the study, the relationship between protein levels, mutations and/or gene expression and clinical outcomes may be explored.

Details of all exploratory analyses will be provided in the SAP.

11.3.6 Subgroup Analyses

Selected safety analyses may be performed on various subgroups such as age (< 65 vs. ≥ 65 years old), gender, race (white vs. nonwhite), CRP levels (>ULN and <ULN), MSI status (high and low), location of primary disease, removal of primary tumor and possibly others. The effects of the primary endpoint of OS will be displayed using a forest plot of the treatment effect HRs by subgroups, including stratum. Further details including the precise subgroups used will be detailed in the SAP.

11.3.7 Safety Analyses

Safety analyses will be conducted on the SS.

Safety data will be presented in tabular and/or graphical format and summarized descriptively by treatment arm and study day, where appropriate. Listings of all safety data sorted by treatment arm, patient and assessment date will be provided. Key safety analyses will also be conducted at the time of the end of study.

11.3.7.1 Study Drug Exposure

Duration of study treatment exposure, actual and relative dose intensity will be summarized by study drug and treatment arm using the SS. The number of patients with dose modifications/interruptions will be presented by study drug and treatment arm, along with reasons for the dose modification. The actual daily doses and reasons for dose modification will be listed.

11.3.7.2 Concomitant Medications

Concomitant medications will be listed and summarized by Anatomical Therapeutic Classification System term, preferred term and treatment arm. These summaries will include medications starting on or after the start of study treatment or medications starting prior to the start of study treatment and continuing after the start of study treatment.

11.3.7.3 Adverse Events

Adverse events will be coded using the Medical Dictionary for Regulatory Activities (MedDRA). Incidence tables will be presented for all AEs by maximum grade. Adverse events, SAEs, AEs assessed as related to study drug and AEs resulting in dose reduction, interruption or discontinuation of study drug will be tabulated, as well as AEs leading to additional therapy.

Summaries for deaths on-study will be provided by system organ class, preferred term, and treatment arm.

Adverse events of special interest for both encorafenib and binimetinib will be identified and additional analyses, including time to onset and duration, may be estimated for these events. Such categories consist of 1 or more well-defined safety events which are similar in nature and for which there is a specific clinical interest in connection with the study treatment. For each specific category, number and percentage of patients with at least 1 event will be reported.

For this study, the categories for encorafenib include the following (based on grouping of selected AEs and laboratory parameters) and may be updated during the course of the study based on accumulating safety data:

- Acute renal failure
- Dermatologic events (rash and skin except rash)
- Facial paresis
- Fatigue/asthenia
- Gastrointestinal events
- Headache
- Heart rate increased events
- Myopathy/rhabdomyolysis events
- Ophthalmic events (retinal, vascular and other eye events)

- QTc prolongation
- Secondary neoplasms

The categories for binimetinib include the following (based on grouping of selected AEs and laboratory parameters) and may be updated during the course of the study based on accumulating safety data:

- Cardiac events (i.e., LVEF decrease)
- Dermatologic events (rash and skin except rash)
- Edema events
- Fatigue and asthenia
- Gastrointestinal events
- Hepatic events
- Hemorrhage
- Hypertension
- Ophthalmic events (retinal, vascular and other eye events)
- Myopathy/rhabdomyolysis events
- Pneumonitis
- QTc prolongation
- Thrombotic/embolic events

Adverse events of special interest do not require special reporting to the Sponsor unless the event(s) meets SAE reporting criteria (see Section 10.9).

11.3.7.4 Laboratory Parameters

Hematology and chemistry laboratory parameters will be presented in shift tables of Baseline grade vs. maximum grade on study. For laboratory parameters that are not gradable by CTCAE, shift tables of normal-abnormal will be provided. Laboratory measurements will be summarized descriptively by visit and treatment arm, and plots of measurements over time will be generated for selected parameters.

Both central and local laboratory values will be considered for summaries and both will be presented in listings.

11.3.7.5 Electrocardiograms

Electrocardiogram values will be summarized descriptively and/or with shift tables by treatment arm.

11.3.7.6 Other Safety Data

Vital signs, body weight, ECOG PS, dermatologic and ophthalmic examination data will be summarized descriptively by visit and/or with shift tables by treatment arm. Summaries of clinically notable measurements will also be provided. Definitions of "clinically notable" will be provided in the SAP.

11.3.7.7 Patient-Reported Outcomes

Quality-of-life data will be analyzed on the FAS.

The EORTC QLQ-C30, FACT-C, EQ-5D-5L and PGIC will be used to assess QoL. For EORTC-QLQ-C30, the global health status/QoL scale score is identified as the primary patient-reported outcome variable of interest; physical functioning, emotional functioning and social functioning scale scores of the QLQ-C30 are considered as secondary. For FACT-C, the functional well-being score is the primary patient-reported outcome variable of interest; the physical well-being, social/family well-being, emotional well-being, and additional concern scores are considered as secondary. The EQ-5D-5L contains 1 item for each of 5 dimensions health-related quality of life (i.e., mobility, self-care, usual activities, pain or discomfort, and anxiety or depression). Response options for each item vary from having no problems, moderate problems, or extreme problems.

The EORTC QLQ-C30, FACT-C, EQ-5D-5L and PGIC will be scored according to respective scoring manuals, respectively. The number of patients completing the questionnaires and the number of missing or incomplete assessments will be summarized by each treatment arm for each scheduled assessment time point.

Descriptive statistics will be used to summarize the scored scales at each scheduled assessment. Additionally, change from baseline in the domain scores at the time of each assessment will be summarized. Patients with an evaluable baseline score and at least 1 evaluable post-baseline score during the treatment period will be included in the change from baseline analyses. In addition, a repeated measurement analysis model may be used to compare the 2 treatment arms with respect to changes in the domain scores longitudinally over time. Full details of the modeling analysis will be provided in the SAP.

Time to definitive deterioration in the QoL domains will be assessed in the treatment arms in the FAS. The time to definitive deterioration is defined as the time from the date of randomization to the date of event, which is defined as at least 10% worsening relative to Baseline of the corresponding scale score with no later improvement above this threshold observed during the course of the study or death due to any cause. If a patient has not had an event prior to analysis cutoff or start of another anticancer therapy, time to deterioration will be censored at the date of the last adequate QoL evaluation. The distribution will be presented descriptively using KM curves. Median time to definitive deterioration along with 2-sided 95% CI will be provided. Additionally, time to definitive deterioration with different cutoff definitions (e.g., 5%, 15%) may be specified in the SAP as deemed appropriate. A Cox model will be fit with treatment arm and stratification factors as the covariates to obtain a HR estimate of the treatment effect along with 95% CI. The stratification factors used in the test will be precisely those used for randomization, and will be based on the actual randomization (IWRS) information.

11.3.8 Interim Safety Reviews

The independent DMC will review the available safety information after the first 30 patients in the randomized Phase 3 portion of the study (i.e., approximately 10 patients in each arm) have had the opportunity to complete at least 1 cycle of treatment to confirm tolerability. Subsequent DMC safety data reviews will occur at regular intervals. Additional details will be outlined in the DMC Charter.

11.3.9 Initial Efficacy Analyses

An initial analysis of the study will be performed when all three of the following criteria have been met:

- approximately 9 months after randomization of the 330th patient (i.e., approximately 110 patients per arm), to allow a majority of responders among the 330 Phase 3 patients to have the opportunity to be followed for approximately 6 months or longer after their first response
- at least 188 OS events have occurred in the Triplet and Control arms combined (i.e., approximately 70% information)
- at least 169 OS events have occurred in the Doublet and Control arms combined (i.e., approximately 50% information)

The primary endpoint of Triplet vs. Control ORR (per BICR) will be formally tested first at this analysis and will be based on the first approximately 330 randomized patients (any additional patient[s] randomized on the same day as the 330th randomized patient will be included in the analysis). If the Triplet vs. Control ORR comparison is positive (i.e., $p < 0.005$), then based on the fallback procedure, the OS endpoint of Triplet vs. Control will be assigned a total 1-sided alpha = 0.025. Otherwise, the Triplet vs. Control OS will be assigned a total 1-sided alpha = 0.020.

An interim analysis for superiority or (non-binding) futility of the Triplet vs Control OS endpoint will be also performed at the time of the primary ORR analysis based on all available data. Futility and superiority boundaries for both the OS interim and final analyses will be determined using a Lan-DeMets spending function that approximates O'Brien-Fleming stopping boundaries.

If the p-value for the Triplet Arm vs Control Arm OS comparison exceeds the superiority boundary at the interim analysis, testing of the endpoints included in the hierarchical approach described in Section 11.3.3 will be conducted. For each endpoint in the hierarchy, a Lan-DeMets spending function that approximates the O'Brien-Fleming stopping boundaries will be used with data from all available patients. The total alpha assigned to each endpoint will be either 0.025 (if the Triplet Arm vs. Control Arm ORR comparison was $p < 0.005$) or 0.020 (if the Triplet Arm vs. Control Arm ORR comparison was $p \geq 0.005$).

If the p-value for the Triplet Arm vs Control Arm OS comparison does not exceed the superiority boundary at the interim analysis, OS of Triplet Arm vs. Control Arm will be tested again at the final analysis (i.e., when at least 268 OS events in Triplet and Control and at least 338 OS events in Doublet and Control have occurred). Table 30 provides the number of expected events, the cumulative alpha spent, and the cumulative power to reject the null hypothesis at the expected analysis timepoints for the Triplet vs Control OS analysis.

Table 30: Expected Number of OS Events and Cumulative Power at Expected OS Analysis Timepoints for the Triplet vs Control Comparison

	OS Analysis			
	**Triplet vs Control OS Analysis**	**Cumulative Number of OS Events**	**Cumulative Alpha Spent on OS**	**Cumulative Power to Reject H0 for OS (%)**
Triplet vs Control ORR is statistically significant (i.e., one-sided p<0.005)	Interim	188	0.0074	61.5
	Final	268	0.0250	88.3
Triplet vs Control ORR is not statistically significant (i.e., one-sided p>0.005)	Interim	188	0.0055	57.4
	Final	268	0.0200	86.3

Note: All values were calculated using East® v6.4, assuming 70% information at the OS interim analysis. Boundaries will be adjusted according to the actual information fraction observed at the interim analysis. Cumulative power values were estimated using simulations within East® under the alternative hypothesis.

Efficacy and futility are determined using Lan-DeMets approximation of O'Brien-Fleming alpha- and (non-binding) beta-spending boundaries, respectively.

12.0 DATA RECORDING, RETENTION AND MONITORING

12.1 Data Management

Data will be collected using an electronic data capture system (EDC) at the clinical site. The Investigator or designee will record data specified in the protocol using eCRFs. Changes or corrections to eCRFs will be made by the Investigator or an authorized member of the study staff according to the policies and procedures at the site.

It is the Investigator's responsibility to ensure eCRFs are complete and accurate. Following review and approval, the Investigator will electronically sign and date the pages. This signature certifies that the Investigator has thoroughly reviewed and confirmed all data on the eCRF.

A portable document format (PDF) file of the eCRFs will be provided to the site after all data have been monitored and reconciled. An electronic copy will be archived at the site.

12.2 Data Monitoring

This study will be closely monitored by representatives of the Sponsor throughout its duration. Monitoring will include personal visits with the Investigator and study staff as well as appropriate communications by telephone, fax, mail, email or use of the EDC system, if applicable. It is the monitor's responsibility to inspect eCRFs at regular intervals throughout the study to verify the completeness, accuracy and consistency of the data and to confirm adherence to the study protocol and to Good Clinical Practice (GCP) guidelines. The Investigator agrees to cooperate with the monitor to ensure that any problems detected during the course of this study are resolved promptly. The Investigator and site will permit study-related monitoring, audits, EC review and regulatory inspection, including direct access to source documents.

It is understood that study monitors and any other personnel authorized by the Sponsor may contact and visit the Investigator and will be permitted to inspect all study records (including eCRFs and other pertinent data) on request, provided that patient confidentiality is maintained and that the inspection is conducted in accordance with local regulations.

Every effort will be made to maintain the anonymity and confidentiality of patients during this study. However, because of the experimental nature of this treatment, the Investigator agrees to allow representatives of the Sponsor, the development partner of the Sponsor, as well as authorized representatives of regulatory authorities to inspect the facilities used in the conduct of this study and to inspect, for purposes of verification, the hospital or clinic records of all patients enrolled in the study.

12.3 Quality Control and Quality Assurance

Quality control procedures will be conducted according to the Sponsor's internal procedures. The study site may be audited by a quality assurance representative of the Sponsor. All necessary data and documents will be made available for inspection.

13.0 REGULATORY, ETHICAL AND LEGAL OBLIGATIONS

13.1 Good Clinical Practice

The study will be performed in accordance with the protocol, guidelines for GCP established by the International Conference on Harmonisation of Technical Requirements for Registration of Pharmaceuticals for Human Use (ICH) and applicable local regulatory requirements and laws and in accordance with European Clinical Trials Directive.

13.2 Ethics Committee Approval

The Investigator must inform and obtain approval from the EC for the conduct of the study at named sites, the protocol, informed consent documents and any other written information that will be provided to the patients and any advertisements that will be used. Written approval must be obtained prior to recruitment of patients into the study and shipment of study drug.

Proposed amendments to the protocol and aforementioned documents must be submitted to the Sponsor for review and approval, then to the EC. Amendments may be implemented only after a copy of the approval letter from the EC has been transmitted to the Sponsor. Amendments that are intended to eliminate an apparent immediate hazard to patients may be implemented prior to receiving Sponsor or EC approval. However, in this case, approval must be obtained as soon as possible after implementation.

Per GCP guidelines, the Investigator will be responsible for ensuring that an annual update is provided to the EC to facilitate continuing review of the study and that the EC is informed about the end of the study. Copies of the update, subsequent approvals and final letter must be sent to the Sponsor.

13.3 Regulatory Authority Approval

The study will be performed in accordance with the requirements of each country’s regulatory authorities (e.g., FDA, European Medicines Agency (EMA) and Health Canada) and will also meet all of the requirements of ICH GCP guidance. Amendments to the protocol will be submitted to the relevant authorities (e.g., FDA, EMA or Health Canada) for approval prior to implementation in accordance with applicable regulations.

13.4 Other Required Approvals

In addition to EC and regulatory authority approval, all other required approvals (e.g., approval from the local research and development board or scientific committee) will be obtained prior to recruitment of patients into the study and shipment of study drug.

13.5 Informed Consent

Informed consent is a process that is initiated prior to the patient's agreeing to participate in the study and continues throughout the patient's study participation. It is the Investigator's responsibility (or designee) to obtain written informed consent from each patient after adequate explanation of the aims, methods, anticipated benefits and potential hazards of the study and before any study procedures are initiated. Each patient should be given a copy of the informed consent document and associated materials. The original copy of the signed and dated informed consent document must be retained at the site and is subject to inspection by representatives of the Sponsor or regulatory authorities. If any amendments occur throughout the course of the study that affect the ICF (i.e., when new study procedures or assessments have been added), all active patients should be reconsented using the same process for the initial consent.

13.6 Patient Confidentiality

The Investigator must ensure that the patient's privacy is maintained. On the eCRF or other documents submitted to the Sponsor, patients will be identified by a patient number only. Documents that are not submitted to the Sponsor (e.g., signed informed consent documents) should be kept in a confidential file by the Principal Investigator.

The Investigator shall permit authorized representatives of the Sponsor, regulatory authorities and ethics committees to review the portion of the patient's medical record that is directly related to the study. As part of the required content of informed consent documents, the patient must be informed that his/her records will be reviewed in this manner.

13.7 Disclosure of Information

Information concerning the study, patent applications, processes, scientific data or other pertinent information is confidential and remains the property of the Sponsor. The Principal Investigator may use this information for the purposes of the study only.

It is understood by the Principal Investigator that the Sponsor will use information obtained in this clinical study in connection with the clinical development program, and therefore may disclose it as required to other clinical investigators and to regulatory authorities. In order to allow the use of the information derived from this clinical study, the Principal Investigator understands that he/she has an obligation to provide complete test results and all data obtained during this study to the Sponsor.

Verbal or written discussion of results prior to study completion and full reporting should only be undertaken with written consent from the Sponsor.

13.8 Publication of Study Data

The conditions regulating dissemination of the information derived from this study are described in the Clinical Trial Agreement.

14.0 ADHERENCE TO THE PROTOCOL

Investigators must apply due diligence to avoid protocol deviations, and the Sponsor (and designee[s]) will not pre-authorize deviations. If the Investigator believes a change to the protocol would improve the conduct of the study, this must be considered for implementation in a protocol amendment. Protocol deviations will be recorded.

14.1 Amendments to the Protocol

Only the Sponsor may modify the protocol. The only exception is when the Investigator considers that a patient's safety is compromised without immediate action. In these circumstances, immediate approval of the chairman of the EC must be sought, and the Investigator should inform the Sponsor and the full EC within 5 working days after the emergency occurred. All amendments that have an impact on patient risk or the study objectives or require revision of the informed consent document must receive approval from the EC prior to implementation.

15.0 REFERENCES

Aaronson NK, Ahmedzai S, Bergman B, et al. The European Organization for Research and Treatment of Cancer QLQ-C30: a quality-of-life instrument for use in international clinical trials in oncology. J Natl Cancer Inst. 1993;3;85(5):365-76.

American Society for Clinical Pathology (ASCP), the College of American Pathologists (CAP), American Society of Clinical Oncology (ASCO) and the Association for Molecular Pathology (AMP): Guideline on the Evaluation of Molecular Markers for Colorectal Cancer Expert Panel Draft Recommendations Summary for Open Comment Period. Available at: http://www.amp.org/committees/clinical_practice/documents/20150327CRCMMDraftRecommendationsforOCP-UPDATEDfinaldraft_001.pdf. Accessed April, 2016.

Amir E, Seruga B, Kwong R, et al. Poor correlation between progression-free and overall survival in modern clinical trials: Are composite endpoints the answer? Eur J Cancer. 2012;48(3):385-8.

Baselga J, Trigo JM, Bourhis J, et al. Phase 2 multicenter study of the anti-epidermal growth factor receptor monoclonal antibody cetuximab in combination with platinum-based chemotherapy in patients with platinum-refractory metastatic and/or recurrent squamous cell carcinoma of the head and neck. J Clin Oncol. 2005;23:5568-77.

Bekaii-Saab, T and Wu C. Seeing the forest through the trees: A systemic review of the safety and efficacy of combination chemotherapies used in the treatment of metastatic colorectal cancer. Crit Rev Onc/Hem. 2014;91:9-34.

Bokemeyer C, Van Cutsem E, Rougier P, et al. Addition of cetuximab to chemotherapy as first-line treatment for KRAS wild-type metastatic colorectal cancer: Pooled analysis of the CRYSTAL and OPUS randomised clinical trials. Eur J Cancer 2012;48:1466-75.

Bonner JA, Harari PM, Giralt J, et al. Radiotherapy plus cetuximab for squamous-cell carcinoma of the head and neck. N Engl J Med. 2006;354:567-78.

Clarke S, Yip S, Brown C, et al. Single-agent irinotecan or 5-fluorouracil and leucovorin (FOLFIRI) as second-line chemotherapy for advanced colorectal cancer; results of a randomised phase II study (DaVINCI) and meta-analysis. Eur J Cancer 2011;47:1826-36.

Clinical Trials Facilitation Group (CTFG) Recommendations related to contraception and pregnancy testing in clinical trials. Final Version 2014-09-15; http://www.hma.eu/fileadmin/dateien/Human_Medicines/01About_HMA/Working_Groups/CTFG/2014_09_HMA_CTFG_Contraception.pdf

Corcoran RB, Ebi H, Turke AB, et al. EGFR-mediated reactivation of MAPK signaling contributes to insensitivity of BRAF-mutant colorectal cancers to RA F inhibition with vemurafenib. Cancer Discov. 2012;2(3):227-35.

Corcoran RB, Atreya C, Falchook G, et al. Combined BRAF and MEK inhibition with dabrafenib and trametinib in BRAF V600-mutant colorectal cancer. J Clin Oncol. 2015;33:1-10.

Cremolini C, Loupakis F, Antoniotti C, et al. FOLFOXIRI plus bevacizumab versus FOLFIRI plus bevacizumab as first-line treatment of patients with metastatic colorectal cancer: updated overall survival and molecular subgroup analyses of the open-label, phase 3 TRIBE study. Lancet Oncol. 2015;16:1306-1315.

Cunningham D, Humblet Y, Siena S, et al. Cetuximab monotherapy and cetuximab plus irinotecan in irinotecan-refractory metastatic colorectal cancer. N Engl J Med. 2004;351:337-45.

De Roock W, Claes B, Bernasconi D, et al. Effects of KRAS, BRAF, NRAS, and PIK3CA mutations on the efficacy of cetuximab plus chemotherapy in chemotherapy-refractory metastatic colorectal cancer: a retrospective consortium analysis. Lancet Oncol. 2010;11:753-62.

Di Nicolantonio F, Martini M, Molinari F. Wild-type BRAF is required for response to panitumumab or cetuximab in metastatic colorectal cancer. J Clin Oncol. 2008;26:5705-12.

Dummer R, Robert C, Nyakas M, et al. Initial results from a phase I, open-label, dose escalation study of the oral BRAF inhibitor LGX818 in patients with BRAF V600 mutant advanced or metastatic melanoma. J Clin Oncol. 2013;31:suppl;abstr 9028.

Eisenhauer EA, Therasse P, Bogaerts J, et al. New response evaluation criteria in solid tumours: revised RECIST guideline (version 1.1). Eur J Cancer 2009;45(2):228-47.

Flaherty KT, Puzanov I, Kim KB, et al. Inhibition of mutated, activated BRAF in metastatic melanoma. N Engl J Med. 2010;363:809-19.

Fuchs CS, Marshall J, Mitchell E, Wierzbicki R, Ganju V, Jeffery M, Schulz J, Richards D, Soufi-Mahjoubi R, Wang B, Barrueco J. Randomized, controlled trial of irinotecan plus infusional, bolus, or oral fluoropyrimidines in first-line treatment of metastatic colorectal cancer: results from the BICC-C Study.J Clin Oncol. 2007;25(30):4779-86.

Gomez-Roca CA, Delord J, Robert C, et al. Encorafenib (LGX818), an oral BRAF inhibitor, in patients (pts) with BRAF V600E metastatic colorectal cancer (mCRC): Results of dose expansion in an open-label, phase 1 study. Ann Oncol. 2014;25(suppl 4):iv167-iv209.

Griggs JJ, Mangu PB, Anderson H, et al. Appropriate chemotherapy dosing for obese adult patients with cancer: American Society of Clinical Oncology clinical practice guideline. J Clin Oncol. 2012;30:1553-61.

Grothey A and Goldberg RM. A review of oxaliplatin and its clinical use in colorectal cancer. Expert Opin Pharmacother. 2004; 5(10):2159-70.

Heinemann V, von Weikersthal LF, Decker T, et al. FOLFIRI plus cetuximab versus FOLFIRI plus bevacizumab as first-line treatment for patients with metastatic colorectal cancer (FIRE-3): a randomised, open-label, phase 3 trial. Lancet Oncol. 2014;15:1065-75.

Holladay JT. Visual acuity measurements. J Cataract Refract Surg. 2004;30:287-90.

Hong D, Morris V, El Osta B, et al. Phase 1b study of vemurafenib in combination with irinotecan and cetuximab in patients with BRAF-mutated metastatic colorectal cancer and advanced cancers. J Clin Oncol. 2015;33(suppl; abstr 3511).

Inlyta®: EPAR Product Information. European Medicines Agency. European public assessment report (EPAR). Available from:
http://www.ema.europa.eu/docs/en_GB/document_library/EPAR_-_Product_Information/human/002406/WC500132188.pdf. Accessed August, 2015.

Kefford R, Arkenau MP, Brown M, et al. Phase 1/2 study of GSK2118436, a selective inhibitor of oncogenic mutant BRAF kinase, in patients with metastatic melanoma and other solid tumors. J Clin Oncol. 2010;28:15s (suppl; abstr 8503).

Kopetz S, Desai J, Chan JR, et al. PLX4032 in metastatic colorectal cancer patients with mutant BRAF tumors. J Clin Oncol. 2010;28:15s (suppl abstr 3534).

Kopetz S, McDonough SL, Lenz H-J et al. Randomized trial of irinotecan and cetuximab with or without vemurafenib in BRAF-mutant metastatic colorectal cancer (SWOG S1406). J Clin Oncol. 2017;35:4s (suppl abstr 3505).

Krishnan G, D'Silva K, Al-Janadi A.l. Cetuximab-related tumor lysis syndrome in metastatic colon carcinoma. J Clin Oncol. 2008;26(14):2406-8.

Lacouture ME, Anadkat MJ, Bensadoun RJ et al. Clinical practice guidelines for the prevention and treatment of EGFR inhibitor-associated dermatologic toxicities. Support Care Cancer 2011;19:1079–95.

Lan KKG, DeMets DL. Discrete sequential boundaries for clinical trials. Biometrika. 1983; 70:659-63.

Larkin J, Ascierto PA, Dréno B, et al. Combined vemurafenib and cobimetinib in BRAF-mutated melanoma. N Eng J Med. 2014;371:1867-76.

Long GV, Stroyakovskiy D, Gogas H, et al. Dabrafenib and trametinib versus dabrafenib and placebo for Val600 BRAF-mutant melanoma: a multicenter, double-blind, phase 3 randomised controlled trial. Lancet 2015;386:444-51.

Martín-Martorell P, Roselló S, Rodríguez-Braun E, et al. Biweekly cetuximab and irinotecan in advanced colorectal cancer patients progressing after at least one previous line of chemotherapy: results of a Phase 2 single institution trial. Br J Cancer 2008;99:455-8.

Mendelsohn J and Baselga J. Epidermal growth factor receptor targeting in cancer. Semin Oncol. 2006;33:369-85.

Menzies A and Long G. Dabrafenib and trametinib, alone and in combination for BRAF-mutant metastatic melanoma. Clin Cancer Res. 2014;20:2025-43.

Mesa AM, Gotlib J, Gupta V, et. al. Effect of ruxolitinib therapy on myelofibrosis-related symptoms and other patient-reported outcomes in COMFORT-1: A randomized, double-blind, placebo-controlled trial. J Clin Oncol. 2013;31:1285-92.

Modest DP, Jung A, Moosmann N, et al. The influence of *KRAS* and *BRAF* mutations on the efficacy of cetuximab-based first-line therapy of metastatic colorectal cancer: An analysis of the AIOKRK-0104-trial. Int J Cancer 2012;131:980-6.

Morris V, Overman MJ, Jiang Z-Q, et. al. Progression-free survival remains poor over sequential lines of systemic therapy in patients with BRAF-mutated colorectal cancer. Clin Col Cancer Res. 2014;13:164-71.

Nardone B, Hensley JR, Kulik L, et al. The effect of hand-foot skin reaction associated with the multikinase inhibitors sorafenib and sunitinib on health-related quality of life. J Drugs Dermatol. 2012; 11(11):e61-5.

National Comprehensive Cancer Network Guidelines. Version 2. 2016. http://www.nccn.org/professionals/physician_gls/pdf/colon.pdf. Accessed April 5, 2016.

Oliner KS, Douillard J, Siena S, et al. Analysis of KRAS/NRAS and BRAF mutations in the phase III PRIME study of panitumumab (pmab) plus FOLOFX versus FOLFIRI as first-line treatment (tx) for metastatic colorectal cancer (mCRC). J Clin Oncol. 2013;31(suppl; abstr 3511).

Peeters M, Price T, Cervantes A, et al. Randomized phase III study of panitumumab with fluorouracil, leucovorin, and irinotecan (FOLFIRI) compared with FOLFIRI alone as second-line treatment in patients with metastatic colorectal cancer. J Clin Oncol. 2010;28:4706-13.

Peeters M, Oliner KS, Price T, et al. Updated analysis of *KRAS/NRAS* and *BRAF* mutations in study 20050181 of panitumumab (pmab) plus FOLFIRI for second-line treatment (tx) of metastatic colorectal cancer (mCRC). J Clin Oncol 32:5s, 2014 (suppl; abstr 3568).

Peeters M, Price TJ, Cervantes A, et al. Final results from a randomized phase 3 study of FOLFIRI {+/-} panitumumab for second-line treatment of metastatic colorectal cancer. Ann Oncol. 2014;25(1):107-16. doi: 10.1093/annonc/mdt523. Erratum in: Ann Oncol. 2014 Mar;25(3):757.

Pfeiffer P, Nielsen D, Bjerregaard J, et al. Biweekly cetuximab and irinotecan as third-line therapy in patients with advanced colorectal cancer after failure to irinotecan, oxaliplatin and 5-fluorouracil. Ann Oncol. 2008;19:1141-5.

Prahallad A, Sun C, Huang S, et al. Unresponsiveness of colon cancer to BRAF(V600E) inhibition through feedback activation of EGFR. Nature 2012;100:100-4.

Rabin R and de Charro F. EQ-5D: a measure of health status from the EuroQol Group. Ann Med. 2001;33:337–43.

Robert C, Arnault JP, Mateus C, et al. RAF inhibition and induction of cutaneous squamous cell carcinoma. Curr Opin Oncol. 2011;23(2):177-82.

Rowland A, Dias MM, Wiese MD, Kichendasse G, McKinnon RA, Karapetis CS et al. Meta-analysis of BRAF mutation as a predictive biomarker of benefit from anti-EGFR monoclonal antibody therapy for RAS wild-type metastatic colorectal cancer. Brit J Canc. 2015;112:1888-94.

Seymour MT, Brown SR, Middleton G et al. Panitumumab and irinotecan versus irinotecan alone for patients with KRAS wild-type, fluorouracil-resistant advanced colorectal cancer (PICCOLO): a prospectively stratified randomised trial. Lancet Oncol. 2013;14:749-59.

Shah R. Tyrosine kinase inhibitor-induced interstitial lung disease: Clinical features, diagnostic challenges, and therapeutic dilemmas. Drug Saf 2016;39:1073-1091.

Siegel RL, Miller KD, and Jemal A. Cancer Statistics, 2015. CA Cancer J Clin 2015;65:5-29.

Smith T, Bohlke K, Lyman GH. Recommendations for the use of WBC growth factors: American Society of Clinical Oncology clinical practice guideline update. J Clin Oncol. 2015;33:1-16.

Sobrero AF, Maurel J, Fehrenbacher L, et al. EPIC: Phase 3 trial of cetuximab plus irinotecan after fluoropyrimidine and oxaliplatin failure in patients with metastatic colorectal cancer. J Clin Oncol. 2008;26:2311-9.

Sorbye H, Dragomir A, Sundström M, Pfeiffer P, Thunberg U, Bergfors M et al. High BRFA mutation frequency and marked survival differences in subgroups according to KRAS/BRAF mutation status and tumor tissue availability in a prospective population-based metastatic colorectal cancer cohort. PLoS One 2015;10(6)e0131046.

Sullivan RJ, Weber JS, Patel SP, et al. A phase Ib/II study of BRAF inhibitor (BRAFi) encorafenib (ENCO) plus MEK inhibitor (MEKi) binimetinib (BINI) in cutaneous melanoma patients naïve to BRAFi treatment. J Clin Oncol. 2015;33:suppl;abstr 9007.

Tabernero J, Yoshino T, Cohn A, et al. Ramucirumab versus placebo in combination with second-line FOLFIRI in patients with metastatic colorectal carcinoma that progressed during or after first-line therapy with bevacizumab, oxaliplatin, and a fluoropyrimidine (RAISE): a randomised, double-blind, multicenter, phase 3 study. Lancet Oncol. 2015;16:499-508.

Thakur MD and Stuart DD. Molecular pathways: Response and resistance to BRAF and MEK inhibitors in BRAFV600E tumors. Clin Cancer Res. 2014;20:1074-80.

Tol J and Punt CJ. Monoclonal antibodies in the treatment of metastatic colorectal cancer: a review. Clin Ther 2010;32(3):437-53.

Trussell J. Contraceptive failure in the United States. Contraception 2004;70:89-96.

Van Cutsem E, Köhne C-H, Láng I, et al. Cetuximab plus irinotecan, fluorouracil, and leucovorin as first-line treatment for metastatic colorectal cancer: Updated analysis of overall survival according to tumor KRAS and BRAF mutation status. J Clin Oncol. 2011;29:2011-9.

Van Cutsem E, Cervantes A, Nordlinger B, et al. Metastatic colorectal cancer: ESMO clinical practice guidelines for diagnosis, treatment and follow-up. Ann Oncol. 2014;25 (suppl 3):iii1-iii9.

Van Cutsem E, Atreya C, André T. Updated results of the MEK inhibitor trametinib (T), BRAF inhibitor dabrafenib (D), and anti-EGFR antibody panitumumab (P) in patient (pts) with BRAF V600E mutated (BRAFm) metastatic colorectal cancer (mCRC). Ann Oncol. 2015;26(suppl 4):iv117-iv121.

Van Moos R, Thuerlimann B, Chair M, et al. (2008) Pegylated liposomal doxorubicin-associated hand-foot syndrome: Recommendations of an international panel of experts. Eur J Cancer 2008;1016:1-10.

Venook AP, Niedzwiecki D, Lenz HJ, et al. CALGB/SWOG 80405: Phase III trial of irinotecan/5-FU/leucovorin (FOLFIRI) or oxaliplatin/5-FU/leucovorin (mFOLFOX6) with bevacizumab (BV) or cetuximab (CET) for patients (pts) with KRAS wild-type (wt) untreated metastatic adenocarcinoma of the colon or rectum (MCRC). J Clin Oncol. 2014;32:suppl;abstr LBA3.

Vermorken JB, Mesia R, Rivera F, et al. Platinum-based chemotherapy plus cetuximab in head and neck cancer. N Engl J Med. 2008;359:1116-27.

Wiens BL, Dmitrienko A. The fallback procedure for evaluating a single family of hypotheses. J Biopharm Stat. 2005; 15:929-42.

Yang H, Higgins B, Kolinsky K, et al. Antitumor activity of BRAF inhibitor vemurafenib in preclinical models of BRAF-mutant colorectal cancer. Cancer Res. 2012;72(3):779-89.

Yeager R, Cercek A, O'Reilly E, et al. Pilot study of vemurafenib and panitumumab combination therapy in patients with BRAF V600E mutated metastatic colorectal cancer. J Clin Oncol. 2015;33 (suppl 3; Abstr 611).

Yokota T, Ura T, Shibata N, et al. BRAF mutation is a powerful prognostic factor in advanced and recurrent colorectal cancer. Br J Cancer 2011;104:856-62.

Appendix 1: Recommended Guidelines for the Management of Cetuximab-induced, Encorafenib-induced and/or Binimetinib-induced Skin Toxicity

Clinical judgment and experience of the treating physician should guide the management plan of each patient. In general, the following interventions are in addition to the cetuximab-induced rash and the encorafenib-induced and/or binimetinib-induced rash dosing guidelines provided in Table 17 and Table 16, respectively, of the protocol.

The Initial Rash Treatment Regimen may be initiated as prophylactic treatment 24 hours prior to the first treatment, or later as needed to treat mild rash (CTCAE Grade 1).

Initial Rash Treatment Regimen:

- Application of topical agents to the most commonly affected skin areas such as face, scalp, neck, upper chest and upper back. Topical agents include the following:
    - Non-oily sunscreen (PABA-free, SPF ≥ 30, UVA/UVB protection);
    - Topical steroids, preferably mometasone cream (e.g., Elocon®);
    - Topical erythromycin (e.g., Eryaknen®);
    - Topical pimocrolimus.

Note: Topical agents should be applied on a daily basis starting on Day 1 of study treatment or 24 hours prior to first treatment, and more often as needed.

- Possibly oral doxycycline (100 mg daily) for the first 2 to 3 weeks of study drug administration.

Other effective medications are antihistamines, other topical corticosteroids, other topical antibiotics and low-dose systemic corticosteroids.

The treatment algorithm based on CTCAE grade is as follows:

Mild Rash (CTCAE Grade 1) Treatment Regimen:

- Initiate Initial Rash Treatment Regimen, if not already started.
- Use of topical corticosteroid (e.g., mometasone cream) and/or topical antibiotic (e.g., erythromycin 2%) is recommended.
- The patient should be reassessed within a maximum of 2 weeks, or as per Investigator opinion.

Moderate Rash (CTCAE Grade 2) Treatment Regimen:

- Use of topical erythromycin or clindamycin (1%) plus topical mometasone or topical pimecrolimus (1% cream) plus oral antibiotics, such as lymecycline (408 mg QD), doxycycline (100 mg BID) or minocycline (50 to 100 mg BID).
- Although there has been no evidence of phototoxicity or photosensitivity in patients treated with binimetinib, doxycycline (or minocycline as second line) should be used with thorough UV protection (i.e., avoidance of direct exposure to sunlight, use of sunscreen and sunglasses, etc.).
- Use of acitretin is not recommended.

Severe Rash (CTCAE Grade 3-4) Treatment Regimen:

CTCAE Grade 3:

- In addition to the interventions recommended for moderate rash, consider oral prednisolone at a dose of 0.5 mg/kg. Upon improvement, taper the dose in a stepwise manner (25 mg for 7 days, subsequently decreasing the dose by 5 mg/day every day).
- Alternatively, in addition to the interventions recommended for moderate rash, consider oral isotretinoin (low dose, i.e., 0.3 to 0.5 mg/kg) (Lacouture et al. 2011).
- Use of acitretin is not recommended.

CTCAE Grade 4 Treatment Regimen:

- Immediately discontinue the patient from study drug and treat the patient with oral or topical medications (see recommendation CTCAE Grade 3).

Symptomatic Treatment Regimen:

It is strongly recommended that patients who develop rash/skin toxicities receive symptomatic treatment:

- For pruritic lesions: use cool compresses and oral antihistamine agents.
- For fissuring: use Monsel's solution, silver nitrate or zinc oxide cream. If not sufficient, use mild corticosteroid ointments or ointments containing a combination of corticosteroid and antibiotic such as Fucicort®.
- For desquamation: use emollients that are mild pH 5/neutral (recommended to contain 10% urea).

- For paronychia: use antiseptic bath and local potent corticosteroids, use oral antibiotics, and, if no improvement is seen, refer to a dermatologist or surgeon.
- For infected lesions: obtain bacterial and fungal cultures and treat with topical or systemic antibiotics, if indicated, based on sensitivity of culture.

References:

Lacouture ME, Anadkat MJ, Bensadoun RJ et al. Clinical practice guidelines for the prevention and treatment of EGFR inhibitor-associated dermatologic toxicities. Support Care Cancer 2011;19:1079–95.

Appendix 2: Recommended Guidelines for the Management of Encorafenib-induced Hand-foot skin reactions (HFSR)

Clinical judgment and experience of the treating physician should guide the management plan of each patient. In addition to the HFSR dosing guidelines in Table 16 of the protocol, the following algorithm is recommended for the management of HFSR based on the severity (CTCAE grading, see Table 14) of HFSR (adapted from Nardone et al. 2012).

Algorithm for the Management of HFSR Based on the Severity of HFSR

HFSR severity	Intervention
**No HFSR**	**Maintain Frequent Contact with physician to ensure early diagnosis of HFSR**
**Therapy initiation**	Full body-skin examination, pedicure, evaluation by podiatrist or orthotist; wear thick cotton gloves and/or socks; avoid hot water, constrictive footwear and excessive friction
	**If symptoms develop, proceed to next step**
**Grade 1**	**Maintain current dose of BRAF inhibitor; monitor patient for change in severity**
Minimal skin changes or dermatitis without pain e.g.:<br>• Numbness<br>• Tingling<br>• Dysesthesia<br>• Paresthesia<br>• Erythema<br>• Edema<br>• Hyperkeratosis<br>No interference with ADL	Avoid hot water; use moisturizing cream for relief; wear thick cotton gloves and/or socks; use a 20-40% urea , salicylic acid 3-6%; ammonium lactate 12 % or lactic acid 12 % based creams to aid exfoliation.
	**If symptoms worsen, proceed to next steps**
**Grade 2**	**Maintain current dose of BRAF inhibitor; monitor patient for change in severity**
Skin changes with pain e.g.<br>Peeling<br>• Blisters<br>• Bleeding<br>• Edema<br>• Hyperkeratosis<br>Limited instrumental ADL	Treat as with Grade 1 toxicity, with the following additions: clobetasol 0.05% ointment, 2-4% lidocaine, opiates, NSAIDS, or GABA agonists for pain; follow dose modifications listed in Table 16
	**If no improvement within 15 days, proceed to next steps.**
**Grade 3**	**Interrupt dose until improvement to Grade 0-1**
Severe skin changes with pain e.g.	Treat as in Grades 1 and 2

Peeling<br>• Blisters<br>• Bleeding<br>• Edema<br>• Hyperkeratosis<br>Limiting self-care ADL	Follow dose modifications listed in Table 16

The following supportive care measures for the prevention, and/or management of HFSR summarized in the table below should be instituted along with proper patient education.

Supportive Care for the Prevention and Management of HFSR

Stage	Recommendations
Prior to initiation of study treatment	Educate the patient about the early signs and symptoms of HFSR and discuss the importance of early reporting. HFSR could start as early as 2-5 days after study drug initiation, and mostly expected to occur during the first 2 months of treatment.
Prevention of HFSR for the first 2 months of treatment with encorafenib	Monitor the patient for signs and symptoms of HFSR. Instruct the patient to:<br>- Apply emollient cream regularly to hands and feet: use 20-40% urea , salicylic acid 3-6%; ammonium lactate 12% or lactic acid 12 % based creams<br>- Wear cotton socks or gloves to bed to enhance the absorption of creams<br>- Avoid tight, irritating or ill-fitting clothing and shoes<br>- Avoid repetitive activity or staying in one position for long periods of time<br>- Pat (do not rub) skin dry with towels<br>- Avoid extremes of temperature, pressure and friction<br>- Avoid performing mechanically stressful manual work<br>- Cushioning of callused areas<br>- Use of moisturizing and keratolytic creams to control existing palmar and plantar hyperkeratosis
Treatment of HFSR	1) Ensure that patient follows treatment interruption or dosage reduction guidelines<br>2) Monitor the patient for worsening/resolution of HFSR (Normal frequency monthly, except if patient has Grade 2 or 3 HFSR, where bi weekly- visits are recommended)<br>3) Prescribe analgesics if necessary<br>4) Instruct the patient to:<br>- Continue the use of prevention strategies<br>- Cushion sore skin<br>- For control or relief of pain symptoms, patient may submerge hands and feet in cool water baths or apply cold compresses for relief

[a] Wear loose-fitting clothing made of soft, natural fabrics and shoes that are wide and comfortable. Avoid tight belts, panties and bras.
This table is adapted from (van Moos et al. 2008).

References:

Nardone B, Hensley JR, Kulik L, et al. (2012). The effect of hand-foot skin reaction associated with the multikinase inhibitors sorafenib and sunitinib on health-related quality of life. Journal of Drugs in Dermatology Nov 2011: 11;11:e61-5.

Van Moos R, Thuerlimann B, Chair M, etal. (2008) Pegylated liposomal doxorubicin-associated hand-foot syndrome: Recommendations of an international panel of experts. Eur J Cancer: 1016:1-10.

Appendix 3: Recommended Guidelines for the Management of Binimetinib-induced Diarrhea

Proactively Investigate for Occurrence of Diarrhea and Educate Patient:

1. Remind patients at each visit to contact the Investigator immediately upon the first sign of loose stool or symptoms of abdominal pain. Additionally, at each study visit, each patient should be asked regarding occurrence of diarrhea or diarrhea-related symptoms. If the patient has had symptoms, the patient should be asked regarding the actions taken for these symptoms and re-instruct if indicated.

2. Patients should be instructed on dietary modification and early warning signs of diarrhea and potentially life-threatening illnesses (e.g., severe cramping might be a sign of severe diarrhea; fever with diarrhea might be a sign of infection; fever and dizziness on standing might be a sign of shock).

3. Patients should be educated about what to report to the Investigator (i.e., number of stools, stool composition, stool volume).

Anti-diarrhea Therapy:

In order to effectively manage diarrhea and mitigate the escalation in severity or duration of diarrhea, patient education as outlined above, as well as proper management of diarrhea is important.

Management of diarrhea should be instituted at the first sign of abdominal cramping, loose stools or overt diarrhea. All concomitant therapies /used for treatment of diarrhea must be recorded on the eCRF. It is recommended that patients be provided loperamide tablets and be instructed on the use of loperamide on the first day of binimetinib dosing. In addition to the binimetinib-induced diarrhea dosing guidelines provided in Table 16 of the protocol, these instructions should be provided at each visit and the site should ensure that the patient understood the instructions.

See Table 15 in the protocol to explain the frequency of diarrhea and its relationship to NCI CTCAE, v.4.03 grading and to determine if diarrhea is complicated or uncomplicated.

Rule out Other or Concomitant Causes:

These may include:

- Infection with *Candida, Salmonella, Clostridium difficile, Campylobacter. Giardia, Entamoeba* or *Cryptosporidium* species, which can lead to severe infections in immunosuppressed patients.

- Medication-induced diarrhea.

- Malabsorption/lactose intolerance.
- Fecal impaction, partial bowel obstruction.

For Uncomplicated Grade 1/2 Diarrhea:

- Stop all lactose-containing products and alcohol, and eat frequent small meals that include bananas, rice, applesauce or toast.
- Stop laxatives, bulk fiber (e.g., Metamucil®), and stool softeners (e.g., docusate sodium, Colace®).
- Stop high-osmolar food supplements (e.g., Ensure® Plus, Jevity® Plus [with fiber]).
- Drink 8 to 10 large glasses of clear liquids per day (e.g., water, Pedialyte®, Gatorade®, broth).
- Consider administration of a standard dose of loperamide: initial administration 4 mg, then 2 mg every 4 hours (maximum of 16 mg/day) or after each unformed stool.
- Discontinue loperamide after 12-hours diarrhea-free (Grade 0) interval.
- Consider temporary interruption of binimetinib until resolved to Grade ≤ 1. Re-treatment may then be resumed at current dose level.
- If uncomplicated Grade 1 to Grade 2 diarrhea persists for more than 24 hours, escalate to high-dose loperamide: 2 mg every 2 hours (maximum of 16 mg/day) or after each unformed stool.

Note: Oral antibiotics may be started as prophylaxis for infections under the discretion of the physician.

- If uncomplicated Grade 1 to Grade 2 diarrhea persists after 48 hours of treatment with loperamide, discontinue loperamide and begin a second-line agent which can be an opiate (opium tincture or paregoric), octreotide acetate or steroid (budesonide).

For Complicated Grade 1/2 Diarrhea or Any Grade 3/4 Diarrhea:

- The patient must call the Investigator immediately.
- Temporarily interrupt binimetinib treatment until resolved to Grade ≤ 1. Restart binimetinib at a reduced dose level.

- If loperamide has not been intitiated, initiate loperamide immediately. Initial administration 4 mg, then 2 mg every 4 hours (maximum of 16 mg/day) or after each unformed stool.
- Administer IV fluids and electrolytes as needed. In case of severe hydration, replace loperamide with octreotide acetate.
- Monitor/continue IV fluids and antibiotics as needed. Intervention should be continued until the patient is diarrhea-free for at least 24 hours.
- Hospitalization may need to be considered.

Appendix 4: Snellen Equivalence (Visual Acuity Conversion Chart)

				Distance				Near					
					Snellen Equivalent								
Line Number	Visual Angle (min)	Spatial Frequency (Cyc/deg)	LogMAR	% Central Visual Efficiency	Feet 20/	Meter 6/	Decimal	% Central Visual Efficiency	Inches (14/)	Centimeters (35/)	Revised Jaeger Standard	American Point-Type	"M" Notation
**−3**	**0.50**	**60.00**	**0.30**	**100**	**10**	**3.0**	**2.00**	**100**	**7.0**	**17.5**	–	–	**0.20**
**−2**	**0.63**	**48.00**	**0.20**	**100**	**12.5**	**3.8**	**1.60**	**100**	**8.8**	**21.9**	–	–	**0.25**
**−1**	**0.80**	**37.50**	**0.10**	**100**	**16**	**4.8**	**1.25**	**100**	**11.2**	**28.0**	–	–	**0.32**
**0**	**1.00**	**30.00**	**0.00**	**100**	**20**	**6.0**	**1.00**	**100**	**14.0**	**35.0**	**1**	**3**	**0.40**
**1**	**1.25**	**24.00**	**−0.10**	**95**	**25**	**7.5**	**0.80**	**100**	**17.5**	**43.8**	**2**	**4**	**0.50**
–	1.50	20.00	−0.18	91	30	9.0	0.67	95	21.0	52.5	3	5	0.60
**2**	**1.60**	**18.75**	**−0.20**	**90**	**32**	**9.6**	**0.63**	**94**	**22.4**	**56.0**	**4**	**6**	**0.64**
**3**	**2.00**	**15.00**	**−0.30**	**85**	**40**	**12.0**	**0.50**	**90**	**28.0**	**70.0**	**5**	**7**	**0.80**
**4**	**2.50**	**12.00**	**−0.40**	**75**	**50**	**15.0**	**0.40**	**50**	**35.0**	**87.5**	**6**	**8**	**1.0**
–	3.00	10.00	−0.48	67	60	18.0	0.33	42	42.0	105.0	7	9	1.2
**5**	**3.15**	**9.52**	**−0.50**	**65**	**63**	**18.9**	**0.32**	**40**	**44.1**	**110.3**	**8**	**10**	**1.3**
–	3.50	8.57	−0.54	63	70	21.0	0.29	32	49.0	122.5	–	–	1.4
**6**	**4.00**	**7.50**	**−0.60**	**60**	**80**	**24.0**	**0.25**	**20**	**56.0**	**140.0**	**9**	**11**	**1.6**
**7**	**5.00**	**6.00**	**−0.70**	**50**	**100**	**30.0**	**0.20**	**15**	**70.0**	**175.0**	**10**	**12**	**2.0**
–	5.70	5.26	−0.76	44	114	34.2	0.18	12	79.8	199.5	11	13	2.3
**8**	**6.25**	**4.80**	**−0.80**	**40**	**125**	**37.5**	**0.16**	**10**	**87.5**	**218.8**	**12**	**14**	**2.5**
–	7.50	4.00	−0.88	32	150	45.0	0.13	6	105.0	262.5	–	–	3.0
**9**	**8.00**	**3.75**	**−0.90**	**30**	**160**	**48.0**	**0.13**	**5**	**112.0**	**280.0**	**13**	**21**	**3.2**
**10**	**10.00**	**3.00**	**−1.00**	**20**	**200**	**60.0**	**0.10**	**2**	**140.0**	**350.0**	**14**	**23**	**4.0**
**11**	**12.50**	**2.40**	**−1.10**	**17**	**250**	**75.0**	**0.08**	**0**	**175.0**	**437.5**	–	–	**5.0**
–	15.00	2.00	−1.18	16	300	90.0	0.07	**0**	210.0	525.0	–	–	6.0
**12**	**16.00**	**1.88**	**−1.20**	**15**	**320**	**96.0**	**0.06**	**0**	**224.0**	**560.0**	–	–	**6.4**
**13**	**20.00**	**1.50**	**−1.30**	**10**	**400**	**120.0**	**0.05**	**0**	**280.0**	**700.0**	–	–	**8.0**
**16**	**40.00**	**0.75**	**−1.60**	**5**	**800**	**240.0**	**0.03**	**0**	**560.0**	**1400.0**	–	–	**16.0**
**20**	**100.00**	**0.30**	**−2.00**	**0**	**2000***	**600.0**	**0.01**	**0**	**1400.0**	**3500.0**	–	–	**40.0**
**30**	**1000.00**	**0.03**	**−3.00**	**0**	**20000†**	**6000.0**	**0.001**	**0**	**14000.0**	**35000.0**	–	–	**400.0**

Bold values are standard logMAR progression.
LogMAR = logarithm of the minimum angle of resolution
*20/2000 is equivalent to counting fingers @ 2 feet
†20/20000 is equivalent to hand motion @ 2 feet

References:

Holladay JT. Visual acuity measurements. J Cataract Refract Surg. 2004;30:287-90.

Appendix 5: List of Concomitant Medications

Table A: List of CYP450 Substrates to be Used with Caution*

CYP2C8	CYP2C9	CYP2C19	CYP3A**	
Amodiaquine	Acenocoumarol	Clopidogrel	Alfentanil [1,2]	Ergotamine [2]
Cerivastatin	Celecoxib	Diazepam	Alpha-dihydroergocryptine [1]	Everolimus [1]
Repaglinide	Diclofenac	Esoprazole	Alprazolam	Felodipine [1]
Rosiglitazone	Glipizide	Lansoprazole	Amlodipine	Fentanyl [2]
Torasemide	Irbesartan	Moclobemide	Aplaviroc	Fluticasone [1]
	Losartan	Omeprazole	Aprepitant [1]	Indinavir [1]
	Phenytoin [2]	Pantoprazole	Aripiprazole	Lopinavir [1]
	Piroxicam	Phenobarbitone	Atorvastatin	Lovastatin [1]
	S-ibuprofen	Phenytoin [2]	Boceprevir	Maraviroc [1]
	Sulfamethoxazole	Proguanil	Brecanavir	Midazolam [1]
	Tolbutamide	Rabeprazole	Brotizolam [1]	Nifedipine
	Torasemide	S-mephenytoin	Budesonide [1]	Nisoldipine
			Buspirone [1]	Nitrendipine
			capravirine	Perospirone [1]
			casopitant	Quinine
			Conivaptan [1]	Saquinavir [1]
			Cyclosporine [2]	Sildenafil [1]
			Darifenacin [1]	Simvastatin [1]
			Darunavir [1]	Sirolimus [1,2]
			Diazepam	Telaprevir
			Diergotamine [2]	Tipranavir [1]
			Diltiazem	Tolvaptan
			Ebastine [1]	Triazolam [1]
			Eletriptan [1]	Verapamil
			Eplerenone [1]	

*Table was compiled from the Indiana University School of Medicine's "Clinically Relevant" table, a list by the United States Food and Drug Administration (FDA) fda.gov/Drugs/DevelopmentApprovalProcess/DevelopmentResources/DrugInteractionsLabeling/ucm080499.htm, and the University of Washington's Drug Interaction Database.

1 Sensitive substrates: Drugs whose plasma area under concentration-time curve (AUC) values have been shown to increase 5-fold or higher when co-administered with a potent inhibitor of the respective enzyme.

2 Substrates with narrow therapeutic index (NTI): Drugs whose exposure-response indicates that increases in their exposure levels by the concomitant use of potent inhibitors may lead to serious safety concerns (e.g., TdP).

Table B: List of CYP450 Substrates to be Used with Caution – CYP2B6

**CYP2B6***
bupropion [1]
cyclophosphamide
Efavirenz [1]
Ifosfamide
Methadone
Thiotepa

*Table was compiled from the Indiana University School of Medicine's "Clinically Relevant" table, a list by the FDA (http://.fda.gov/Drugs/DevelopmentApprovalProcess/DevelopmentResources/DrugInteractionsLabeling/ucm080499.htm) and the University of Washington's Drug Interaction Database.

[1] Sensitive substrates: The area under the concentration-time curves (AUCs) of these substrates were not increased by 5-fold or more with a CYP2B6 inhibitor, but they represent the most sensitive substrates studied with available inhibitors evaluated to date.

Table C: Strong/Moderate CYP3A4 Inhibitors and CYP3A4 Inducers to be Prohibited or Administered with Caution when Co-administered with Encorafenib

**Strong Inhibitors (prohibited)**	
boceprevir	nefazodone
Clarithromycin	Nelfinavir
Conivaptan	posaconazole
Indinavir	Ritonavir
Itraconazole	Saquinavir
Ketoconazole	telithromycin
Lopinavir	troleandomycin
Telaprevir	Grapefruit juice (citrus paradisi fruit juice)
Mibefradil	Voriconazole
**Moderate inhibitors (use with caution)**	
Ciprofloxacin	Erythromycin
Fluconazole	Amprenavir
Verapamil	Imatinib
Atazanavir	Schisandra sphenanthera
Aprepitant	Casopitant
Cyclosporine	Cimetidine
Tofisopam	Dronedarone
Fosamprenavir	Darunavir
Diltiazem	
**Strong Inducers (use with caution)**	
Avasimibe	Rifampin
Carbamazepine	St. John's wort
Phenytoin	

Reproduced from
fda.gov/Drugs/DevelopmentApprovalProcess/DevelopmentResources/DrugInteractionsLabeling/ucm080499.htm

Table D: Substrates of BCRP, OAT, OCT s and OATPs to be Administered with Caution

	Substrates
BCRP	imatinib, irrinotecan, lapatinib, methotrexate, mitoxantrone, rosuvastatin, sulfasalazine, topotecan
P-gp	Aliskiren, ambrisentan, colchicine, dabigatran etexilate, digoxin, everolimus, fexofenadine, imatinib, lapatinib, maraviroc, nilotinib, posaconazole, ranolazine, saxagliptin, sirolimus, sitagliptin, talinolol, tolvaptan, topotecan
OCT2	Amantadine, amiloride, cimetidine, dopamine, famotidine, memantine, metformin, pindolol, procainamide, ranitidine, varenicline, oxaliplatin
OAT1	Adefovir, captopril, furosemide, lamivudine, methotrexate, oseltamivir, tenofovir, zalcitabine, zidovudine
OAT3	Acyclovir, bumetanide, ciprofloxacin, famotidine, furosemide, methotrexate, zidovudine, oseltamivir acid, (the active metabolite of oseltamivir), penicillin G, pravastatin, rosuvastatin, sitagliptin
OATP1B1	Atrasentan, atorvastatin, bosentan, ezetimibe, fluvastatin, glyburide, SN-38 (active metabolite of irinotecan), rosuvastatin, simvastatin acid, pitavastatin, pravastatin, repaglinide, rifampin, valsartan, olmesartan
OATP1B3	Atorvastatin, rosuvastatin, pitavastatin, telmisartan, valsartan, olmesartan

Reproduced from
fda.gov/Drugs/DevelopmentApprovalProcess/DevelopmentResources/DrugInteractionsLabeling/ucm080499.htm

Table E: Pg-P and BCRP Inhibitors/Inducers to be Used with Caution

Transporter	Gene	Inhibitor [1]	Inducer [2]
P-gp	ABCB1	Amiodarone, azithromycin,captopril, carvedilol, clarithromycin, conivaptan, cyclosporine, diltiazem, dronedarone, erythromycin, felodipine, itraconazole, ketoconazole, lopinavir and ritonavir, quercetin, quinidine, ranolazine, verapamil	Avasimibe,carbamazepine,phenytoin, rifampin, St John’s wort, tipranavir/ritonavir
BCRP	ABCG2	Cyclosporine, elacridar (GF120918), eltrombopag, gefitinib	Not known

Reproduced from
fda.gov/Drugs/DevelopmentApprovalProcess/DevelopmentResources/DrugInteractionsLabeling/ucm080499.htm

1 Inhibitors listed for P-gp are those that showed > 25% increase in digoxin area under the concentration-time curve (AUC) or otherwise indicated if substrate is other than digoxin.

2 Inducers listed for P-gp are those that showed > 20% decrease in digoxin AUC or otherwise indicated if substrate is other than digoxin.

Table F: List of Inhibitors of UGT1A1 to be Used with Caution

**Inhibitors of UGT1A1**	atazanavir, erlotinib, flunitrazepam, gemfibrozil, indinavir, ketoconazole, nilotinib, pazopanib, propofol, regorafenib, sorafenib

Table G: List of Potential QT Prolonging Drugs[1]

Drug	QT risk[2]	Comment
Alfuzosin	possible risk	
Amantadine	possible risk	
Amiodarone	known risk	Females > Males, TdP risk regarded as low
Amitriptyline	conditional risk	Risk of TdP with overdosage. Substrate of CYP2C19

Drug	QT risk[2]	Comment
Arsenic trioxide	known risk	
Astemizole	known risk	No Longer available in U.S. Substrate for 3A4
Atazanavir	possible risk	
Azithromycin	possible risk	Rare reports
Bepridil	known risk	Females > Males
Chloral hydrate	possible risk	
Chloroquine	known risk	
Chlorpromazine	known risk	
Ciprofloxacin	conditional risk	Drug metabolism inhibitor- Risk for drug interactions
Cisapride	known risk	No longer available in the U.S.; available in Mexico. Substrate for 3A4
Citalopram	known risk	
Clarithromycin	known risk	Substrate for 3A4
Clomipramine	conditional risk	
Clozapine	possible risk	
Desipramine	conditional risk	Risk of TdP with overdosage
Diphenhydramine	conditional risk	Risk of QT increase/TdP in overdosages
Disopyramide	known risk	Females > Males
Dofetilide	known risk	
Dolasetron	possible risk	
Domperidone	known risk	Not available in the U.S.
Doxepin	conditional risk	
Dronedarone	possible risk	Substrate for 3A4
Droperidol	known risk	
Eribulin	possible risk	
Erythromycin	known risk	Females>Males. Substrate for 3A4
Escitalopram	possible risk	
Famotidine	possible risk	
Felbamate	possible risk	
Fingolimod	possible risk	
Flecainide	known risk	
Fluconazole	conditional risk	Drug metabolism inhibitor- Risk for drug interactions
Fluoxetine	conditional risk	
Foscarnet	possible risk	
Fosphenytoin	possible risk	
Galantamine	conditional risk	
Gatifloxacin	possible risk	
Gemifloxacin	possible risk	
Granisetron	possible risk	
Halofantrine	known risk	Females>Males
Haloperidol	known risk	When given intravenously or at higher-than- recommended doses, risk of sudden death, QT prolongation and torsades increases. Substrate for 3A4

Drug	QT risk[2]	Comment
Ibutilide	known risk	Females>Males
Imipramine	conditional risk	Risk of TdP in overdosage
Indapamide	possible risk	
Isradipine	possible risk	
Itraconazole	conditional risk	Drug metabolism inhibitor- Risk for drug interactions
Ketoconazole	conditional risk	Drug metabolism inhibitor
Levofloxacin	possible risk	
Levomethadyl	known risk	Not available in the U.S.
Lithium	possible risk	
Mesoridazine	known risk	
Methadone	known risk	Females>Males. Substrate for 3A4
Moexipril/HCTZ	possible risk	
Moxifloxacin	known risk	
Nicardipine	possible risk	
Nortriptyline	conditional risk	
Octreotide	possible risk	
Ofloxacin	possible risk	
Ondansetron	possible risk	
Oxytocin	possible risk	
Paliperidone	possible risk	
Paroxetine	conditional risk	
Pentamidine	known risk	Females > Males
Perflutren lipid microspheres	possible risk	
Pimozide	known risk	Females > Males. Substrate for 3A4
Probucol	known risk	No longer available in U.S.
Procainamide	known risk	
Protriptyline	conditional risk	
Quetiapine	possible risk	Substrate for 3A4
Quinidine	known risk	Females > Males. Substrate for 3A4
Ranolazine	possible risk	
Risperidone	possible risk	
Ritonavir	conditional risk	Substrate for 3A4
Roxithromycin*	possible risk	*not available in the United States
Sertindole	possible risk	
Sertraline	conditional risk	
Solifenacin	conditional risk	
Sotalol	known risk	Females > Males
Sparfloxacin	known risk	
Tacrolimus	possible risk	Substrate for 3A4
Telithromycin	possible risk	Substrate for 3A4

Drug	QT risk[2]	Comment
Terfenadine	known risk	No longer available in U.S.Substrate for 3A4
Thioridazine	known risk	
Tizanidine	possible risk	
Trazodone	conditional risk	Substrate for 3A4
Trimethoprim-Sulfa	conditional risk	
Trimipramine	conditional risk	
Vandetanib	known risk	
Vardenafil	possible risk	Substrate for 3A4
Venlafaxine	possible risk	
Voriconazole	possible risk	
Ziprasidone	possible risk	

[1] Additional agents can be found at https://www.crediblemeds.org
[2] Classification according to the Qtdrugs.org Advisory Board of the Arizona CERT

Appendix 6: Response Evaluation Criteria in Solid Tumors (RECIST), Version 1.1

1.0 MEASURABILITY OF TUMOUR AT BASELINE

1.1 Definitions

At baseline, tumour lesions/lymph nodes will be categorized measurable or non-measurable as follows:

1.1.1 Measurable

Tumour lesions: Must be accurately measured in at least one dimension (*longest* diameter in the plane of measurement is to be recorded) with a *minimum* size of:

- 10 mm by CT scan (CT scan slice thickness no greater than 5 mm);
- 10 mm caliper measurement by clinical examination (lesions which cannot be accurately measured with calipers should be recorded as non-measurable);
- 20 mm by chest X-ray.

Malignant lymph nodes: To be considered pathologically enlarged *and* measurable, a lymph node must be ≥ 15 mm in *short* axis when assessed by CT scan (CT scan slice thickness recommended to be no greater than 5 mm). At baseline and in follow-up, only the *short* axis will be measured and followed.

1.1.2 Non-measurable

All other lesions, including small lesions (longest diameter < 10 mm or pathological lymph nodes with ≥ 10 to < 15 mm short axis) as well as truly non-measurable lesions. Lesions considered truly non-measurable include: leptomeningeal disease, ascites, pleural or pericardial effusion, inflammatory breast disease, lymphangitic involvement of skin or lung, abdominal masses/abdominal organomegaly identified by physical examination that is not measurable by reproducible imaging techniques.

1.1.3 Special Considerations Regarding Lesion Measurability

Bone lesions, cystic lesions, and lesions previously treated with local therapy require particular comment:

Bone lesions:

- Bone scan, PET scan or plain films are not considered adequate imaging techniques to measure bone lesions. However, these techniques can be used to confirm the presence or disappearance of bone lesions.

- Lytic bone lesions or mixed lytic-blastic lesions, with *identifiable soft tissue components*, that can be evaluated by cross sectional imaging techniques such as CT or MRI can be considered measurable lesions if the *soft tissue component* meets the definition of measurability described above.

- Blastic bone lesions are non-measurable.

Cystic lesions:

- Lesions that meet the criteria for radiographically defined simple cysts should not be considered as malignant lesions (neither measurable nor non-measurable) since they are, by definition, simple cysts.

- 'Cystic lesions' thought to represent cystic mestastases can be considered as measurable lesions, if they meet the definition of measurability described above. However, if non-cystic lesions are present in the same patient, these are preferred for selection as target lesions.

Lesions with prior local treatment:

- Tumour lesions situated in a previously irradiated area, or in an area subjected to other loco-regional therapy, are usually not considered measurable unless there has been demonstrated progression in the lesion. Study protocols should detail the conditions under which such lesions would be considered measurable.

1.2 Specifications by Methods of Measurements

1.2.1 Measurement of Lesions

All measurements should be recorded in metric notation, using calipers if clinically assessed. All baseline evaluations should be performed as close as possible to the treatment start and never more than 4 weeks before the beginning of the treatment.

1.2.2 Method of Assessment

The same method of assessment and the same technique should be used to characterize each identified and reported lesion at baseline and during follow-up. Imaging based evaluation should always be done rather than clinical examination unless the lesion(s) being followed cannot be imaged but are assessable by clinical exam.

Clinical lesions: Clinical lesions will only be considered measurable when they are superficial and ≥ 10 mm diameter as assessed using calipers (e.g., skin nodules). For the case of skin lesions, documentation by colour photography including a ruler to estimate the size of the lesion is suggested. As noted above, when lesions can be evaluated by both clinical examination and

imaging, imaging evaluation should be undertaken since it is more objective and may also be reviewed at the end of the study.

Chest X-ray: Chest CT is preferred over chest X-ray, particularly when progression is an important endpoint, since CT is more sensitive than X-ray, particularly in identifying new lesions. However, lesions on chest X-ray may be considered measurable if they are clearly defined and surrounded by aerated lung.

CT, MRI: CT is the best currently available and reproducible method to measure lesions selected for response assessment. This guideline has defined measurability of lesions on CT scan based on the assumption that CT slice thickness is 5 mm or less. When CT scans have slice thickness greater than 5 mm, the minimum size for a measurable lesion should be twice the slice thickness. MRI is also acceptable in certain situations (e.g., for body scans).

Ultrasound: Ultrasound is not useful in assessment of lesion size and should not be used as a method of measurement. Ultrasound examinations cannot be reproduced in their entirety for independent review at a later date and, because they are operator dependent, it cannot be guaranteed that the same technique and measurements will be taken from one assessment to the next. If new lesions are identified by ultrasound in the course of the study, confirmation by CT or MRI is advised. If there is concern about radiation exposure at CT, MRI may be used instead of CT in selected instances.

Endoscopy, laparoscopy: The utilization of these techniques for objective tumour evaluation is not advised. However, they can be useful to confirm complete pathological response when biopsies are obtained or to determine relapse in trials were recurrence following complete response or surgical resection is an endpoint.

Tumour markers: Tumour markers *alone* cannot be used to assess *objective* tumour response. If markers are initially above the upper normal limit, however, they must normalize for a patient to be considered in complete response. Because tumour markers are disease specific, instructions for their measurement should be incorporated into protocols on a disease specific basis.

Cytology, histology: These techniques can be used to differentiate between PR and CR in rare cases if required by protocol (for example, residual lesions in tumour types such as germ cell tumours, where known residual benign tumours can remain).When effusions are known to be a potential adverse effect of treatment (e.g., with certain taxane compounds of angiogenesis inhibitors), the cytological confirmation of the neoplastic origin of any effusion that appears or worsens during treatment can be considered if the measurable tumour has met criteria for response or stable disease in order to differentiate between response (or stable disease) and progressive disease.

2.0 TUMOUR RESPONSE EVALUATION

2.1 Assessment of Overall Tumour Burden and Measurable Disease

To assess objective response or future progression, it is necessary to estimate the *overall tumour burden at baseline* and use this as a comparator for subsequent measurements. Measurable disease is defined by the presence of at least one measurable lesion (as detailed above).

2.2 Baseline Documentation of 'Target' and 'Non-Target' Lesions

When more than one measurable lesion is present at baseline all lesions up to a maximum of five lesions total (and a maximum of two lesions per organ) representative of all involved organs should be identified as *target lesions* and will be recorded and measured at baseline (this means in instances where patients have only one or two organ sites involved, a *maximum* of two and four lesions respectively will be recorded).

Target lesions should be selected on the basis of their size (lesions with the longest diameter), be representative of all involved organs, but in addition should be those that lend themselves to *reproducible repeated measurements*. It may be the case that, on occasion, the largest lesion does not lend itself to reproducible measurement in which circumstance the next largest lesion which can be measured reproducibly should be selected.

Lymph nodes merit special mention since they are normal anatomical structures which may be visible by imaging even if not involved by tumour. As previously noted, pathological nodes which are defined as measurable and may be identified as target lesions must meet the criterion of a short axis of ≥ 15 mm by CT scan. Only the *short* axis of these nodes will contribute to the baseline sum. The short axis of the node is the diameter normally used by radiologists to judge if a node is involved by solid tumour. Nodal size is normally reported as two dimensions in the plane in which the image is obtained (for CT scan this is almost always the axial plane; for MRI the plane of acquisition may be axial, saggital, or coronal). The smaller of these measures is the short axis. For example, an abdominal node which is reported as being 20 mm X 30 mm has a short axis of 20 mm and qualifies as a malignant, measurable node. In this example, 20 mm should be recorded as the node measurement. All other pathological nodes (those with short axis ≥ 10 mm but < 15 mm) should be considered non-target lesions. Nodes that have a short axis < 10 mm are considered non-pathological and should not be recorded or followed.

A *sum of the diameters* (longest for non-nodal lesions, short axis for nodal lesions) for all target lesions will be calculated and reported as the *baseline sum diameters*. If lymph nodes are to be included in the sum, then as previously noted, only the *short* axis is added into the sum. The baseline sum diameters will be used as reference to further characterize any objective tumour regression in the measurable dimension of the disease.

All other lesions (or sites of disease) including pathological lymph nodes should be identified as *non-target lesions* and should also be recorded at baseline. Measurements are not required and

these lesions should be followed as ‘present’, ‘absent’, or in rare cases ‘unequivocal progression’ (more details to follow). In addition, it is possible to record multiple non-target lesions involving the same organ as a single item on the case record form (e.g., ‘multiple enlarged pelvic lymph nodes’ or ‘multiple liver metastases’).

2.3 Response Criteria

This section provides the definitions of the criteria used to determine objective tumour response for target lesions.

2.3.1 Evaluation of Target Lesions

Complete Response (CR): Disappearance of all target lesions. Any pathological lymph nodes (whether target or non-target) must have reduction in short axis to < 10 mm.

Partial Response (PR): At least a 30% decrease in the sum of diameters of target lesions, taking as reference the baseline sum diameters.

Progressive Disease (PD): At least a 20% increase in the sum of diameters of target lesions, taking as reference the *smallest sum on study* (this includes the baseline sum if that is the smallest on study). In addition to the relative increase of 20%, the sum must also demonstrate an absolute increase of at least 5 mm. The appearance of one or more new lesions is also considered progression.

Stable Disease (SD): Neither sufficient shrinkage to qualify for PR nor sufficient increase to qualify for PD, taking as reference the smallest sum diameters while on study.

2.3.2 Special Notes on the Assessment of Target Lesions

Lymph nodes. Lymph nodes identified as target lesions should always have the actual short axis measurement recorded (measured in the same anatomical plane as the baseline examination), even if the nodes regress to below 10 mm on study. This means that when lymph nodes are included as target lesions, the ‘sum’ of lesions may not be zero even if complete response criteria are met, since a normal lymph node is defined as having a short axis of < 10 mm. Case report forms (CRFs) or other data collection methods may therefore be designed to have target nodal lesions recorded in a separate section where, in order to qualify for CR, each node must achieve a short axis < 10 mm. For PR, SD, and PD, the actual short axis measurement of the nodes is to be included in the sum of target lesion.

Target lesions that become ‘too small to measure’. While on study, all lesions (nodal and non-nodal) recorded at baseline should have their actual measurements recorded at each subsequent evaluation, even when very small (e.g., 2 mm). However, sometimes lesions or lymph nodes which are recorded as target lesions at baseline become so faint on CT scan that the radiologist may not feel comfortable assigning an exact measure and may report them as being ‘too small to

measure'. When this occurs it is important that a value be recorded on the CRF. If it is the opinion of the radiologist that the lesion has likely disappeared, the measurement should be recorded as 0 mm. If the lesion is believed to be present and is faintly seen but too small to measure, a default value of 5 mm should be assigned (*Note*: It is less likely that this rule will be used for lymph nodes since they usually have a definable size when normal and are frequently surrounded by fat such as in the retroperitoneum; however, if a lymph node is believed to be present and is faintly seen but too small to measure, a default value of 5 mm should be assigned in this circumstance as well). This default value is derived from the 5 mm CT slice thickness (but should not be changed with varying CT slice thickness). The measurement of these lesions is potentially non-reproducible, therefore providing this default value will prevent false responses or progressions based upon measurement error. If the radiologist is able to provide an actual measurement, that should be recorded, even if below 5 mm.

Lesions that split or coalesce on treatment. When non-nodal lesions 'fragment', the longest diameters of the fragmented portions should be added together to calculate the target lesion sum. Similarly, as lesions coalesce, a plane between them may be maintained that would aid in obtaining maximal diameter measurements of each individual lesion. If the lesions have truly coalesced such that they are no longer separable, the vector of the longest diameter in this instance should be the maximal longest diameter for the 'coalesced lesion'.

2.3.2 Evaluation of Non-Target Lesion

This section provides the definitions of the criteria used to determine the tumour response for the group of non-target lesions. While some non-target lesions may actually be measurable, they need not be measured and instead should be assessed only *qualitatively* at the time points specified in the protocol.

Complete Response (CR): Disappearance of all non-target lesions and normalization of tumour marker level. All lymph nodes must be non-pathological in size (< 10 mm short axis).

Non-CR/Non-PD: Persistence of one or more non-target lesion(s) and/or maintenance of tumour marker level above the normal limits.

Progressive Disease (PD): *Unequivocal progression* of existing non-target lesions. The appearance of one or more new lesions is also considered progression.

2.3.3 Special Notes on Assessment of Progression of Non-Target Disease

The concept of progression of non-target disease requires additional explanations as follows:

When the patient also has measurable disease. In this setting, to achieve 'unequivocal progression' on the basis of the non-target disease, there must be an overall level of substantial worsening in non-target disease such that, even in presence of SD or PR in target disease, the overall tumour burden has increased sufficiently to merit discontinuation of therapy. A modest

'increase' in the size of one or more non-target lesions is usually not sufficient to qualify for unequivocal progression status. The designation of overall progression *solely* on the basis of change in non-target disease in the face of SD or PR of target disease will therefore be extremely rare.

When the patient has only non-measurable disease. This circumstance arises in some phase III trials when it is not a criterion of study entry to have measurable disease. The same general concepts apply here as previously noted, however, in this instance there is no measurable disease assessment to factor into the interpretation of an increase in non-measurable disease burden. Because worsening in non-target disease cannot be easily quantified, a useful test that can be applied when assessing patients for unequivocal progression is to consider if the increase in overall disease burden based on the change in non-measurable disease is comparable in magnitude to the increase that would be required to declare PD for measurable disease: i.e., an increase in tumour burden representing an additional 73% increase in 'volume' (which is equivalent to a 20% increase in diameter in a measurable lesion). Examples include an increase in a pleural effusion from 'trace' to 'large', an increase in lymphangitic disease from localized to widespread, or may be described in protocols as 'sufficient to require a change in therapy'. If 'unequivocal progression' is seen, the patient should be considered to have had overall PD at that point. While it would be ideal to have objective criteria to apply to non-measurable disease, the very nature of that disease makes it impossible to do so, therefore the increase must be substantial.

2.3.4 New Lesions

The appearance of new malignant lesions denotes disease progression. There are no specific criteria for the identification of new radiographic lesions; however, the finding of a new lesion should be unequivocal: i.e., not attributable to differences in scanning technique, change in imaging modality or findings thought to represent something other than tumour. This is particularly important when the patient's baseline lesions show partial or complete response.

A lesion identified on a follow-up study in an anatomical location that was *not* scanned at baseline is considered a new lesion and will indicate disease progression. An example of this is the patient who has visceral disease at baseline and while on study has a CT or MRI brain scan ordered which reveals metastases. The patient's brain metastases are considered to be evidence of PD even if he/she did not have brain imaging at baseline.

If a new lesion is equivocal, for example because of its small size, continued therapy and follow-up evaluation will clarify if it represents truly new disease. If repeat scans confirm there is definitely a new lesion, then progression should be declared using the date of the initial scan.

While FDG-PET response assessments need additional study, it is sometimes reasonable to incorporate the use of FDG-PET scanning to complement CT scanning in assessment of

progression (particularly possible 'new' disease). New lesions on the basis of FDG-PET imagine can be identified according to the following algorithm:

a. Negative FDG-PET at baseline, with a positive FDG-PET at follow-up is a sign of PD based on a new lesion. A 'positive' FDG-PET scan lesion is one which is FDG avid with an uptake greater than twice that of the surrounding tissue on the attenuation corrected image.

b. No FDG-PET at baseline and a positive FDG-PET at follow-up:

If the positive FDG-PET at follow-up corresponds to a new site of disease confirmed by CT, this is PD.

If the positive FDG-PET at follow-up is not confirmed as a new site of disease on CT, additional follow-up CT scans are needed to determine if there is truly progression occurring at that time (if so, the date of PD will be the date of the initial abnormal FDG-PET scan).

If the positive FDG-PET at follow-up corresponds to a pre-existing site of disease on CT that is not progressing on the basis of the anatomic images, this is not PD.

2.4 Evaluation of Best Overall Response

The best overall response is the best response recorded from the start of the study treatment until the end of treatment taking into account any requirement for confirmation. On occasion a response may not be documented until after the end of therapy so protocols should be clear if post-treatment assessments are to be considered in determination of best overall response. Protocols must specify how any new therapy introduced before progression will affect best response designation. The patient's best overall response assignment will depend on the findings of both target and non-target disease and will also take into consideration the appearance of new lesions. Furthermore, depending on the nature of the study and the protocol requirements, it may also require confirmatory measurement. Specifically, in non-randomised trials where response is the primary endpoint, confirmation of PR or CR is needed to deem either one the 'best overall response'.

2.4.1 Time Point Response

It is assumed that at each protocol specified time point, a response assessment occurs. Table 1 provides a summary of the overall response status calculation at each time point for patients who have measurable disease at baseline.

Table 1: Time Point Response: Patients With Target (± Non-Target) Disease

Target lesions	Non-target lesions	New lesions	Overall response
CR	CR	No	**CR**
CR	Non-CR/non-PD	No	**PR**
CR	Not evaluated	No	**PR**
PR	Non-PD or not all evaluated	No	**PR**
SD	Non-PD or not all evaluated	No	**SD**
Not all evaluated	Non-PD	No	**NE**
PD	Any	Yes or No	**PD**
Any	PD	Yes or No	**PD**
Any	Any	Yes	**PD**

CR=complete response, PR=partial response, SD=stable disease, PD=progressive disease, NE=inevaluable

When patients have non-measurable (therefore non-target) disease only, Table 2 is to be used.

Table 2: Time Point Response: Patients With Non-Target Disease Only

Non-target lesions	New lesions	Overall response
CR	No	CR
Non-CR/non-PD	No	Non-CR/non-PD[a]
Not all evaluated	No	NE
Unequivocal PD	Yes or No	PD
Any	Yes	PD

[a] 'Non-CR/non-PD' is preferred over 'stable disease' for non-target disease since SD is increasingly used as endpoint for assessment of efficacy in some trials so to assign this category when no lesions can be measured is not advised.

2.4.2 Missing Assessments and Inevaluable Designation

When no imaging/measurement is done at all at a particular time point, the patient is not evaluable (NE) at that time point. If only a subset of lesion measurements are made at an assessment, usually the case is also considered NE at that time point, unless convincing argument can be made that the contribution of the individual missing lesion(s) would not change the assigned time point response. This would be most likely to happen in the case of PD.

2.4.3 Best Overall Response: All Time Points

The *best overall response* is determined once all the data for the patient is known.

Best response is defined as the best response across all time points (for example, a patient who has SD at first assessment, PR at second assessment, and PD on last assessment has a best overall response of PR). When SD is believed to be the best response, it must also meet the protocol specified minimum time from baseline. If the minimum time is not met when SD is otherwise the best time point response, the patient's best response depends on the subsequent assessments. For example, a patient who has SD at first assessment, PD at second and does not meet minimum duration for SD, will have a best response of PD. The same patient lost to follow-up after the first SD assessment would be considered inevaluable.

2.4.4 Special Notes on Response Assessment

When nodal disease is included in the sum of target lesions and the nodes decrease to 'normal' size (< 10 mm), they may still have a measurement reported on scans. This measurement should be recorded even though the nodes are normal in order not to overstate progression should it be based on the increase in size of the nodes. As noted earlier, this means that patients with CR may not have total sum of 'zero' on the CRF.

Patients with a global deterioration of health status requiring discontinuation of treatment without objective evidence of disease progression at that time should be reported as 'symptomatic deterioration'. Every effort should be made to document objective progression even after discontinuation of treatment. Symptomatic deterioration is *not* a descriptor of an objective response: it is a reason for stopping study therapy. The objective response status of such patients is to be determined by evaluation of target and non-target disease.

Conditions that define 'early progression, early death and inevaluability' are study specific and should be clearly described in each protocol (depending on treatment duration, treatment periodicity).

In some circumstances it may be difficult to distinguish residual disease from normal tissue. When the evaluation of complete response depends upon this determination, it is recommended that the residual lesion be investigated (fine needle aspirate/biopsy) before assigning a status of complete response. FDG-PET may be used to upgrade a response to a CR in a manner similar to

a biopsy in cases where a residual radiographic abnormality is thought to represent fibrosis or scarring. The use of FDG-PET in this circumstance should be prospectively described in the protocol and supported by disease specific medical literature for the indication. However, it must be acknowledged that both approaches may lead to false positive CR due to limitations of FDG-PET and biopsy resolution/sensitivity.

For equivocal findings of progression (e.g., very small and uncertain new lesions; cystic changes or necrosis in existing lesion), treatment may continue until the next scheduled assessment. If at the next scheduled assessment, progression is confirmed, the date of progression should be the earlier date when progression was suspected.

References:

Eisenhauer EA, Therasse P, Bogaerts J, et al. New response evaluation criteria in solid tumours: revised RECIST guideline (version 1.1). Eur J Cancer 2009;45(2):228-47.

Appendix 7: Patient Global Impression of Change

Patients will be asked the following:

Since starting treatment, my colorectal cancer symptoms are:

(1) Very much improved

(2) Much improved

(3) Minimally improved

(4) No change

(5) Minimally worse

(6) Much worse

(7) Very much worse

References:

Mesa AM, Gotlib J, Gupta V, et. al. Effect of ruxolitinib therapy on myelofibrosis-related symptoms and other patient-reported outcomes in COMFORT-1: A randomized, double-blind, placebo-controlled trial. J Clin Oncol. 2013;31:1285-92.

Appendix 8: Recommended Guidelines for the Management of Binimetinib-associated Interstitial Lung Disease

Clinical judgment and experience of the treating physician should guide the management plan of each patient. In general, the following interventions are in addition to the binimetinib-associated interstitial lung disease (ILD) dosing guidelines provided in Table 16 of the protocol.

Drug-associated ILD or pneumonitis is a clinical diagnosis based on clinical signs and symptoms, radiological changes, pulmonary function tests (PFT) and exclusion of other possible etiologies of parenchymal lung disease. The most common symptoms of ILD are nonspecific and include dyspnea, dry cough, fever, fatigue, hypoxia, and occasional hemoptysis. The CTCAE v.4.03 criteria for ILD (pneumonitis) are provided below.

CTCAE v. 4.03 Criteria for Pneumonitis

	**Grade**				
**Adverse Event**	**1**	**2**	**3**	**4**	**5**
Pneumonitis	Asymptomatic; clinical or diagnostic observations only; intervention not indicated	Symptomatic; medical intervention indicated; limiting instrumental ADL	Severe symptoms; limiting self care ADL; oxygen indicated	Life-threatening respiratory compromise; urgent intervention indicated (e.g., tracheotomy or intubation)	Death

Definition: A disorder characterized by inflammation or diffusely affecting the lung parenchyma.

All patients should be instructed to immediately report new or worsening respiratory symptoms. Diagnostic procedures include PFT and high-resolution CT scans. The principal management of ILD consists of drug interruption and/or dose reduction and treatment with steroids as specified below. Empirical antibiotics directed at likely pathogens should also be considered while the results of diagnostic procedures and cultures are pending.

- Prednisolone 40 mg oral, daily
    - Reduce dose by 10 mg every 2 weeks × 2 (until dose reduced to 20 mg oral, daily)
    - Reduce dose by 5 mg weekly × 4 weeks
- Combine with empirical antimicrobial therapy while awaiting results of diagnostic procedures

References:

Shah R. Tyrosine kinase inhibitor-induced interstitial lung disease: Clinical features, diagnostic challenges, and therapeutic dilemmas. Drug Saf 2016;39:1073-1091.

Supplementary Tables

Supplementary Table 1 | Overview of patients in each treatment arm across analysis sets.

Analysis set	Enco+Bini+Cetux	Enco+Cetux	Control	Total
Full analysis set, n (%)	224 (100.0)	220 (100.0)	221 (100.0)	665 (100.0)
Safety set, n (%)	222 (99.1)	216 (98.2)	193 (87.3)	631 (94.9)
Biomarker set, n (%)				
WES, WES, or ctDNA	221 (98.7)	211 (95.9)	189 (85.5)	621 (93.4)
Baseline tumor WES	182 (81.3)	166 (75.5)	155 (70.1)	503 (75.6)
Baseline tumor WTS	163 (72.8)	144 (65.5)	134 (60.6)	441 (66.3)
Baseline ctDNA	196 (87.5)	187 (85.0)	161 (72.9)	544 (81.8)
Paired tumor (WES):ctDNA	157 (70.1)	143 (65.0)	128 (57.9)	428 (64.4)
Paired ctDNA (Baseline:EoT)	112 (50.0)	113 (51.4)	95 (43.0)	320 (48.1)

Percentages are calculated based on the number of patients in the full analysis set as the denominator. Bini, binimetinib; Cetux, cetuximab; ctDNA, circulating tumor DNA; Enco, encorafenib; EoT, end of treatment; WES, whole exome sequencing; WTS, whole transcriptome sequencing.

Supplementary Table 2 | Baseline BRAF V600E, MSI, and TMB characteristics in BEACON CRC.

	WES				ctDNA			
	**Enco+ Bini+ Cetux (n=182)**	**Enco +Cetux (n=166)**	**Control (n=155)**	**Total (N=503)**	**Enco +Bini+ Cetux (n=196)**	**Enco +Cetux (n=187)**	**Control (n=161)**	**Total (N=544)**
***BRAF* V600E status**								
Detected, n (%)	174 (95.6)	154 (92.8)	148 (95.5)	476 (94.6)	177 (90.3)	169 (90.4)	146 (90.7)	492 (90.4)
Not detected[a], n (%)	8 (4.4)	12 (7.2)	7 (4.5)	27 (5.4)	19 (9.7)	18 (9.6)	15 (9.3)	52 (9.6)
**MSI status**								
MSI-H, n (%)	23 (12.6)	20 (12.0)	13 (8.4)	56 (11.1)	23 (11.7)	16 (8.6)	9 (5.6)	48 (8.8)
**TMB**								
Median (range)	7.630 (5.74–42.00)	7.780 (5.45–30.27)	7.780 (5.55–30.90)	7.740 (5.45–42.00)	11.480 (0.96–545.05)	11.480 (0.00–288.36)	11.480 (0.96–229.20)	11.480 (0.00–545.05)
TMB ≥ 10 mutations/Mb, n (%)	33 (18.1)	22 (13.3)	24 (15.5)	79 (15.7)	-	-	-	-

[a]The “not detected” group in the WES subset included patients who either did not have results found for this analyte or had *BRAF* mutations other than V600E; in the ctDNA subset, this group included patients with no ctDNA reported. Bini, binimetinib; Cetux, cetuximab; ctDNA, circulating tumor DNA; Enco, encorafenib; MSI, microsatellite instability; MSI-H, microsatellite instability-high; TMB, tumor mutational burden; WES, whole exome sequencing.

Supplementary Table 3 | Concordance between tumor (WES) and ctDNA in baseline BRAF V600E status.

**WES vs ctDNA**		**Tumor (WES)**	
		Detected, n (%)	Not detected, n (%)
**ctDNA**	Detected, n (%)	368 (91.1)	0
	Not detected, n (%)	36 (8.9)	4 (100.0)

Percentages are based on the total number of patients with paired tumor and ctDNA samples and are calculated using the number of patients with tumor samples in each column as the denominator. ctDNA, circulating tumor DNA; WES, whole exome sequencing.

Supplementary Table 4 | Association between OS with Enco+Cetux±Bini treatment and baseline RNF43 mutational status in the MSS subgroup.

***RNF43* mutational status**	**Unadjusted analysis**				**Adjusted analysis**			
	**No. of events/ no. of patients (%)**		**HR (95% CI)**	**P value[a]**	**No. of events/ no. of patients (%)**		**HR (95% CI)**	**P value[a]**
	**Enco+ Cetux**	**Control**	**Enco+Cetux vs Control**		**Enco+ Cetux**	**Control**	**Enco+Cetux vs Control**	
**Overall**								
Wild type	63/114 (55.3)	87/120 (72.5)	0.53 (0.381, 0.733)	0.0693	62/113 (54.9)	85/117 (72.6)	0.55 (0.396, 0.772)	0.3518
Mutant	32/52 (61.5)	21/35 (60.0)	0.93 (0.535, 1.622)		32/52 (61.5)	21/35 (60.0)	0.71 (0.386, 1.322)	
**MSS subgroup[b]**								
Wild type	53/96 (55.2)	72/95 (75.8)	0.47 (0.329, 0.675)	0.0571	52/95 (54.7)	71/94 (75.5)	0.57 (0.392, 0.825)	0.4662
Mutant	20/32 (62.5)	13/23 (56.5)	0.99 (0.487, 2.009)		20/32 (62.5)	13/23 (56.5)	0.49 (0.204, 1.163)	

RNF43 mutational status	Unadjusted analysis				Adjusted analysis			
	No. of events/no. of patients (%)		HR (95% CI)	P value[a]	No. of events/no. of patients (%)		HR (95% CI)	P value[a]
	Enco+Bini+Cetux	Control	Enco+Bini+Cetux vs Control		Enco+Bini+Cetux	Control	Enco+Bini+Cetux vs Control	
**Overall**								
Wild type	82/136 (60.3)	87/120 (72.5)	0.55 (0.405, 0.745)	0.6269	78/130 (60.0)	85/117 (72.6)	0.51 (0.372, 0.697)	0.7447
Mutant	25/46 (54.3)	21/35 (60.0)	0.71 (0.394, 1.263)		25/46 (54.3)	21/35 (60.0)	0.44 (0.226, 0.870)	
**MSS subgroup[b]**								
Wild type	66/111 (59.5)	72/95 (75.8)	0.45 (0.323, 0.638)	0.8265	62/106 (58.5)	71/94 (75.5)	0.45 (0.315, 0.640)	0.3624
Mutant	10/23 (43.5)	13/23 (56.5)	0.55 (0.238, 1.247)		10/23 (43.5)	13/23 (56.5)	0.18 (0.058, 0.572)	

[a]P value (two-sided Wald test) for the mutation-by-treatment interaction based on Cox model with no adjustment of baseline covariates (unadjusted analysis) and with adjustment for baseline covariates (adjusted analysis: ECOG performance status, C-reactive protein, number of organs, tumor status, cetuximab source, and prior use of irinotecan at randomization). [b]In patients with MSS only. Cetux, cetuximab; CI, confidence interval; ECOG, Eastern Cooperative Oncology Group; Enco, encorafenib; HR, hazard ratio; MSS, microsatellite stability.

Supplementary Table 5 | Data for Kaplan–Meier plots of OS by cytolytic score (split on median) in each treatment arm.

	**Median OS (95% CI), months**	**Unadjusted**		**Adjusted**	
		**HR (95%) > median vs ≤ median**	**$P_{interaction}$**	**HR (95%) > median vs ≤ median**	**$P_{interaction}$**
Enco+Bini+Cetux: ≤ median	7.458 (6.407–9.823)	-	0.0003	-	0.0035
Enco+Bini+Cetux: > median	11.99 (8.378–16.26)	0.56 (0.377–0.844)		0.48 (0.310–0.740)	
Enco+Cetux: ≤ median	14.16 (8.772–17.68)	-		-	
Enco+Cetux: > median	7.261 (5.027–9.298)	1.85 (1.203–2.847)		1.46 (0.909–2.360)	
Control: ≤ median	5.191 (3.943–6.867)	-		-	
Control: > median	6.834 (5.421–9.068)	0.77 (0.511–1.158)		0.64 (0.415–0.988)	

P values for interaction (two-sided Wald test) were based on Cox proportional hazard models with and without adjustment for baseline covariates. CI, confidence interval; HR, hazard ratio; OS, overall survival.

Supplementary Table 6 | Summary of key acquired alterations in paired samples.

**Acquired alteration**	**Enco+Bini+Cetux**<br>**n/N (%)**	**Enco+Cetux**<br>**n/N (%)**	**Control**<br>**n/N (%)**	**Total**<br>**n/N (%)**
BRAF amplification	6/112 (5.4)	2/111 (1.8)	1/94 (1.1)	9/317 (2.8)
IGF1R amplification	5/112 (4.5)	1/109 (0.9)	0/93 (0)	6/314 (1.9)
KRAS amplification	10/112 (8.9)	5/112 (4.5)	0/94 (0)	15/318 (4.7)
ALK fusion	4/112 (3.6)	3/112 (2.7)	0/94 (0)	7/318 (2.2)
BRAF fusion	5/112 (4.5)	6/112 (5.4)	0/94 (0)	11/318 (3.5)
MET fusion	4/112 (3.6)	9/112 (8.0)	0/94 (0)	13/318 (4.1)
EGFR mutation	8/104 (7.7)	4/102 (3.9)	0/87 (0)	12/293 (4.1)

Bini, binimetinib; Cetux, cetuximab; Enco, encorafenib.

Supplementary Table 7 | Frequency of top acquired resistance alterations.

**Acquired gene alteration**	**Treatment arm**	**Frequency n/N (%)**	**P Value (Enco+Bini+Cetux vs Enco+Cetux)**
KRAS mutant	Enco+Bini+Cetux	45/106 (42.5)	0.7844
	Enco+Cetux	50/111 (45.0)	
	Control	0/91 (0)	
NRAS mutant	Enco+Bini+Cetux	28/110 (25.5)	0.0808
	Enco+Cetux	41/110 (37.3)	
	Control	3/93 (3.2)	
MAP2K1 mutant	Enco+Bini+Cetux	4/109 (3.7)	0.0028
	Enco+Cetux	18/112 (16.1)	
	Control	3/92 (3.3)	
MET amplification	Enco+Bini+Cetux	22/112 (19.6)	0.7300
	Enco+Cetux	19/112 (17.0)	
	Control	0/94 (0)	
Any top acquired	Enco+Bini+Cetux	63/108 (58.3)	0.6784
	Enco+Cetux	68/110 (61.8)	
	Control	6/88 (6.8)	

P value was calculated based on the Fisher exact test (two-sided). Amp, amplification; Bini, binimetinib; Cetux, cetuximab; ctDNA, circulating tumor DNA; Enco, encorafenib; mut, mutation.

Supplementary Table 8 | Summary of MAP2K1 mutations and RAF dependency.

	Enco+Bini+Cetux (n = 4)	Enco+Cetux (n = 28)	Control (n = 3)	Total (N = 35)
**RAF dependency, n**				
**Dependent**	2	18[a]	0	20
**Independent**	0	0	1	1
**Unknown**	2	10	2	14
***MAP2K1* mutations, n (%)[b]**<br>RAF Dependency<br>Dependent Independent Unknown				
**C121S[c]**	0	2 (7.1)	0	2 (5.7)
**E102_I103del[c]**	0	0	1 (33.3)	1 (2.9)
**E203K[c]**	0	2 (7.1)	0	2 (5.7)
**E41K**	1 (25.0)	0	0	1 (2.9)
**F53L[c]**	2 (50.0)	6 (21.4)	0	8 (22.9)
**F53V[c]**	0	1 (3.6)	0	1 (2.9)
**G128D[c]**	0	1 (3.6)	0	1 (2.9)
**G128V[c]**	0	1 (3.6)	0	1 (2.9)
**G380V**	0	1 (3.6)	0	1 (2.9)

**I204T**	0	0	1 (33.3)	1 (2.9)
**K205T**	1 (25.0)	0	0	1 (2.9)
**K57E[c]**	0	1 (3.6)	0	1 (2.9)
**K57N[c]**	0	6 (21.4)	0	6 (17.1)
**K57T[c]**	0	2 (7.1)	0	2 (5.7)
**L42F**	0	1 (3.6)	0	1 (2.9)
**P105_A106del**	0	1 (3.6)	0	1 (2.9)
**P265R**	0	1 (3.6)	0	1 (2.9)
**Q56P[c]**	0	1 (3.6)	0	1 (2.9)
**T378del**	0	0	1 (33.3)	1 (2.9)
**W374C**	0	1 (3.6)	0	1 (2.9)

[a]Five patients treated with Enco+Cetux had multiple *MAP2K1* mutations. [b]*MAP2K1* mutations are classified as previously published.[56] [c]*MAP2K1* mutations associated with ERK phosphorylation (p-ERK) as previously reported.[57] bini, binimetinib; cetux, cetuximab; del, deletion; enco, encorafenib.

Supplementary Figures

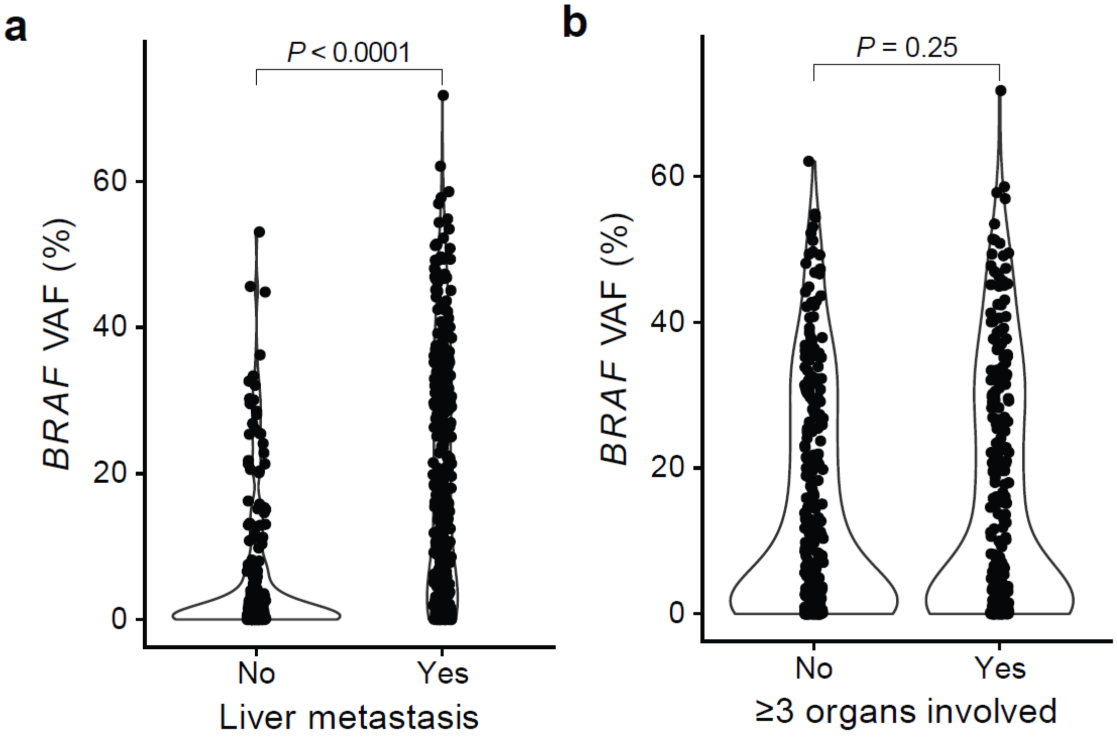

Supplementary Fig. 1 | ctDNA BRAF V600E VAF by disease characteristics at baseline.

a, ctDNA *BRAF* V600E VAF by the presence of liver metastasis (yes vs no). **b,** ctDNA *BRAF* V600E VAF by the presence of ≥ 3 involved organs (yes vs no). P values are based on two-sample t-tests (two-sided).

a

CRC subtypes	No. of Events/No. of Patients (%) Enco+Cetux	Control	HR (95% CI)	*P* value
**BM subtypes**				0.3038
BM1	31/49 (63.3)	26/40 (65.0)	0.72 (0.420, 1.260)	
BM2	55/94 (58.5)	66/91 (72.5)	0.53 (0.370, 0.770)	
**CMS***				0.0718
CMS1	45/73 (61.6)	43/59 (72.9)	0.46 (0.300, 0.720)	
CMS3	9/13 (69.2)	14/17 (82.4)	1.47 (0.460, 4.740)	
CMS4	32/54 (59.3)	32/50 (64.0)	0.94 (0.560, 1.600)	

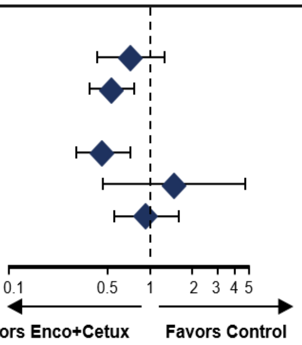

CRC subtypes	Enco+Bini+Cetux	Control	HR (95% CI)	*P* value
**BM subtypes**				0.7151
BM1	28/55 (50.9)	26/40 (65.0)	0.53 (0.300, 0.940)	
BM2	67/103 (65.0)	66/91 (72.5)	0.48 (0.330, 0.690)	
**CMS***				0.7485
CMS1	48/76 (63.2)	43/59 (72.9)	0.39 (0.250, 0.610)	
CMS3	14/18 (77.8)	14/17 (82.4)	0.35 (0.120, 1.060)	
CMS4	30/59 (50.8)	32/50 (64.0)	0.49 (0.290, 0.820)	

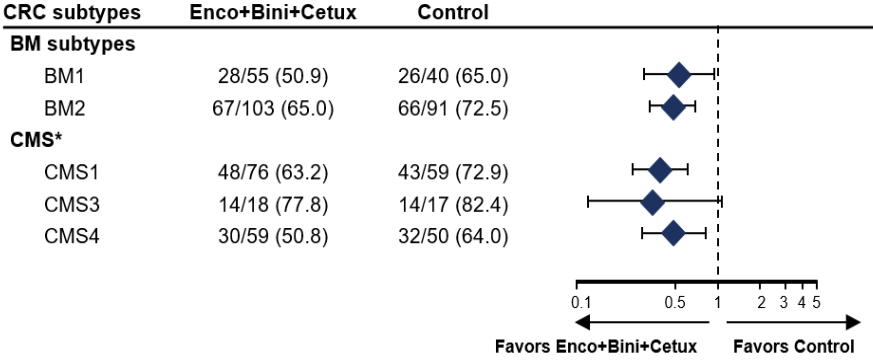

b

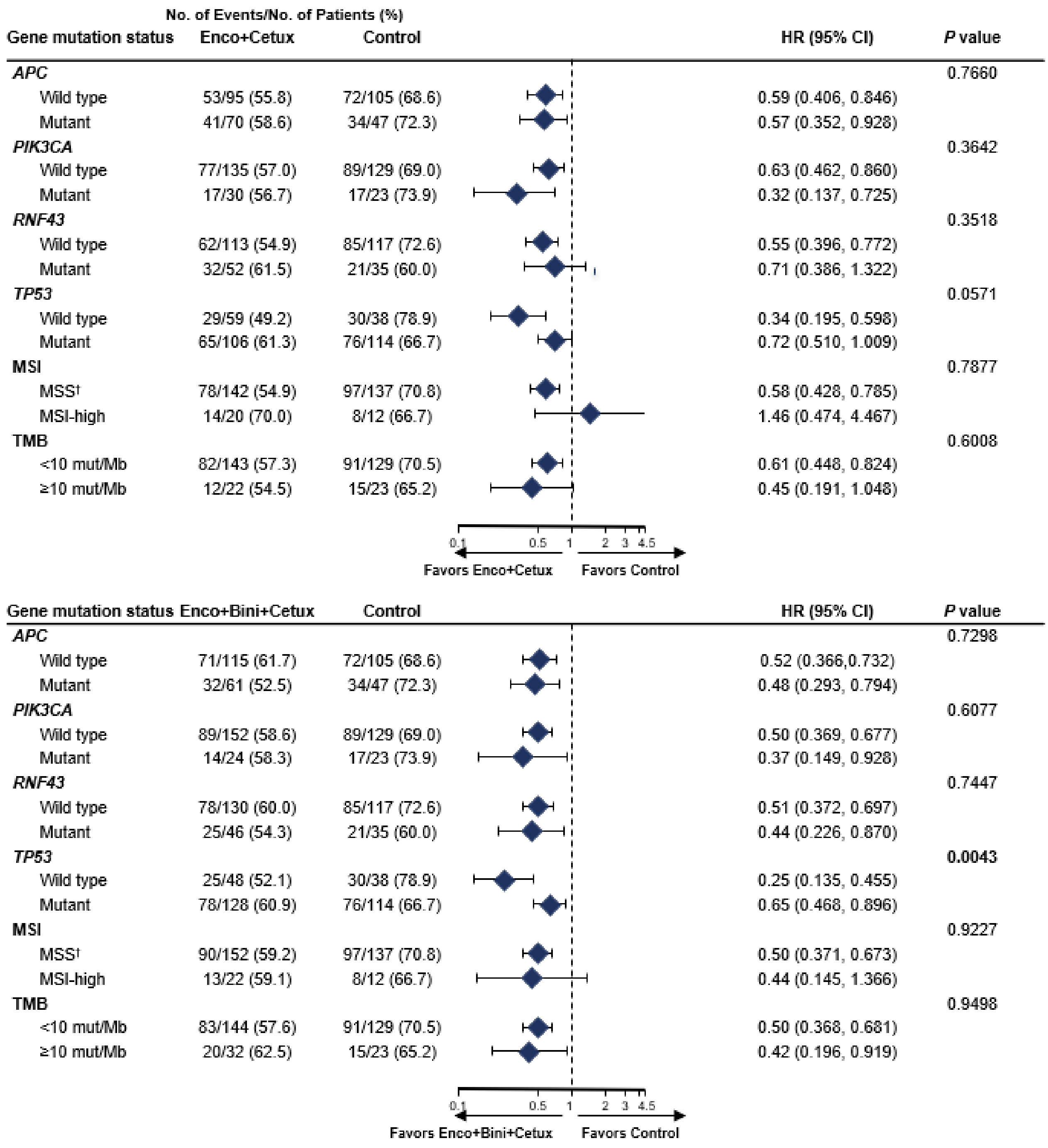

Supplementary Fig. 2 | Association between OS and CRC subtypes or baseline mutational status (adjusted for covariates).

a, Forest plots of HRs for OS with Enco+Cetux (top panel) or Enco+Bini+Cetux (bottom panel) vs control in subgroups defined by BM subtypes and CMS. **b,** Forest plots of HRs for OS with

Enco+Cetux (top panel) or Enco+Bini+Cetux (bottom panel) vs control in subgroups defined by specific gene mutations, MSI status, or TMB levels. The diamonds show HRs and the lines show 95% CI. P values for interaction (two-sided Wald test) were based on the Cox model with adjustment for baseline covariates (ECOG performance status, C-reactive protein, number of organs, tumor status, cetuximab source, and prior use of irinotecan at randomization). A two-sided Wald test was used for P value of interaction terms. *The CMS2 subgroup was excluded due to small sample size. †The MSS subgroup included patients with MSI-low; those with inconclusive results were excluded from the analysis. BM, *BRAF* mutant; Bini, binimetinib; Cetux, cetuximab; CI, confidence interval; CMS, consensus molecular subtypes; CRC, colorectal cancer; ECOG, Eastern Cooperative Oncology Group; Enco, encorafenib; HR, hazard ratio; MSI, microsatellite instability; MSI-H, microsatellite instability-high; MSS, microsatellite stability; mut, mutation; OS, overall survival; TMB, tumor mutational burden.

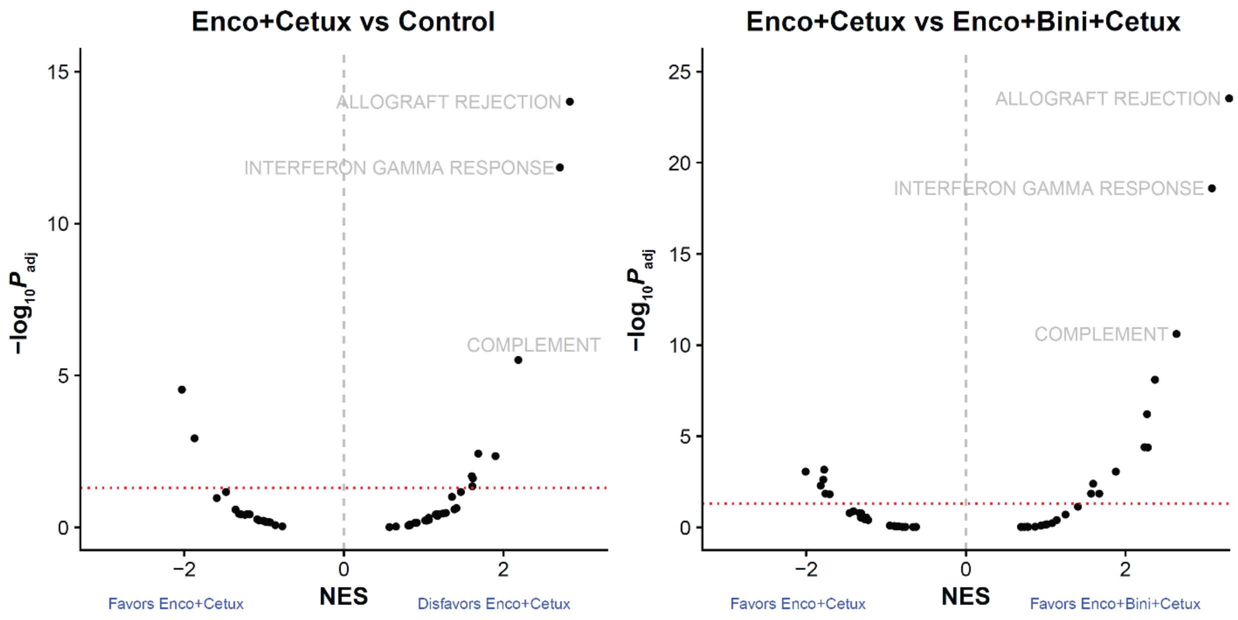

Supplementary Fig. 3 | Association between immune gene signatures and OS benefit in the Enco+Bini+Cetux and Enco+Cetux arms.

Associations between OS and hallmark gene signatures, based on gene set enrichment analysis of treatment arm by gene expression interaction *z* scores from Cox proportional models using scaled gene expression. Gene sets showing the highest NES based on interaction between the Enco+Bini+Cetux or control arm vs the Enco+Cetux arm are labeled; Benjamini–Hochberg-adjusted P values (two-sided) are based on permutation tests. Bini, binimetinib; BM, *BRAF* mutant; Cetux, cetuximab; Enco, encorafenib; NES, normalized enrichment; IQR, interquartile range; OS, overall survival; TPM, transcripts per million.

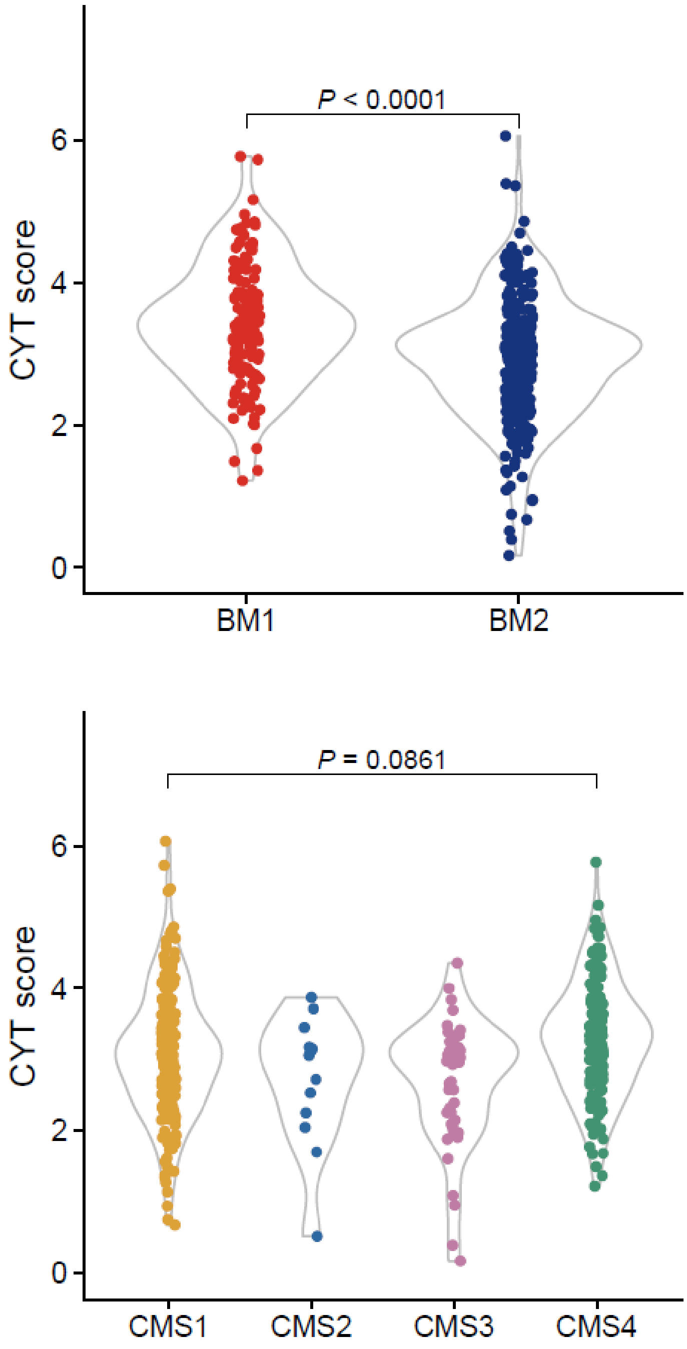

Supplementary Fig. 4 | Cytolytic scores in CRC subtypes.

Violin plots of cytolytic score by BM subtypes and CMS. Top panel: P value from two-sample t-test (two-sided). Bottom panel: P value for the comparison between CMS1 and CMS4 is based on one-way ANOVA (2-sided) with Tukey's honest significant difference test. BM, BRAF mutant; CMS, consensus molecular subtypes; CRC, colorectal cancer; CYT, cytolytic.

a

Acquired gene alteration	No. of Events/No. of Patients (%) Acquired	Not acquired	HR (95% CI)
***KRAS* mutation**			
OS	60/95 (63.2)	69/122 (56.6)	1.49 (1.045, 2.133)
PFS	79/95 (83.2)	90/122 (73.8)	1.45 (1.058, 1.976)
***NRAS* mutation**			
OS	45/69 (65.2)	85/151 (56.3)	1.50 (1.038, 2.156)
PFS	64/69 (92.8)	109/151 (72.2)	1.80 (1.310, 2.464)
***MAP2K1* mutation**			
OS	17/22 (77.3)	116/199 (58.3)	1.91 (1.142, 3.184)
PFS	18/22 (81.8)	156/199 (78.4)	1.71 (1.044, 2.806)
***MET* amplification**			
OS	33/41 (80.5)	101/183 (55.2)	2.43 (1.616, 3.650)
PFS	39/41 (95.1)	137/183 (74.9)	2.28 (1.560, 3.321)
**Any top acquired**			
OS	81/131 (61.8)	49/87 (56.3)	1.64 (1.134, 2.365)
PFS	111/131 (84.7)	60/87 (69.0)	1.75 (1.258, 2.435)

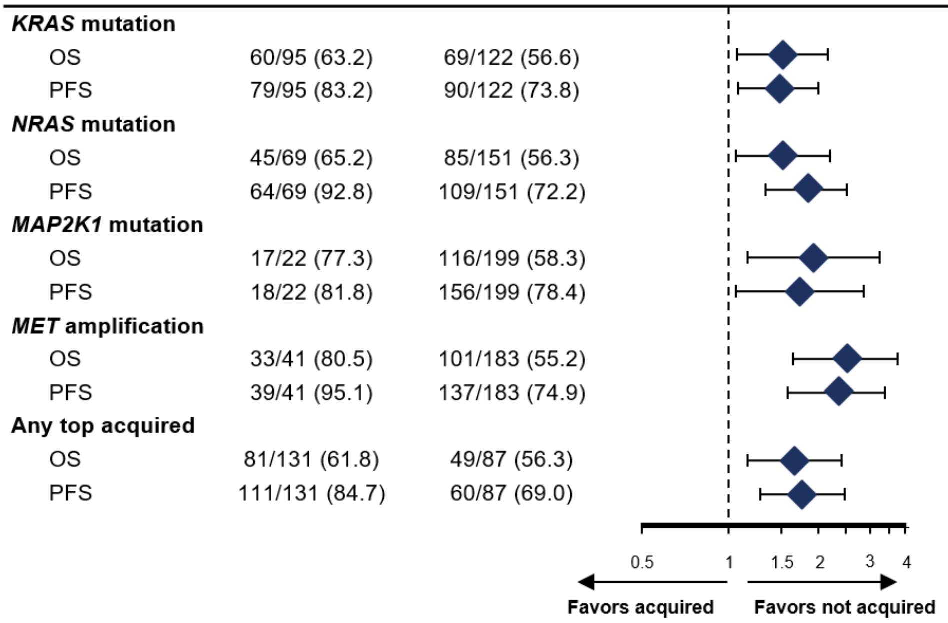

b

Acquired gene alteration	No. of Events/No. of Patients (%) Acquired	Not acquired	HR (95% CI)
***KRAS* mutation**			
OS	59/94 (62.8)	68/121 (56.2)	1.51 (1.040, 2.190)
PFS	78/94 (83.0)	89/121 (73.6)	1.42 (1.030, 1.950)
***NRAS* mutation**			
OS	45/69 (65.2)	83/149 (55.7)	1.23 (0.840, 1.810)
PFS	64/69 (92.8)	107/149 (71.8)	1.76 (1.260, 2.450)
***MAP2K1* mutation**			
OS	17/22 (77.3)	114/197 (57.9)	1.67 (0.970, 2.870)
PFS	18/22 (81.8)	154/197 (78.2)	1.73 (1.040, 2.880)
***MET* amplification**			
OS	32/40 (80.0)	100/182 (54.9)	2.11 (1.370, 3.250)
PFS	38/40 (95.0)	136/182 (74.7)	1.95 (1.300, 2.920)
**Any top acquired**			
OS	80/130 (61.5)	48/86 (55.8)	1.51 (1.020, 2.230)
PFS	110/130 (84.6)	59/86 (68.6)	1.67 (1.190, 2.350)

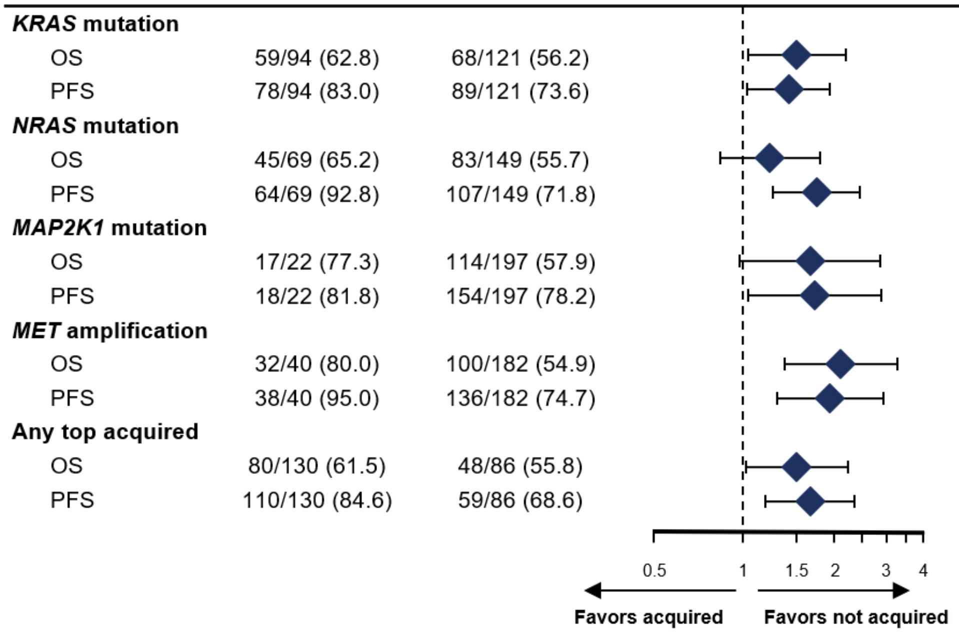

Supplementary Fig. 5* | *Correlation of top acquired resistance alterations with survival.

Forest plots of HRs for OS and PFS by presence of top acquired alterations. The diamonds show HRs and the lines show 95% CI. Cox proportional hazard models **a,** without and **b,** with adjustment for baseline covariates [ECOG performance status, C-reactive protein, number of organs, tumor status, cetuximab source, and prior use of irinotecan at randomization]) were used. For each acquired alteration type, only patients who did not have the specific alteration at baseline and were either in the Enco+Cetux or Enco+Cetux+ Bini arms were analyzed. Acq, acquired; amp, amplification; Bini, binimetinib; Cetux, cetuximab; CI, confidence interval; ECOG, Eastern Cooperative Oncology Group; Enco, encorafenib; HR, hazard ratio; mut, mutation; OS, overall survival; PFS, progression-free survival.

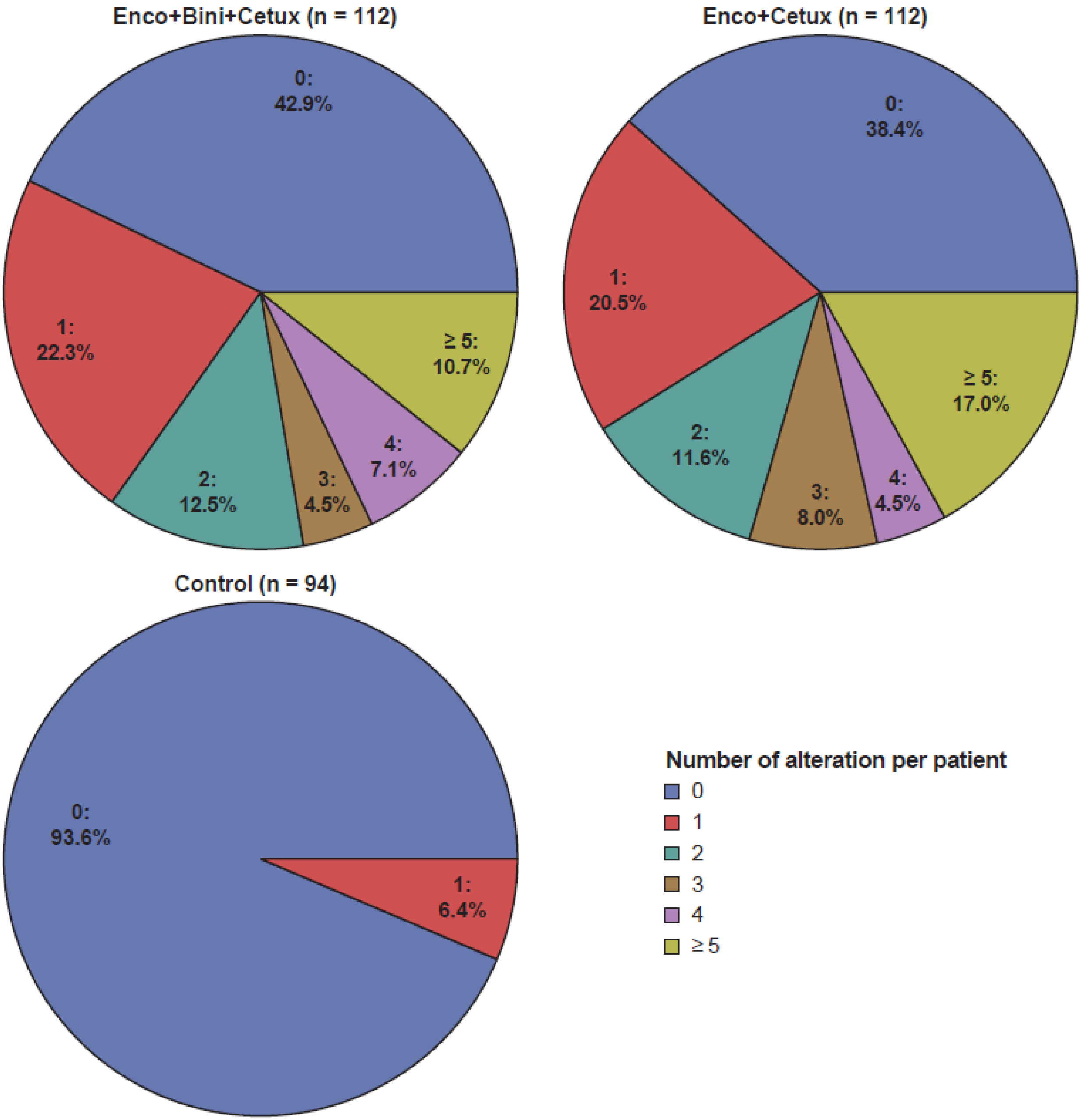

Supplementary Fig. 6 | Distribution of the proportions of patients with different numbers of acquired alterations in each treatment arm.

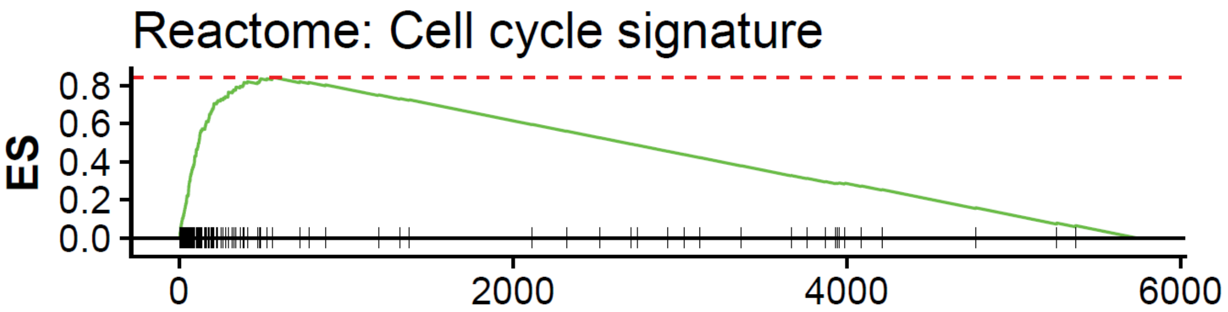

Supplementary Fig. 7 | Enrichment plots from the GSEA against hallmark cell cycle signatures associated with acquiring ≥ 1 top alteration at EoT.

EoT, end of treatment; ES, enrichment score; GSEA, gene set enrichment analysis.

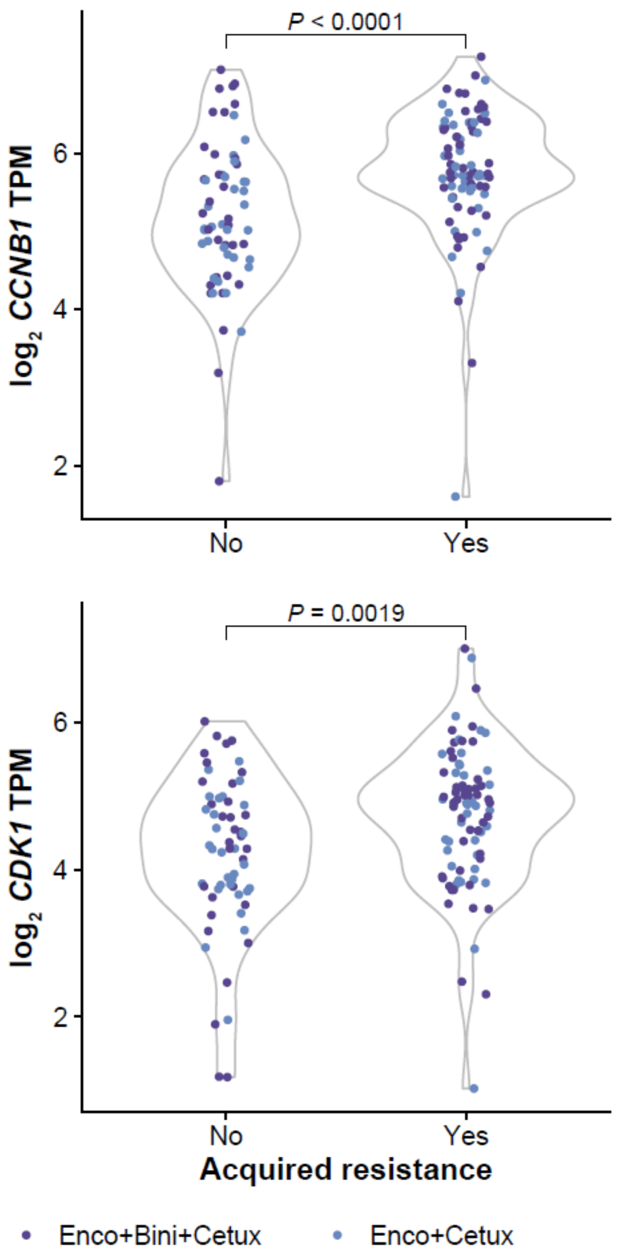

Supplementary Fig. 8 | CCNB1 and CDK1 expression by acquired resistance status in patients treated with Enco+Cetux±Bini.

Violin plots showing *CCNB1* and *CDK1* expression in patients who acquired a top gene alteration vs those who did not. P values from two-sample t-test (2-sided). Data points are color coded by treatment arm. Bini, binimetinib; Cetux, cetuximab; Enco, encorafenib; TPM, transcripts per million.

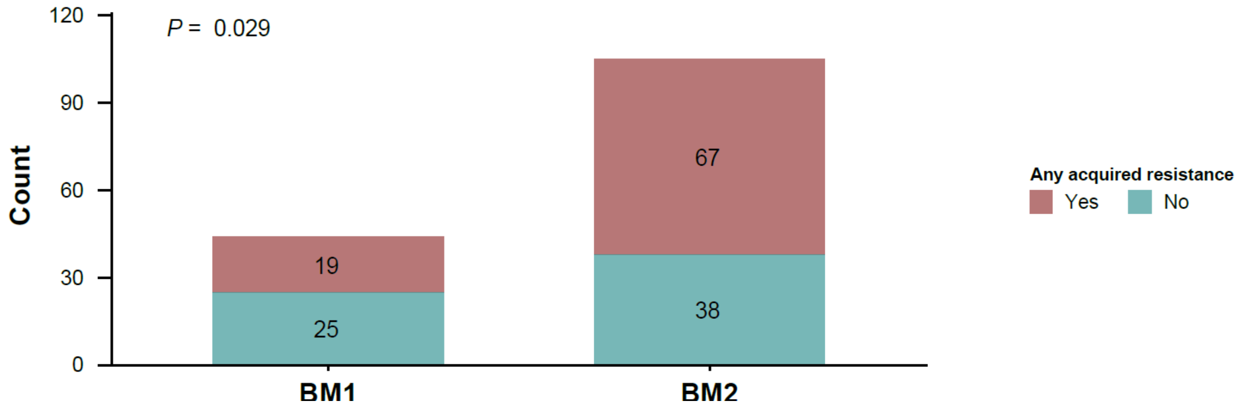

Supplementary Fig. 9 | Detection of acquired alterations across BM1 and BM2 subtypes.

P value is based on the Fisher's exact test (2-sided). BM, *BRAF* mutant.

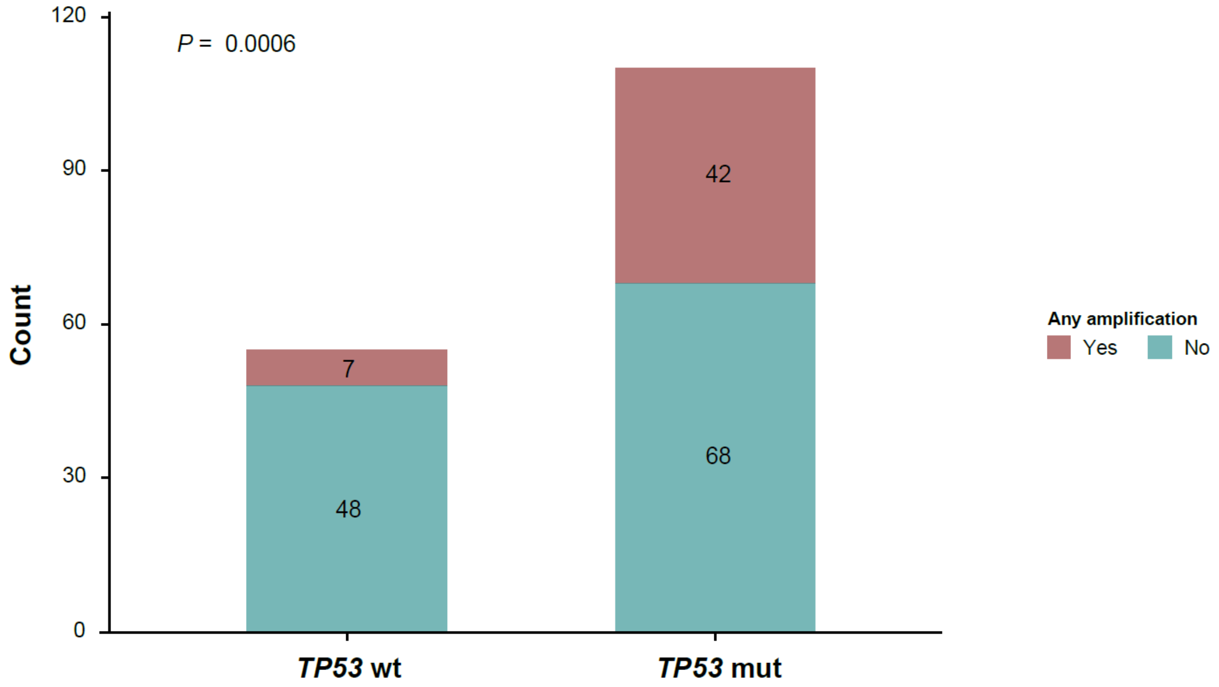

Supplementary Fig. 10 | Association between baseline TP53 mutation status and any acquired putative resistance amplification (i.e. MET, KRAS, BRAF, IGF1R)

P value is based on Fisher's exact test (2-sided). Mut, mutation; wt, wild-type.

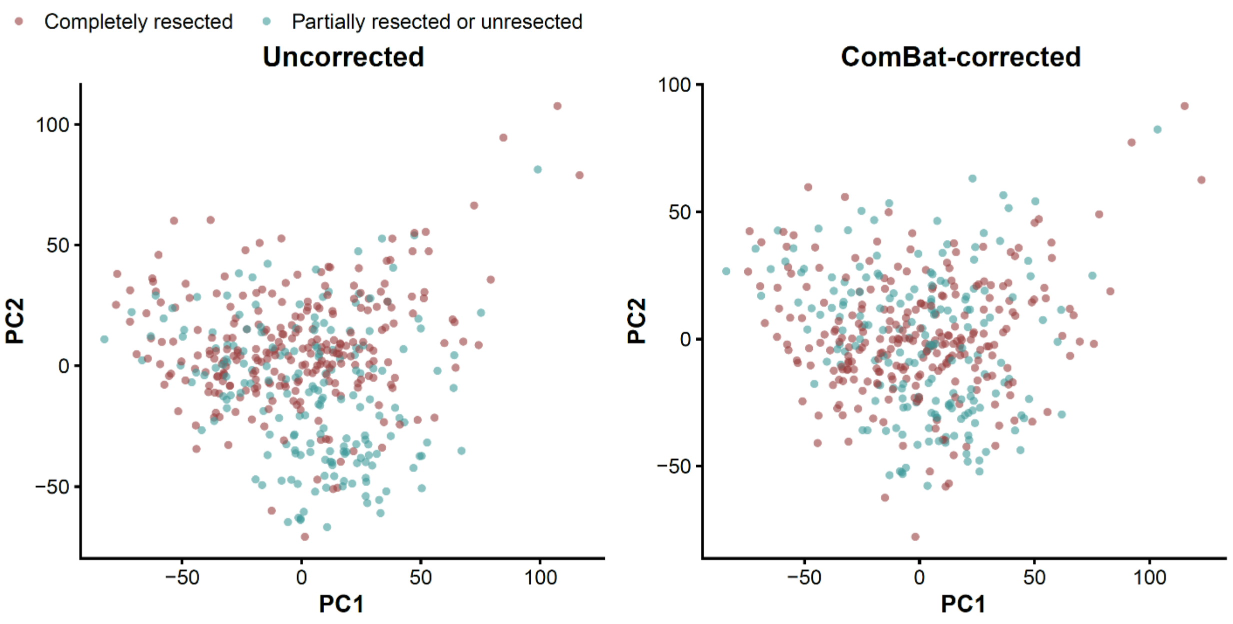

Supplementary Fig. 11 | Principal component analysis for batch effect in whole transcriptome sequencing for patients with completely resected vs partially resected or unresected primary tumors.

PC, principal component.
