# Supplementary material for: Molecular profiling of BRAF-V600E-mutant metastatic colorectal cancer in the phase 3 BEACON CRC trial
Source: Nat Med. 2024 Sep 23;30(11):3261–71. doi: 10.1038/s41591-024-03235-9 (PMC11564101; doi:10.1038/s41591-024-03235-9)
Supplement: Supplementary file 2 — Reporting Summary [file 41591_2024_3235_MOESM2_ESM.pdf]

nature portfolio

Corresponding author(s): Scott Kopetz

Last updated by author(s): Jul 26, 2024

Reporting Summary

Nature Portfolio wishes to improve the reproducibility of the work that we publish. This form provides structure for consistency and transparency in reporting. For further information on Nature Portfolio policies, see our Editorial Policies and the Editorial Policy Checklist.

Statistics

For all statistical analyses, confirm that the following items are present in the figure legend, table legend, main text, or Methods section.

n/a	Confirmed	
☐	☒	The exact sample size (*n*) for each experimental group/condition, given as a discrete number and unit of measurement
☒	☐	A statement on whether measurements were taken from distinct samples or whether the same sample was measured repeatedly
☐	☒	The statistical test(s) used AND whether they are one- or two-sided<br>*Only common tests should be described solely by name; describe more complex techniques in the Methods section.*
☐	☒	A description of all covariates tested
☐	☒	A description of any assumptions or corrections, such as tests of normality and adjustment for multiple comparisons
☐	☒	A full description of the statistical parameters including central tendency (e.g. means) or other basic estimates (e.g. regression coefficient) AND variation (e.g. standard deviation) or associated estimates of uncertainty (e.g. confidence intervals)
☐	☒	For null hypothesis testing, the test statistic (e.g. *F*, *t*, *r*) with confidence intervals, effect sizes, degrees of freedom and *P* value noted<br>*Give P values as exact values whenever suitable.*
☒	☐	For Bayesian analysis, information on the choice of priors and Markov chain Monte Carlo settings
☒	☐	For hierarchical and complex designs, identification of the appropriate level for tests and full reporting of outcomes
☐	☒	Estimates of effect sizes (e.g. Cohen's *d*, Pearson's *r*), indicating how they were calculated

Our web collection on statistics for biologists contains articles on many of the points above.

Software and code

Policy information about availability of computer code

Data collection	ComBat in the SVA R package, version 3.50.0; CMSclassifier package, version 1.0.0; AnnotationDbi: R package, version 1.62.2; fgsea package, version 1.28.0 (Bioconductor); MSigDB R package, version 6.2.1; xCell, version 1.1.0; MCPcounter, version 1.2.0; SigProfilerExtractor, version 1.1
Data analysis	Statistical analyses were done using SAS version 9.4 or R version 4.0 and above.

For manuscripts utilizing custom algorithms or software that are central to the research but not yet described in published literature, software must be made available to editors and reviewers. We strongly encourage code deposition in a community repository (e.g. GitHub). See the Nature Portfolio guidelines for submitting code & software for further information.

Data

Policy information about availability of data

All manuscripts must include a data availability statement. This statement should provide the following information, where applicable:

- Accession codes, unique identifiers, or web links for publicly available datasets
- A description of any restrictions on data availability
- For clinical datasets or third party data, please ensure that the statement adheres to our policy

The analyses in this paper were based on a data cutoff of August 15, 2019.
Upon request, and subject to review, Pfizer will provide the data that support the findings of this study. Subject to certain criteria, conditions, and exceptions, Pfizer

may also provide access to the related individual de-identified participant data. Pfizer will also consider requests for the protocol, data dictionary, and statistical analysis plan. See https://www.pfizer.com/science/clinical-trials/trial-data-and-results for more information.
Data may be requested from Pfizer trials 24 months after study completion. The BEACON CRC study has been completed on November 2022. The de-identified participant data will be made available to researchers whose proposals meet the research criteria and other conditions, and for which an exception does not apply, via a secure portal. To gain access, data requestors must enter into a data access agreement with Pfizer.

Research involving human participants, their data, or biological material

Policy information about studies with human participants or human data. See also policy information about sex, gender (identity/presentation), and sexual orientation and race, ethnicity and racism.

Reporting on sex and gender	Sex and gender are described in the primary publication of the BEACON CRC study (DOI: 10.1056/NEJMoa1908075). We did not take sex into account in this study as it is not a relevant issue in this analysis - subgroup results reported in the primary publication did not show any difference in outcomes based on sex.
Reporting on race, ethnicity, or other socially relevant groupings	N/A, we did not take race, ethnicity, or other socially relevant groupings into account in this study as it is not a relevant issue in this analysis.
Population characteristics	Patients with histologically or cytologically confirmed, metastatic CRC with the BRAF V600E mutation who had disease progression after 1 or 2 previous treatment regimens were enrolled. Baseline characteristic are presented in the primary publication of the BEACON CRC study (DOI: 10.1056/NEJMoa1908075). In the primary publication of the BEACON CRC study, the median age of the patients was 61-62 years across the treatment arms; ECOG PS was 0 and 1 in 48% and 52% of patients, respectively (one patient had ECOG PS of 2); 47% had ≥ 3 organs involved; 57% had their primary tumor completely resected; 61% had liver metastases; 43% had baseline CRP > 10 mg/L; 62% and 37% had 1 and 2 prior lines of therapy, respectively (2 patients received > 2 prior lines); and 8% had high microsatellite instability.
Recruitment	Participants were recruited by investigators at each participating study site. Sites typically present the study to the oncology physicians at the site who then offer participation of the study to patients that they believe may be eligible. Although there may be some selection bias on the part of the physician and/or the patient, this is difficult to control, is not expected to be higher on this study than on other similar clinical trials, and is unlikely to substantially impact results. Participants were not compensated for study participation, although certain trial-related expenses (e.g., hotel rooms, transportation) were reimbursed for some patients.
Ethics oversight	The trial was approved by the institutional review board or independent ethics committee at each participating center.

Note that full information on the approval of the study protocol must also be provided in the manuscript.

Field-specific reporting

Please select the one below that is the best fit for your research. If you are not sure, read the appropriate sections before making your selection.

☒ Life sciences ☐ Behavioural & social sciences ☐ Ecological, evolutionary & environmental sciences

For a reference copy of the document with all sections, see nature.com/documents/nr-reporting-summary-flat.pdf

Life sciences study design

All studies must disclose on these points even when the disclosure is negative.

Sample size	621 patients were included in this prespecified exploratory biomarker analysis.
Data exclusions	Analysis of the CMS2 subgroup was excluded in the analysis of the association between OS and CRC subtypesd due to small subgroup sample size. Putative mutations in TET2 were excluded from further whole exome sequencing analyses as we were unable to verify that these were not germline and/or CHIP mutations. These exclusion criteria were not pre-established.
Replication	Not applicable as this is a clinical trial.
Randomization	For the primary analysis of the BEACON CRC study, the randomization schedule was created and managed by a third-party vendor, and treatments was assigned according to a computerized central randomization list using an interactive web response system (IWRS). For this prespecified exploratory biomarker analysis, randomization was not applicable; nevertheless, we showed that the patients analyzed were representative of those randomly assigned to each arm. .
Blinding	For the primary analysis of the BEACON CRC study, the Sponsor (and their designee trial team) was blinded to treatment group assignments presented in aggregate data summaries. For this prespecified exploratory biomarker analysis, the PFS outcome was assessed by both blinded independent central review and investigators. This is an open-label study.

Reporting for specific materials, systems and methods

We require information from authors about some types of materials, experimental systems and methods used in many studies. Here, indicate whether each material, system or method listed is relevant to your study. If you are not sure if a list item applies to your research, read the appropriate section before selecting a response.

Materials & experimental systems

n/a	Involved in the study
☒	☐ Antibodies
☒	☐ Eukaryotic cell lines
☒	☐ Palaeontology and archaeology
☒	☐ Animals and other organisms
☐	☒ Clinical data
☒	☐ Dual use research of concern
☒	☐ Plants

Methods

n/a	Involved in the study
☒	☐ ChIP-seq
☒	☐ Flow cytometry
☒	☐ MRI-based neuroimaging

Clinical data

Policy information about clinical studies

All manuscripts should comply with the ICMJE guidelines for publication of clinical research and a completed CONSORT checklist must be included with all submissions.

Clinical trial registration	NCT02928224
Study protocol	The redacted BEACON CRC protocol has been previously published (DOI: 10.1056/NEJMoa1908075) and is available at this link: https://www.nejm.org/doi/suppl/10.1056/NEJMoa1908075/suppl_file/nejmoa1908075_protocol.pdf
Data collection	Data were collected at various sites in the United States, Australia, United Kingdom, Europe, and Asia. Patients were recruited between May 2017 and January 2019; the data cutoff for this prespecified exploratory biomarker analyses was August 15, 2019.
Outcomes	Exploratory outcomes of the BEACON CRC study reported here were genomic and transcriptomic correlates of survival outcomes (OS and PFS). Patients were grouped by CRC subtype, individual gene alterations, and gene signatures, based on WTS and WES using their tumor tissue samples at baseline and ctDNA analysis using their blood samples at baseline and EoT, where available. PFS, assessed by blinded independent central review and investigators, was defined as the time from randomization to the earliest documented disease progression or death due to any cause. OS was defined as the time from randomization to death due to any cause.

Plants

Seed stocks	N/A, plants are not used in this study.
Novel plant genotypes	N/A, plants are not used in this study.
Authentication	N/A, plants are not used in this study.
